# Unilaterally Fluorinated Acenes: Synthesis and Solid‐State Properties

**DOI:** 10.1002/anie.202006489

**Published:** 2020-07-15

**Authors:** Philipp E. Hofmann, Matthias W. Tripp, Daniel Bischof, Yvonne Grell, Anna L. C. Schiller, Tobias Breuer, Sergei I. Ivlev, Gregor Witte, Ulrich Koert

**Affiliations:** ^1^ Fachbereich Chemie Philipps-Universität Marburg Hans-Meerwein-Straße 4 35043 Marburg Germany; ^2^ Fachbereich Physik Philipps-Universität Marburg Renthof 7 35032 Marburg Germany

**Keywords:** acenes, electronic structure, fluorinated acenes, solid-state structures, unilateral substitution

## Abstract

The rapid development of organic electronics is closely related to the availability of molecular materials with specific electronic properties. Here, we introduce a novel synthetic route enabling a unilateral functionalization of acenes along their long side, which is demonstrated by the synthesis of 1,2,10,11,12,14‐hexafluoropentacene (**1**) and the related 1,2,9,10,11‐pentafluorotetracene (**2**). Quantum chemical DFT calculations in combination with optical and X‐ray absorption spectroscopy data indicate that the single‐molecule properties of **1** are a connecting link between the organic semiconductor model systems pentacene (PEN) and perfluoropentacene (PFP). In contrast, the crystal structure analysis reveals a different packing motif than for the parent molecules. This can be related to distinct F⋅⋅⋅H interactions identified in the corresponding Hirshfeld surface analysis and also affects solid‐state properties such as the exciton binding energy and the sublimation enthalpy.

## Graphical abstract

**Regioselective functionalization of acenes**: A novel synthetic strategy is introduced that enables a regioselective functionalization of acenes, which is demonstrated using the example of unilaterally substituted fluoroacenes. The packing motif and optoelectronic solid‐state properties of these new materials are compared to the properties of their parental acenes.

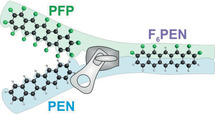

Molecule‐based organic electronics is a rapidly growing field of technology that holds promise for the fabrication of flexible and cost‐effective optoelectronic devices and sensors^1^ and has expressed the need to explore new molecular materials with specific characteristics.^2^ While electronic properties of molecules can be tailored through appropriate design, the optoelectronic properties of molecular solids also depend crucially on the molecular packing and intermolecular coupling.^3^, ^4^ Nowadays, the electronic properties of proposed molecules can be calculated precisely,^5^ whereas their concrete synthesis often remains a big challenge. Predicting crystal structures and packing motifs of such van der Waals bound molecular solids is also difficult since small molecular modifications can induce drastic changes of the packing motifs, leading to significantly altered optoelectronic solid‐state properties.^4^, ^6^, ^7^, ^8^, ^9^ Among the organic semiconductors (OSC), acenes are frequently studied because their aromatic frame enables versatile control of the electronic structure through their topology (e.g. length, branching, etc.) as well as chemical functionalization.^7^, ^10^ In particular, pentacene and its derivatives have become prototypical model systems because they form crystalline films and are sufficiently thermally stable.^11^ Fluorination is a common strategy to modify the electronic properties of organic semiconductors.^12^ While functionalization by fluorinated side groups such as trifluoromethyl leads to a significant change in the molecular shape and thereby affects the packing motif due to steric hindrance,^13^ the molecular shape is not significantly changed by direct fluorination. In this case, the polar C−F bonds directly modify the energy levels of the π‐system and, in the case of perfluorination, cause an inversion of the charge density distribution, as depicted in Figure 1 for pentacene.^14^ So far, only a small number of partially fluoro‐substituted pentacenes have been synthesized, mainly with symmetrical fluorination at the outer rings,^15^ or in the case of asymmetric substitution only at the short molecular sides.^13^, ^16^ The latter molecules have a permanent dipole moment and geometrically combine two smaller aromatic subsystems (one being fluorinated) to one π‐system with a push–pull character. More challenging, however, is the case where all aromatic rings are partially fluorinated by introduction of a unilateral substitution pattern, as this would cause a true mixing of the electronic states of the parental acene and its perfluorinated analogue. However, common synthetic methods based on symmetric cycloadditions cannot be applied here, as they do not allow the control of the regioselectivity of unilateral substitution patterns.

**Figure 1:**
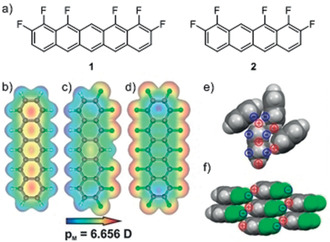
a) Synthetic targets in this study and electrostatic contour plots of b) PEN, c) the newly synthesized unilaterally fluorinated pentacene **1** and d) PFP obtained by DFT. Packing motifs in the crystalline phase of e) PEN and f) **1**, together with molecular charge distribution.

Here, we introduce a novel synthetic strategy that can be applied to realize nearly any kind of substitution. Our new route provides access to a class of unilaterally substituted acenes, which is demonstrated for the case of the unilaterally fluorinated 1,2,10,11,12,14‐hexafluoropentacene (**1**) and the related 1,2,9,10,11‐pentafluorotetracene (**2**). Using DFT‐based electronic structure calculations, X‐ray absorption spectroscopy (NEXAFS), and UV/Vis measurements, we show that **1** exhibits a truly bivalent behavior between PEN and PFP and has a distinct dipole moment of 6.6 D along the molecular M‐axis. Due to the changed electrostatic potential at the molecular rim, a different packing motif stabilized by F⋅⋅⋅H bonds can now be expected, as shown in Figure 1 f, which leads to a significant modification of the solid‐state properties, in particular the exciton binding energy and sublimation enthalpy.

Most known syntheses of symmetrically substituted pentacenes of type **3** use symmetrical intermediates such as **4** and **5** which are accessible by [4+2] cycloadditions (cf. Scheme 1).^13^, ^15^, ^16^ Due to regioselectivity problems within the [4+2] cycloadditions, unilaterally substituted pentacenes **6** require a different approach, with two bonds being formed in subsequent steps. Closure of the central ring via the bond marked in red could be possible from a key intermediate **7**, which should be accessible in a convergent manner from two naphthalene building blocks **8** and **9** by formation of the bond marked in blue.

**Scheme 1:**
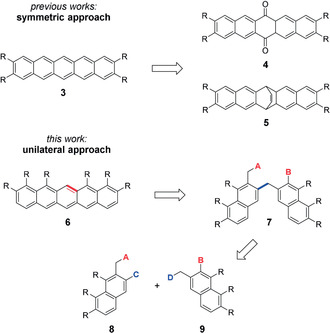
Different synthetic routes to symmetrical pentacenes **3** and unilaterally substituted pentacenes **6**.

The synthesis of unilaterally substituted hexafluoropentacene **1** started from trifluoronaphthol **10** as the common precursor for both building blocks (see Scheme 2).^17^ Triflation of naphthol **10** followed by carbonylative cross‐coupling^18^ gave the methyl ester **11**, which was subjected to an iridium‐catalyzed directed *ortho* C−H borylation to deliver the boronate **12**.^19^ Alternatively, MOM‐protection of naphthol **10** allowed for an *ortho*‐lithiation^20^ leading to aldehyde **13**. Reduction to the corresponding alcohol and Appel reaction resulted in benzylic bromide **14**.

**Scheme 2:**
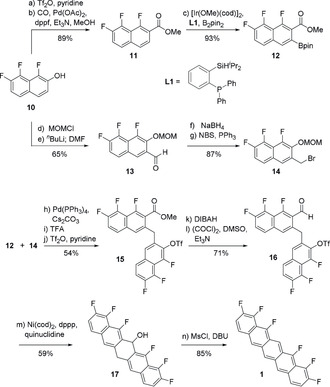
Synthesis of F6‐PEN **1**. Reagents and conditions: a) Tf_2_O (1.2 equiv), pyridine (2.0 equiv), CH_2_Cl_2_, 0 °C, 50 min; b) CO (1 atm), Pd(OAc)_2_ (5 mol %), dppf (10 mol %), Et_3_N (2.0 equiv), DMF/MeOH 9:5, 65 °C, 4.5 h; c) [Ir(OMe)(cod)]_2_ (2.5 mol %), L1 (5.0 mol %), B_2_pin_2_ (1.0 equiv), THF, 75 °C, 26 h; d) NaH (1.5 equiv), MOMCl (1.5 equiv), DMF, rt, 1 h; e) *n*‐BuLi (1.0 equiv), THF/*n*‐pentane 20:9, −78 °C, 6 h; DMF (1.0 equiv), −78 °C to rt, 45 min; f) NaBH_4_ (6.0 equiv), THF, rt, 30 min; g) NBS (2.0 equiv), PPh_3_ (2.0 equiv), CH_2_Cl_2_, 0 °C, 2 h; h) **14** (1.0 equiv.), **12** (1.1 equiv), Pd(PPh_3_)_4_ (3.0 mol %), Cs_2_CO_3_ (3.0 equiv), THF/H_2_O 10:1, 75 °C, 18 h; i) TFA (5.0 equiv), CH_2_Cl_2_, 0 °C to rt, 5.5 h; j) Tf_2_O (1.2 equiv), pyridine (2.8 equiv), CH_2_Cl_2_, 0 °C, 30 min; k) DIBAH (2.5 equiv), THF, 0 °C to rt, 18 h; l) (COCl)_2_ (1.5 equiv), DMSO (3.0 equiv), CH_2_Cl_2_, −78 °C, 30 min; Et_3_N (5.0 equiv), −78 °C, 30 min; rt, 1 h; m) Ni(cod)_2_ (1.0 equiv), dppp (1.2 equiv), quinuclidine (1.0 equiv), toluene, 70 °C, 43 h; n) MsCl (3.0 equiv), DBU (5.0 equiv), CH_2_Cl_2_, 0 °C to rt, 1 h; 40 °C, 2 h. cod=1,5‐cyclooctadiene, DBU=1,8‐diazabicyclo[5.4.0]undec‐7‐ene, DIBAH=diisobutylaluminium hydride, dppf=1,1′‐bis(diphenylphosphino)ferrocene, dppp=1,3‐bis(diphenylphosphino)propane, MOM=methoxymethyl, Ms=methanesulfonyl, NBS=*N*‐bromosuccinimide, pin=pinacolyl, TFA=trifluoroacetic acid, Tf_2_O=trifluoromethanesulfonic anhydride.

A Suzuki coupling of naphthalenes **12** and **14** provided the desired linkage of the two building blocks. Subsequent cleavage of the MOM ether with TFA and installation of a triflate gave methylene‐bridged bisnaphthalene **15**. Reduction of methyl ester and Swern oxidation led to aldehyde **16**. With **16** in hand, we investigated the closure of the central ring next. Using the conditions for a Ni^0^‐catalyzed intramolecular carbonyl Heck reaction^21^ resulted in complete decomposition of the starting material. Decreasing the reaction temperature to 70 °C gave alcohol **17** in low yield, probably as a product of a Barbier‐type reaction instead of a carbonyl Heck reaction (see the Supporting Information). Stoichiometric addition of Ni^0^ reagent in combination with dppp and quinuclidine in toluene at 70 °C provided **17** in good yield. A subsequent mesylation/elimination resulted in the formation of the hexafluoropentacene **1**. ^1^H and ^19^F NMR analysis of **1** was performed in naphthalene‐*d* ^*8*^ at 368 K (95 °C) which showed all the significant proton and the expected fluorine signals, respectively. No fluorine substituent was chosen at C13 in the central ring of **1** because of the known chemical instability of this position which causes a partial defluorination of PFP upon contact with metal substrates.^22^ Following the above described route, related 1,2,9,10,11‐pentafluorotetracene (**2**) was synthesized too (see the Supporting Information).

Next, we report on the physico‐chemical characterization of **1**, considering first the electronic molecular properties. UV/Vis solution spectra yield a HOMO–LUMO gap of 2.11 eV, which is very similar to that of the parent molecules (PEN: 2.13 eV, PFP: 1.99 eV, cf. Figure 2 a). This may appear surprising at first glance, since fluorination is expected to have a notable impact on the molecular orbital energy levels. Accompanying DFT calculations reveal indeed a distinct energetic shift of the frontier orbitals of **1** which are located between those of PEN and PFP (see the Supporting Information). However, as this affects both the HOMO as well as the LUMO, the optical gap is not significantly altered upon partial fluorination, an effect that was previously found also for other aromatic molecules.^23^ Complementary information on the unoccupied states were obtained from NEXAFS spectroscopy. The C1s NEXAFS spectrum (cf. Figure 2 b) exhibits characteristic sharp π* resonances (corresponding to transitions from core levels into unoccupied π orbitals, associated with the LUMO, LUMO+1, … levels) and broad resonances due to transitions into unoccupied σ orbitals. Comparison of the π* region of the differently fluorinated pentacenes (cf. Figure 2 c) shows that the π* resonances of **1** are well described as a superposition of the respective signatures of PEN and PFP, thus demonstrating that it exhibits final states with mixed character of both parent acenes. This conclusion is corroborated by DFT calculations of the frontier orbitals (see the Supporting Information), unveiling **1** as a connecting link between PEN and PFP in terms of its single‐molecule electronic structure.

**Figure 2:**
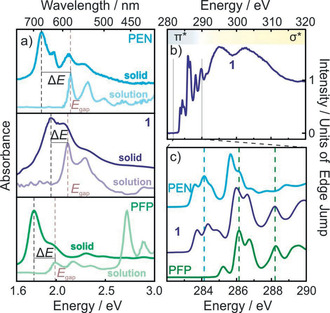
a) UV/Vis spectra of PEN, **1**, and PFP in solution (saturated solution in CH_2_Cl_2_) and as solid films evaporated onto glass substrates with labeled maximum of lowest absorption band (*E*_gap_) and exciton binding energies (Δ*E*). b) C1s NEXAFS spectrum of a thin film of **1** prepared on SiO_2_. c) Comparison of the leading C1s NEXAFS resonances of PEN, **1**, and PFP thin films.

Since for device applications, the solid‐state properties are more relevant, we also carried out UV/Vis absorption measurements on vapor‐deposited molecular films. As depicted in Figure 2 a (dark lines), the absorption spectra reveal a new band below the HOMO–LUMO transition, due to excitonic excitations in the molecular solid. The exciton binding energies (Δ*E*), which can be approximated by the difference between the lowest energy excitations in solution (*E*_gap_) and in the solid, is significantly smaller in **1** (150 meV) compared to PEN (320 meV) and PFP (210 meV), hence reflecting significant difference in the solid‐state electronic properties. To rationalize this effect, a crystal structure analysis is required. Since the low solubility of **1** hampers conventional crystallization from solution, we have instead employed liquid‐assisted crystallization techniques using ionic liquids,^24^ which yields distinct mesoscopic single crystals (cf. Figure 3 b) and thus enabled a crystal structure analysis by X‐ray diffraction (for details see the Supporting Information). While PEN and PFP adopt a herringbone arrangement in their bulk structure,^8^ the novel molecules **1** and **2** crystallize in a criss‐cross packing motif with a dipole parallel packing, as depicted in Figure 3 a (see also the Supporting Information). This contradicts the expected compensation of the dipoles by antiparallel stacking, as observed for acenes partially fluorinated along the short side.^13^

**Figure 3:**
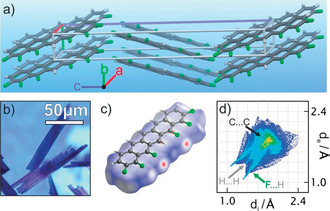
a) Crystal packing of **1**, b) optical micrograph of crystals of **1**, c) Hirshfeld surface, and d) corresponding fingerprint plot of **1**.

A more detailed insight into the intermolecular interactions leading to this packing motif is provided by Hirshfeld surface plots and two‐dimensional fingerprint spectra of **1** (for computational details and comparison with other acenes see the Supporting Information).^25^ Figure 3 c depicts the Hirshfeld surface of **1**, where the red dots mark the regions of strong intermolecular interactions, which can be associated with F⋅⋅⋅H hydrogen bonds. This is further evidenced by the statistical analysis of all atomic contact points between neighboring molecules in the crystal, in the form of a corresponding 2D fingerprint plot as shown in Figure 3 d. In contrast to fingerprint plots of PEN and PFP (see ref. 8 and the Supporting Information), in the case of **1** conspicuous spikes appear, which reflect the F⋅⋅⋅H interactions and provide approximately 45 % of all intermolecular contacts. This strongly indicates that the fluorine–hydrogen interactions govern this packing motif, acting as mediators for the alignment of molecules in the solid state.^26^ Interestingly, also strong C⋅⋅⋅C interactions are observed, which can be ascribed to the π‐stacked like packing along the *b*‐axis and might indicate stronger intermolecular electronic coupling than in pentacene.^7^, ^27^ To quantify the overall strength of intermolecular van der Waals interactions, the sublimation enthalpy of **1** was determined using the Knudsen method (for details see the Supporting Information). Surprisingly, the determined value Δ*H*_sub_=121.3±7.5 kJ mol^−1^ is considerably smaller than the value of PEN (Δ*H*_sub_=156.9±13.6 kJ mol^−1^).^28^ This effect can be rationalized by the different quadrupole moments of these molecules (see the Supporting Information) and demonstrates that additional electrostatic and F⋅⋅⋅H interactions influence the molecular packing motif but at the same time can reduce the dispersion interaction. A similar situation was found previously for oxo species of PEN,^29^ which reveal similar packing motifs as **1**.

In conclusion, we have introduced a novel synthetic route enabling the realization of a new class of unilaterally functionalized acenes, which was demonstrated by the synthesis of pentacene and tetracene derivates with unilateral fluoro substitution patterns. While these compounds reveal single‐molecule properties that can be considered to be intermediate between the non‐ and perfluorinated parent acenes, they also show distinctly different solid‐state properties. This emphasizes the necessity to characterize not only single‐molecule electronic but also solid‐state electronic properties of newly synthesized materials, when such new materials are employed in solid thin‐film devices. The novel synthetic strategy not only provides access to unilateral substitution patterns but is also applicable to various kinds of nonsymmetric substitutions. Further studies to utilize this synthetic route to correlate structural and electronic solid‐state properties of novel partially fluorinated acenes are underway.

## Conflict of interest

The authors declare no conflict of interest.

## Supporting information

As a service to our authors and readers, this journal provides supporting information supplied by the authors. Such materials are peer reviewed and may be re‐organized for online delivery, but are not copy‐edited or typeset. Technical support issues arising from supporting information (other than missing files) should be addressed to the authors.

Supplementary
