# Supplementary material for: Unilaterally Fluorinated Acenes: Synthesis and Solid‐State Properties
Source: Angew Chem Int Ed Engl. 2020 Jul 15;59(38):16501–5. doi: 10.1002/anie.202006489 (PMC7540388; doi:10.1002/anie.202006489)
Supplement: Supplementary file 1 — Supplementary [file ANIE-59-16501-s001.pdf]

Angewandte Chemie
Eine Zeitschrift der Gesellschaft Deutscher Chemiker

Supporting Information

Unilaterally Fluorinated Acenes: Synthesis and Solid-State Properties

Philipp E. Hofmann[+], Matthias W. Tripp[+], Daniel Bischof[+], Yvonne Grell[+], Anna L. C. Schiller, Tobias Breuer, Sergei I. Ivlev, Gregor Witte, and Ulrich Koert**

anie_202006489_sm_miscellaneous_information.pdf

Table of Contents

1. Experimental Characterization Methods **3**
2. Synthetic Conditions **5**
3. Synthesis of Ligand L1 **6**
4. Synthesis of 1,2,10,11,12,14-Hexafluoropentacene **8**
5. Synthesis of 1,2,9,10,11-Pentafluorotetracene **29**
6. Mechanistic Proposal **41**
7. Crystallographic Data **42**
8. Determination of Sublimation Enthalpy **47**
9. Quantum Chemical Calculations **48**
10. Hirshfeld Analysis **52**
11. NMR Spectra of All Compounds **54**
12. References **92**

SUPPORTING INFORMATION

1. Experimental Characterization Methods

Thin film and crystal preparation: The organic thin films were grown under high vacuum conditions by organic molecular beam deposition (OMBD) from aluminium crucibles of resistively heated Knudsen cells. Transparent glass substrates were used for optical measurements. The glass substrates were cleaned by rinsing in ethanol and acetone and heated in vacuum before film deposition. The film growth rates were monitored by a quartz crystal microbalance (QCM) and processed at rates of about 4 Å min^{-1}. Due to the low solubility of the compounds 1,2,10,11,12,14-hexafluoropentacene (F_6PEN, **1**) and 1,2,9,10,11-pentafluoropentacene (F_5TET, **2**), single crystal growth from solution was not possible. Instead single crystals of compounds **1** and **2** were obtained by means of liquid-mediated growth. In that process, which is described more in detail in literature[1-3], a supersaturated solution of the desired acene is maintained in the ionic liquid 1-methyl-3-octylimidazolium-bis(trifluoromethylsulfonyl)amide (Alfa Aesar) and kept at elevated temperatures (40°C for tetracenes, 100°C for pentacenes) while continuously evaporating molecules into the solution. Upon appropriate choice of growth parameters, single crystalline needles of an area of 40µm x 150µm (**1**), respectively 50µm × 300µm (**2**) and a thickness of several microns can be prepared.

Spectroscopy: The optical UV/Vis absorption spectra of the thin films, as well as the solution spectra, have been acquired using an Agilent 8453 spectrometer. The NEXAFS measurements were carried out at the HE-SGM dipole beamline of the synchrotron storage ring BESSY II in Berlin (Germany), which provides linearly-polarized light (polarization factor = 0.91 and an energy resolution at the carbon K-edge of about 300 meV). All NEXAFS-spectra were recorded in partial electron-yield (PEY) mode at a sample orientation of 55° (magic angle) using a channel-plate detector with a retarding field of -150 V. The acquired spectra were normalized by considering the transmission of the beamline and energy-calibrated via a reference signal. All samples for the NEXAFS measurements were prepared without contact to air in order to prevent effects from contaminations. Details on the experimental setup and data evaluation of NEXAFS measurements are provided in literature.[4]

Mass spectroscopy: Mass spectra were recorded by the mass service department of the Philipps-Universität Marburg. HR-ESI & HR-APCI mass spectra were acquired with a LTQ-FT mass spectrometer (THERMO FISCHER SCIENTIFIC). The resolution was set to 100.000. HRS-EI mass spectra were acquired with an MAT95 mass spectrometer (FINNIGAN).

NMR Spectroscopy: NMR-spectra were recorded on a Bruker AVIII HD250, AVII 300, AVIII HD300, AVIII 500 or AVIII HD500 spectrometer at r.t. unless otherwise mentioned. Chemical shifts are reported in ppm with the solvent resonance as internal standard. ^{11}B-NMR-spectra were referenced to external $BF_3{\cdot}OEt_2$, ^{19}F-NMR-spectra were referenced to external $CFCl_3$ and ^{31}P-NMR-spectra were referenced to external H_3PO_4. Unless otherwise noted, all reported ^{19}F-NMR-spectra are decoupled ^{19}F{^{1}H}-measurements. Data are reported as follows: s = singlet, d = doublet, t = triplet, q = quartet, quin = quintet, m = multiplet and combination thereof. Due to the low solubility of compounds **1** & **2** no ^{13}C-NMR spectra were obtained. All correlations of atoms from NMR-spectra of new compounds could be achieved via additionally 2D-NMR-data (COSY-, HSQC- and HMBC-spectra) which is not shown within these SI. For compound **31** the structure could be solved via ^{13}C-data where the exact suppression of each ^{19}F-signal was performed (e.g. ^{13}C{^{19}F at –146.2 ppm} etc.). Melting points were determined on a MP70 (METTLER TOLEDO) using one end closed capillary tubes.

Single crystal X-ray diffraction: X-ray structure analysis of single crystals was carried out using a STOE STADIVARI diffractometer with a microfocus copper X-ray source (Cu-Kα, λ = 1.54186 Å) and a Dectris PILATUS 300K detector. Evaluation and integration of the diffraction data was carried out by using the STOE X-Area software suite[5], and a multi-scan absorption correction was applied. The structures were solved using direct methods (SHELXT) and refined against F^2 (SHELXL).[6-7] Missing atoms were located by difference Fourier synthesis. In case **1** restraints on the C–C and C–F bond lengths were applied as well as on the anisotropic displacement parameters. CCDC 1998747 (**1**) and CCDC 1998748 (**2**) contain the supplementary data for the structure determination. These data can be obtained free of charge from The Cambridge Crystallographic Data Centre via http://www.ccdc.cam.ac.uk/structures.

Powder X-ray diffraction: The powder X-ray pattern of **1** was recorded with a STOE StadiMP diffractometer in Debye-Scherrer geometry using a quartz capillary (Ø 0.7 mm). The diffractometer was operated with Cu-$K_{\alpha 1}$ radiation (1.540598 Å, germanium monochromator) and equipped with a MYTHEN 1K detector. The diffraction pattern was processed using the WinXPOW suite.[8] Profile fitting was done with the Topas-Academic software.[9]

Density measurement: The density of **1** was measured using the automated gas displacement pycnometry system AccuPyc II 1340 (Micromeritics) with a calibrated 0.1 cm^3 sample holder and helium as the displacement gas. A total sample mass of 7.0 mg was used. The number of preliminary purges was set to 30, while the subsequent density measurements were performed 100 times with measurement averaging. The density of the sample was determined to be 1.722(16) g/cm^3 at 295 K.

2. Synthetic Conditions

All non-aqueous reactions were carried out using flame-dried glassware under argon atmosphere. All solvents were distilled by rotary evaporation. THF for non-aqueous reactions was dried with KOH and subsequently distilled from sodium/benzophenone and from Solvona® respectively. Toluene for non-aqueous reactions was distilled from sodium. The $CDCl_3$ used for NMR-measurements was removed from DCl by passage through basic aluminum oxide and degassed three times by freeze-pump-thaw cycles. All commercially available reagents and reactants were used without further purification unless otherwise noted. Reactions were monitored by thin layer chromatography (TLC) using Merck Silica Gel60 F254 and visualized by fluorescence quenching under UV-light. In addition, TLCplates were stained using a cerium sulfate/phosphomolybdic acid stain or a potassium permanganate stain. Chromatographic purification of products was performed on Macherey-Nagel Silica Gel 60 M (0.04-0.063 mm) or Macherey-Nagel Aluminium Oxide 90 (neutral, pH 7 ± 0.5, 0.05-0.2 mm) using a forced flow of eluents. Concentration under reduced pressure was performed by rotary evaporation at 40 °C and appropriate pressure and by exposing to high vacuum at r.t. if necessary.

3. Synthesis Ligand L1

(2-bromophenyl)diphenylphosphane (**20**)

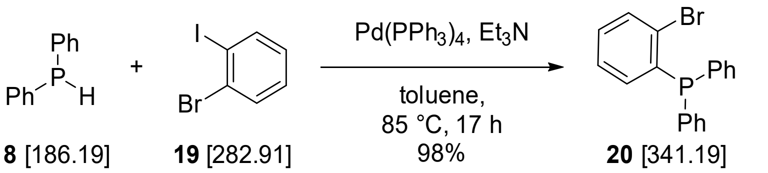

To a stirred solution of $Pd(PPh_3)_4$ (31.0 mg, 0.03 mmol, 0.5 mol%) in toluene (10.0 mL) was added diphenylphosphine (**18**) (0.93 mL, 5.37 mmol, 1.00 eq), 1-bromo-2-iodobenzene (**19**) (0.70 mL, 5.43 mmol, 1.01 eq) and Et_3N (0.93 mL, 6.71 mmol, 1.25 eq) under argon. The resulting mixture was stirred for 17 h at 85 °C. The reaction was allowed to cool to r.t. and then diluted with water (40 mL) and extracted with toluene (6 × 20 mL). The solvent was removed under reduced pressure and the obtained residue was again dissolved in toluene (3 mL) and filtered over a short silica pad (~15 cm), which was eluted with toluene. The solvent was removed under reduced pressure to give **20** (1.79 g, 5.25 mmol, 98%) as white solid. As a precaution, the product was stored under argon. Analytical data was in agreement with the literature.[10-11]

TLC R_f = 0.67 (Pe/EtOAc 5:1).

^{1}H-NMR: 300 MHz, $CDCl_3$; δ (ppm) = 7.62 - 7.58 (m, 1H, H_{arom}), 7.38 - 7.33 (m, 6H, 6 × H_{arom}), 7.32 - 7.36 (m, 4H, 4 × H_{arom}), 7.22 - 7.18 (m, 2H, 2 × H_{arom}), 6.78 - 6.74 (m, 1H, H_{arom}).

^{31}P{^{1}H}-NMR: 101 MHz, $CDCl_3$; δ (ppm) = −5.69 (s, 1P).

(2-(diisopropylsilyl)phenyl)diphenylphosphane (**L1**)

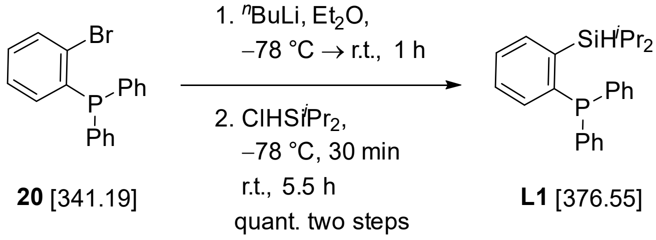

(2-Bromophenyl)diphenylphosphane (**20**, 1.78 g, 5.22 mmol, 1.00 eq) was dissolved in Et_2O (27.5 mL) and cooled to –78 °C before *n*-BuLi (2.5 M in hexane, 2.19 mL, 5.48 mmol, 1.05 eq) was added dropwise under argon. The resulting solution was stirred at this temperature for 30 min before another 30 min at r.t. The mixture was cooled back to –78 °C and chlorodi*iso*propylsilane (0.98 mL, 5.48 mmol, 1.05 eq) was added over 5 min. The reaction was stirred at –78 °C for 30 min, then it was allowed to warm to r.t. and stirred for further 5.5 h. The sus-

pension was filtered over a frit under argon and the precipitate was washed with toluene (2 × 5 mL). Concentration of the solvent *in vacuo* afforded **L1** (1.97 g, 5.22 mmol, quant.) as white solid, which was stored under argon.

TLC R$_f$ = 0.83 (Pe/EtOAc 10:1).

^{1}H-NMR: 500 MHz, $CDCl_3$; δ (ppm) = 7.65 - 7.53 (m, 1H, H_{arom}), 7.37 - 7.31 (m, 8H, 8 × H_{arom}), 7.30 - 7.26 (m, 4H, 4 × H_{arom}), 7.14 - 7.12 (m, 1H, H_{arom}), 4.29 - 4.26 (m, 1H, Si-*H*), 1.43 - 1.36 (m, 2H, 2 × Si-C*H*(CH_3)$_2$), 1.12 (d, 6H, *J* = 7.4 Hz, Si-CH(CH_3)$_2$), 0.90 (d, 6H, *J* = 7.4 Hz, Si-CH(CH_3)$_2$).

^{13}C-NMR: 126 MHz, $CDCl_3$; δ (ppm) = 143.9 (d, *J* = 10.5 Hz, 1C, C_q), 143.6 (s, 1C, C_q), 143.2 (s, 1C, C_q), 138.0 (d, *J* = 11.8 Hz, 1C, C_q), 136.9 (d, *J* = 14.8 Hz, 1C, CH_{arom}), 134.4 (s, 1C, CH_{arom}), 133.8 (d, *J* = 18.7 Hz, 4C, 4 × CH_{arom}), 129.4 (s, 1C, CH_{arom}), 128.5 (s, 2C, 2 × CH_{arom}), 128.5 (d, *J* = 4.0 Hz, 4C, 4 × CH_{arom}), 128.1 (s, 1C, CH_{arom}), 19.5 (s, 2C, Si-CH(*C*H$_3$)$_2$), 19.4 (s, 2C, Si-CH(*C*H$_3$)$_2$), 12.3 (s, 1C, Si-*C*H(CH_3)$_2$), 12.2 (s, 1C, Si-*C*H(CH_3)$_2$).

^{31}P{^{1}H}-NMR: 101 MHz, $CDCl_3$; δ (ppm) = −9.09 (s, 1P).

4. Synthesis of 1,2,10,11,12,14-Hexafluoropentacene

1. ethyl 2-(6-bromo-2,3-difluorophenyl)-2-hydroxyacetate (**22**)

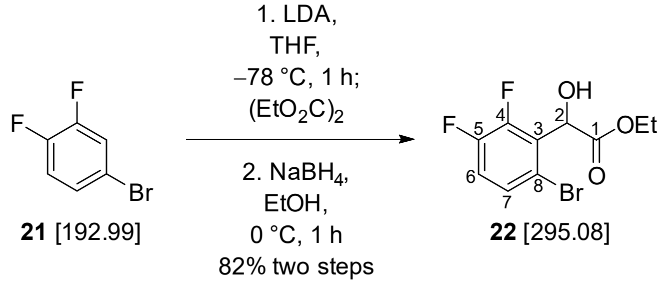

21 [192.99] **22** [295.08]

To a stirred solution of *N*,*N*-diisopropylamine (6.53 mL, 46.4 mmol, 1.05 eq) in THF (80 mL) was added dropwise a solution of *n*-BuLi (1.6 M in hexane, 17.7 mL, 44.2 mmol, 1.00 eq) at 0 °C under argon. The resulting reaction mixture was stirred for 15 min at 0 °C before it was cooled to –78 °C and a solution of difluorobenzene (**21**, 5.00 mL, 44.2 mmol, 1.00 eq) in THF (25 mL) was added slowly. The mixture was stirred for 1 h at –78 °C and then diethyl oxalate (6.04 mL, 44.7 mmol, 1.01 eq) was added dropwise. The solution was stirred for another 15 min at this temperature before EtOH (43 mL) was added followed by $NaBH_4$ (1.69 g, 44.7 mmol, 1.01 eq). The mixture was warmed to 0 °C and stirred for 1 h. The reaction was quenched with sat. aq. NH_4Cl-solution (100 mL) and then stirred for additional 15 min at r.t. The reaction mixture was extracted with EtOAc (3 × 100 mL), the combined organic layers were washed brine (100 mL) and then dried over Na_2SO_4. The solvent was removed under reduced pressure and the crude product was adsorbed onto silica gel and then purified via column chromatography (pentane/EtOAc 10:1 to 8:1) to give **22** (10.7 g, 36.3 mmol, 82%) as light yellow solid. Analytical data was in agreement with the literature.[12]

TLC R_f = 0.30 (Pe/EtOAc 5:1).

m.p.: 42 °C (EtOAc).

^{1}H-NMR: 500 MHz, $CDCl_3$; δ (ppm) = 7.35 (ddd, *J* = 8.9, 4.4, 2.1 Hz, 1H, *H*7), 7.08 (td, *J* = 9.1, 8.4 Hz, 1H, *H*6), 7.61 (d, *J* = 5.3 Hz, 1H, *H*2), 4.30 (q, *J* = 7.2 Hz, 2H, OCH_2), 3.63 (d, *J* = 5.4 Hz, 1H, O*H*), 1.25 (t, *J* = 7.1 Hz, 3H, CH_3).

^{13}C-NMR: 126 MHz, $CDCl_3$; δ (ppm) = 172.0 (s, 1C, *C*1), 150.1 (dd, *J* = 250.3, 13.0 Hz, 1C, *C*5), 149.9 (dd, *J* = 254.1, 13.3 Hz, 1C, *C*4), 128.7 (dd, *J* = 6.0, 4.2 Hz, 1C, *C*7), 128.5 (d, *J* = 12.0 Hz, 1C, *C*3), 118.6 - 118.4 (m, 2C, *C*6 & *C*8), 69.0-68.9 (m, 1C, *C*2), 63.0 (s, 1C, OCH_2), 14.1 (s, 1C, CH_3).

^{19}F-NMR: 283 MHz, $CDCl_3$; δ (ppm) = −136.6 (d, *J* = 19.9 Hz, 1F), −137.3 (d, *J* = 20.0 Hz, 1F).

2. ethyl 2-(6-bromo-2,3-difluorophenyl)-2-fluoroacetate (**23**)

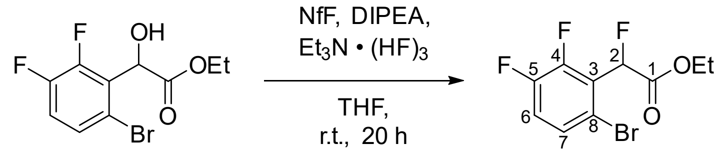

22 [295.08] **23** [297.07]

To a stirred solution of difluorobenzene **22** (9.62 g, 32.6 mmol, 1.00 eq) in THF (130 mL) was added DIPEA (19.4 mL, 114 mmol, 3.50 eq), NfF (8.78 mL, 48.9 mmol, 1.50 eq) and $NEt_3 \cdot (HF)_3$ (5.42 mL, 32.6 mmol, 1.00 eq) under argon. The resulting solution was stirred at r.t. for 20 h. The reaction was quenched with sat. aq. $NaHCO_3$-solution (100 mL) and the aqueous layer was extracted with EtOAc (3 × 100 mL). The combined extracts were washed with brine (100 mL), dried over Na_2SO_4 and the solvent was removed under reduced pressure. The crude product was adsorbed onto silica gel and then purified by column chromatography (pentane/EtOAc 10:1) to give **23** (9.05 g, 30.5 mmol, 94%) as yellow oil. Analytical data was in agreement with the literature.[12]

TLC R_f = 0.43 (Pe/EtOAc 10:1).

^{1}H-NMR: 500 MHz, $CDCl_3$; δ (ppm) = 7.41 (dddd, *J* = 9.0, 4.4, 2.1, 1.1 Hz, 1H, *H*7), 7.20-7.14 (m, 1H, *H*6), 6.23 (d, *J* = 45.7 Hz, 1H, *H*2), 4.33 (q, *J* = 7.1 Hz, 2H, OC*H*$_2$), 1.30 (t, *J* = 7.2 Hz, 3H, C*H*$_3$).

^{13}C-NMR: 126 MHz, $CDCl_3$; δ (ppm) = 167.1 (dd, *J* = 25.6, 0.5 Hz, 1C, *C*1), 150.2 (ddd, *J* = 257.3, 14.3, 3.0 Hz, 1C, *C*5), 150.0 (ddd, *J* = 251.6, 13.0, 2.3 Hz, 1C, *C*4), 129.0 - 128.9 (m, 1C, *C*7), 124.8 (dd, *J* = 18.8, 12.3 Hz, 1C, *C*3), 120.1 - 120.0 (m, 1C, *C*6), 119.0-118.9 (m, 1C, *C*8), 84.5 (dt, *J* = 189.6, 2.5 Hz, 1C, *C*2), 62.7 (s, 1C, O*C*H$_2$), 14.2 (s, 1C, *C*H$_3$).

^{19}F-NMR: 283 MHz, $CDCl_3$; δ (ppm) = −134.1 (dd, *J* = 20.0, 5.5 Hz, 1F, *F*4), −136.6 (d, *J* = 19.9 Hz, 1F, *F*5), −182.8 (d, *J* = 5.0 Hz, 1F, *F*2).

3. ethyl 2-(2,3-difluoro-6-vinylphenyl)-2-fluoroacetate (**24**)

VinylBF_3K (1.62 g, 12.1 mmol, 1.20 eq), K_2CO_3 (4.19 g, 30.3 mmol, 3.00 eq), PPh_3 (397 mg, 1.52 mmol, 0.15 eq) and $PdCl_2$ (90 mg, 0.51 mmol, 0.05 eq) were suspended in THF (6.2 mL) and degassed H_2O (2.00 mL) under argon. Difluorobenzene **23** (3.00 g, 10.1 mmol, 1.00 eq) was dissolved in THF (12.0 mL) under argon and was added. The suspension was again degassed (3×) and then stirred at 85 °C for 18 h (full conversion with monitored with GC-MS because the starting material and the product have the same R_f-values). The reaction mixture was allowed to cool to r.t. before 2 M HCl (25 mL) was added. The aqueous layer was extracted with EtOAc (3 × 20 mL). The combined extracts were washed with brine (20 mL), dried over Na_2SO_4 and the solvent was removed under reduced pressure. The crude product was adsorbed onto silica gel and then purified via column chromatography (pentane/EtOAc 20:1 to 18:1) to give **24** (2.17 g, 8.88 mmol, 88%) as colorless oil. Attention don't dry the product longer than 15 min in high vacuum, because it is volatile. Analytical data was in agreement with the literature.[12]

TLC R_f = 0.43 (Pe/EtOAc 10:1).

^{1}H-NMR: 500 MHz, $CDCl_3$; δ (ppm) = 7.29-7.27 (m, 1H, *H*7), 7.23-7.18 (m, 1H, *H*6), 6.96 (ddd, *J* = 17.2, 11.0, 1.9 Hz, 1H, *H*9), 6.19 (d, *J* = 46.2 Hz, 1H, *H*2), 5.63 (d, *J* = 17.3 Hz, 1H, H_{trans}10), 5.42 (d, *J* = 11.0 Hz, 1H, H_{cis}10), 4.32-4.22 (m, 2H, OCH_2), 1.25 (t, *J* = 7.2 Hz, 3H, CH_3).

^{13}C-NMR: 126 MHz, $CDCl_3$; δ (ppm) = 167.9 (dd, *J* = 26.7, 1.6 Hz, 1C, *C*1), 149.8 (ddd, *J* = 249.5, 13.5, 0.7 Hz, 1C, *C*5), 149.2 (ddd, *J* = 251.3, 13.7, 4.3 Hz, 1C, *C*4), 135.6 (d, *J* = 4.0 Hz, 1C, *C*8), 132.2 - 132.1 (m, 1C, *C*9), 122.7 - 122.6 (m, 1C, *C*7), 121.4 (dd, *J* = 19.2, 9.7 Hz, 1C, *C*3), 119.4 - 119.4 (m, 1C, *C*10), 118.8 - 118.7 (m, 1C, *C*6), 81.8 (ddd, *J* = 186.4, 6.2, 2.6 Hz, 1C, C2), 62.4 (s, 1C, OCH_2), 14.1 (s, 1C, CH_3).

^{19}F-NMR: 283 MHz, $CDCl_3$; δ (ppm) = −137.8 (d, *J* = 20.6 Hz, 1F, *F*5), −140.7 (dd, *J* = 20.6, 2.8 Hz, 1F, *F*4), −181.8 (d, *J* = 2.6 Hz, 1F, *F*2).

4. 1,7,8-trifluoronaphthalen-2-yl acetate (**25**)

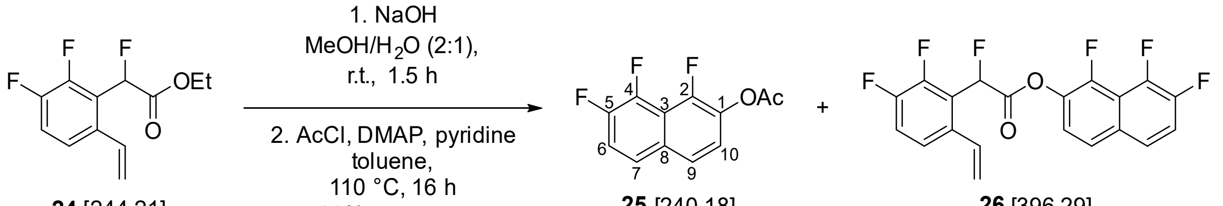

24 [244.21] **25** [240.18] **26** [396.29]

To a stirred solution of styrene **24** (500 mg, 2.05 mmol, 1.00 eq) in MeOH (4.00 mL) and H_2O (2.00 mL) was added NaOH (205 mg, 5.12 mmol, 2.50 eq) under argon. The resulting solution was stirred for 1.5 h at r.t. before it was quenched with 2 M HCl (6 mL). The aqueous layer was extracted with Et_2O (3 × 15 mL), the combined extracts were washed with brine (10 mL) and dried over Na_2SO_4. Removal of the solvent under reduced pressure afforded the corresponding acid as white solid, which was used in the next step without further purification.

The acid (443 mg) was suspended in toluene (10.0 mL) and DMAP (25.0 mg, 0.21 mmol, 0.10 eq), pyridine (0.50 mL, 6.14 mmol, 3.00 eq) and acetyl chloride (0.44 mL, 6.14 mmol, 3.00 eq) were added sequentially under argon. The resulting mixture was heated to 120 °C for 15 h before it was allowed to cool to r.t. and 1 M HCl (6 mL) was added. The aqueous layer was extracted with EtOAc (3 × 15 mL). The extracts were washed with brine (10 mL), dried over Na_2SO_4 and the solvent was removed under reduced pressure. The crude product was adsorbed onto silica gel and then purified by column chromatography (toluene) to give naphthalene **25** (433 mg, 1.80 mmol, 88%) as white solid. Analytical data was in agreement with the literature.[12]

TLC R$_f$ = 0.48 (Pe/EtOAc 5:1).

m.p.: 118 °C (EtOAc).

^{1}H-NMR: 500 MHz, $CDCl_3$; δ (ppm) = 7.61-7.56 (m, 2H, *H7* & *H*9), 7.34 (td, *J* = 9.6, 7.1 Hz, 1H, *H*6), 7.24 (dd, *J* = 8.9, 6.8 Hz, 1H, *H*10), 2.41 (s, 3H, C*H*$_3$).

^{13}C-NMR: 126 MHz, $CDCl_3$; δ (ppm) = 168.4 (s, 1C, *C*O$_2$Me), 148.0 (ddd, *J* = 258.9, 6.7, 2.0 Hz, 1C, *C*4), 147.9 (ddd, *J* = 248.2, 11.8, 1.8 Hz, 1C, *C*2), 144.1 (ddd, *J* = 257.8, 14.7, 1.6 Hz, 1C, *C*5), 135.6 - 135.5 (m, 1C, *C*1), 130.7-130.6 (m, 1C, *C*8), 124.4 - 124.3 (m, 1C, *C*7), 124.2 - 124.1 (m, 1C, *C*9), 122.4 - 122.3 (m, 1C, *C*10), 117.7 (dd, *J* = 21.2, 1.7 Hz, 1C, *C*6), 116.2-116.0 (m, 1C, *C*3), 20.7 (s, 1C, *C*H$_3$).

^{19}F-NMR: 283 MHz, $CDCl_3$; δ (ppm) = −135.1 (dd, *J* = 52.7, 4.9 Hz, 1F, *F*5), −139.9 (dd, *J* = 17.4, 4.7 Hz, 1F, *F*2), −145.9 (dd, *J* = 52.6, 17.2 Hz, 1F, *F*4).

5. 1,7,8-trifluoronaphthalen-2-ol (**10**)

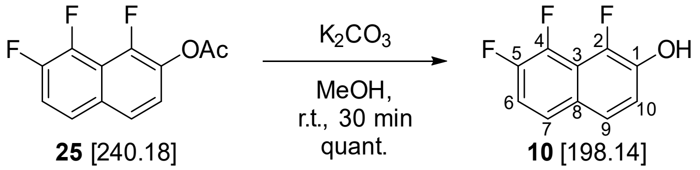

To a stirred solution of acetate **25** (3.52 g, 14.6 mmol, 1.00 eq) in MeOH (195 mL) was added K_2CO_3 (4.37 g, 31.5 mmol, 2.15 eq) under argon and the resulting suspension was stirred for 1.5 h at r.t. The reaction was quenched with sat. aq. NH_4Cl-solution (150 mL) and extracted with EtOAc (4 × 100 mL). The combined organic layers were washed with brine (10 mL), dried over Na_2SO_4 and the solvent was removed under reduced pressure to give naphthol **10** (2.89 g, 14.6 mmol, quant.) as white solid, which could be used without further purification. Analytical data was in agreement with the literature.[12]

TLC R_f = 0.27 (toluene).

m.p.: 107 °C (EtOAc).

^{1}H-NMR: 300 MHz, $CDCl_3$; δ (ppm) = 7.54-7.47 (m, 2H, *H*7 & *H*9), 7.25-7.15 (m, 2H, *H*6 & *H*10), 5.48 (s, 1H, O*H*).

^{13}C-NMR: 75 MHz, $CDCl_3$; δ (ppm) = 148.1 (ddd, *J* = 246.9, 11.8, 1.8 Hz, 1C, *C*5), 143.3 (ddd, *J* = 241.8, 6.9, 1.7 Hz, 1C, *C*2 or *C*4), 143.1 (ddd, *J* = 254.3, 14.8, 1.7 Hz, 1C, *C*2 or *C*4), 141.5 (dd, *J* = 13.6, 2.3 Hz, 1C, *C*1), 127.4 - 127.3 (m, 1C, *C*8), 124.8 - 124.6 (m, 1C, *C*9), 124.5 - 124.3 (m, 1C, *C*7), 118.5 - 118.4 (m, 1C, *C*10), 115.9 - 115.6 (m, 1C, *C*3), 115.3 (dd, *J* = 21.2, 1.5 Hz, 1C, *C*6).

^{19}F-NMR: 283 MHz, $CDCl_3$; δ (ppm) = −139.9 (dd, *J* = 17.0, 5.1 Hz, 1F, *F*2), −147.9 (dd, *J* = 47.1, 17.8 Hz, 1F, *F*4), −151.4 (dd, *J* = 47.0, 4.9 Hz, 1F, *F*5).

6. 1,7,8-trifluoronaphthalen-2-yl trifluoromethanesulfonate (**27**)

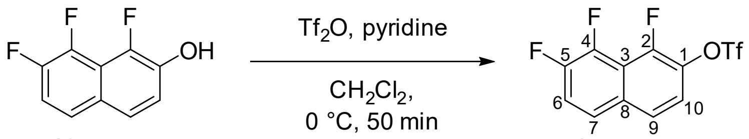

10 [198.14] **27** [330.20]

To a stirred solution of naphthol **10** (664 mg, 3.35 mmol, 1.00 eq) and pyridine (0.54 mL, 6.70 mmol, 2.00 eq) in CH_2Cl_2 (13.2 mL) was added dropwise Tf_2O (0.68 mL, 4.02 mmol, 1.20 eq) at 0 °C under argon. The mixture was stirred for 50 min at 0 °C before it was diluted with Et_2O (5 mL) and poured into 1 m HCl (15 mL). The aqueous layer was extracted with Et_2O (3 × 10 mL) and the combined extracts were washed with sat. aq. $NaHCO_3$-solution (2 × 10 mL) and brine (10 mL). After drying over Na_2SO_4, the solvent was removed under reduced pressure and the residue was adsorbed onto silica gel. Purification by column chromatography (pentane/EtOAc 20:1 to 15:1) gave triflate **27** (1.08 g, 3.28 mmol) as white solid in 98% yield.

TLC R_f = 0.53 (Pe/EtOAc 5:1).

m.p.: 46 °C (EtOAc).

^{1}H-NMR: 500 MHz, $CDCl_3$; δ (ppm) = 7.70 - 7.64 (m, 2H, *H7* & *H*9), 7.47 (td, *J* = 9.4, 7.1 Hz, *H*6), 7.41 (d, *J* = 9.1, 6.7 Hz, 1H, *H*10).

^{13}C-NMR: 126 MHz, $CDCl_3$; δ (ppm) = 148.6 (ddd, *J* = 246.5, 6.9, 1.9 Hz, 1C, *C*2), 148.4 (ddd, *J* = 250.5, 11.6, 1.8 Hz, 1C, *C*5), 144.3 (ddd, *J* = 259.8, 14.9, 1.9 Hz, 1C, *C*4), 134.3 - 134.2 (m, 1C, *C*1), 131.5 - 131.4 (m, 1C, *C*8), 125.0 - 124.9 (m, 1C, *C*9), 124.8 - 124.7 (m, 1C, *C*7), 120.8 (s, 1C, *C*10), 119.5 (dd, *J* = 21.2, 1.7 Hz, 1C, *C*6), 118.8 (q, *J* = 320.8 Hz, 1C, CF_3), 116.0 (td, *J* = 9.3, 1.8 Hz, 1C, *C*3).

^{19}F-NMR: 283 MHz, $CDCl_3$; δ (ppm) = −73.9 (d, *J* = 5.3 Hz, 3F, CF_3), −132.7 (dqui, *J* = 52.8, 5.2 Hz 1F, *F*5), −137.7 (dd, *J* = 17.1, 5.3 Hz, 1F, *F*2), −144.3 (dd, *J* = 53.0, 17.1 Hz, 1F, *F*4).

IR: neat, $\tilde{\nu}$(cm^{-1}) = 1633 (m), 1516 (w), 1483 (m), 1456 (w), 1420 (m), 1377 (w), 1355 (m), 1269 (w), 1213 (s), 1163 (w), 1130 (m), 1063 (m), 1020 (w), 961 (w), 877 (m), 831 (s), 813 (w), 780 (w), 749 (m), 698 (w), 675 (m), 646 (m), 615 (s), 596 (w), 583 (w), 515 (w), 494 (m), 433 (w).

HRMS: EI(+); *m/z* calcd. for $C_{11}H_4F_6O_3S_1$ $[M]^+$: 329.97853, found: 329.97757.

7. methyl 1,7,8-trifluoro-2-naphthoate (**11**)

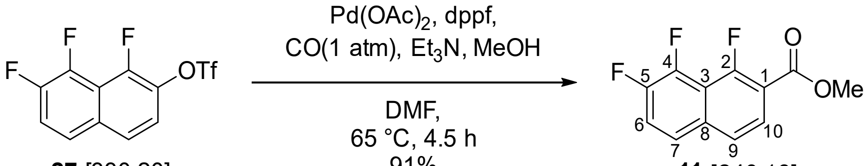

27 [330.20] **11** [240.18]

Triflate **27** (126 mg, 0.38 mmol, 1.00 eq), dppf (21.0 mg, 0.04 mmol, 10 mol%) and $Pd(OAc)_2$ (4.3 mg, 0.02 mmol, 5 mol%) were evacuated and refilled with argon (3×) before DMF (0.63 mL), MeOH (0.35 mL) and Et_3N (0.11 mL, 0.76 mmol, 2.00 eq) were added. The resulting solution was carefully evacuated and refilled with CO (3×) under vigorous stirring, sealed and then heated to 65 °C for 4.5 h. The reaction was allowed to cool to r.t., quenched with water (2 mL) and extracted with Et_2O (3 × 10 mL). The extracts were combined and washed with 1 M HCl (10 mL), sat. aq. $NaHCO_3$-solution (10 mL) and brine (10 mL) and dried over Na_2SO_4. The solvent was removed under reduced pressure, the crude product was adsorbed onto silica gel and then purified by column chromatography (pentane/EtOAc 15:1 to 14:1 to 13:1) to give **11** (83.0 mg, 0.35 mmol, 91%) as white solid.

TLC R_f = 0.28 (Pe/EtOAc 10:1).

m.p.: 116 °C (EtOAc).

^{1}H-NMR: 500 MHz, $CDCl_3$; δ (ppm) = 7.88 (dd, *J* = 8.7, 6.5 Hz, 1H, *H*10), 7.63 - 7.59 (m, 2H, *H*7 & *H*9), 7.48 (td, *J* = 9.4, 7.1 Hz, 1H, *H*6), 4.00 (s, 3H, C*H*$_3$).

^{13}C-NMR: 126 MHz, $CDCl_3$; δ (ppm) = 165.0 (d, *J* = 3.0 Hz, 1C, *C*O$_2$Me), 158.0 (ddd, *J* = 273.6, 6.6, 2.2 Hz, 1C, *C*4), 148.0 (ddd, *J* = 248.6, 12.2, 1.6 Hz, 1C, *C*2), 145.3 (ddd, *J* = 261.3, 14.3, 1.4 Hz, 1C, *C*5), 134.8 - 134.8 (m, 1C, *C*8), 126.9 - 126.8 (m, 1C, *C*10), 124.2 - 124.1 (m, 1C, *C*7), 123.4 - 123.4 (m, 1C, *C*9), 120.2 (dd, *J* = 21.0, 1.6 Hz, 1C, *C*6), 115.5 (ddd, *J* = 12.6, 8.0, 1.4 Hz, 1C, *C*3), 115.2 - 115.1 (m, 1C, *C*1), 52.8 (s, 1C, O*C*H$_3$).

^{19}F-NMR: 283 MHz, $CDCl_3$; δ (ppm) = −111.4 (dd, *J* = 66.7, 4.6 Hz, 1F, *F*5), −139.2 (dd, *J* = 17.5, 5.1 Hz, 1F, *F*2), −142.6 (dd, *J* = 66.9, 17.1 Hz, 1F, *F*4).

IR: neat, ṽ(cm^{-1}) = 3039 (w), 2958 (w), 2920 (w), 2850 (w), 1718 (s), 1646 (w), 1613 (m), 1581 (w), 1510 (w), 1438 (m), 1357 (s), 1282 (w), 1261 (s), 1218 (m), 1201 (w), 1137 (m), 1045 (m), 1024 (w), 950 (m), 846 (m), 809 (m), 751 (m), 732 (m), 673 (m), 656 (w), 608 (w), 596 (m), 561 (w), 444 (w), 425 (w).

HRMS: EI(+); *m/z* calcd. for $C_{12}H_7F_3O_2$ $[M]^+$: 240.03981, found: 240.03938.

8. methyl 1,7,8-trifluoro-3-(4,4,5,5-tetramethyl-1,3,2-dioxaborolan-2-yl)-2-naphthoate (**12**)

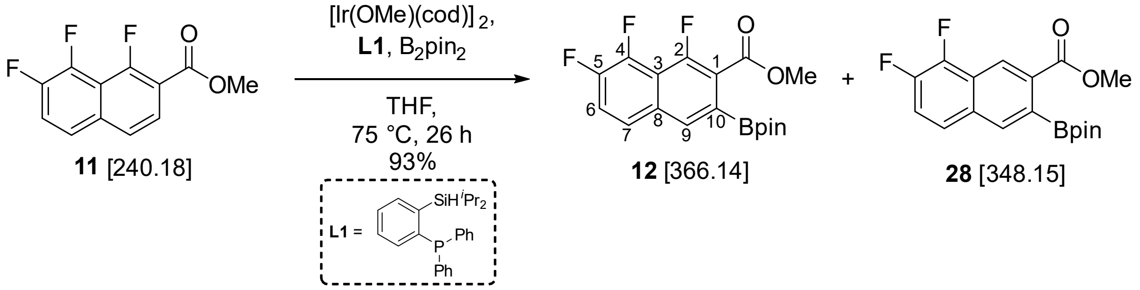

11 [240.18] **12** [366.14] **28** [348.15]

$[Ir(OMe)(cod)]_2$ (10.3 mg, 16 µmol, 2.5 mol%) and **L1** (11.8 mg, 31 µmol, 5.0 mol%) were dissolved in THF (0.63 mL) and stirred for 5 min at r.t. under argon before B_2pin_2 (159 mg, 625 µmol, 1.00 eq) and ester **11** (150 mg, 625 µmol, 1.00 eq) were added sequentially. The reaction mixture was degassed (3×) and then heated to 75 °C for 26 h. The reaction was allowed to cool to r.t. and was then diluted with EtOAc (5 mL) and poured into sat. aq. NH_4Cl-solution (20 mL). The aqueous layer was extracted with EtOAc (3 × 15 mL). The combined extracts were washed with brine (10 mL), dried over Na_2SO_4 and filtered over a short plug of celite, which was rinsed with EtOAc. Since the product decomposed on silica, the solvent was removed under reduced pressure to give the crude product **12** (283 mg, 93% yield det. by NMR, 75wt%) as yellow solid, which was used without further purification. The main impurity consists of pinacolborane, which is generated during the reaction, as well as F_2-compound **28**.

TLC R_f = 0.44 (Pe/EtOAc 5:1).

m.p.: 134 °C decomposition (EtOAc).

^{1}H-NMR: 500 MHz, $CDCl_3$; δ (ppm) = 7.94 (d, *J* = 2.2 Hz, 1H, *H*9), 7.64 - 7.61 (m, 1H, *H*7), 7.44 (td, *J* = 9.5, 7.0 Hz, 1H, *H*6), 3.98 (s, 3H, C*H*3), 1.38 (s, 12H, 4 × C*H*3).

^{13}C-NMR: 126 MHz, $CDCl_3$; δ (ppm) = 166.7 (s, 1C, *C*O2Me), 155.0 (ddd, *J* = 265.5, 6.6, 1.5 Hz, 1C, *C*2), 148.5 (ddd, *J* = 249.6, 12.0, 1.4 Hz, 1C, *C*5), 144.8 (ddd, *J* = 260.2, 14.4, 1.5 Hz, 1C, *C*4), 133.3 - 133.3 (m, 1C, *C*8), 130.6 - 130.5 (m, 1C, *C*9), 128.1 (brs, 1C, *C*10), 124.8 - 124.7 (m, 1C, *C*7), 120.8 (d, *J* = 12.9 Hz, 1C, C1), 119.5 (dd, *J* = 21.0, 1.4 Hz, 1C, *C*6), 116.0 (ddd, *J* = 12.9, 8.3, 1.7 Hz, 1C, *C*3), 84.7 (s, 2C, 2 × *C*(CH3)2), 53.0 (s, O*C*H3), 25.0 (s, 4C, 4 × *C*H3).

^{19}F-NMR: 283 MHz, $CDCl_3$; δ (ppm) = −118.0 (dd, *J* = 59.8, 3.9 Hz, 1F, *F*5), −137.9 (dd, *J* = 16.9, 5.2 Hz, 1F, *F*2), −143.6 (dd, *J* = 59.8, 16.9 Hz, 1F, *F*4).

^{11}B{^{1}H}-NMR: 161 MHz, $CDCl_3$; δ (ppm) = 30.4 (brs, 1B, *B*pin).

IR: neat, ṽ(cm^{-1}) = 3411 (w), 2980 (w), 2931 (w), 1697 (s), 1645 (w), 1611 (w), 1575 (w), 1475 (s), 1445 (m), 1378 (m), 1344 (w), 1318 (w), 1290 (s), 1261 (w), 1214 (w), 1190 (w), 1171 (w), 1138 (s), 1107 (w), 1053 (s), 962 (m), 915 (w), 888 (m), 850 (s), 807 (m), 792 (w), 751 (m), 706 (m), 667 (m), 616 (w), 597 (m), 567 (w), 520 (w), 493 (w), 472 (w), 427 (w).

HRMS: EI(+); *m/z* calcd. for $C_{18}H_{18}B_1F_3O_4$ $[M]^+$: 366.12535, found: 366.12603.

9. 1,2,8-trifluoro-7-(methoxymethoxy)naphthalene (**29**)

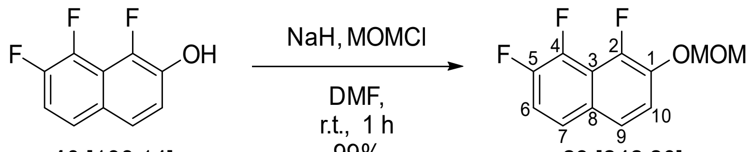

10 [198.14] **29** [242.20]

To a stirred solution of naphthol **10** (900 mg, 4.54 mmol, 1.00 eq) in DMF (2.00 mL) was added sodium hydride (272 mg, 6.81 mmol, 1.50 eq) under argon and the resulting solution was stirred at room temperature for 30 min. The reaction mixture was cooled to 0 °C and MOMCl (0.52 mL, 6.81 mmol, 1.50 eq) was added dropwise. The reaction mixture was stirred for another 30 min at 0 °C, then water (6 mL) was added and the aqueous layer was extracted with ethyl acetate (3 × 5 mL). The combined extracts were washed with brine (5 mL), then dried over Na_2SO_4. The solvent was removed under reduced pressure, the crude product was adsorbed onto silica gel and then purified by column chromatography (pentane/EtOAc 10:1) to give **29** (1.1 g, 4.49 mmol) as white solid in 99% yield.

TLC R_f = 0.38 (Pe/EtOAc 10:1).

m.p.: 68 °C (CH_2Cl_2).

^{1}H-NMR: 500 MHz, $CDCl_3$; δ (ppm) = 7.56 - 7.51 (m, 2H, *H*7 & *H*9), 7.41 (dd, *J* = 8.5, 7.8 Hz, 1H, *H*10), 7.27 - 7.22 (m, 1H, *H*6), 5.31 (s, 2H, OC*H*$_2$), 3.58 (s, 3H, OC*H*$_3$).

^{13}C-NMR: 126 MHz, $CDCl_3$; δ (ppm) = 147.9 (ddd, *J* = 246.9, 11.9, 1.8 Hz, 1C, *C*5), 146.7 (ddd, *J* = 253.8, 6.8, 1.9 Hz, 1C, *C*2), 143.7 (ddd, *J* = 256.4, 14.7, 1.5 Hz, 1C, *C*4), 142.3 - 142.2 (m, 1C, *C*1), 128.5 - 128.4 (m, 1C, *C*8), 124.3 - 124.1 (m, 2C, *C*7 & *C*9), 119.2 - 119.2 (m, 1C, *C*10), 116.5 - 116.3 (m, 1C, *C*3), 116.1 (dd, *J* = 21.3, 1.8 Hz, 1C, *C*6), 96.5 (d, *J* = 1.9 Hz, 1C, O*C*H$_2$), 56.8 (s, 1C, O*C*H$_3$).

^{19}F-NMR: 283 MHz, $CDCl_3$; δ (ppm) = −139.9 (dd, *J* = 17.0, 5.2 Hz, 1F, *F*2), −140.9 (dd, *J* = 52.7, 4.5 Hz, 1F, *F*5), −146.4 (dd, *J* = 53.3, 16.9 Hz, 1F, *F*4).

IR: neat, ṽ(cm^{-1}) = 3070 (w), 2973 (w), 2913 (w), 2843 (w), 2330 (w), 2063 (w), 1885 (w), 1647 (w), 1630 (m), 1515 (m), 1482 (w), 1458 (s), 1411 (w), 1350 (s), 1311 (w), 1255 (s), 1191 (m), 1150 (m), 1133 (w), 1100 (m), 1071 (s), 1022 (s), 968 (s), 913 (m), 826 (s), 786 (w), 763 (w), 682 (s), 618 (m), 591 (m), 557 (w), 541 (w), 477 (w), 437 (w), 408 (w).

HRMS: EI(+); *m/z* calcd. for $C_{12}H_9F_3O_2$ $[M]^+$: 242.05546, found: 242.05647.

10. 4,5,6-trifluoro-3-(methoxymethoxy)-2-naphthaldehyde (**13**)

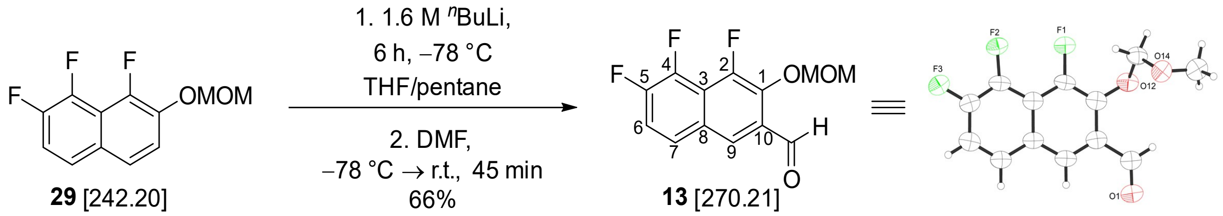

To a stirred solution of MOM-compound **29** (100 mg, 0.41 mmol, 1.00 eq) in THF (0.80 mL) and pentane (0.35 mL) was added *n*-BuLi (0.17 mL, 0.41 mmol, 1.00 eq) dropwise at –78 °C under argon over 15 min. The reaction mixture was stirred for 6 h at –78 °C and after adding DMF (0.03 mL, 0.41 mmol, 1.00 eq) the mixture was stirred for 45 min while it was allowed to warm to room temperature. To the reaction mixture was added sat. aq. NH_4Cl-solution (1 mL) and the aqueous layer was extracted with ethyl acetate (3 × 5 mL). The combined extracts were washed with brine (5 mL), then dried over Na_2SO_4. The solvent was removed under reduced pressure, the crude product was adsorbed onto silica gel and then purified by column chromatography (pentane/ethyl acetate, 15:1 to 12:1 to 10:1) to give **13** (74 mg, 0.27 mmol) as white solid in 66% yield. To proof the correct regioisomer aldehyde **13** was crystallized by dissolving 50 mg sample in EtOAc and slow diffusion of pentane into the sample at 0 °C (see above).

TLC R_f = 0.52 (Pe/EtOAc 5:1).

m.p.: 101 °C (CH_2Cl_2).

^{1}H-NMR: 500 MHz, $CDCl_3$; δ (ppm) = 10.50 (s, 1H, C*H*O), 8.18 (t, *J* = 1.5 Hz, 1H, *H*9), 7.74 - 7.70 (m, 1H, *H*7), 7.37 (td, *J* = 9.5, 7.0 Hz, 1H, *H*6), 5.40 (d, *J* = 1.0 Hz, 2H, OCH_2), 3.63 (s, 3H, OCH_3).

^{13}C-NMR: 126 MHz, $CDCl_3$; δ (ppm) = 188.9 (d, *J* = 3.0 Hz, 1C, *C*HO), 149.6 (ddd, *J* = 252.0, 12.0, 2.0 Hz, 1C, *C*5), 148.8 (ddd, *J* = 257.0, 7.0, 2.0 Hz, 1C, *C*2), 143.6 (ddd, *J* = 258.2, 14.8, 1.8 Hz, 1C, *C*4), 142.1 (d, *J* = 10.0 Hz, 1C, *C*1), 129.1 - 129.0 (m, 1C, *C*10), 127.9 - 127.8 (m, 1C, *C*8), 126.8 - 126.7 (m, 1C, *C*7), 125.7 - 125.7 (m, 1C, *C*9), 119.3 - 119.1 (m, 1C, *C*3), 117.9 (dd, *J* = 21.6, 1.5 Hz, *C*6), 100.3 (d, *J* = 8.0 Hz, O*C*H_2), 58.1 - 58.1 (m, O*C*H_3).

^{19}F-NMR: 283 MHz, $CDCl_3$; δ (ppm) = –134.3 (dd, *J* = 16.9, 5.2 Hz, 1F, *F*2), –136.1 (dd, *J* = 55.9, 5.2 Hz, 1F, *F*5), –144.9 (dd, *J* = 54.6, 16.9 Hz, 1F, *F*4).

IR: neat, ṽ(cm^{-1}) = 3070 (w), 2973 (w), 2913 (w), 2843 (w), 2330 (w), 2063 (w), 1885 (w), 1647 (w), 1630 (m), 1515 (m), 1482 (w), 1458 (s), 1411 (w), 1350 (s), 1311 (w), 1255 (s), 1191 (m), 1150 (m), 1133 (w), 1100 (m), 1071 (s), 1022 (s), 968 (s), 913 (m), 826 (s), 786 (w), 763 (w), 682 (s), 618 (m), 591 (m), 557 (w), 541 (w), 477 (w), 437 (w), 408 (w).

HRMS: EI(+); *m/z* calcd. for $C_{13}H_9F_3O_3$ $[M]^+$: 270.05038, found: 270.04978.

11. (4,5,6-trifluoro-3-(methoxymethoxy)naphthalen-2-yl)methanol (**30**)

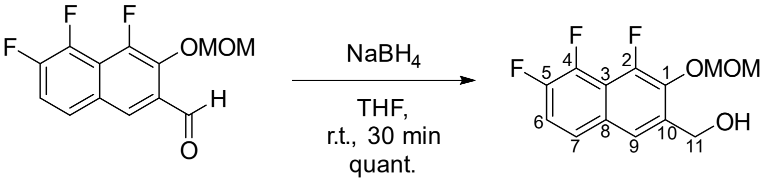

13 [270.21] **30** [272.22]

To a stirred solution of aldehyde **13** (1.73 g, 6.38 mmol, 1.00 eq) in THF (125 mL) was added sodium borohydride (1.45 g, 38.3 mmol, 6.00 eq) and the resulting suspension was stirred for 2 h at room temperature. To the reaction mixture was added water (50 mL) and the aqueous layer was extracted with ethyl acetate (3 × 100 mL). The combined extracts were washed with brine (100 mL), then dried over Na_2SO_4. The crude product was filtered over a pad of silica which was rinsed with EtOAc. The solvent was removed under reduced pressure and the white solid crude product **30** (1.73 g, 6.38 mmol, quant.) was used without further purification.

TLC: R_f = 0.33 (Pe/EtOAc 5:1).

m.p.: 166 °C (CH_2Cl_2).

^{1}H-NMR: 500 MHz, $CDCl_3$; δ (ppm) = 7.60 (s, 1H, *H*9), 7.55 - 7.51 (m, 1H, *H*7), 7.31 - 7.28 (m, 1H, *H*6), 5.32 (d, *J* = 0.9 Hz, 2H, OCH_2), 4.83 (s, 2H, CH_2OH), 3.62 (s, 3H, OCH_3), 2.80 (s, 1H, O*H*).

^{13}C-NMR: 126 MHz, $CDCl_3$; δ (ppm) = 148.4 (ddd, *J* = 254.3, 6.4, 1.8 Hz, 1C, *C*2), 147.8 (ddd, *J* = 247.5, 11.8, 1.4 Hz, 1C, *C*5), 143.6 (ddd, *J* = 257.0, 14.5, 0.9 Hz, 1C, *C*4), 141.0 (d, *J* = 10.0 Hz 1C, *C*1), 135.1 - 135.0 (m, 1C, *C*10), 128.9 - 128.9 (m, 1C, *C*3), 124.4 - 124.3 (m, 1C, *C*7), 122.9 - 122.9 (m, 1C, *C*9), 117.0 (d, *J* = 20.0 Hz, 1C, *C*6), 116.0 - 115.8 (m, 1C, *C*8), 100.0 (d, *J* = 7.3 Hz, 1C, OCH_2), 61.3 (d, *J* = 2.7 Hz, 1C, *C*11), 57.9 (s, 1C, OCH_3).

^{19}F-NMR: 283 MHz, $CDCl_3$; δ (ppm) = −138.6 (dd, *J* = 53.3, 3.9 Hz, 1F, *F*5), −140.5 (dd, *J* = 16.9, 5.2 Hz, 1F, *F*2), −147.0 (dd, *J* = 53.3, 18.2 Hz, 1F, *F*4).

IR: neat, ṽ(cm^{-1}) = 3281 (w), 2945 (w), 2837 (w), 1743 (w), 1648 (w), 1630 (s), 1580 (w), 1505 (w), 1480 (s), 1454 (w), 1439 (m), 1407 (m), 1367 (m), 1354 (w), 1338 (s), 1262 (w), 1248 (s), 1228 (w), 1157 (s), 1107 (w), 1082 (w), 1065 (s), 1039 (s), 986 (w), 922 (s), 873 (m), 817 (s), 796 (w), 767 (w), 727 (w), 706 (w), 689 (s), 626 (w), 605 (m), 588 (s), 566 (w), 544 (w), 510 (w), 473 (w), 458 (w), 424 (w).

HRMS: EI(+); *m/z* calcd. for $C_{13}H_{11}F_3O_3$ $[M]^+$: 272.06603, found: 272.06526.

12. 3-(bromomethyl)-1,7,8-trifluoro-2-(methoxymethoxy)naphthalene (**14**)

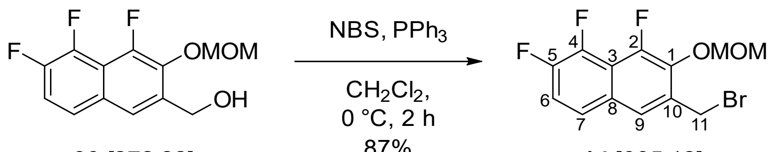

30 [272.22] **14** [335.12]

To a stirred solution of benzyl alcohol **30** (253 mg, 0.93 mmol, 1.00 eq) in dichloromethane (9.3 mL) was added PPh_3 (488 mg, 1.86 mmol, 2.00 eq) and NBS (331 mg, 1.86 mmol, 2.00 eq) sequentially under argon at 0 °C and the resulting solution was stirred for 2 h. To the reaction mixture water (10 mL) was added and the aqueous layer was extracted with ethyl acetate (3 × 10 mL). The combined extracts were washed with brine (10 mL), then dried over Na_2SO_4. The solvent was removed under reduced pressure, the crude product was adsorbed onto silica gel and then purified by column chromatography (pentane/EtOAc 10:1) to give **14** (270 mg, 0.81 mmol) as white solid in 87% yield.

TLC R_f = 0.31 (Pe/EtOAc 10:1).

m.p.: 85 °C (CH_2Cl_2).

^{1}H-NMR: 300 MHz, $CDCl_3$; δ (ppm) = 7.65 (t, *J* = 1.9 Hz, 1H, *H*9), 7.54 - 7.48 (m, 1H, *H7*), 7.33 - 7.24 (m, 1H, *H*6), 5.41 (d, *J* = 1.1 Hz, 2H, OCH_2), 4.69 (s, 2H, $H_2$11), 3.68 (s, 3H, OCH_3).

^{1}H-NMR: 500 MHz, DMSO-d^6; δ (ppm) = 8.02 (s, 1H, *H*9), 7.87 - 7.83 (m, 1H, *H7*), 7.63 (ddd, *J* = 10.5, 9.2, 7.6 Hz, 1H, *H*6), 5.36 (d, *J* = 1.3 Hz, 2H, OCH_2), 4.83 (s, 2H, $H_2$11), 3.57 (s, 3H, OCH_3).

^{13}C-NMR: 126 MHz, DMSO-d^6; δ (ppm) = 147.5 (dd, *J* = 246.0, 11.0 Hz, 1C, *C*5), 147.3 (dd, *J* = 252.5, 5.5 Hz, 1C, *C*2), 142.3 (dd, *J* = 254.0, 15.1 Hz, 1C, *C*4), 139.9 (d, *J* = 11.0 Hz 1C, *C*1), 132.2 - 132.1 (m, 1C, *C*10), 128.0 - 127.9 (m, 1C, *C*8), 126.0 - 125.9 (m, 1C, *C*9), 125.4 - 125.3 (m, 1C, *C7*), 117.1 (d, *J* = 21.1 Hz, 1C, *C*6), 115.2 - 115.0 (m, 1C, *C*3), 99.2 (d, *J* = 8.0 Hz, 1C, O*C*H$_2$), 57.2 (s, 1C, O*C*H$_3$), 28.5 (d, *J* = 2.0 Hz, 1C, *C*11).

^{19}F-NMR: 283 MHz, $CDCl_3$; δ (ppm) = −137.4 (dd, *J* = 54.6, 3.9 Hz, 1F, *F*5), −139.3 (dd, *J* = 16.9, 5.2 Hz, 1F, *F*2), −146.6 (dd, *J* = 54.0, 17.6 Hz, 1F, *F*4).

IR: neat, ṽ(cm^{-1}) = 3281 (w), 2945 (w), 2837 (w), 1743 (w), 1648 (w), 1630 (s), 1580 (w), 1505 (w), 3465 (w), 3280 (w), 3013 (w), 2918 (m), 2852 (w), 2073 (w), 1917 (w), 1743 (w), 1628 (s), 1579 (w), 1504 (w), 1478 (s), 1458 (w), 1440 (w), 1403 (w), 1343 (s), 1261 (s), 1237 (w), 1212 (m), 1154 (s), 1105 (m), 1079 (s), 1038 (s), 997 (m), 954 (m), 918 (s), 896 (w), 879 (w), 836 (w), 812 (s), 763 (w), 722 (w), 690 (w), 656 (w), 623 (w), 605 (w).

HRMS: EI(+); *m/z* calcd. for $C_{13}H_{10}Br_1F_3O_2$ $[M]^+$: 333.98163, found: 333.98203.

13. methyl 1,7,8-trifluoro-3-((4,5,6-trifluoro-3-(methoxymethoxy)naphthalen-2-yl)methyl)-2-naphthoate (**31**)

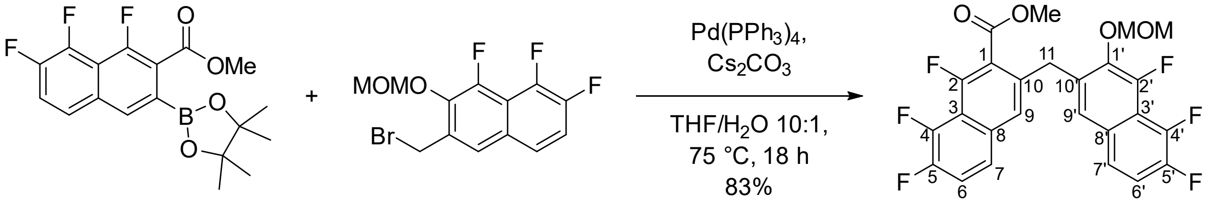

12 [366.14] **14** [335.12] **31** [494.39]

Benzyl bromide **14** (500 mg, 1.49 mmol, 1.00 eq), pinacol boronic ester **12** (84 w%, 683 mg, 1.57 mmol, 1.05 eq), $Pd(PPh_3)_4$ (51.7 mg, 45 µmol, 3.0 mol%) and Cs_2CO_3 (1.46 g, 4.48 mmol, 3.00 eq) were suspended in THF (13.3 mL) and degassed H_2O (1.25 mL) under argon. The resulting mixture was degassed (3×) and then heated to 75 °C for 18 h. The suspension was allowed to cool to r.t. before it was diluted with EtOAc (20 mL) and poured into water (20 mL). The aqueous layer was extracted with EtOAc (3 × 20 mL). The combined extracts were washed with brine (20 mL) and dried over Na_2SO_4. The solvent was removed under reduced pressure and the crude product was adsorbed onto silica gel before it was purified by column chromatography (toluene) to give **31** (610 mg, 1.23 mmol) as yellow solid in 83% yield.

TLC R_f = 0.33 (toluene).

m.p.: 145 °C (EtOAc).

^{1}H-NMR: 500 MHz, $CDCl_3$; δ (ppm) = 7.50 - 7.47 (m, 1H, *H*7), 7.43 - 7.38 (m, 2H, *H*6 & *H*7'), 7.35 (s, 1H, *H*9), 7.27 - 7.21 (m, 2H, *H*6' & *H*9'), 5.27 (d, *J* = 1.2 Hz, 2H, OC*H*2), 4.38 (s, 2H, $H_2$11), 3.83 (s, 3H, CO_2CH_3), 3.53 (s, 3H, OC*H*3).

^{13}C-NMR: 126 MHz, $CDCl_3$; δ (ppm) = 165.6 (s, 1C, *C*O2Me), 155.3 (ddd, *J* = 262.9, 6.7, 2.0 Hz, 1C, *C*2), 148.3 (ddd, *J* = 254.2, 6.4, 1.9 Hz, 1C, *C*4’), 147.7 (ddd, *J* = 248.5, 11.6, 1.3 Hz, 1C, *C*5 or *C*5’), 147.6 (ddd, *J* = 246.8, 11.7, 1.4 Hz, 1C, *C*5 or *C*5’), 144.5 (ddd, *J* = 259.9, 14.4, 1.5 Hz, 1C, *C*2’), 143.4 (ddd, *J* = 256.5, 14.8, 1.0 Hz, 1C, *C*4), 140.9 (dd, *J* = 9.8, 1.2 Hz, 1C, C1'), 135.5 - 135.4 (m, 1C, *C*10 or *C*10'), 133.4 - 133.4 (m, 1C, *C*10 or *C*10'), 132.8 - 132.7 (m, 1C, *C*8), 128.5 - 128.4 (m, 1C, *C*8'), 124.3 - 124.2 (m, 2C, *C*9 & *C*9'), 123.9 - 123.7 (m, 2C, *C*7 & *C*7'), 119.6 (dd, *J* = 21.0, 1.4 Hz, 1C, *C*6), 119.2 - 119.1 (m, 1C, *C*1), 116.6 (dd, *J* = 21.1, 1.3 Hz, 1C, *C*6'), 115.5-115.3 (m, 1C, *C*3'), 113.7 (ddd, *J* = 12.9, 8.6, 1.5 Hz, 1C, *C*3), 99.4 (d, *J* = 8.8 Hz, 1C, O*C*H2), 57.7 (s, 1C, O*C*H3), 52.8 (s, 1C, CO_2CH_3), 33.9 (t, *J* = 2.1 Hz, 1C, *C*11).

^{19}F-NMR: 283 MHz, $CDCl_3$; δ (ppm) = −117.1 (dd, *J* = 58.5, 5.2 Hz, 1F), −138.1 (dd, *J* = 54.0, 4.5 Hz, 1F), −139.6 (dd, *J* = 17.6, 4.5 Hz, 1F), −140.8 (dd, *J* = 16.9, 3.9 Hz, 1F), −144.2 (dd, *J* = 57.9, 17.6 Hz, 1F), −147.0 (dd, *J* = 54.0, 17.6 Hz, 1F).

IR: neat, ṽ(cm^{-1}) = 2960 (w), 2921 (w), 2850 (w), 1730 (m), 1648 (w), 1622 (m), 1579 (w), 1502 (w), 1477 (w), 1451 (m), 1403 (w), 1354 (s), 1260 (s), 1203 (w), 1156 (w), 1130 (w), 1102 (w), 1059 (m), 1040 (w), 938 (s), 890 (w), 875 (w), 858 (w), 798 (s), 767 (w), 734 (w), 705 (w), 680 (w), 602 (m), 459 (w).

HRMS: EI(+); *m/z* calcd. for $C_{25}H_{16}F_6O_4$ $[M]^+$: 494.09528, found: 494.09469.

14. methyl 1,7,8-trifluoro-3-((4,5,6-trifluoro-3-hydroxynaphthalen-2-yl)methyl)-2-naphthoate (**32**)

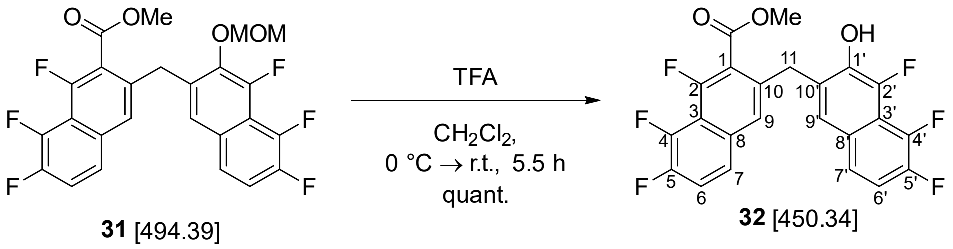

31 [494.39] → **32** [450.34]

To a stirred solution of MOM-ether **31** (92.0 mg, 0.19 mmol, 1.00 eq) in CH_2Cl_2 (0.96 mL) was added dropwise TFA (0.07 mL, 0.93 mmol, 5.00 eq) at 0 °C under argon and the resulting suspension was allowed to warm to r.t. over 5.5 h. The reaction was quenched with water (5 mL) and extracted with EtOAc (3 × 5 mL). The combined organic layers were washed with sat. aq. $NaHCO_3$-solution (5 mL) and brine (5 mL) and dried over Na_2SO_4. The crude product was filtered over a plug of silica which was rinsed with toluene. The solvent was removed under reduced pressure to give **32** (84.0 mg, 0.19 mmol, quant.) as light yellow solid, which could be used without further purification.

TLC R_f = 0.15 (toluene).

m.p.: 132 °C (EtOAc).

^{1}H-NMR: 500 MHz, DMSO-d^6; δ (ppm) = 10.52 (s, 1H, O*H*), 7.88 - 7.85 (m, 1H, *H*7), 7.76 (td, *J* = 9.5, 7.8 Hz, 1H, *H*6), 7.70 (s, 1H, *H*9), 7.68 - 7.65 (m, 1H, *H*7'), 7.43 (s, 1H, *H*9'), 7.39 (td, *J* = 9.6, 7.8 Hz, 1H, *H*6'), 4.29 (s, 2H, $H_2$11), 3.77 (s, 3H, CO_2CH_3).

^{13}C-NMR: 126 MHz, DMSO-d^6; δ (ppm) = 164.8 (s, 1C, CO_2Me), 153.7 (ddd, *J* = 258.7, 6.6. 1.1 Hz, 1C, *C*2), 146.9 (ddd, *J* = 243.0, 11.2. 1.0 Hz, 1C, *C*4 or *C*5), 147.1 (dd, *J* = 245.4, 11.3 Hz, 1C, *C*4' or *C*5'), 143.3 (dd, *J* = 255.4, 15.0 Hz, 1C, *C*4), 142. 7 (dd, *J* = 242.7, 6.2 Hz, 1C, *C*2'), 141.6 (dd, *J* = 251.3, 14.6 Hz, 1C, *C*4' or *C*5'), 141.4 (d, *J* = 13.0 Hz, 1C, *C*10'), 135.4 - 135.4 (m, 1C, *C*1'), 132.6 (d, *J* = 3.7 Hz, 1C, *C*8), 130.7 - 130.6 (m, 1C, *C*1), 125.6 - 125.5 (m, 1C, *C*3' or *C*8'), 125.2 - 125.1 (m, 1C, *C*7),

124.9 - 124.7 (m, 2C, *C*7' & *C*9), 124.5 - 124.4 (m, 1C, *C*9'), 119.8 (d, *J* = 20.8 Hz, 1C, *C*6), 119.2 (d, *J* = 15.0 Hz, 1C, *C*10), 114.4 (d, *J* = 20.9 Hz, 1C, *C*6'), 114.1 - 114.0 (m, 1C, *C*3' or *C*8'), 112.4 - 112.2 (m, 1C, *C*3), 52.9 (s, 1C, CO_2CH_3), 33.1 (s, 1C, *C*11).

^{19}F-NMR: 283 MHz, DMSO-d^6; δ (ppm) = −120.1 (dd, *J* = 55.3, 3.2 Hz, 1F), −140.2 (dd, *J* = 18.9, 3.2 Hz, 1F), −142.1 (dd, *J* = 18.9, 3.2 Hz, 1F), −146.4 (dd, *J* = 54.9, 18.7 Hz, 1F), −147.9 (d, *J* = 46.9 Hz, 1F), −150.6 (dd, *J* = 46.6, 18.6 Hz, 1F).

IR: neat, ṽ(cm^{-1}) = 3082 (w), 2953 (w), 2920 (m), 2851 (w), 1719 (s), 1621 (s), 1579 (w), 1486 (w), 1465 (m), 1447 (w), 1350 (s), 1278 (w), 1258 (s), 1222 (w), 1173 (w), 1131 (m), 1101 (w), 1060 (w), 1041 (m), 1024 (w), 1001 (s), 967 (m), 946 (w), 892 (w), 877 (m), 839 (m), 803 (m), 761 (w), 723 (w), 709 (w), 670 (w), 649 (w), 617 (w), 592 (m), 539 (w), 512 (w), 475 (w), 459 (m), 426 (w).

HRMS: EI(+); *m/z* calcd. for $C_{23}H_{12}F_6O_3$ $[M]^+$: 450.06906, found: 450.06944.

15. methyl 1,7,8-trifluoro-3-((4,5,6-trifluoro-3-(((trifluoromethyl)sulfonyl)oxy)naphthalen-2-yl)methyl)-2-naphthoate (**15**)

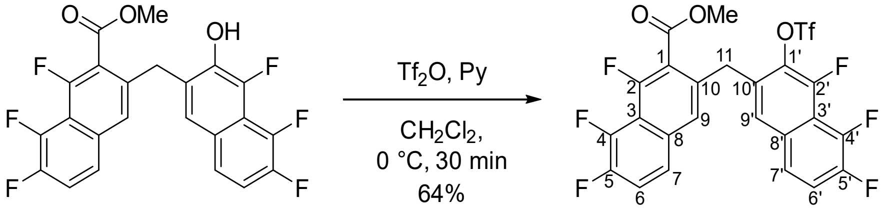

32 [450.34] **15** [582.39]

To a stirred solution of alcohol **32** (572 mg, 1.27 mmol, 1.00 eq) and pyridine (281 µL, 3.49 mmol, 2.75 eq) in CH_2Cl_2 (20.0 mL) was added dropwise Tf_2O (256 µL, 1.52 mmol, 1.20 eq) at 0 °C under argon. The mixture was stirred for 30 min at 0 °C before it was diluted with Et_2O (10 mL) and poured into sat. aq. $NaHCO_3$-solution (20 mL). The aqueous layer was extracted with Et_2O (3 × 20 mL) and the combined extracts were washed with brine (20 mL). After drying over Na_2SO_4, the solvent was removed under reduced pressure and the residue was adsorbed onto silica gel. Purification by column chromatography (pentane/EtOAc 10:1 to 7:1) yielded triflate **15** (475 mg, 816 µmol) as white solid in 64%.

TLC R_f = 0.42 (Pe/EtOAc 5:1).

m.p.: 127 °C (EtOAc).

^{1}H-NMR: 500 MHz, $CDCl_3$; δ (ppm) = 7.58 - 7.54 (m, 1H, *H*7), 7.49 - 7.44 (m, 3H, *H*6, *H*7' & *H*9), 7.40 (td, *J* = 9.4, 7.0 Hz, 1H, *H*6'), 7.23 (s, 1H, *H*9'), 4.46 (s, 2H, $H_2$11), 3.77 (s, 3H, CO_2CH_3).

^{13}C-NMR: 126 MHz, $CDCl_3$; δ (ppm) = 165.3 (s, 1C, CO_2Me), 156.0 (ddd, *J* = 265.4, 6.5, 0.5 Hz, 1C, *C*2), 148.9 (ddd, *J* = 263.5, 6.1, 0.5 Hz, 1C, *C*2'), 148.1 (dd, *J* = 249.8, 11.7 Hz, 2C, *C*5 & *C*5'), 144.7 (ddd, *J* = 260.0, 15.1, 2.0 Hz, 1C, *C*4), 144.1 (ddd, *J* = 260.0, 15.1, 2.0 Hz, 1C, *C*4'), 133.6 (s, 1C, *C*1'), 133.5 - 133.4 (m, 1C, *C*10'), 132.9 (d, *J* = 3.9 Hz, 1C, *C*8), 132.4 - 132.3 (m, 1C, *C*10), 130.8 - 130.7 (m, 1C, *C*8'), 125.3 - 125.3 (m, 1C, *C*9), 125.0 - 124.9 (m, 1C, *C*9'), 124.5 - 124.3 (m, 1C, *C*7'), 124.2 - 124.1 (m, 1C, *C*7), 120.1 (d, *J* = 21.2 Hz, 1C, *C*6), 119.7 (d, *J* = 21.1 Hz, 1C, *C*6'), 118.8 (d, *J* = 14.2 Hz, 1C, *C*1), 118.7 (q, *J* = 320.9 Hz, 1C, CF_3), 114.8 - 114.7 (m, 1C, *C*3'), 114.4 - 114.3 (m, 1C, *C*3), 53.0 (s, 1C, CO_2CH_3), 34.2 (t, *J* = 2.0 Hz, 1C, *C*11).

^{19}F-NMR: 283 MHz, $CDCl_3$; δ (ppm) = −73.4 (d, *J* = 14.0 Hz, 3F, CF_3), −115.4 (dd, *J* = 59.1, 4.5 Hz, 1F), −130.5 (dqd, *J* = 50.4, 14.2, 5.5 Hz, 1F), −138.2 (dd, *J* = 17.0, 5.1 Hz, 1F), −138.7 (dd, *J* = 17.1, 4.1 Hz, 1F), −143.7 (dd, *J* = 59.2, 17.3 Hz, 1F), −144.7 (dd, *J* = 51.8, 17.2 Hz, 1F).

IR: neat, ṽ(cm^{-1}) = 2918 (w), 2850 (w), 1734 (m), 1712 (w), 1638 (w), 1620 (m), 1581 (w), 1501 (w), 1486 (w), 1446 (w), 1411 (m), 1352 (s), 1265 (m), 1216 (s), 1181 (w), 1130 (s), 1080 (m), 1060 (w), 1044 (w), 1014 (w), 972 (w), 947 (w), 903 (w), 879 (s), 811 (m), 783 (w), 767 (w), 751 (m), 714 (w), 696 (w), 661 (w), 644 (w), 619 (s), 597 (w), 539 (w), 519 (w), 495 (m), 461 (w).

HRMS: EI(+); *m/z* calcd. for $C_{24}H_{11}F_9O_5S_1$ $[M]^+$: 582.01835, found: 582.01872.

16. 1,7,8-trifluoro-3-((4,5,6-trifluoro-3-(hydroxymethyl)naphthalen-2-yl)methyl)naphthalen-2-yl trifluoromethanesulfonate (**33**)

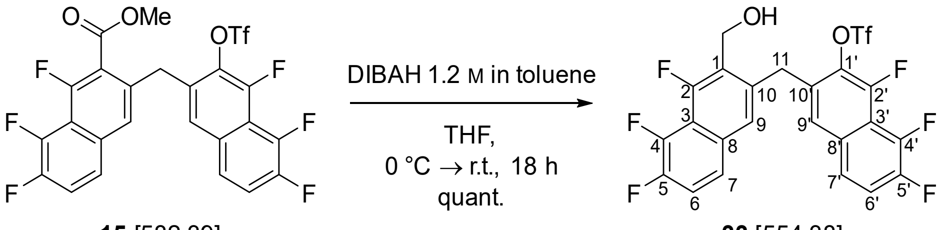

15 [582.39] **33** [554.38]

To a stirred solution of ester **15** (438 mg, 752 µmol, 1.00 eq) in THF (3.50 mL) DIBAH (1.2 M in toluene, 1.57 mL, 1.88 mmol, 2.50 eq) was added dropwise at 0 °C under argon and the resulting solution was warmed to r.t. Since the conversion was not complete after 8 h additional DIBAH (0.62 mL, 0.75 mmol, 1.00 eq) was added dropwise. After 22 h, 2 M HCl (5 mL) was added and the aqueous layer was extracted with EtOAc (3 × 10 mL). The combined organic extracts were washed with brine (5 mL), dried over Na_2SO_4 and the solvent was removed under reduced pressure. The crude product was adsorbed onto silica gel and then purified via column

chromatography (pentane/EtOAc 5:1 to 3:1) to give **33** (415 mg, 749 µmol) as colorless oil in quant. yield.

TLC R_f = 0.18 (toluene).

^{1}H-NMR: 500 MHz, DMSO-*d*^{6}; δ (ppm) = 7.90 - 7.87 (m, 1H, *H*7'), 7.82 - 7.75 (m, 2H, *H*6' & *H*7), 7.66 (td, *J* = 9.4, 7.9 Hz, 1H, *H*6), 7.62 (s, 1H, *H*9'), 7.53 (s, 1H, *H*9), 5.32 (t, *J* = 5.4 Hz, 1H, O*H*), 4.64 (dd, *J* = 5.3, 2.6 Hz, 2H, CH_2OH), 4.56 (s, 2H, $H_2$11).

^{13}C-NMR: 126 MHz, DMSO-*d*^{6}; δ (ppm) = 154.0 (dd, *J* = 252.8, 6.1 Hz, 1C, *C*2), 147.8 (dd, *J* = 260.2, 6.2 Hz, 1C, *C*4’), 147.4 (dd, *J* = 247.9, 11.2 Hz, 1C, *C*5’), 146.9 (dd, *J* = 244.6, 11.7 Hz, 1C, *C*5), 143.0 (dd, *J* = 254.6, 14.9 Hz, 1C, *C*4), 142.8 (dd, *J* = 256.3, 15.4 Hz, 1C, *C*2’), 136.7 - 136.6 (m, 1C, *C*10), 133.2 (d, *J* = 12.0 Hz, 1C, *C*1'), 132.2 - 132.1 (m, 1C, *C*10'), 131.8 - 131.7 (m, 1C, *C*8), 130.9 - 130.8 (m, 1C, *C*8'), 125.9 - 125.8 (m, 1C, *C*9'), 125.8 - 125.6 (m, 1C, *C*7'), 125.0 - 124.9 (m, 1C, *C*7), 124.8 (d, *J* = 13.1 Hz, 1C, *C*1), 124.5 - 124.4 (m, 1C, *C*9), 119.8 (d, *J* = 20.8 Hz, 1C, *C*6'), 118.2 (d, *J* = 20.9 Hz, 1C, *C*6), 118.1 (q, *J* = 320.7 Hz, 1C, *C*F_3), 113.4 - 113.2 (m, 1C, *C*3'), 113.0 (dd, *J* = 14.3, 7.9 Hz, 1C, *C*3), 53.2 (d, *J* = 7.5 Hz, 1C, *C*H_2OH), 32.1 (t, *J* = 2.5 Hz, 1C, *C*11).

^{19}F-NMR: 283 MHz, DMSO-*d*^{6}; δ (ppm) = −73.5 (d, *J* = 12.8 Hz, 3F, C*F*$_3$), −124.0 (d, *J* = 56.3 Hz, 1F), −132.8 (dqd, *J* = 49.7, 12.7, 4.7 Hz, 1F), −139.1 (dd, *J* = 18.7, 4.6 Hz, 1F), −141.1 (dd, *J* = 18.6, 3.5 Hz, 1F), −147.2 (dd, *J* = 59.7, 18.9 Hz, 1F), −147.4 (dd, *J* = 56.6, 18.7 Hz, 1F).

IR: neat, ṽ(cm^{-1}) = 3409 (w), 2924 (w), 2855 (w), 1713 (w), 1626 (m), 1581 (w), 1504 (w), 1482 (m), 1419 (m), 1354 (s), 1264 (m), 1211 (s), 1131 (m), 1081 (s), 1045 (m), 1006 (w), 924 (w), 867 (s), 804 (w), 784 (m), 752 (m), 712 (w), 617 (s), 599 (w), 528 (w), 495 (m), 447 (w).

HRMS: EI(+); *m/z* calcd. for $C_{23}H_{11}F_9O_4S_1$ $[M]^+$: 554.02343, found: 554.02395.

17. 1,7,8-trifluoro-3-((4,5,6-trifluoro-3-formylnaphthalen-2-yl)methyl)naphthalen-2-yl trifluoromethanesulfonate (**16**)

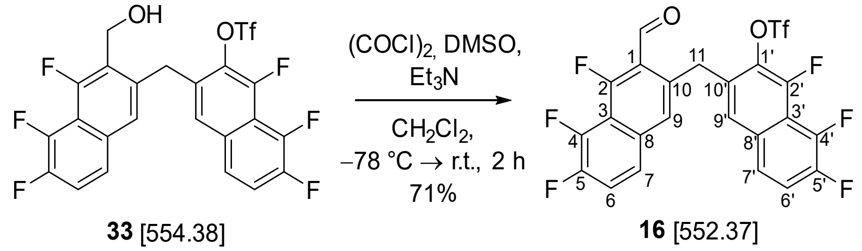

To a stirred solution of DMSO (0.22 mL, 3.15 mmol, 3.00 eq) in CH_2Cl_2 (19.0 mL) was added oxalyl chloride (0.14 mL, 1.58 mmol, 1.50 eq) at –78 °C under argon. After 10 min the reaction mixture

was added to a solution of alcohol **33** (583 mg, 1.05 mmol, 1.00 eq) in CH_2Cl_2 (19.0 mL) at –78 °C and stirred for 30 min. Then Et_3N (0.73 mL, 5.25 mmol, 5.00 eq) was added dropwise and the reaction mixture was stirred additional 30 min at that temperature before it was allowed to warm to r.t. After 1 h H_2O (100 mL) was added and the aqueous layer was extracted with EtOAc (3 × 50 mL). The combined organic extracts were washed with brine (50 mL), dried over Na_2SO_4 and the solvent was removed under reduced pressure. The crude product was adsorbed onto silica gel and then purified via column chromatography (pentane/EtOAc 8:1 to 5:1) to give **16** (422 mg, 746 µmol) as white solid in 71% yield.

TLC R_f = 0.31 (pentane/EtOAc 5:1).

m.p.: 194 °C (EtOAc).

^{1}H-NMR: 500 MHz, $CDCl_3$; δ (ppm) = 10.63 (s, 1H, C*H*O), 7.60 - 7.54 (m, 2H, *H*6 &*H*7'), 7.43 (s, 1H, *H*9), 7.41 - 7.34 (m, 2H, *H*6' & *H*7), 7.06 (s, 1H, *H*9'), 4.69 (s, 2H, $H_2$11).

^{13}C-NMR: 126 MHz, $CDCl_3$; δ (ppm) = 187.9 (d, *J* = 15.1 Hz, 1C, *C*HO), 164.2 (ddd, *J* = 272.8, 6.8, 1.8 Hz, 1C, *C*2), 148.9 (ddd, *J* = 264.0, 7.0, 2.0 Hz, 1C, *C*2'), 148.2 (dd, *J* = 249.5, 11.5 Hz, 1C, *C*5 or *C*5'), 147.9 (ddd, *J* = 248.5, 11.5, 0.5 Hz, 1C, *C*5 or *C*5'), 144.9 (dd, *J* = 262.5, 14.6 Hz, 1C, *C*4), 144.2 (ddd, *J* = 260.0, 15.1, 2.0 Hz, 1C, *C*4'), 134.6 - 134.5 (m, 1C, *C*10), 134.4 (d, *J* = 6.0 Hz, 1C, *C*1), 133.9 (d, *J* = 13.0 Hz, 1C, *C*1'), 133.4 - 133.3 (m, 1C, *C*10'), 130.8 - 130.7 (m, 1C, *C*8'), 126.6 - 126.5 (m, 1C, *C*9), 124.4 - 124.1 (m, 2C, *C*7 & *C*7'), 123.8 - 123.7 (m, 1C, *C*9'), 121.8 (d, *J* = 21.1 Hz, 1C, *C*6), 119.5 (d, *J* = 21.1 Hz, 1C, *C*6'), 119.3 - 119.2 (m, 1C, *C*8), 118.8 (q, *J* = 320.6 Hz, 1C, CF_3), 114.8 - 114.6 (m, 1C, *C*3'), 114.5 - 114.3 (m, 1C, *C*3), 34.3 (t, *J* = 2.0 Hz, 1C, *C*11).

^{19}F-NMR: 283 MHz, $CDCl_3$; δ (ppm) = –72.7 (d, *J* = 14.3 Hz, 3F, CF_3), –121.5 (dd, *J* = 62.4, 4.5 Hz, 1F), –129.9 (dqd, *J* = 51.7, 14.0, 5.0 Hz, 1F), –137.3 (dd, *J* = 17.5, 4.5 Hz, 1F), –138.0 (dd, *J* = 16.9, 5.2 Hz, 1F), –140.9 (dd, *J* = 62.7, 17.2 Hz, 1F), –144.1 (dd, *J* = 52.0, 17.6 Hz, 1F).

IR: neat, ṽ(cm^{-1}) = 2923 (w), 2853 (w), 1690 (s), 1639 (m), 1616 (w), 1582 (w), 1503 (w), 1486 (w), 1475 (m), 1446 (w), 1408 (m), 1358 (s), 1311 (w), 1268 (m), 1233 (w), 1217 (s), 1179 (w), 1157 (w), 1129 (m), 1082 (m), 1045 (m), 1007 (w), 976 (w), 961 (w), 897 (s), 884 (w), 816 (m), 784 (m), 748 (s), 694 (w), 665 (w), 646 (w), 614 (s), 600 (w), 574 (w), 532 (w), 505 (m), 451 (w), 437 (w).

HRMS: EI(+); *m/z* calcd. for $C_{23}H_9F_9O_4S_1$ $[M]^+$: 552.00778, found: 552.00840.

18. 3,4,5,7,8,9-hexafluoro-6,13-dihydropentacen-6-ol (**17**)

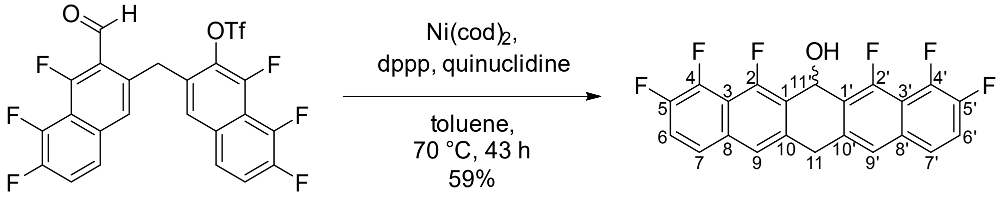

16 [552.37] **17** [404.31]

Triflate **16** (30.0 mg, 54 µmol, 1.00 eq) and dppp (26.9 mg, 65 µmol, 1.20 eq) were evacuated and backfilled with argon three times. In a glovebox Ni(cod)$_2$ (14.9 mg, 54 µmol, 1.00 eq) and toluene (0.55 mL) were added sequentially. Outside the glovebox quinuclidine (6.3 mg, 54 µmol, 1.00 eq) was added under argon and the resulting golden reaction mixture was stirred at r.t. for 1 h. It was then heated to 70 °C for 43 h. The reaction mixture was allowed to cool to r.t. before it was filtered over a plug of celite which was rinsed with EtOAc. The solvent was removed under reduced pressure and the crude product was adsorbed onto silica gel before it was purified by column chromatography (pentane/EtOAc 3:1 to 1:1) to give **17** (13.0 mg, 32 µmol) as pale orange solid in 59% yield.

TLC R_f = 0.57 (pentane/EtOAc 1:1).

m.p.: 197 °C decomposition (EtOAc).

^{1}H-NMR: 500 MHz, acetone-*d*^{6}; δ (ppm) = 7.82 - 7.78 (m, 4H, *H*7, *H*7', *H*9 & *H*9’), 7.58 (td, *J* = 9.7, 7.5 Hz, 2H, *H*6 & *H*6’), 6.84 (s, 1H, *H*11’), 4.58 (d, *J* = 17.4 Hz, 1H, H_a11), 4.27 (d, *J* = 17.5 Hz, 1H, H_b11), 3.79 (s, 1H, O*H*).

^{13}C-NMR: 126 MHz, acetone-*d*^{6}; δ (ppm) = 154.8 (ddd, *J* = 256.0, 7.0, 2.0 Hz, 2C, *C*2 & C2’), 148.0 (ddd, *J* = 244.4, 12.0, 1.5 Hz, 2C, *C*4 & *C*4’), 144.7 (ddd, *J* = 255.7, 13.8, 1.3 Hz, 2C, *C*5 & *C*5’), 138.0 - 137.9 (m, 2C, *C*1 & *C*1’), 133.3 - 133.2 (m, 2C, *C*8 & *C*8’), 125.4 - 125.2 (m, 2C, *C*7 & *C*7'), 124.0 (d, *J* = 14.0 Hz, 2C, *C*10 & *C*10’), 122.6 - 122.5 (m, 2C, *C*9 & *C*9’), 119.1 (dd, *J* = 21.6, 1.5 Hz, 2C, *C*6 & *C*6’), 114.1 (dd, *J* = 14.1, 9.0 Hz, 2C, *C*3 & *C*3'), 56.7 (t, *J* = 7.0 Hz, 1C, *C*11’), 35.1 - 35.0 (m, 1C, *C*11).

^{19}F-NMR: 283 MHz, acetone-*d*^{6}; δ (ppm) = −125.7 (dd, *J* = 56.6, 4.5 Hz, 2F, *F*5 & *F*5’), −141.4 (dd, *J* = 16.9, 3.9 Hz, 2F, *F*2 & *F*2’), −146.1 (dd, *J* = 55.9, 16.9 Hz, 2F, *F*4 & *F*4’).

IR: neat, ṽ(cm^{-1}) = 3582 (w), 3415 (w), 2936 (w), 1630 (s), 1582 (w), 1503 (w), 1480 (m), 1419 (w), 1349 (s), 1259 (m), 1173 (w), 1082 (m), 1038 (m), 1010 (w), 982 (w), 960 (w), 864 (m), 806 (m), 774 (w), 756 (w), 706 (w), 664 (w), 645 (w), 601 (m), 557 (w), 528 (w), 500 (w), 424 (w).

HRMS: EI(+); *m/z* calcd. for $C_{22}H_{10}F_6O_1$ $[M]^+$: 404.06358, found: 404.06522.

19. 1,2,10,11,12,14-hexafluoropentacene (F_6PEN, **1**)

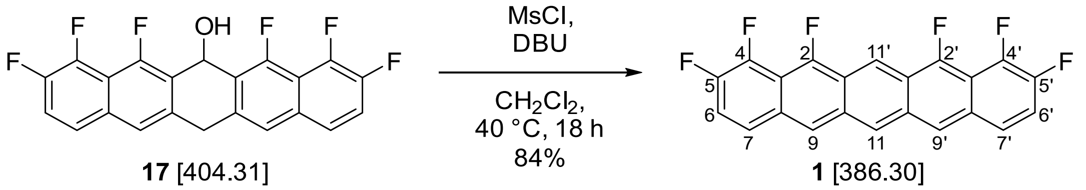

17 [404.31] **1** [386.30]

Alcohol **17** (22 mg, 55.4 µmol, 1.00 eq) was suspended in CH_2Cl_2 (1.4 mL) and DBU (41.3 µL, 277 µmol, 5.00 eq) and MsCl (12.8 µL, 166 µmol, 3.00 eq) were added sequentially at 0 °C under argon. The reaction mixture was heated to 40 °C and stirred for 22 h under exclusion of light during which a blue solid deposed. The solid was centrifuged and washed with H_2O (2 × 1.0 mL) and acetone (2 × 1.0 mL) to give F_6PEN **1** (18 mg, 46.6 µmol, 84%) as dark blue solid.

m.p.: 197 °C decomposition (EtOAc).

^{1}H-NMR: 300 MHz, 368 *K*, naphthalene-*d*^{8}; δ (ppm) = 9.42 (s, 1H, *H*11’), 7.91 (s, 1H, *H*11), 7.50 (s, 2H, *H*9 & *H*9’), 6.85 - 6.80 (m, 2H, *H7* & *H7*’), 6.72 - 6.63 (m, 2H, *H*6 & *H*6’).

^{1}H{^{19}F}-NMR: 300 MHz, 368 *K*, naphthalene-*d*^{8}; δ (ppm) = 9.44 - 9.41 (m, 1H, *H*11’), 7.90 (s, 1H, *H*11), 7.50 (s, 2H, *H*9 & *H*9’), 6.82 (d, *J* = 9.7 Hz, 2H, *H7* & *H7*’), 6.67 (d, *J* = 9.5 Hz, 2H, *H*6 & *H*6’).

^{13}C-NMR: due to very low solubility no ^{13}C-NMR could be obtained.

^{19}F-NMR: 283 MHz, 368 *K*, naphthalene-*d*^{8}; δ (ppm) = −121.5 - −121.7 (m, 2F, *F*5 & *F*5’), −139.7 (dd, *J* = 15.2, 11.8 Hz, 2F, *F*2 & *F*2’), −145.5 (dd, *J* = 49.6, 11.8 Hz, 2F, *F*4 & *F*4’).

IR: neat, ṽ(cm^{-1}) = 2962 (w), 1655 (s), 1621 (w), 1571 (w), 1549 (w), 1522 (w), 1468 (w), 1445 (m), 1407 (w), 1360 (s), 1298 (w), 1253 (s), 1175 (w), 1088 (w), 1045 (w), 1026 (s), 1005 (w), 893 (m), 874 (m), 856 (w), 797 (w), 779 (s), 694 (w), 663 (m), 582 (m), 511 (w), 490 (w).

HRMS: APCI(-); *m/z* calcd. for $C_{22}H_8F_6$ [M]$^-$: 386.0536, found: 386.0553.

UV-VIS: CH_2Cl_2, λ_{max} (nm) = 430, 500, 513, 540, 560, 587.

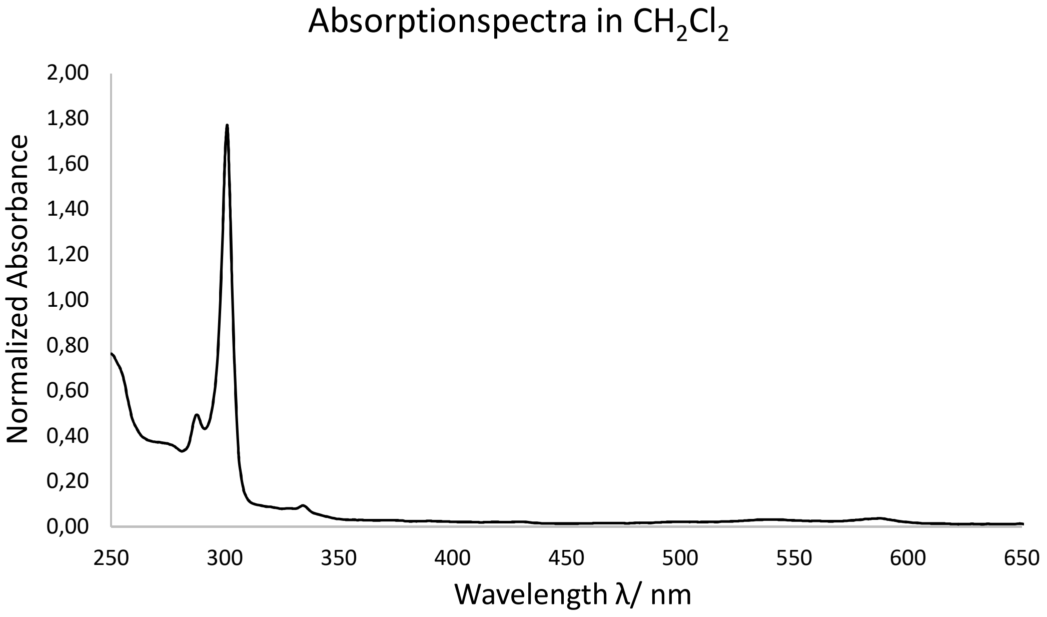
Absorptionspectra in CH_2Cl_2
Normalized Absorbance
2,00
1,80
1,60
1,40
1,20
1,00
0,80
0,60
0,40
0,20
0,00
250
300
350
400
450
500
550
600
650
Wavelength λ/ nm

5. Synthesis 1,2,9,10,11-Pentafluorotetracene 2

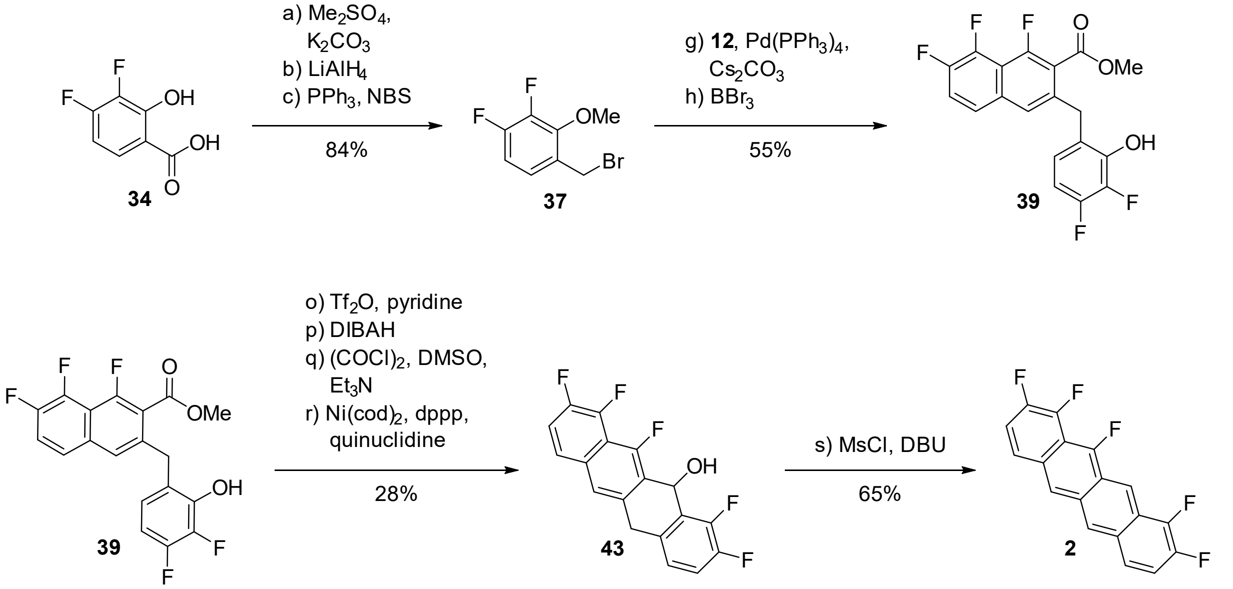

Scheme S1. Synthesis of unsymmetrical partially fluorinated pentafluorotetracene **2** using the regioselective stepwise coupling approach to build up one aromatic ring.

1. methyl 3,4-difluoro-2-methoxybenzoate (35)

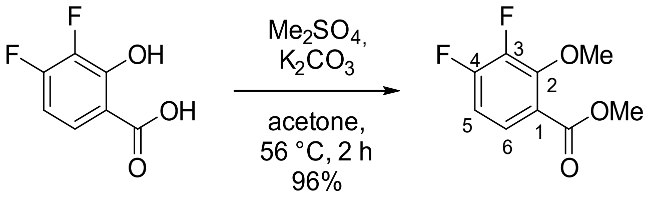

34 [174.10] **35** [202.16]

Salicylic acid **34** (1.00 g, 5.74 mmol, 1.00 eq.) and K_2CO_3 (2.38 g, 17.2 mmol, 3.00 eq.) were suspended in acetone (35 mL) and dimethylsulfate (1.1 mL, 11.5 mmol, 2.00 eq.) was added . The suspension was stirred for 2 h at 56 °C and after cooling to rt was filtered through a pad of Celite. The filter cake was rinsed with acetone (50 mL) and brine (50 mL) was added. The mixture was extracted with EtOAc (3 × 30 mL) and the combined organic layers were dried over $MgSO_4$. After removal of the solvent, ester **35** (1.11 g, 5.50 mmol, 96%) was obtained as yellowish oil, which was used in the next step without further purification.

TLC: R_f = 0.71 (Pe/EtOAc 5:1).

^{1}H-NMR: 500 MHz, $CDCl_3$; δ (ppm) = 7.58 (ddd, *J* = 8.9, 6.0, 2.4 Hz, 1H, *H*6), 6.93 (dt, *J* = 9.1, 7.0 Hz, 1H, *H*5), 4.02 (d, *J* = 1.6 Hz, 3H, OMe), 3.91 (s, 3H, CO_2Me).

^{13}C-NMR: 126 MHz, $CDCl_3$; δ (ppm) = 165.2 (d, *J* = 3.8 Hz, 1C, *C*O_2Me), 154.0 (dd, *J* = 255.9, 11.3 Hz, 1C, *C*3), 150.1 (dd, *J* = 8.9, 2.6 Hz, 1C, *C*2), 145.2 (dd, *J* = 250.3, 13.1 Hz, 1C, *C*4), 126.2 (dd, *J* = 9.3, 5.0 Hz, 1C, *C*6), 121.8 (d, *J* = 3.0 Hz, 1C, *C*1), 111.6 (d, *J* = 18.0 Hz, 1C, *C*5), 62.6 (d, *J* = 5.0 Hz, 1C, OMe), 52.6 (s, 1C, CO_2*Me*).

^{19}F-NMR: 283 MHz, $CDCl_3$; δ (ppm) = –129.1 (d, J = 19.6 Hz, 1F, *F*3), –153.4 (d, J = 19.5 Hz, 1F, *F*4).

IR: film, ṽ(cm^{-1}) = 2960 (w), 1730 (m), 1618 (w), 1503 (w), 1474 (m), 1426 (m), 1304 (w), 1267 (m), 1241 (w), 1202 (m), 1121 (w), 1096 (w), 1061 (s), 1006 (w), 953 (m), 915 (w), 891 (w), 798 (m), 784 (w), 736 (w), 690 (w), 642 (w).

HRMS: ESI(+); *m/z* calcd. for $C_9H_8F_2O_3Na$ [M+Na]$^+$: 225.0334 , found: 225.0340.

2. (3,4-difluoro-2-methoxyphenyl)methanol (**36**)

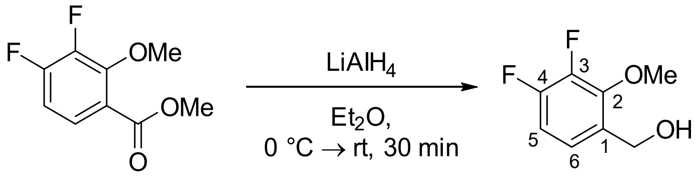

35 [202.16] **36** [174.15]

$LiAlH_4$ (176 mg, 4.63 mmol, 1.10 eq.) was added to a solution of ester **35** (851 mg, 4.21 mmol, 1.00 eq.) in Et_2O (20 mL) at 0 °C under argon. The suspension was allowed to warm up to rt and stirred for 30 min. The suspension was cooled to 0 °C and carefully acidified to pH = 1 using 2 M HCl. The mixture was extracted with Et_2O (4 × 50 mL) and the combined organic layers were washed with brine (30 mL) and dried over $MgSO_4$. The solvent was carefully removed at 100 mbar and the alcohol **36** was used in the next step without further purification.

TLC: R_f = 0.25 (Pe/EtOAc 3:1).

^{1}H-NMR: 500 MHz, $CDCl_3$; δ (ppm) = 7.04-7.01 (m, 1H, *H*6), 6.87-6.82 (m, 1H, *H*5), 4.64 (s, 2H, C*H*$_2$OH), 4.03 (d, J = 2.6 Hz, 3H, OMe).

^{13}C-NMR: 126 MHz, $CDCl_3$; δ (ppm) = 151.4 (dd, J = 248.4, 11.5 Hz, 1C, *C*3), 146.8 (d, J = 6.0 Hz, 1C, *C*2), 143.9 (dd, J = 249.2, 13.8 Hz, 1C, *C*4), 129.9 (d, J = 4.2 Hz, 1C, *C*1), 122.9 (dd, J = 9.4, 4.0 Hz, 1C, *C*6), 110.9 (d, J = 17.1 Hz, 1C, *C*5), 61.7 (d, J = 7.0 Hz, OMe), 61.0 (d, J = 2.3 Hz, 1C, *C*H$_2$OH).

^{19}F-NMR: 283 MHz, $CDCl_3$; δ (ppm) = –136.4 (d, J = 19.0 Hz, 1F, *F*3), –154.9 (d, J = 19.2 Hz, 1F, *F*4).

IR: film, ṽ(cm^{-1}) = 3348 (w), 2932 (w), 2852 (w), 1621 (m), 1503 (m), 1477 (s), 1431 (m), 1372 (w), 1296 (m), 1242 (w), 1187 (w), 1151 (w), 1069 (s), 1049 (w), 1020 (w), 955 (w), 927 (m), 808 (s), 775 (w), 739 (w), 670 (w), 630 (w), 508 (w).

HRMS: APCI(–); *m/z* calcd. for $C_8H_7F_2O_2$ [M-H]$^-$: 173.0420, found: 173.0425.

3. 1-(bromomethyl)-3,4-difluoro-2-methoxybenzene (**37**)

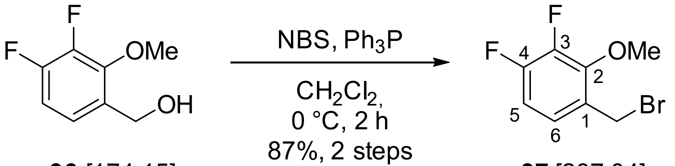

36 [174.15] **37** [237.04]

To a stirred solution of benzyl alcohol **36** (~4.21 mmol, 1.00 eq) in CH_2Cl_2 (20 mL) was added PPh_3 (2.21 g, 8.42 mmol, 2.00 eq) and NBS (1.50 g, 8.42 mmol, 2.00 eq) sequentially under argon at 0 °C and the resulting solution was stirred for 90 min. To the reaction mixture water (50 mL) was added and the aqueous layer was extracted with CH_2Cl_2 (3 × 60 mL). The combined extracts were washed with brine (30 mL), then dried over $MgSO_4$. The solvent was carefully removed at 100 mbar, the crude product was adsorbed onto silica gel and then purified by column chromatography (pentane/Et_2O 49:1) to give benzyl bromide **37** (869 mg, 3.67 mmol, 87% over 2 steps) as colorless liquid.

TLC: R_f = 0.59 (Pe/EtOAc 20:1).

^{1}H-NMR: 250 MHz, $CDCl_3$; δ (ppm) = 7.09-7.02 (m, 1H, *H*6), 6.84 (dt, *J* = 9.1, 7.2 Hz, 1H, *H*5), 4.48 (s, 2H, C*H*$_2$Br), 4.09 (d, *J* = 2.4 Hz, 3H, OMe).

^{13}C-NMR: 126 MHz, $CDCl_3$; δ (ppm) = 151.9 (dd, *J* = 251.0, 11.4 Hz, 1C, *C*3), 147.4 (dd, *J* = 7.0, 2.0 Hz, 1C, *C*2), 144.3 (dd, *J* = 249.7, 14.0 Hz, 1C, *C*4), 127.6-127.5 (m, 1C, *C*1), 124.9 (dd, *J* = 8.6, 4.0 Hz, 1C, *C*6), 111.3 (d, *J* = 17.5 Hz, 1C, *C*5), 61.7 (d, *J* = 7.0 Hz, 1C, O*Me*), 27.4 (d, *J* = 3.2 Hz, 1C, *C*H$_2$Br).

^{19}F-NMR: 235 MHz, $CDCl_3$; δ (ppm) = −134.5 (d, *J* = 19.1 Hz, 1F, *F*3), −154.0 (d, *J* = 19.1 Hz, 1F, *F*4).

IR: film, ṽ(cm^{-1}) = 2944 (w), 2842 (w), 1621 (w), 1505 (s), 1481 (m), 1433 (m), 1306 (m), 1246 (m), 1214 (w), 1188 (w), 1155 (w), 1126 (w), 1062 (s), 984 (w), 950 (w), 809 (w), 768 (w), 745 (w), 667 (w), 639 (w), 597 (w), 572 (w), 536 (w), 471 (w).

HRMS: EI(+); *m/z* calcd. for $C_8H_7BrF_2O$ [M]$^+$: 235.96483, found: 235.96521.

4. methyl 3-(3,4-difluoro-2-methoxybenzyl)-1,7,8-trifluoro-2-naphthoate (**38**)

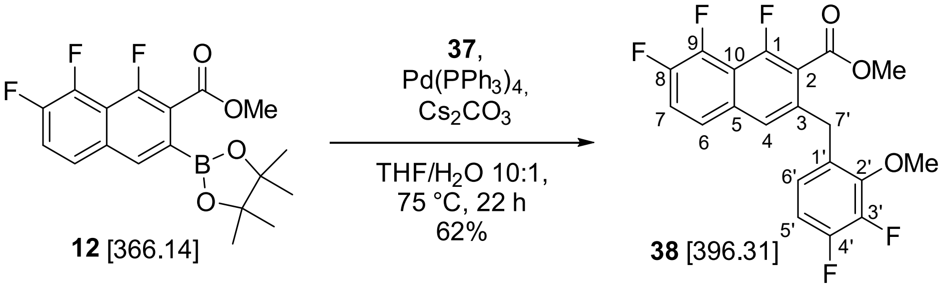

Benzyl bromide **37** (847 mg, 3.57 mmol, 1.00 eq), pinacol boronic ester **12** (79 w%, 1.85 g, 3.99 mmol, 1.12 eq), $Pd(PPh_3)_4$ (127 mg, 0.11 mmol, 3.0 mol%) and Cs_2CO_3 (3.49 g, 10.7 mmol, 3.00 eq) were suspended in THF (32 mL) and degassed H_2O (3.2 mL) under argon. The resulting mixture was degassed (3×) and then heated to 75 °C for 22 h. The suspension was allowed to cool to r.t. and H_2O (50 mL) and brine (20 mL) were added. The layers were separated and the aqueous layer was extracted with EtOAc (2 × 90 mL). The combined extracts were washed with brine (40 mL) and dried over $MgSO_4$. The solvent was removed under reduced pressure and the crude product was adsorbed onto silica gel before it was purified by column chromatography (pentane/EtOAc 19:1) to give **38** (875 mg, 2.21 mmol, 62%) as brownish solid.

TLC: R_f = 0.22 (Pe/EtOAc 20:1).

m.p.: 69 °C (Et_2O).

^{1}H-NMR: 500 MHz, $CDCl_3$; δ (ppm) = 7.50-7.47 (m, 1H, *H*6), 7.39 (dt, *J* = 9.4, 7.1 Hz, 1H, *H*7), 7.29 (s, 1H, *H*4), 6.81 (dt, *J* = 9.1, 7.1 Hz, 1H, *H*5'), 6.77-6.74 (m, 1H, *H*6'), 4.14 (s, 2H, *H*7'), 3.89 (s, 3H, CO_2Me), 3.85 (d, *J* = 2.3 Hz, 3H, OMe).

^{13}C-NMR: 126 MHz, $CDCl_3$; δ (ppm) = 165.8 (s, 1C, *C*O_2Me), 155.2 (ddd, *J* = 262.5, 6.9, 1.8 Hz, 1C, *C*1), 150.7 (dd, *J* = 248.3, 12.1 Hz, 1C, *C*3'), 147.7 (ddd, *J* = 248.5, 12.6, 0.6 Hz, 1C, *C*8), 147.2 (dd, *J* = 7.5, 1.1 Hz, 1C, *C*2'), 144.5 (ddd, *J* = 259.2, 13.5, 1.0 Hz, 1C, *C*9), 144.4 (dd, *J* = 249.0, 13.9 Hz, 1C, *C*4'), 136.3 (s, 1C, *C*3), 132.8 (d, *J* = 4.3 Hz, 1C, *C*10), 128.4 (d, *J* = 3.2 Hz, 1C, *C*1'), 124.5 (dd, *J* = 8.6, 3.3 Hz, 1C, *C*6'), 124.0-123.8 (m, 2C, *C*4 & *C*6), 119.6 (d, *J* = 21.1 Hz, 1C, *C*7), 119.3 (d, *J* = 15.4 Hz, 1C, *C*2), 113.6 (dd, *J* = 12.2, 9.1 Hz, 1C, *C*5), 110.9 (d, *J* = 17.1 Hz, 1C, *C*5'), 61.3 (d, *J* = 7.0 Hz, 1C, O*Me*), 52.9 (s, 1C, CO_2*Me*), 33.2 (s, 1C, *C*7').

^{19}F-NMR: 283 MHz, $CDCl_3$; δ (ppm) = −116.4 (dd, *J* = 57.7, 4.4 Hz, 1F, *F*8), −137.3 (d, *J* = 19.4 Hz, 1F, *F*3'), −138.6 (dd, *J* = 17.3, 4.5 Hz, 1F, *F*1), −143.1 (dd, *J* = 57.6, 17.3 Hz, 1F, *F*9), −154.1 (d, *J* = 19.3 Hz, 1F, *F*4').

IR: film, ṽ(cm^{-1}) = 2954 (w), 2841 (w), 1730 (s), 1649 (w), 1620 (m), 1581 (w), 1501 (s), 1476 (m), 1450 (w), 1432 (w), 1360 (m), 1260 (s), 1200 (w), 1176 (w), 1151 (w), 1130

(m), 1060 (s), 1002 (w), 949 (m), 881 (w), 802 (m), 769 (w), 742 (w), 712 (w), 687 (w), 651 (w), 604 (m), 553 (w), 505 (w), 448 (w).

HRMS: ESI(+); *m/z* calcd. for $C_{20}H_{13}F_5O_3Na$ $[M+Na]^+$: 419.0677, found: 419.0682.

5. methyl 3-(3,4-difluoro-2-hydroxybenzyl)-1,7,8-trifluoro-2-naphthoate (**39**)

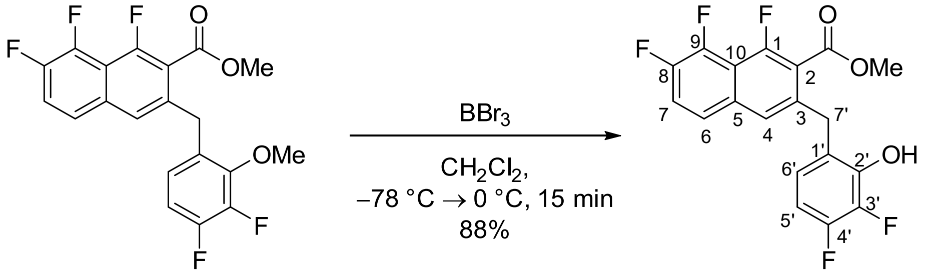

38 [396.31] **39** [396.31]

To a stirred solution of anisole **38** (20 mg, 50.5 µmol, 1.00 eq) in CH_2Cl_2 (1.0 mL) was added BBr_3 (1.0 M in CH_2Cl_2, 0.10 mL, 101 µmol, 2.00 eq) at −78 °C under argon and the solution was stirred at this temperature for 5 min. The mixture was allowed to warm up to 0 °C and stirred for further 10 min. The reaction was quenched with H_2O (10 mL) and the mixture was extracted with CH_2Cl_2 (4 × 10 mL). The combined organic layers were washed with brine (10 mL) and dried over $MgSO_4$. The solvent was removed under reduced pressure and the crude product was adsorbed onto silica gel before it was purified by column chromatography (pentane/EtOAc 7:1) to give phenol **39** (17 mg, 44.5 µmol, 88%) as colorless solid.

TLC: R_f = 0.33 (Pe/EtOAc 5:1).

m.p.: 184 °C (EtOAc).

^{1}H-NMR: 500 MHz, acetone-d$_6$; δ (ppm) = 9.20 (s, 1H, OH), 7.81-7.78 (m, 1H, *H*6), 7.66-7.60 (m, 2H, *H*4 & *H7*), 6.88-6.84 (m, 1H, *H*6’), 6.78-6.73 (m, 1H, *H*5’), 4.20 (s, 2H, *H*7’), 3.90 (s, 3H, CO_2Me).

^{13}C-NMR: 126 MHz, acetone-d$_6$; δ (ppm) = 166.0 (s, 1C, *C*O$_2$Me), 155.2 (ddd, *J* = 259.6, 6.4, 0.6 Hz, 1C, *C*1), 150.8 (dd, *J* = 244.5, 11.0 Hz, 1C, *C*3’), 148.4 (ddd, *J* = 245.4, 11.5, 0.6 Hz, 1C, *C*8), 145.2-145.0 (m, 1C, *C*2’), 144.9 (ddd, *J* = 256.8, 15.0, 0.6 Hz, 1C, *C*9), 141.3 (dd, *J* = 239.2, 14.6 Hz, 1C, *C*4’), 137.2 (s, 1C, *C*3), 133.9 (d, *J* = 4.0 Hz, 1C, *C*10), 125.9 (dd, *J* = 8.3, 3.8 Hz, 1C, *C*6’), 125.8-125.6 (m, 1C, *C*1’), 125.3 (m, 1C, *C*6), 125.0-124.9 (m, 1C, *C*4), 120.7 (d, *J* = 14.9 Hz, 1C, *C*2), 120.3 (dd, *J* = 20.7, 1.1 Hz, 1C, *C*7), 113.8 (dd, *J* = 12.1, 9.2 Hz, 1C, *C*5), 107.9 (d, *J* = 17.2 Hz, 1C, *C*5’), 53.1 (s, 1C, CO$_2$*Me*), 33.3 (s, 1C, *C*7’).

^{19}F-NMR: 283 MHz, acetone-d$_6$; δ (ppm) = −120.1 (dd, *J* = 57.0, 4.1 Hz, 1F, *F*8), −141.3 (dd, *J* = 16.9, 3.9 Hz, 1F, *F*1), −141.4 (d, *J* = 19.5 Hz, 1F, *F*3'), −146.6 (dd, *J* = 57.9, 16.9 Hz, 1F, *F*9), −162.9 - −163.0 (m, 1F, *F*4').

IR: film, ṽ(cm^{-1}) = 3576 (w), 3390 (w), 2957 (w), 2853 (w), 1720 (m), 1649 (w), 1621 (m), 1581 (w), 1510 (m), 1479 (s), 1450 (w), 1360 (s), 1281 (w), 1262 (s), 1204 (w), 1177 (w), 1149 (w), 1132 (m), 1061 (w), 1045 (s), 990 (w), 957 (m), 914 (w), 882 (w), 799 (m), 769 (w), 737 (w), 713 (w), 686 (w), 649 (w), 602 (m), 552 (w), 513 (w), 483 (w), 449 (w).

HRMS: ESI(+); *m/z* calcd. for $C_{19}H_{11}F_5O_3Na$ [M+Na]$^+$: 405.0521, found: 405.0532.

6. methyl 3-(3,4-difluoro-2-(((trifluoromethyl)sulfonyl)oxy)benzyl)-1,7,8-trifluoro-2-naphthoate (**40**)

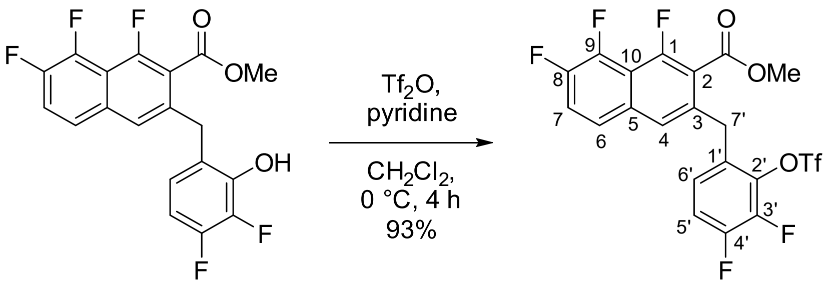

39 [396.31] **40** [514.34]

To a stirred solution of phenol **39** (87 mg, 228 µmol, 1.00 eq) and pyridine (0.05 mL, 627 µmol, 2.75 eq) in CH_2Cl_2 (2.1 mL) was added dropwise Tf_2O (0.05 mL, 273 µmol, 1.20 eq) at 0 °C under argon. The mixture was stirred for 4 h at 0 °C before it was quenched with sat. aq. $NaHCO_3$ (10 mL). The mixture was extracted with EtOAc (3 × 15 mL) and the combined extracts were washed with 2 M HCl (10 mL) and brine (10 mL). After drying over Na_2SO_4, the solvent was removed under reduced pressure and the residue was adsorbed onto silica gel. Purification by column chromatography (pentane/EtOAc 7:1) gave triflate **40** (109 mg, 212 µmol, 93%) as colorless solid.

TLC: R_f = 0.51 (Pe/EtOAc 5:1).

m.p.: 111 °C (EtOAc).

^{1}H-NMR: 500 MHz, $CDCl_3$; δ (ppm) = 7.55-7.53 (m, 1H, *H*6), 7.45 (dt, *J* = 9.4, 7.1 Hz, 1H, *H*7), 7.39 (s, 1H, *H*4), 7.13 (dt, *J* = 9.0, 7.4 Hz, 1H, *H*5'), 6.84-6.80 (m, 1H, *H*6'), 4.30 (s, 2H, *H*7'), 3.85 (s, 3H, CO_2Me).

^{13}C-NMR: 126 MHz, $CDCl_3$; δ (ppm) = 165.3 (s, 1C, *C*O_2Me), 155.9 (ddd, *J* = 264.9, 7.6, 0.3 Hz, 1C, *C*1), 149.9 (dd, *J* = 252.5, 10.9 Hz, 1C, *C*3'), 148.0 (dd, *J* = 249.1, 12.4 Hz, 1C, *C*8), 144.7 (ddd, *J* = 260.5, 14.9, 0.5 Hz, 1C, *C*9), 143.5 (dd, *J* = 256.3, 15.4 Hz, 1C, *C*4'), 136.1 (dd, *J* = 11.5, 2.0 Hz, 1C, *C*1'), 133.6 (s, 1C, *C*3), 132.8 (d, *J* = 4.0 Hz, 1C,

*C*10), 130.5 (m, 1C, *C*2'), 125.2 (dd, *J* = 7.5, 4.1 Hz, 1C, *C*6'), 125.0 (m, 1C, *C*4), 124.1-124.0 (m, 1C, *C*6), 120.1 (d, *J* = 21.1 Hz, 1C, *C*7), 118.8 (d, *J* = 14.3 Hz, 1C, *C*2), 118.6 (q, *J* = 320.6 Hz, 1C, CF_3), 117.0 (d, *J* = 16.0 Hz, 1C, *C*5'), 114.3-114.2 (m, 1C, *C*5), 53.0 (s, 1C, CO_2Me), 33.4 (s, 1C, *C*7').

^{19}F-NMR: 283 MHz, $CDCl_3$; δ (ppm) = −72.3 (d, *J* = 13.0 Hz, 3F, OTf), −114.4 (dd, *J* = 58.5, 3.9 Hz, 1F, *F*8), −134.1 (d, *J* = 20.8 Hz, 1F, *F*3'), −137.5 (dd, *J* = 16.9, 5.2 Hz, 1F, *F*1), −142.5 (dd, *J* = 59.2, 16.9 Hz, 1F, *F*9), −147.0 - −147.3 (m, 1F, *F*4').

IR: film, ṽ(cm^{-1}) = 2959 (w), 1731 (m), 1649 (w), 1622 (m), 1582 (w), 1511 (m), 1458 (w), 1424 (m), 1362 (m), 1263 (w), 1213 (s), 1178 (w), 1131 (s), 1063 (m), 1024 (m), 1001 (w), 962 (w), 947 (w), 922 (w), 884 (w), 822 (s), 766 (w), 738 (w), 713 (w), 674 (w), 655 (w), 632 (w), 596 (m), 515 (w), 498 (w), 448 (w).

HRMS: APCI(+); *m/z* calcd. for $C_{20}H_{10}F_8O_5SNa$ $[M+Na]^+$: 537.0013, found: 537.0039.

7. 2,3-difluoro-6-((4,5,6-trifluoro-3-(hydroxymethyl)naphthalen-2-yl)methyl)phenyl trifluoromethanesulfonate (**41**)

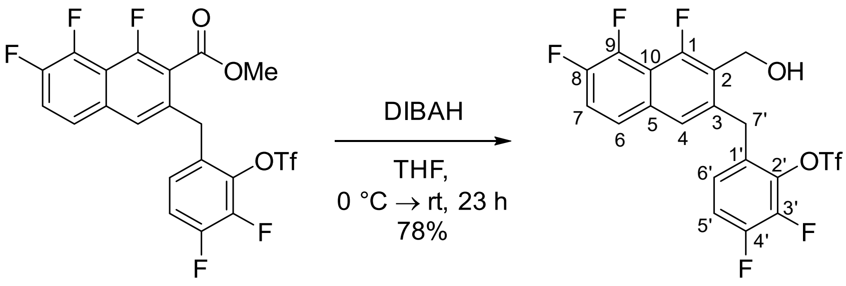

40 [514.34] **41** [486.33]

To a stirred solution of ester **40** (90 mg, 175 µmol, 1.00 eq) in THF (1.0 mL) DIBAH (1.0 M in toluene, 0.44 mL, 437 µmol, 2.50 eq) was added dropwise at 0 °C under argon and the resulting solution was warmed to r.t. Since the conversion was not complete after 2.5 h additional DIBAH (0.09 mL, 87.5 µmol, 0.50 eq) was added dropwise. After stirring for 20 h the reaction was quenched with H_2O (20 mL) and acidified using 2 M HCl (5 mL). The mixture was extracted with EtOAc (4 × 25 mL). The combined organic extracts were washed with brine (20 mL), dried over Na_2SO_4 and the solvent was removed under reduced pressure. The crude product was adsorbed onto silica gel and then purified via column chromatography (pentane/EtOAc 5:1) to give alcohol **41** (66 mg, 136 µmol, 78%) as colorless solid.

TLC: R_f = 0.32 (Pe/EtOAc 5:1).

m.p.: 83 °C (EtOAc).

^{1}H-NMR: 500 MHz, $CDCl_3$; δ (ppm) = 7.49-7.46 (m, 1H, *H*6), 7.37 (dt, *J* = 9.5, 7.1 Hz, 1H, *H*7), 7.26 (s, 1H, *H*4), 7.14 (dt, *J* = 9.0, 7.4 Hz, 1H, *H*5'), 6.83-6.79 (m, 1H, *H*6'), 4.85 (d, *J* = 2.6 Hz, 2H, C*H*$_2$OH), 4.40 (s, 2H, *H*7').

^{13}C-NMR: 126 MHz, $CDCl_3$; δ (ppm) = 155.9 (ddd, *J* = 257.0, 7.3, 1.2 Hz, 1C, *C*1), 150.0 (dd, *J* = 251.6, 11.2 Hz, 1C, *C*3'), 147.8 (ddd, *J* = 247.7, 11.8, 0.4 Hz, 1C, *C*8), 144.4 (ddd, *J* = 259.4, 14.7, 0.5 Hz, 1C, *C*9), 143.6 (dd, *J* = 256.4, 15.5 Hz, 1C, *C*4'), 136.2 (dd, *J* = 10.7, 2.8 Hz, 1C, *C*1'), 136.0 (d, *J* = 1.8 Hz, 1C, *C*3), 132.2 (d, *J* = 4.4 Hz, 1C, *C*10), 131.0-130.9 (m, 1C, *C*2'), 125.1 (dd, *J* = 7.2, 4.2 Hz, 1C, *C*6'), 124.7-124.6 (m, 1C, *C*4), 124.0-123.8 (m, 1C, *C*6), 123.4 (d, *J* = 13.2 Hz, 1C, *C*2), 118.7 (dd, *J* = 21.2, 1.8 Hz, 1C, *C*7), 118.7 (q, *J* = 320.7 Hz, 1C, *C*F_3), 117.1 (d, *J* = 16.7 Hz, 1C, *C*5'), 114.4 (ddd, *J* = 14.7, 9.0, 1.2 Hz, 1C, *C*5), 55.3 (d, *J* = 9.1 Hz, 1C, *C*H_2OH), 32.4 (s, 1C, *C*7').

^{19}F-NMR: 283 MHz, $CDCl_3$; δ (ppm) = −72.7 (d, *J* = 14.3 Hz, 3F, OTf), −121.3 (dd, *J* = 59.6, 4.2 Hz, 1F, *F*8), −134.7 (d, *J* = 19.2 Hz, 1F, *F*3'), −139.3 (dd, *J* = 16.9, 4.5 Hz, 1F, *F*1), −144.3 (dd, *J* = 59.2, 16.9 Hz, 1F, *F*9), −147.4 - −147.7 (m, 1F, *F*4').

IR: film, ṽ(cm^{-1}) = 3609 (w), 3380 (w), 2923 (w), 1626 (m), 1582 (w), 1511 (m), 1477 (w), 1460 (w), 1421 (m), 1356 (m), 1299 (w), 1262 (w), 1216 (s), 1133 (s), 1083 (m), 1039 (w), 1015 (s), 964 (w), 922 (w), 885 (w), 824 (s), 764 (w), 737 (w), 713 (w), 669 (w), 632 (w), 600 (m), 516 (w), 496 (w).

HRMS: EI(+); *m/z* calcd. for $C_{19}H_{10}F_8O_4S$ $[M]^+$: 486.01720, found: 486.01775.

8. 2,3-difluoro-6-((4,5,6-trifluoro-3-formylnaphthalen-2-yl)methyl)phenyl trifluoromethane-sulfonate (**42**)

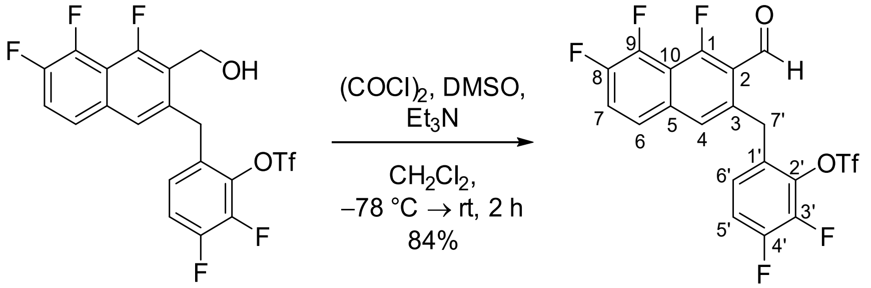

41 [486.33] **42** [484.32]

To a stirred solution of DMSO (19.2 µL, 271 µmol, 3.00 eq) in CH_2Cl_2 (1.6 mL) was added oxalyl chloride (11.7 µL, 136 µmol, 1.50 eq) at –78 °C under argon. After 10 min the reaction mixture was added to a solution of alcohol **41** (44 mg, 90.5 µmol, 1.00 eq) in CH_2Cl_2 (1.6 mL) at –78 °C and stirred for 30 min. Then Et_3N (62.8 µL, 453 µmol, 5.00 eq) was added dropwise and the reaction mixture was stirred additional 30 min at –78 °C. The reaction was allowed to warm up to rt and was

stirred at that temperature for 15 min. Then H_2O (20 mL) and brine (10 mL) were added and the mixture was extracted with CH_2Cl_2 (3 × 25 mL). The combined organic extracts were washed with brine (20 mL), dried over $MgSO_4$ and the solvent was removed under reduced pressure. The crude product was adsorbed onto silica gel and then purified via column chromatography (pentane/EtOAc 10:1) to give aldehyde **42** (37 mg, 76.4 µmol, 84%) as colorless solid.

TLC: R_f = 0.62 (Pe/EtOAc 5:1).

m.p.: 134 °C (EtOAc).

^{1}H-NMR: 500 MHz, $CDCl_3$; δ (ppm) = 10.63 (s, 1H, CHO), 7.57-7.54 (m, 2H, *H*6 & *H*7), 7.34 (s, 1H, *H*4), 7.10-7.05 (m, 1H, *H*5'), 6.70-6.66 (m, 1H, *H*6'), 4.55 (s, 2H, *H*7').

^{13}C-NMR: 126 MHz, $CDCl_3$; δ (ppm) = 188.0 (d, *J* = 19.4 Hz, 1C, *C*HO), 164.2 (ddd, *J* = 272.6, 7.1, 1.7 Hz, 1C, *C*1), 149.8 (dd, *J* = 251.3, 11.0 Hz, 1C, *C*3'), 148.1 (dd, *J* = 250.0, 12.0 Hz, 1C, *C*8), 144.8 (dd, *J* = 262.6, 14.5 Hz, 1C, *C*9), 143.6 (dd, *J* = 256.0, 15.5 Hz, 1C, *C*4'), 136.3 (dd, *J* = 11.1, 2.5 Hz, 1C, *C*1'), 134.7 (s, 1C, *C*3), 134.3 (d, *J* = 6.3 Hz, 1C, *C*10), 131.1 (dd, *J* = 4.2, 2.1 Hz, 1C, *C*2'), 126.2 (m, 1C, *C*4), 124.3-124.2 (m, 2C, *C*6 & *C*6'), 121.8 (d, *J* = 20.7 Hz, 1C, *C*7), 119.3 (s, 1C, *C*2), 118.7 (q, *J* = 320.6 Hz, 1C, *C*F_3), 116.8 (d, *J* = 17.0 Hz, 1C, *C*5'), 114.3-114.2 (m, 1C, *C*5), 33.6 (s, 1C, *C*7').

^{19}F-NMR: 283 MHz, $CDCl_3$; δ (ppm) = −72.4 (d, *J* = 13.0 Hz, 3F, OTf), −121.1 (dd, *J* = 63.1, 4.0 Hz, 1F, *F*8), −134.8 (d, *J* = 20.8 Hz, 1F, *F*3'), −136.8 (dd, *J* = 16.7, 3.7 Hz, 1F, *F*1), −140.5 (dd, *J* = 63.1, 17.0 Hz, 1F, *F*9), −147.1 - −147.3 (m, 1F, *F*4').

IR: film, ṽ(cm^{-1}) = 3090 (w), 2895 (w), 1694 (s), 1649 (w), 1614 (m), 1583 (w), 1511 (m), 1473 (m), 1423 (m), 1360 (m), 1299 (m), 1265 (w), 1215 (s), 1134 (s), 1085 (m), 1047 (w), 1022 (m), 1001 (w), 961 (w), 923 (w), 890 (w), 824 (s), 789 (w), 765 (w), 737 (w), 704 (w), 674 (w), 657 (w), 632 (w), 600 (m), 515 (w), 499 (w), 442 (w).

HRMS: EI(+); *m/z* calcd. for $C_{19}H_8F_8O_4S$ $[M]^+$: 484.00155, found: 484.00151.

9. 3,4,6,7,8-pentafluoro-5,12-dihydrotetracen-5-ol (**43**)

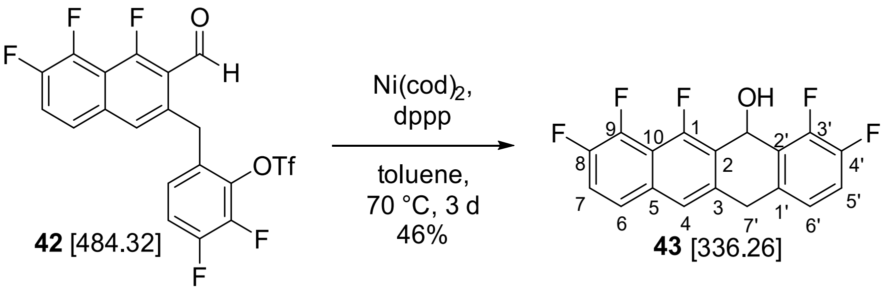

42 [484.32] **43** [336.26]

Aldehyde **42** (50 mg, 103 µmol, 1.00 eq) and dppp (51 mg, 124 µmol, 1.20 eq) were evacuated and backfilled with argon three times. In a glovebox $Ni(cod)_2$ (28 mg, 103 µmol, 1.00 eq) and toluene (1.4 mL) were added sequentially. The reaction mixture was heated to 70 °C for 3 d and was then allowed to cool to r.t. before it was diluted with EtOAc (20 mL). The solvent was removed under reduced pressure and the crude product was adsorbed onto silica gel before it was purified by column chromatography (pentane/EtOAc 3:1) to give alcohol **43** (16 mg, 47.6 µmol, 46%) as yellowish solid.

TLC: R_f = 0.27 (Pe/EtOAc 3:1).

m.p.: 160 °C decomposition (EtOAc).

^{1}H-NMR: 500 MHz, acetone-d$_6$; δ (ppm) = 7.81-7.77 (m, 2H, *H*4 & *H*6), 7.60-7.54 (m, 1H, *H7*), 7.31-7.27 (m, 2H, *H*5' & *H*6'), 6.65 (d, *J* = 5.4 Hz, 1H, C*H*OH), 4.40 (d, *J* = 18.2 Hz, 1H, H_a7'), 4.17 (d, *J* = 18.2 Hz, 1H, H_b7'), 2.79 (br s, 1H, OH).

^{13}C-NMR: 126 MHz, acetone-d$_6$; δ (ppm) = 155.3 (ddd, *J* = 255.8, 5.7, 0.6 Hz, 1C, *C*1), 149.9 (dd, *J* = 245.0, 13.2 Hz, 1C, *C*3'), 149.2 (dd, *J* = 247.8, 13.0 Hz, 1C, *C*8), 148.2 (ddd, *J* = 244.7, 11.4, 1.8 Hz, 1C, *C*9), 144.9 (ddd, *J* = 255.7, 14.6, 1.8 Hz, 1C, *C*4'), 138.1 (s, 1C, *C*1'), 136.1 (s, 1C, *C*3), 133.4 (s, 1C, *C*10), 128.4 (dd, *J* = 12.7, 3.0 Hz, 1C, *C*2'), 125.5-125.4 (m, 1C, *C*6), 124.8-124.7 (m, 1C, *C*6'), 124.0 (dd, *J* = 13.6, 3.0 Hz, 1C, *C*2), 122.8-122.7 (m, 1C, *C*4), 119.3 (dd, *J* = 21.5, 1.7 Hz, 1C, *C*7), 117.5 (d, *J* = 17.7 Hz, 1C, *C*5'), 114.3 (dd, *J* = 13.9, 9.5 Hz, 1C, *C*5), 57.2-56.9 (m, 1C, *C*HOH), 34.3 (s, 1C, *C*7').

^{19}F-NMR: 283 MHz, acetone-d$_6$; δ (ppm) = −126.2 (dt, *J* = 56.5, 5.2 Hz, 1F, *F*8), −142.6 (dd, *J* = 17.4, 5.1 Hz, 1F, *F*1), −142.9 (d, *J* = 20.8 Hz, 1F, *F*3'), −147.2 (dd, *J* = 57.6, 16.8 Hz, 1F, *F*9), −147.4 (dd, *J* = 20.6, 4.3 Hz, 1F, *F*4').

IR: film, ṽ(cm^{-1}) = 3600 (w), 3363 (br, w), 2934 (w), 1694 (w), 1629 (s), 1581 (w), 1501 (s), 1483 (w), 1457 (w), 1421 (w), 1355 (s), 1319 (w), 1260 (s), 1226 (w), 1177 (w), 1151 (w), 1091 (m), 1053 (w), 1035 (w), 1003 (m), 979 (w), 941 (w), 870 (w), 822 (w),

806 (m), 777 (w), 727 (m), 691 (w), 639 (w), 605 (m), 560 (w), 532 (w), 513 (w), 482 (w), 440 (w), 421 (w).

HRMS: EI(+); *m/z* calcd. for $C_{18}H_9F_5O$ $[M]^+$: 336.05736, found: 336.05783.

10. 1,2,9,10,11-pentafluorotetracene (F_5TET, **2**)

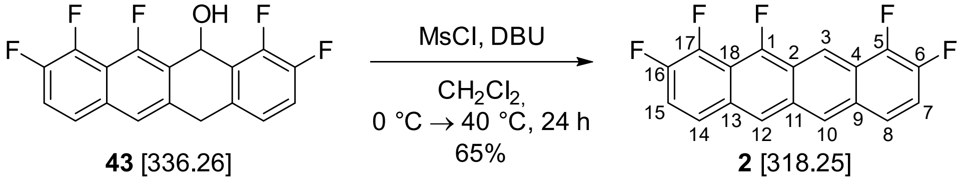

43 [336.26] **2** [318.25]

Alcohol **43** (13 mg, 38.7 µmol, 1.00 eq) was suspended in CH_2Cl_2 (1.0 mL) and DBU (28.8 µL, 193 µmol, 5.00 eq) and MsCl (8.98 µL, 116 µmol, 3.00 eq) were added sequentially at 0 °C under argon. The reaction mixture was heated to 40 °C and stirred for 24 h under exclusion of light during which an orange solid deposed. The solid was centrifuged and washed with H_2O (2 × 1.0 mL) and acetone (2 × 1 mL) to give F_5TET **2** (8 mg, 25.1 µmol, 65%) as orange solid.

TLC: R_f = 0.40 (Pe/EtOAc 5:1).

m.p.: 282 °C decomposition (EtOAc).

^{1}H-NMR: 500 MHz, CD_2Cl_2; δ (ppm) = 9.11 (s, 1H, *H*3 or *H*10), 8.74 (s, 1H, *H*3 or *H*10), 8.54 (s, 1H, *H*12), 7.87-7.80 (m, 2H, *H*8 & *H*14), 7.41-7.31 (m, 2H, *H*7 & *H*15).

^{13}C-NMR: due to very low solubility no ^{13}C-NMR could be obtained.

^{19}F-NMR: 283 MHz, CD_2Cl_2; δ (ppm) = −124.1 - −124.3 (m, 1F, *F*16), −140.6 - −140.7 (m, 2F, *F*1 & *F*5), −147.3 (dd, *J* = 53.7, 13.1 Hz, 1F, *F*17), −150.5 (d, *J* = 15.2 Hz, 1F, *F*6).

IR: neat, ṽ(cm^{-1}) = 1650 (s), 1587 (m), 1547 (w), 1471 (s), 1447 (w), 1412 (w), 1396 (w), 1351 (s), 1312 (w), 1281 (w), 1251 (s), 1227 (w), 1171 (w), 1115 (w), 1089 (m), 1031 (w), 1016 (s), 968 (m), 889 (m), 860 (m), 827 (w), 787 (m), 731 (m), 694 (m), 644 (m), 595 (m), 580 (w), 520 (m), 433 (w).

HRMS: APCI(+); *m/z* calcd. for $C_{18}H_7F_5$ $[M]^+$: 318.0468, found: 318.0474.

UV-VIS: CH_2Cl_2, λ_{max} (nm) = 380, 401, 425, 452, 482.

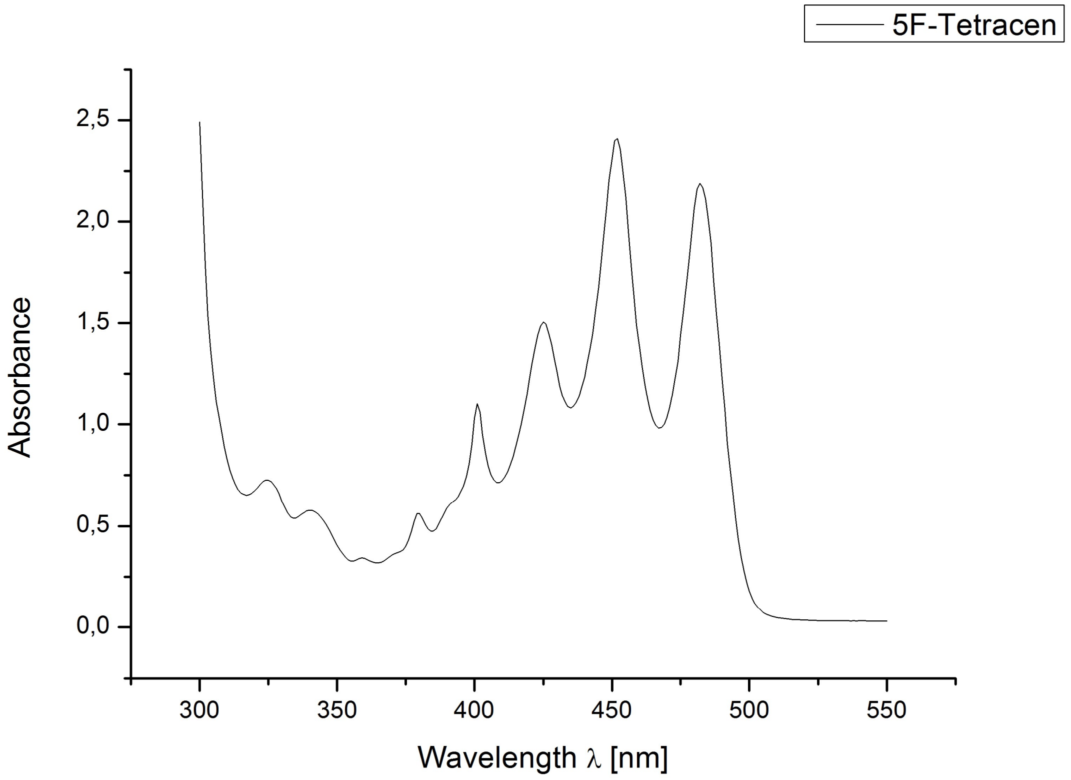
5F-Tetracen
Absorbance
2,5
2,0
1,5
1,0
0,5
0,0
300
350
400
450
500
550
Wavelength λ [nm]

6. Mechanistic proposal

Using the published conditions[13] with Ni(cod)$_2$, Triphos and tetramethylpiperidine in boiling toluene resulted in complete decomposition of the starting material for the final ring closure. By decreasing the reaction temperature to 70 °C a new compound was isolated which did not feature a carbonyl group. By showing a diastereotopic benzylic CH$_2$ group in the ^{1}H-NMR, alcohol **17** was identified. Low yields of **17** correlated with the substoichiometric amounts of Ni-catalyst. Therefore a stoichiometric addition of Ni(0) reagent was applied resulting in a better yield. Best results were obtained using dppp as ligand and quinuclidine as base in toluene at 70 °C. Mechanistically we think that after oxidative addition of Ni(0) into the CO-triflate bond a Barbier-type attack to the aldehyde group occurs (Scheme S2).[14-18] In contrast to the results of Newman *et al.* the β-H-elimination of the formed nickel alcoholate to a ketone **III** is hindered in this case. The Barbier-type reaction pathway would also explain the necessity of stoichiometric amounts of Ni(cod)$_2$.

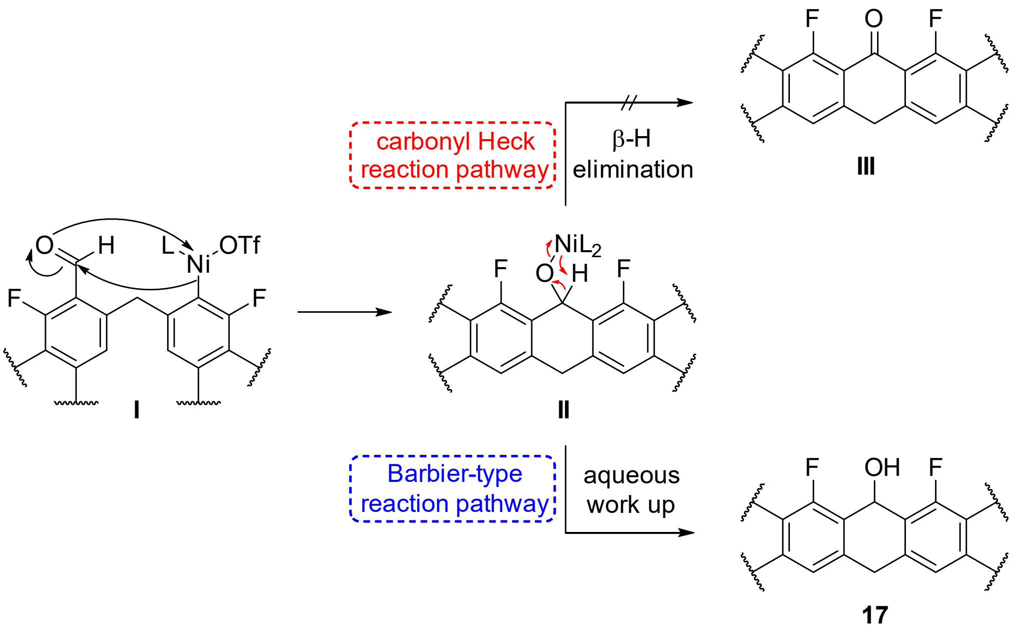

Scheme S2. Mechanistic proposal for the Ni(0)-catalyzed ring closure.

7. Cystallographic Data

1. F_6PEN (**1**) and F_5TET (**2**)

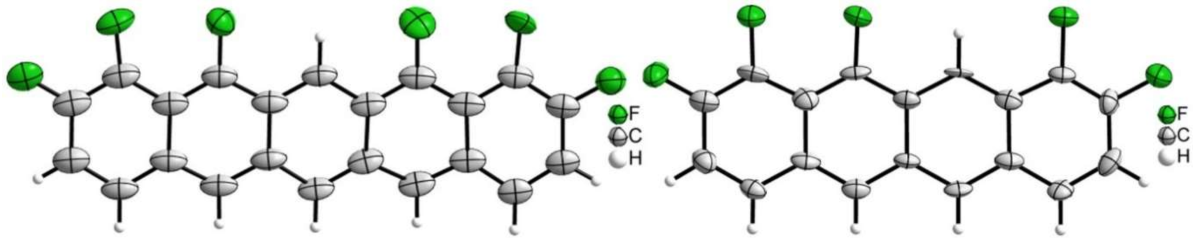

Figure S1. Molecules of F_6PEN (left) and F_5TET (right). Disordered fluorine and hydrogen atoms in F_5TET are omitted for clarity. Displacement ellipsoids are shown at the 50% probability level at 100 K.

Note on the refinement of F_6PEN (**1**):

The crystals of F_6PEN appeared as slightly bent, extremely thin needles. Although we attempted to select or cut a visually non-bent smaller crystal, most reflections in the diffraction experiment at higher diffraction angles 2θ were noticeably smeared and weak. This fact significantly impaired the quality of the dataset. The following unit cell was selected: monoclinic primitive, a = 6.8140(14), b = 3.6768(4), c = 29.714(5) Å, β = 94.875(14)°, V = 741.8(2) Å^3 at 100 K. The cell choice was confirmed by profile fitting of the powder diffraction pattern (Figure S1), which resulted in the following refined cell parameters: a = 6.8742(9), b = 3.7454(4), c = 29.667(5) Å, β = 95.135(5)°, V = 760.77(17) Å^3 at 293 K. It was found later that the compound (**2**), F_5TET, crystallizes isotypic, which increased the confidence in the refinement of (**1**).

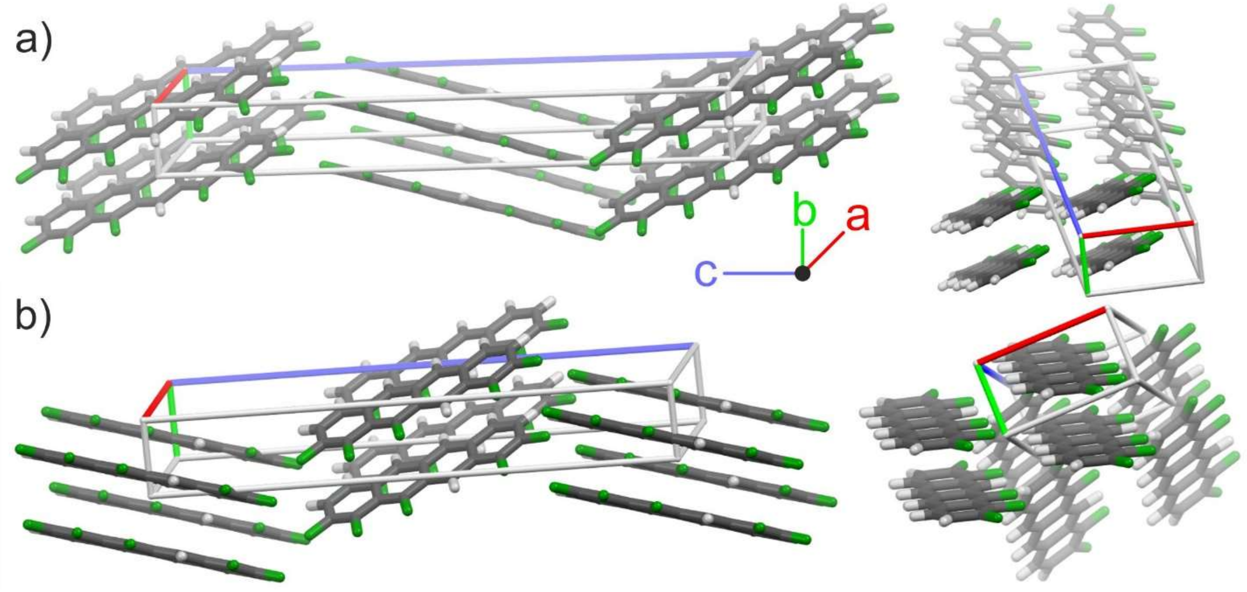

Figure S2. Crystal structures of a) **1** (F_6PEN) and b) **2** (F_5TET).

Note on the refinement of F_5TET (**2**):

The crystals of F_5TET appeared to have a similar habitus as the crystals of (**1**). Despite that, the diffraction quality was found to be considerably better, so that full crystal structure determination was possible. The compound (**2**) crystallizes isotypic to (**1**) and has the following lattice parameters: space group *Pn* (No. 7), a = 6.7840(8), b = 3.6780(4), c = 25.031(3) Å, β = 94.241(10)°, V = 622.86(13) Å^3 , Z = 2 at 100 K. The molecule of F_5TET is disordered in the crystal structure between two equally possible orientations with the central hydrogen atom closer either to the left or to the right side of the molecule (Fig. S1).

Table S1. Selected crystallographic data and details of the structure determination for **1** and **2**.

Compound	F_6PEN* (**1**)	F_5TET (**2**)
CCDC Number	1998747	1998748
Empirical formula	$C_{22}H_8F_6$	$C_{18}H_7F_5$
Molar mass / g·mol^{-1}	386.28	318.24
Space group (No.)	*Pn* (7)	*Pn* (7)
a / Å	6.8140(14)	6.7840(8)
b / Å	3.6768(4)	3.6780(4)
c / Å	29.714(5)	25.031(3)
β / °	94.875(14)	94.241(10)
V / Å^3	741.8(2)	622.86(13)
Z	2	2
$\rho_{calc.}$ / g·cm^{-3}	1.730	1.697
$\rho_{exp.}$ / g·cm^{-3}	1.722(16)	not measured
μ / mm^{-1}	1.340	1.321
Color	blue	orange
Crystal habitus	needle	needle
Crystal size / mm^3	0.100×0.020×0.001	0.080×0.010×0.002
T / K	100	100
λ / Å	1.54186 (Cu-K$_\alpha$)	1.54186 (Cu-K$_\alpha$)
θ range / °	5.979 to 40.862	3.541 to 66.566
Range of Miller indices	$-5 \le h \le 5$<br>$-3 \le k \le 3$<br>$-24 \le l \le 24$	$-8 \le h \le 8$<br>$-4 \le k \le 1$<br>$-29 \le l \le 29$
Absorption correction	multi-scan	multi-scan
T_{min}, T_{max}	0.1024, 1.0000	0.902, 0.997
R_{int}, R_σ	0.1059, 0.0805	0.0808, 0.0777
Completeness of the data set	0.970	0.972
No. of measured reflections	1703	10553
No. of independent reflections	764	2082
No. of parameters	253	218
No. of restrains	534	5
S (all data)	0.836	0.911
$R(F)$ ($I \ge 2\sigma(I)$, all data)	0.1756, 0.1915	0.0627, 0.1116
$wR(F^2)$ ($I \ge 2\sigma(I)$, all data)	0.4348, 0.4641	0.1491, 0.1682
Flack parameter x	2.7(10)**	0.17(12)
Extinction coefficient	not refined	not refined
$\Delta\rho_{max}$, $\Delta\rho_{min}$ / e·Å^{-3}	0.826, –0.684	0.332, –0.319

* Due to the crystal quality only a heavily restrained refinement was possible.
** Not representative due to quality of the dataset

2. Powder X-ray pattern of F_6PEN (**1**)

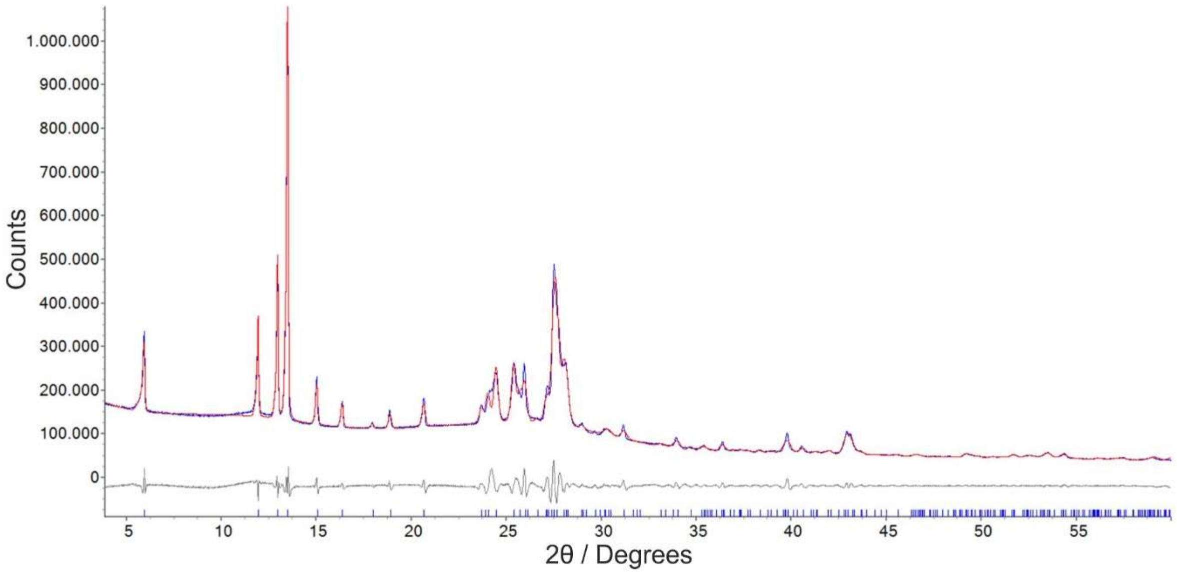

Figure S3. Observed (blue line) and calculated (red line) powder X-ray diffraction pattern of F_6PEN at 293 K after Pawley profile fitting. The calculated reflection positions are indicated by the vertical bars below the pattern. The curve at the bottom represents the difference between the observed and the calculated intensities. R_p = 0.0269, R_{wp} = 0.0396, background-corrected values: cR_p = 0.1231, cR_{wp} = 0.1242.

3. 4,5,6-Trifluoro-3-(methoxymethoxy)-2-naphthaldehyde (13)[19]

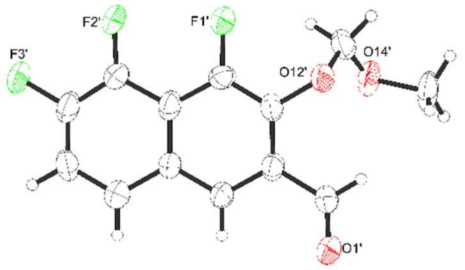

Crystal data:		
Habitus, colour	needle, colorless	
Crystal size	0.21 × 0.04 × 0.04 mm^3	
Crystal system	Triclinic	
Space group	P-1	Z = 4
Unit cell dimensions	a = 4.3889(6) Å	α = 70.143(11)°.
	b = 14.138(2) Å	β = 84.832(11)°.
	c = 19.228(3) Å	γ = 81.781(11)°.
Volume	1109.5(3) Å^3	
Cell determination	6913 peaks with Theta 3.3 to 76.2°.	
Empirical formula	$C_{13}H_9F_3O_3$	
Moiety formula	$C_{13}H_9F_3O_3$	
Formula weight	270.20 g/mol	
Density (calculated)	1.618 Mg/m^3	
Absorption coefficient	1.291 mm^{-1}	
F(000)	552	
Data collection:		
Diffractometer type	STOE STADIVARI	
Wavelength	1.54186 Å	
Temperature	100(2) K	
Theta range for data collection	3.348 to 67.739°.	
Index ranges	-5<=h<=5, -16<=k<=8, -22<=l<=20	
Data collection software	X-Area Pilatus3_SV 1.31.127.0 (STOE, 2016)	
Cell refinement software	X-Area Recipe 1.33.0.0 (STOE, 2015)	
Data reduction software	X-Area Integrate 1.71.0.0 (STOE, 2016)	
	X-Area LANA 1.68.2.0 (STOE, 2016)	
Solution and refinement:		
Reflections collected	17397	
Independent reflections	3998 [R_{int} = 0.1286]	
Completeness to theta = 67.686°	99.2%	
Observed reflections	1821[$I > 2\sigma(I)$]	
Reflections used for refinement	3998	
Absorption correction	Semi-empirical from equivalents	
Max. and min. transmission	1.0000 and 0.0338	
Largest diff. peak and hole	$\Delta\rho_{max}$ = 0.392 and $\Delta\rho_{min}$ = –0.453 e.Å^{-3}	
Solution	dual space algorithm	
Refinement	Full-matrix least-squares on F^2	
Treatment of hydrogen atoms	Calculated positions, constr ref.	
Programs used	XT V2014/1 (Bruker AXS Inc., 2014)	
	SHELXL-2017/1 (Sheldrick, 2017)	
	DIAMOND (Crystal Impact)	
	ShelXle (Hübschle, Sheldrick, Dittrich, 2011)	
Data / restraints / parameters	3998 / 0 / 345	
Goodness-of-fit on F^2	1.011	
R index (all data)	*wR2* = 0.2229	
R index conventional [$I>2\sigma(I)$]	*R1* = 0.0832	

8. Determination of Sublimation Enthalpy

The sublimation enthalpy of F_6PEN was determined via the Knudsen method, which is described in literature.[20] F_6PEN powder was heated in a Knudsen cell (realized as an aluminium crucible with small pinhole) under vacuum conditions and the resulting vapor pressure, which is proportional to the deposition rate, is recorded by a QCM. In order to measure the vapor pressure under equilibrium conditions, the Knudsen cell is heated and kept on the desired temperature for ca. 15 minutes before each measurement to guarantee thermal equilibrium. For each data point, the molecular flux was measured for 4 minutes and subtracted from the dark flux, which we obtained by closing the pinhole and also was measured for 4 minutes.

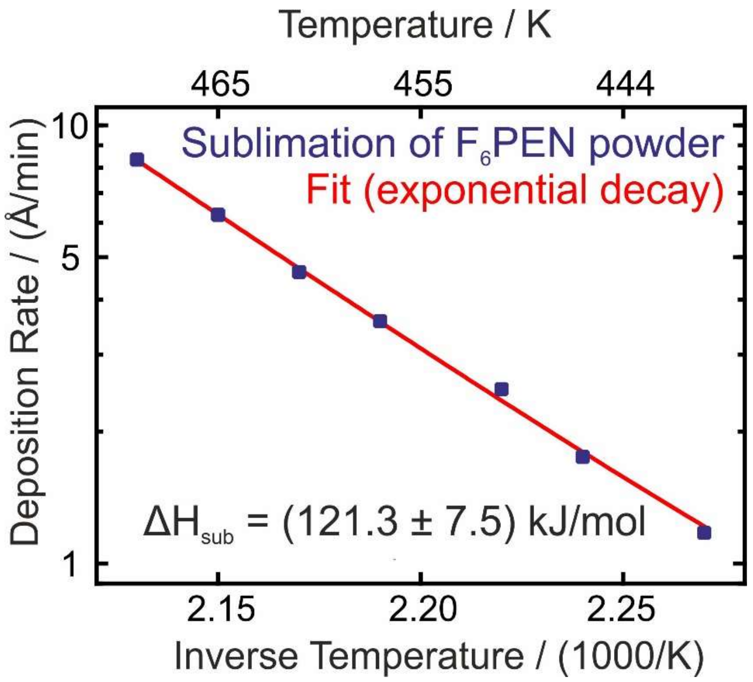

Figure S4. Determination of the sublimation enthalpy of F_6PEN via the Knudsen method.

In figure S4 the deposition is shown against the inverse temperature. In logarithmic scale a linear behavior is visible, indicating the presence of an exponential relation. From the slope of the curve a sublimation enthalpy of ΔH_{sub} = (121.3 ± 7.5) kJ/mol can be obtained.

9. Quantum Chemical Calculations

The electronic structures of compounds PEN, F_6PEN (**1**), PFP, TET, F_5TET (**2**) and PFTET have been analyzed theoretically in the frame of DFT-calculations carried out with an aug-cc-pVTZ basis set, using the B3LYP functional as implemented in the US GAMESS-code.[21-22] In each case the structure of the individual molecules (i.e. gas phase) was optimized using the highest available symmetry. Based on these data precise energy levels and dipole moments, as well as frontier orbitals and molecular electrostatic potentials (MEPs) are derived. The orbital visualizations were performed with the MacMolPlt package,[23] whereas the MEPs are generated by Molekel.[24]

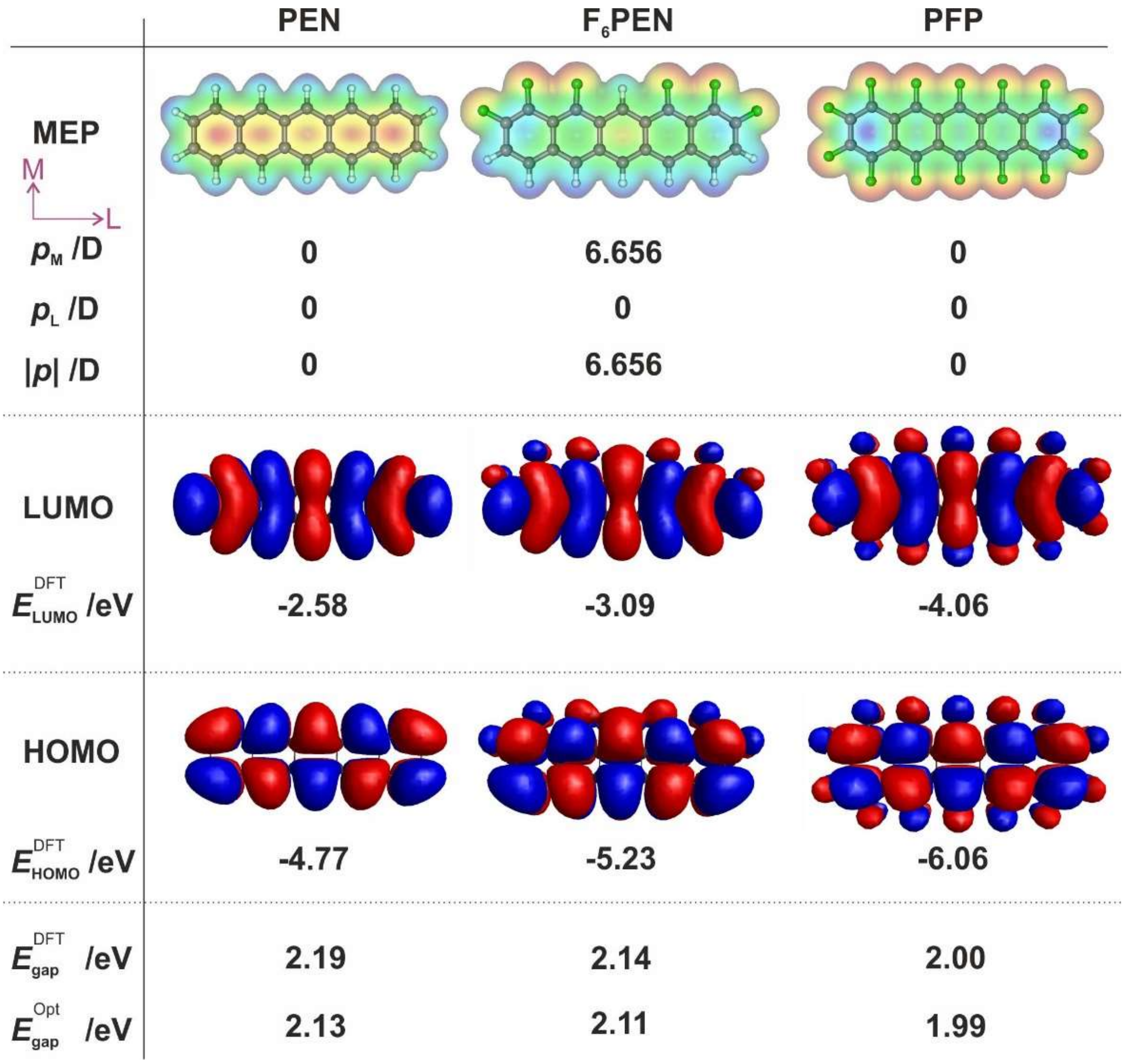

Figure S5. Comparison of electronic properties of pentacene derivatives.

The calculated MEPs show that the electrostatic potential is high at the hydrogen atoms, indicating a deficit of electrons and low at the fluorine atoms, indicating a surplus of electrons. Hence the charge redistribution due to fluorination is clearly visible, which is in particular present in comparison of PEN with PFP. In this view, F_6PEN appears to be an interlink between PEN and PFP, as it exhibits a PEN-like charge distribution on one side and a PFP-like charge distribution on the other side. The asymmetric charge distribution of the partially fluorinated F_6PEN leads to a strong permanent dipole moment. Also considering the electronic structure F_6PEN is a connecting link between PEN and PFP, since the frontier orbitals exhibit the same π-backbone. The fluorine atoms contribute to the π-system, leading to a significant shift of the absolute energies of the frontier orbitals. As this shift affects both the HOMO and the LUMO, the band gap only changes slightly due to fluorination. Similar observations are also observed in the case of F_5TET, which is shown together with TET and PFTET below.

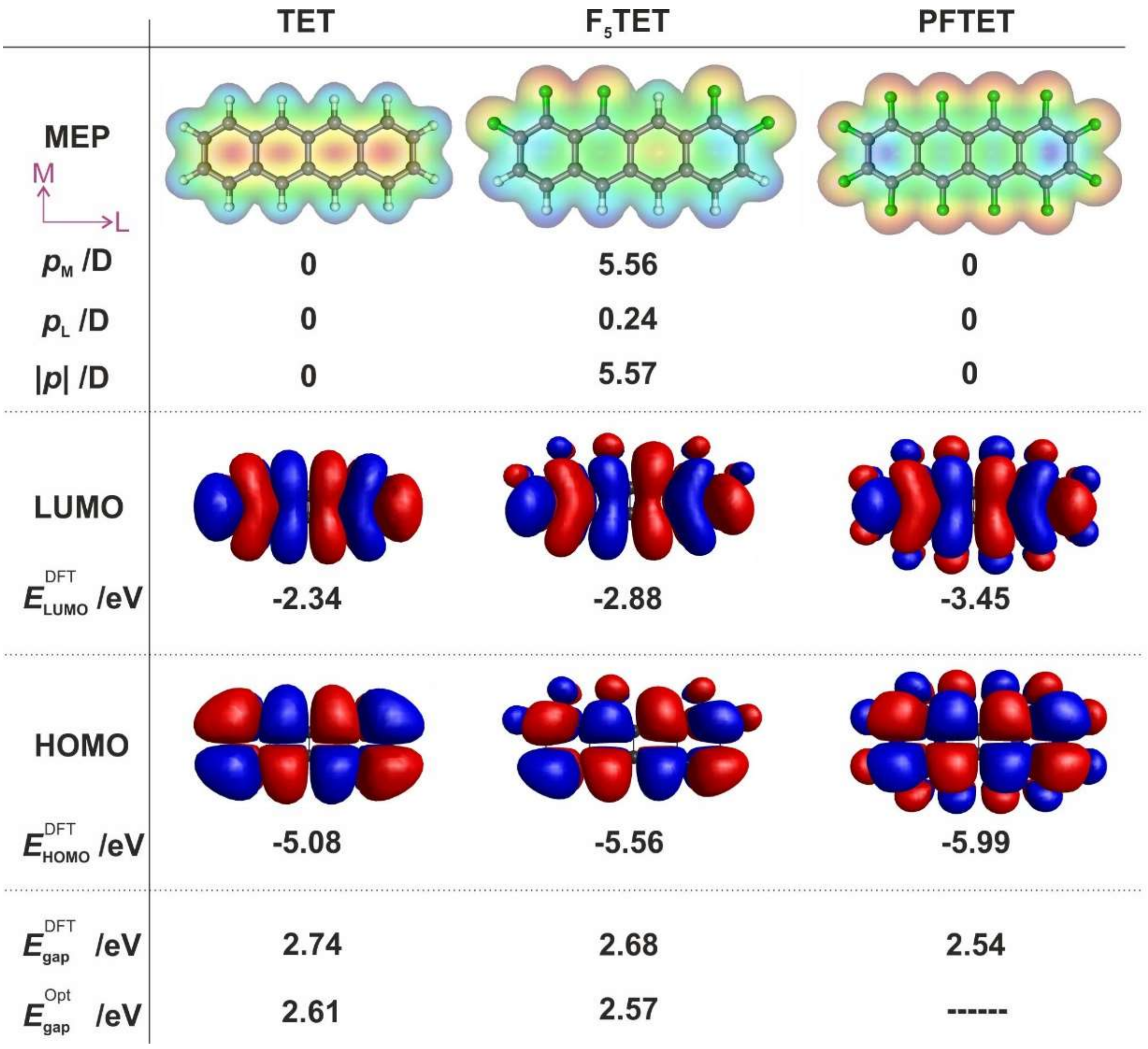

Figure S6. Comparison of electronic properties of tetracene derivatives.

The polarizabilities and quadrupole moments of the presented acenes were calculated at the 6-311G(d,p)+B3LYP level as implemented in the US-GAMESS code[21-22] to enable comparability with the values reported in literature.[25] The corresponding values are shown in figure S7, examining that while the polarizability essentially is not modified by partial fluorination, the quadrupole moments are significantly changed. In particular along the axis perpendicular to the molecular plane (N-axis) the quadrupole moments almost vanish, which is expected to reduce intermolecular electrostatic interactions.

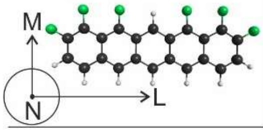

		Polarizabilities				Quadrupole Moments			
		α_L	α_M	α_N	α_{tot}	θ_L	θ_M	θ_N	θ_{tot}
Pentacenes	PEN	90.7	36.2	12.9	46.6	4.9	4.0	-8.9	8.9
	F_6PEN	91.0	36.6	12.9	46.9	-3.2	4.3	-1.1	4.5
	PFP	94.8	38.1	13.0	48.7	-3.7	-5.5	9.2	9.2
Tetracenes	TET	63.8	29.5	10.7	34.7	3.3	4.0	-7.3	7.3
	F_5TET	64.1	29.8	10.8	34.9	-3.4	4.0	-0.6	4.3
	PFTET	66.3	31.0	10.8	36.0	-3.6	-5.4	9.0	9.1

Figure S7. Comparison of polarizabilities (given in 10^{-24}cm^3) and quadrupole moments (given in 10^{-34}Ccm^2) of selected acenes. The magnitude of quadrupole moments Θ_{tot} as well as the static polarizability α_{tot} are calculated as shown in literature.[25]

The intramolecular distances and angles of the presented acenes are derived from geometry optimizations at the aug-cc-pVTZ+B3LYP level as implemented in the US GAMESS-code.[21-22]

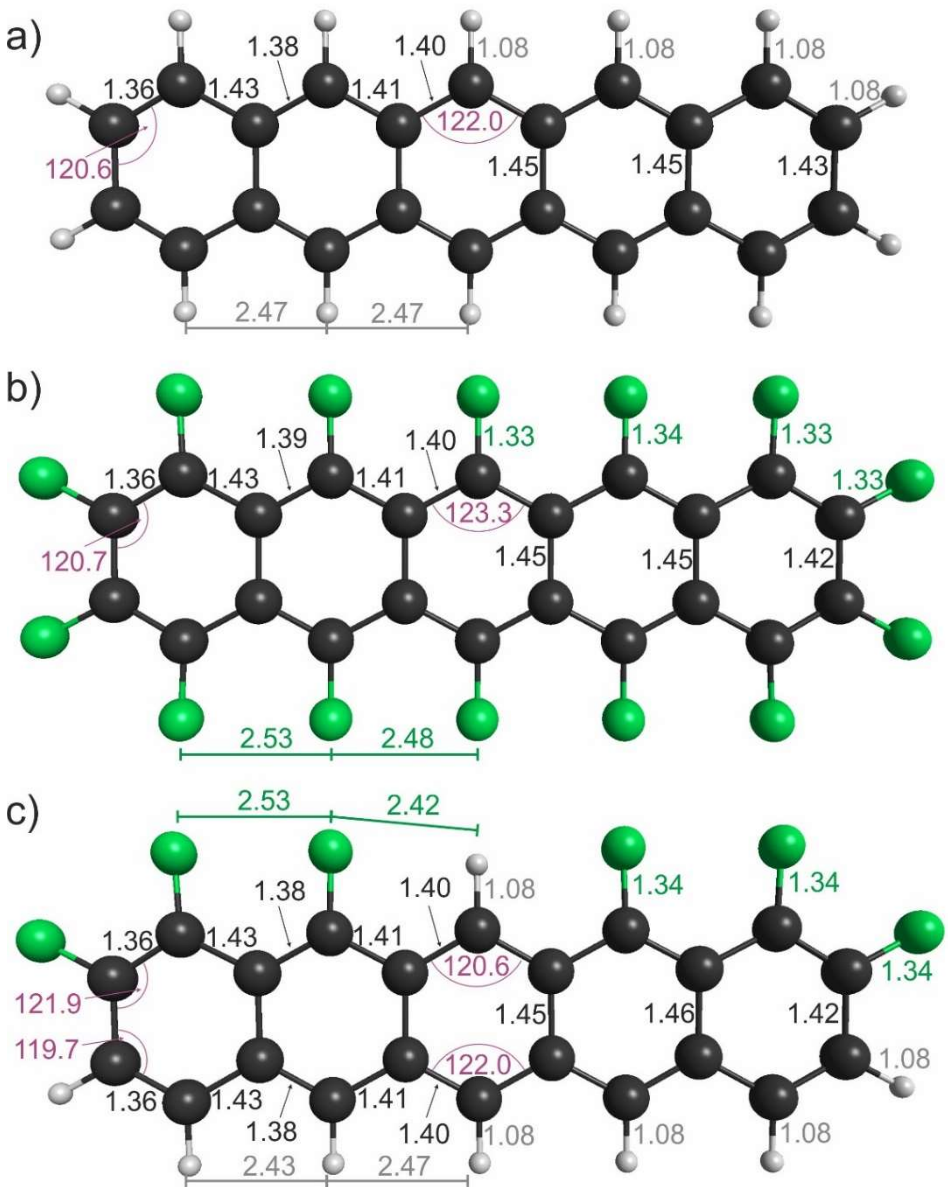

Figure S7. Comparison of intramolecular distances (given in Å) and angles (given in °) of a) PEN, b) PFP and c) F_6PEN (**1**), examining the presence of slight structural modifications due to fluorination. The calculated bond lengths and angles are in close agreement to the values observed in the crystal structures.[26-27]

10. Hirshfeld Analysis

Hirshfeld surface analyses were performed using the CrystalExplorer software package.[28-29] For every point on the Hirshfeld surface the distance to the nearest atom interior and exterior is determined and compared with the expected van der Waals distances for the respective combinations of contacted elements (e.g. hydrogen and fluorine). A coloration of the Hirshfeld surface is then achieved by this means, where red color corresponds to a smaller and blue color corresponds to a larger distance than expected from the vdW distances. Hence red points identify the positions, where intermolecular bindings are created.

The evaluation of the so-called Hirshfeld fingerprint plots allows further statements, which provide an objective and quantitative measure for the packing in the crystal. For this purpose for each point of the Hirshfeld surface the distances to the nearest atom interior (d_i) and exterior (d_e) or the Hirshfeld volume are plotted against each other. Since distances can appear many times, these distances are binned into discrete intervals (of width 0.01 Å) and a coloration indicates the frequency of occurrences ranging from blue (relatively few points) through green to red (many points).

In figure S8 the element specific fingerprint plots of F_6PEN are shown, whereas the F-H contact points provide more than 45% of all determined contacts. Hence these interactions strongly govern intermolecular binding.

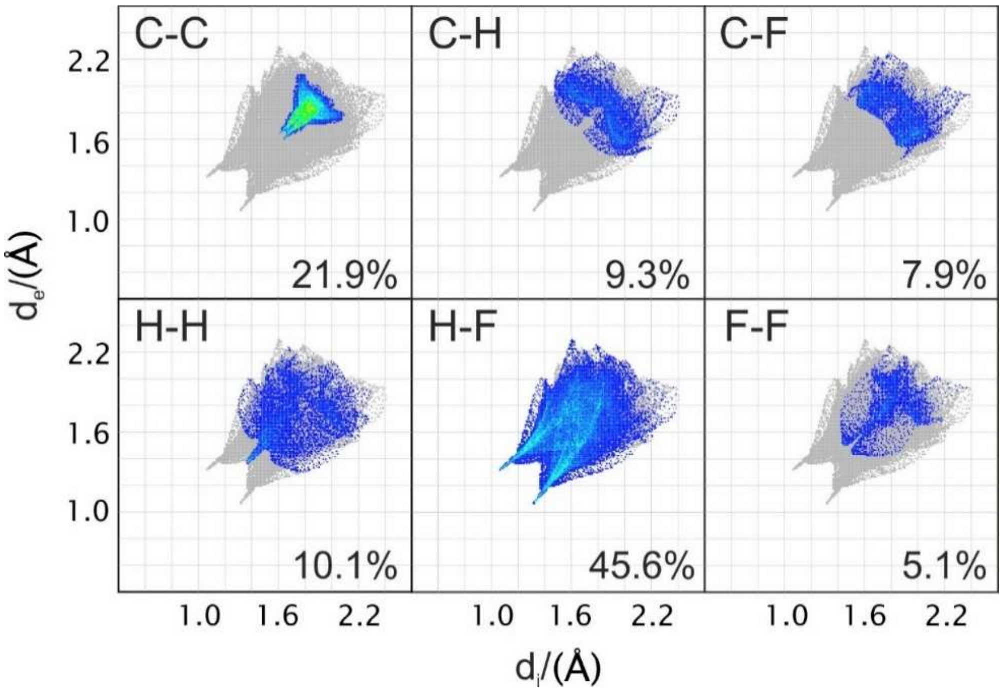

Figure S8. Element specific fingerprint plots for F_6PEN. All possible atom combinations are considered. Fingerprint plots of heteroatomic combinations always include also the mirrored case, e.g. C...H = C…H+H…C.

Figure S9 shows a comparison of the packing motifs and crystal structures of TET, PEN, F_5TET, F_6PEN, PFP bulk and PFP π-stacked phase, indicating that the novel molecules 1 and 2 both exhibit a criss-cross packing, while for TET, PEN and PFP herringbone packing is observed in bulk. When comparing the Hirshfeld surfaces of these molecules, it is noticeable that there are significantly fewer contact points for the non-fluorinated acenes than for the partially fluorinated acenes. Consequently the criss-cross packing motif of the partially fluorinated acenes is stabilized by intermolecular interactions at the molecular rims. Comparing the 2D-fingerprint plots yields strong similarities between tetracene and pentacene, as well as between their unilaterally fluorinated conterparts. These however are significantly different than the fingerprint plots of perfluoropentacene in various polymorphs. Hence the crystalline packing in unilaterally fluorinated acenes is strongly governed by interactions (in particular fluorine-hydrogen interactions), which do not appear in case of un- or perfluorinated acenes.

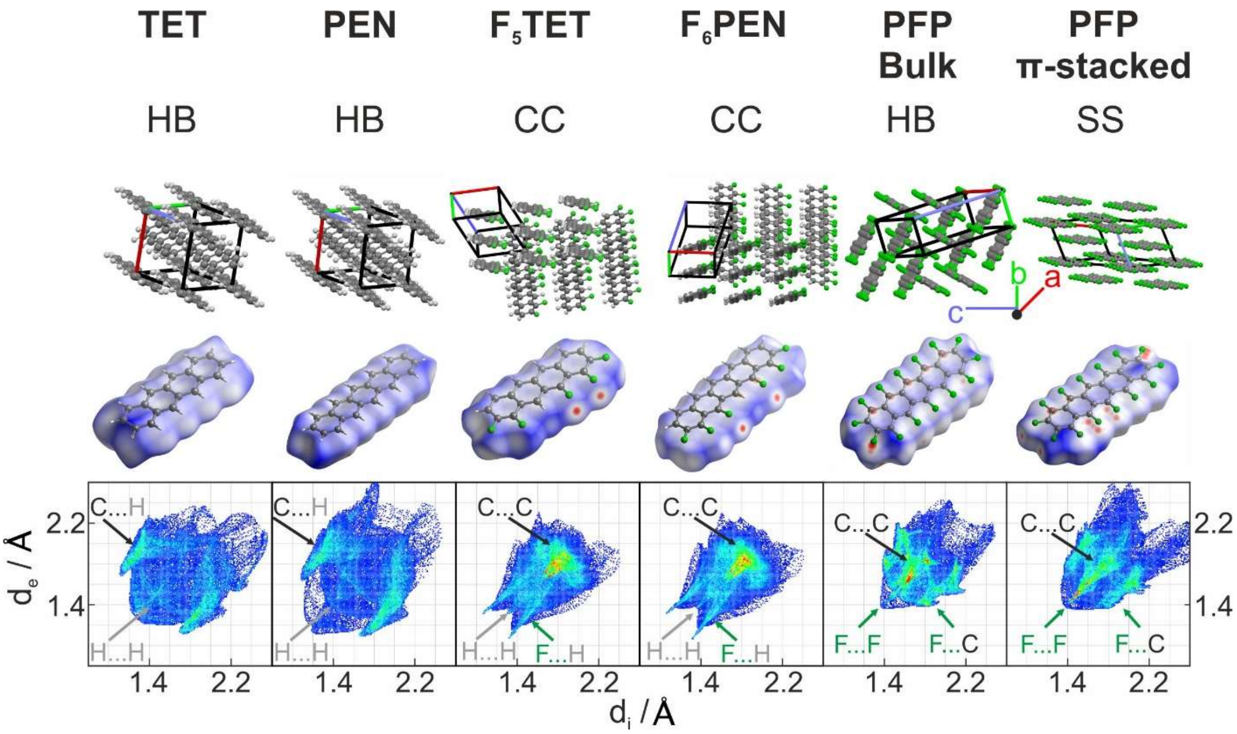

Figure S9. Comparison of packing motifs (Herringbone - HB, Criss-Cross - CC and Slip Stacked - SS)[25], crystal structures[26-27,30] Hirshfeld surfaces and 2D-fingerprint plots of different tetracenes and pentacenes.

11. NMR Spectra of All Compounds

1. 1,7,8-trifluoronaphthalen-2-yl trifluoromethanesulfonate

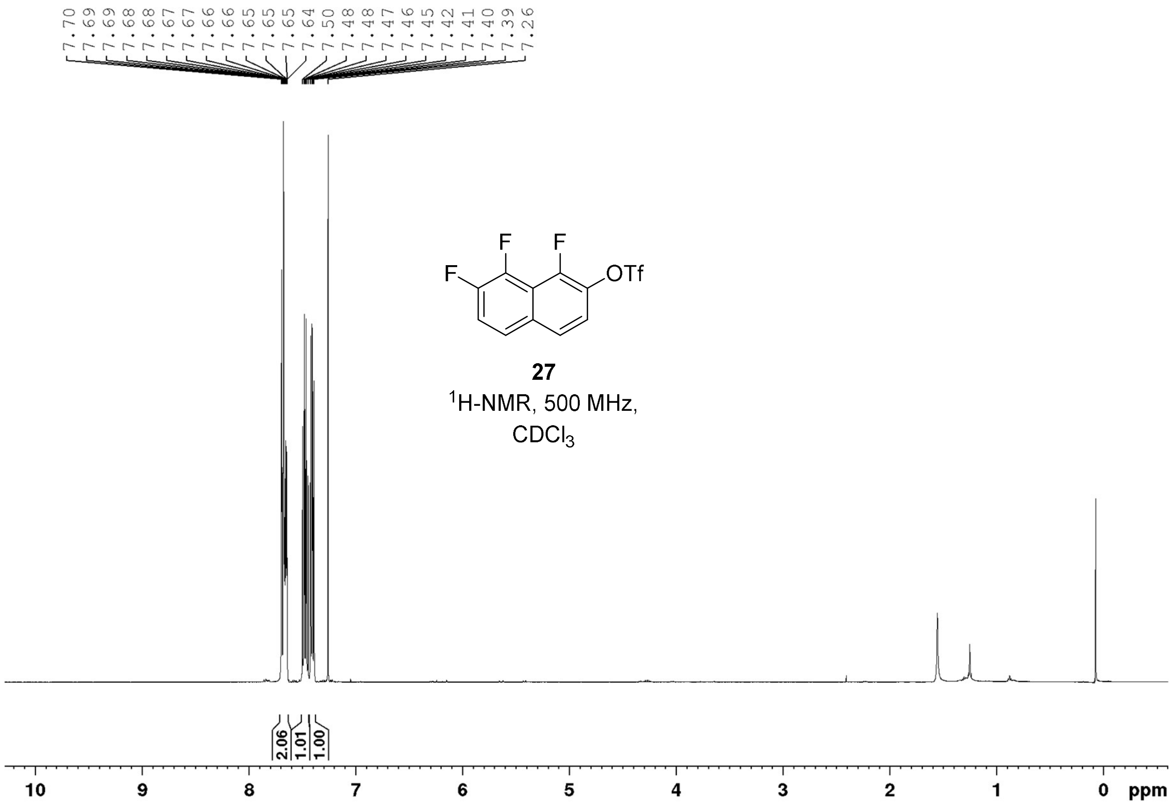

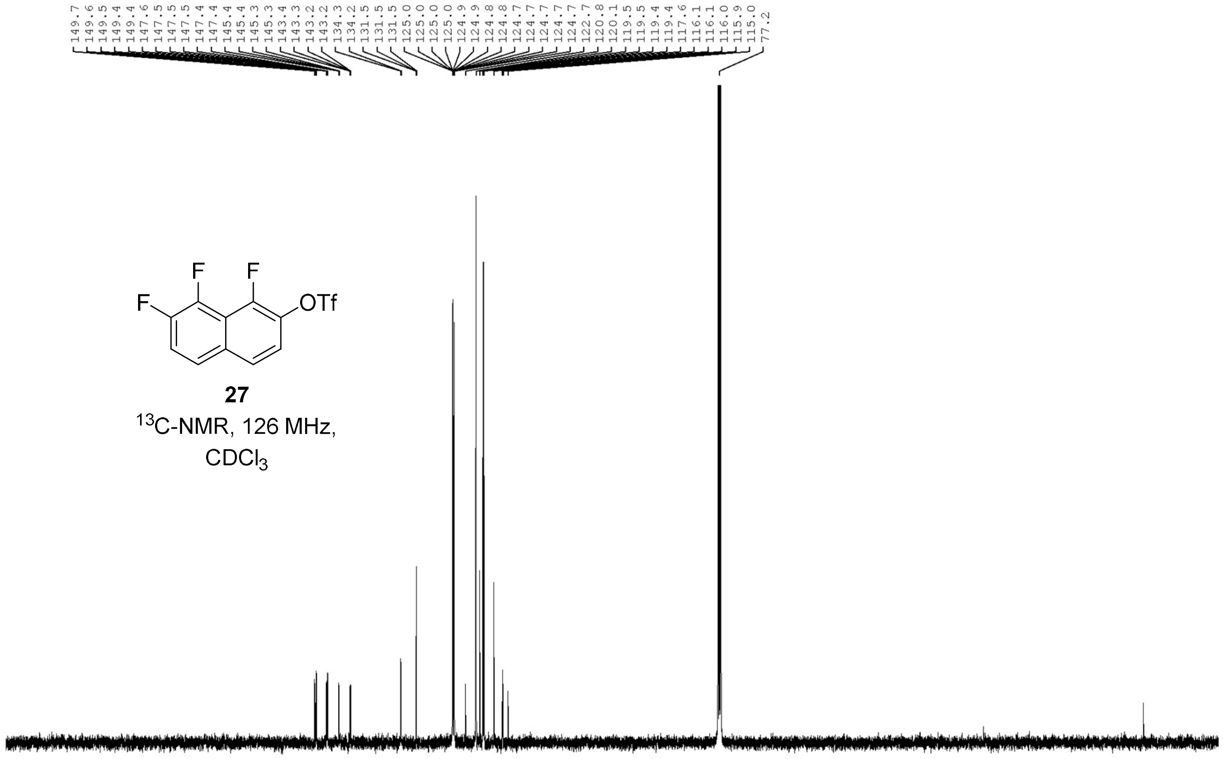

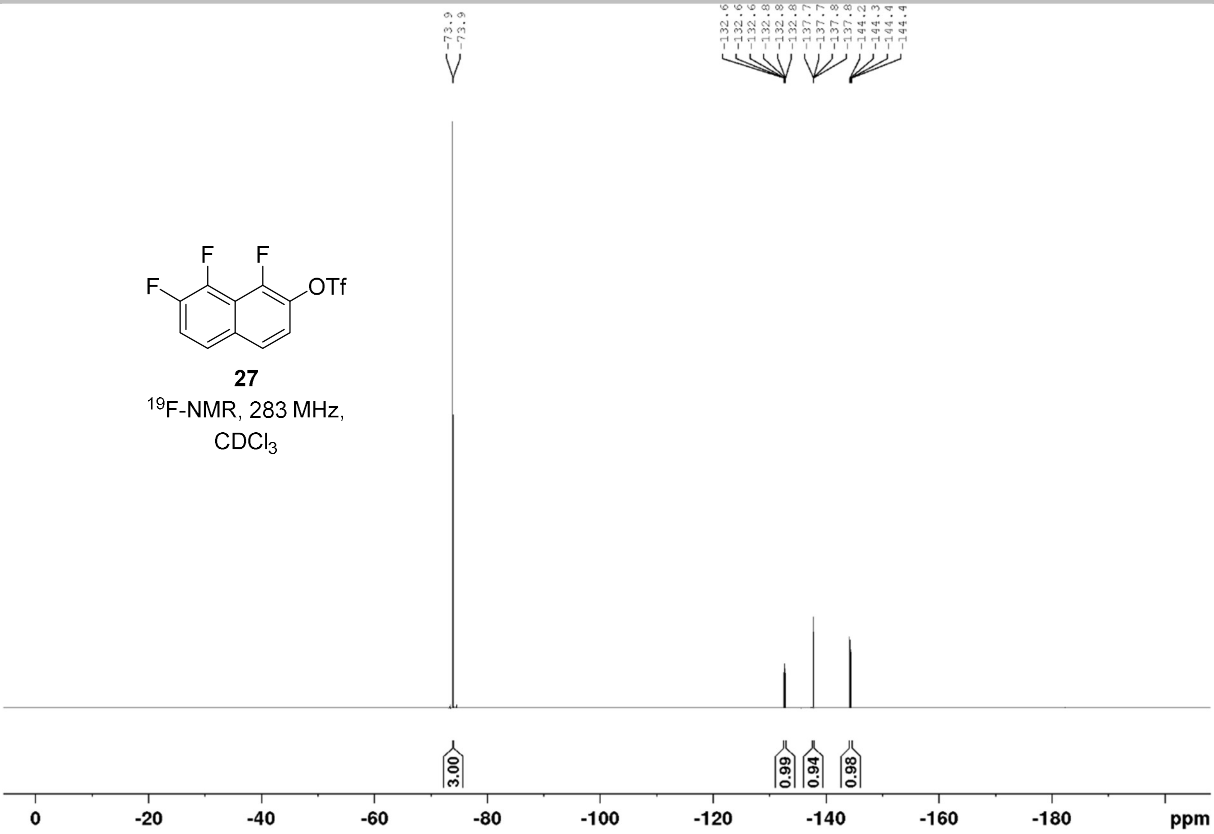

2. methyl 1,7,8-trifluoro-2-naphthoate

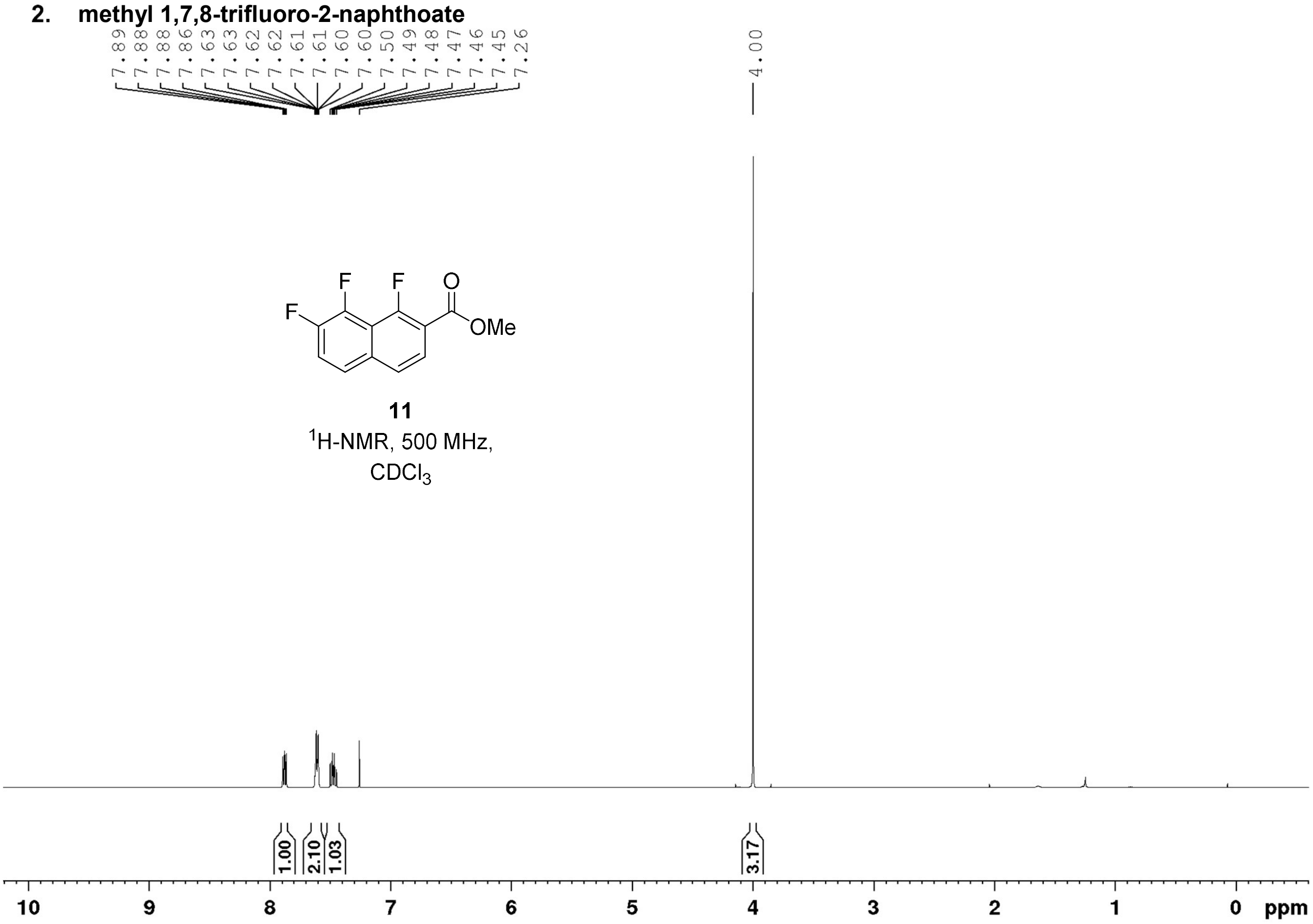

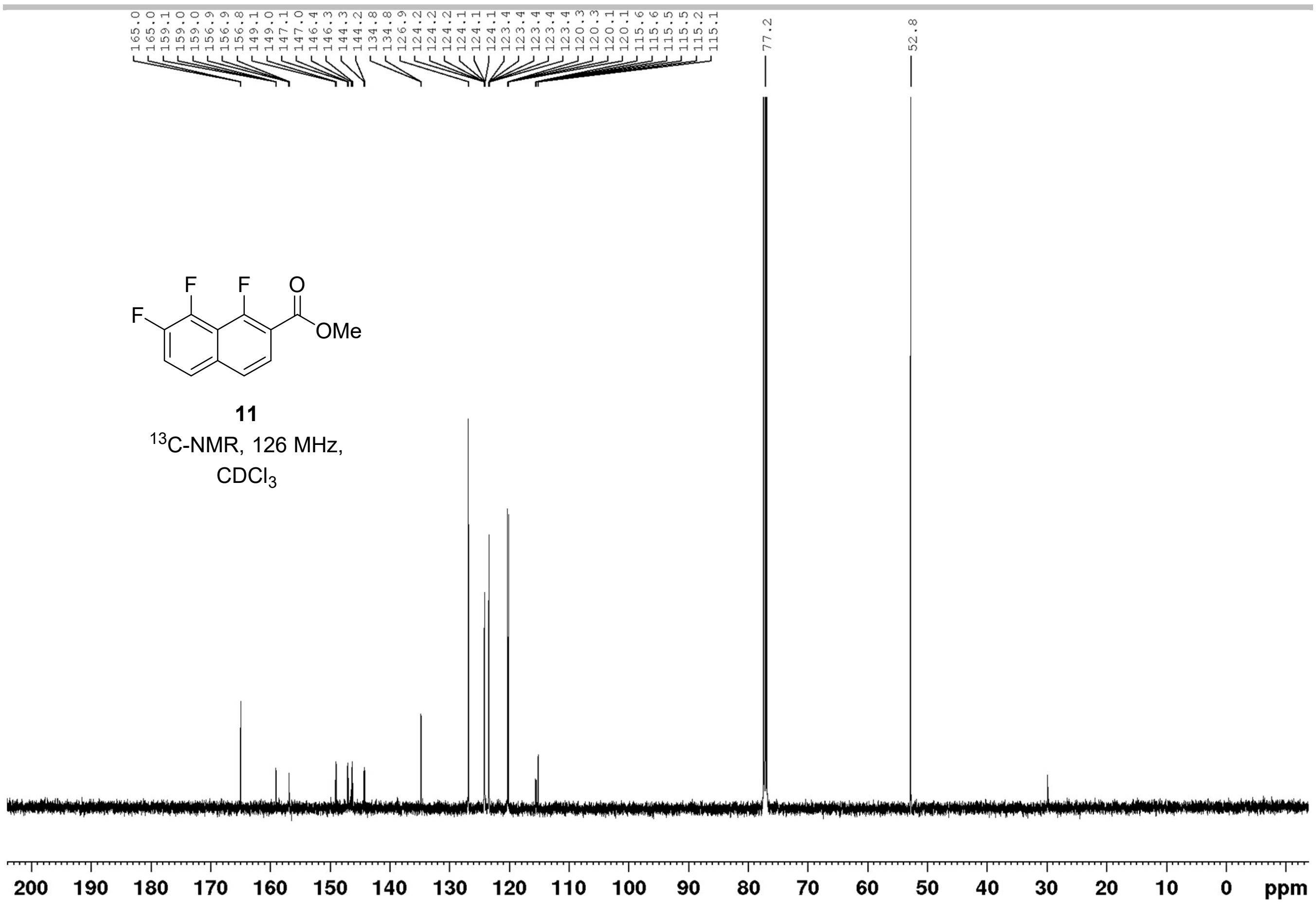
165.0
165.0
159.1
159.0
159.0
156.9
156.9
156.8
149.1
149.0
147.1
147.0
146.4
146.3
144.3
144.2
134.8
134.8
126.9
124.2
124.2
124.2
124.1
124.1
124.1
123.4
123.4
123.4
123.4
120.3
120.3
120.1
120.1
115.6
115.5
115.5
115.2
115.1
77.2
52.8
F
F
F
O
OMe
11
13C-NMR, 126 MHz,
CDCl3
200
190
180
170
160
150
140
130
120
110
100
90
80
70
60
50
40
30
20
10
0
ppm

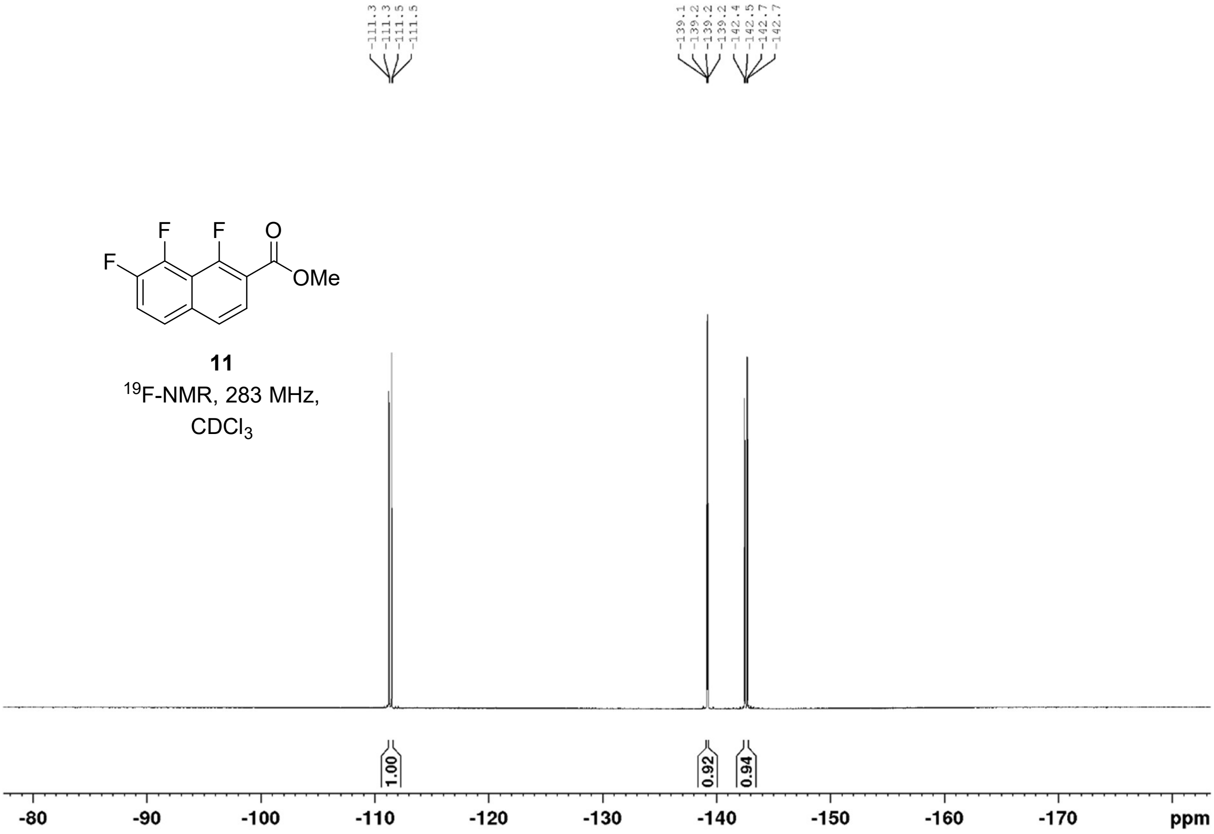
-111.3
-111.3
-111.5
-111.5
-139.1
-139.2
-139.2
-139.2
-142.4
-142.5
-142.7
-142.7
F
F
F
O
OMe
11
19F-NMR, 283 MHz,
CDCl3
1.00
0.92
0.94
-80
-90
-100
-110
-120
-130
-140
-150
-160
-170
ppm

3. **methyl 1,7,8-trifluoro-3-(4,4,5,5-tetramethyl-1,3,2-dioxaborolan-2-yl)-2-naphthoate**

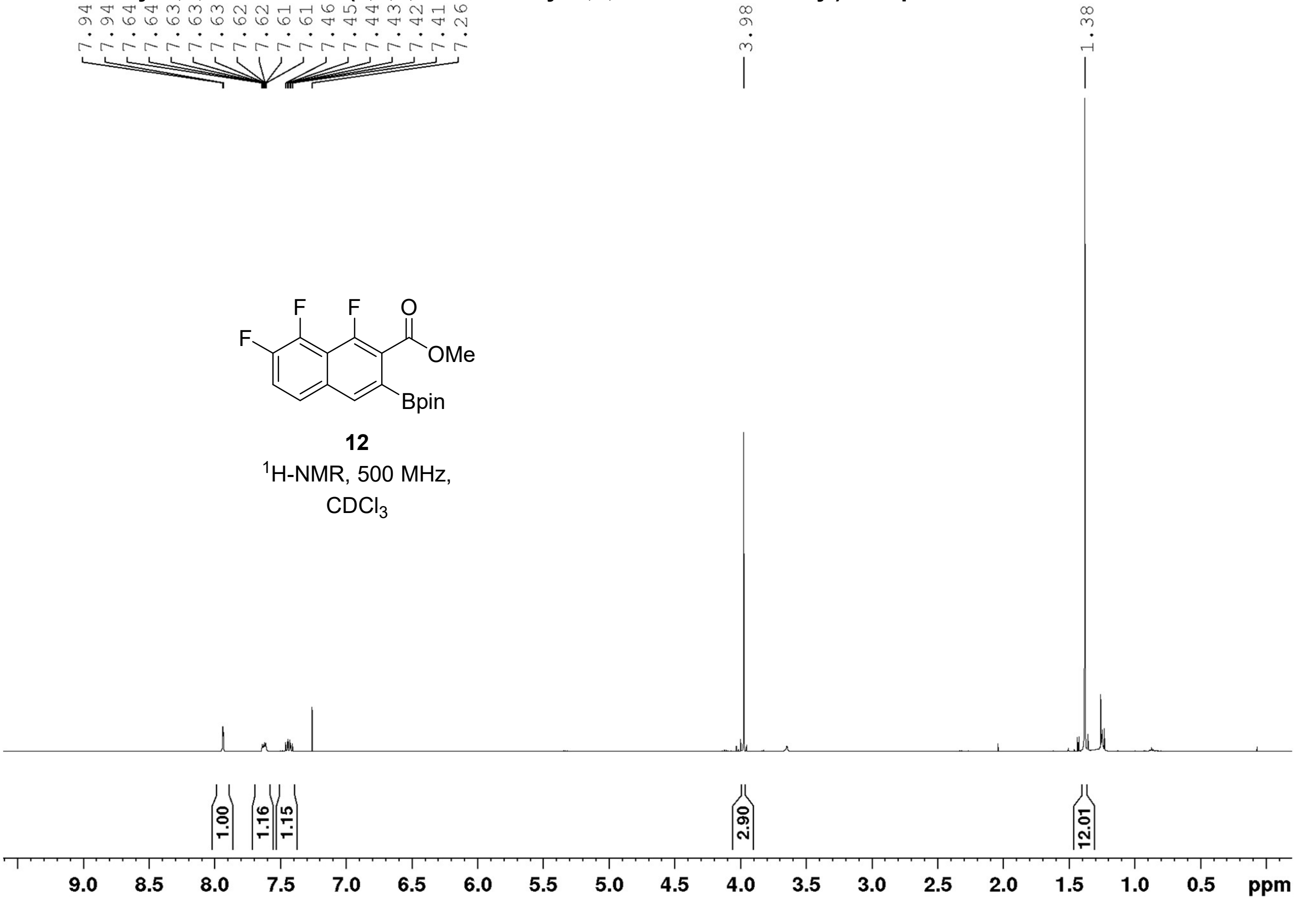

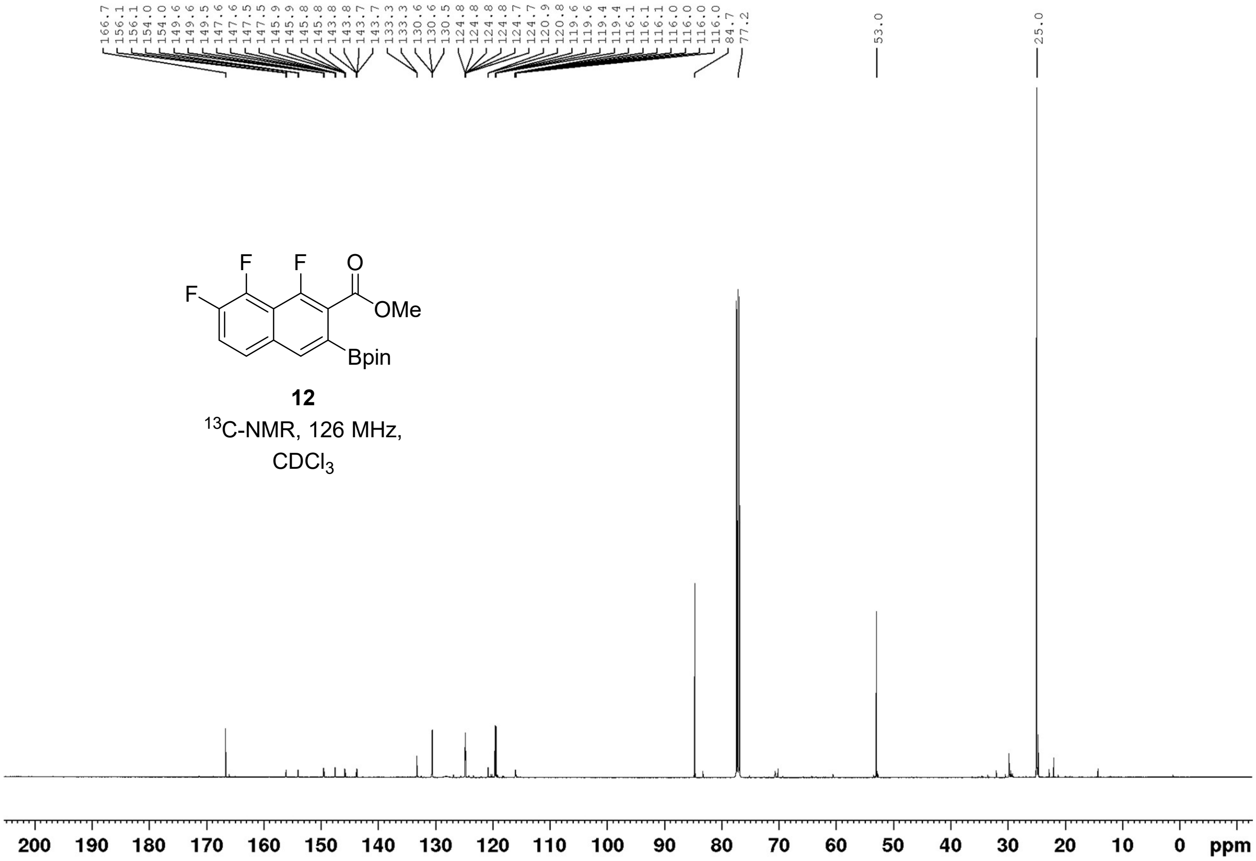

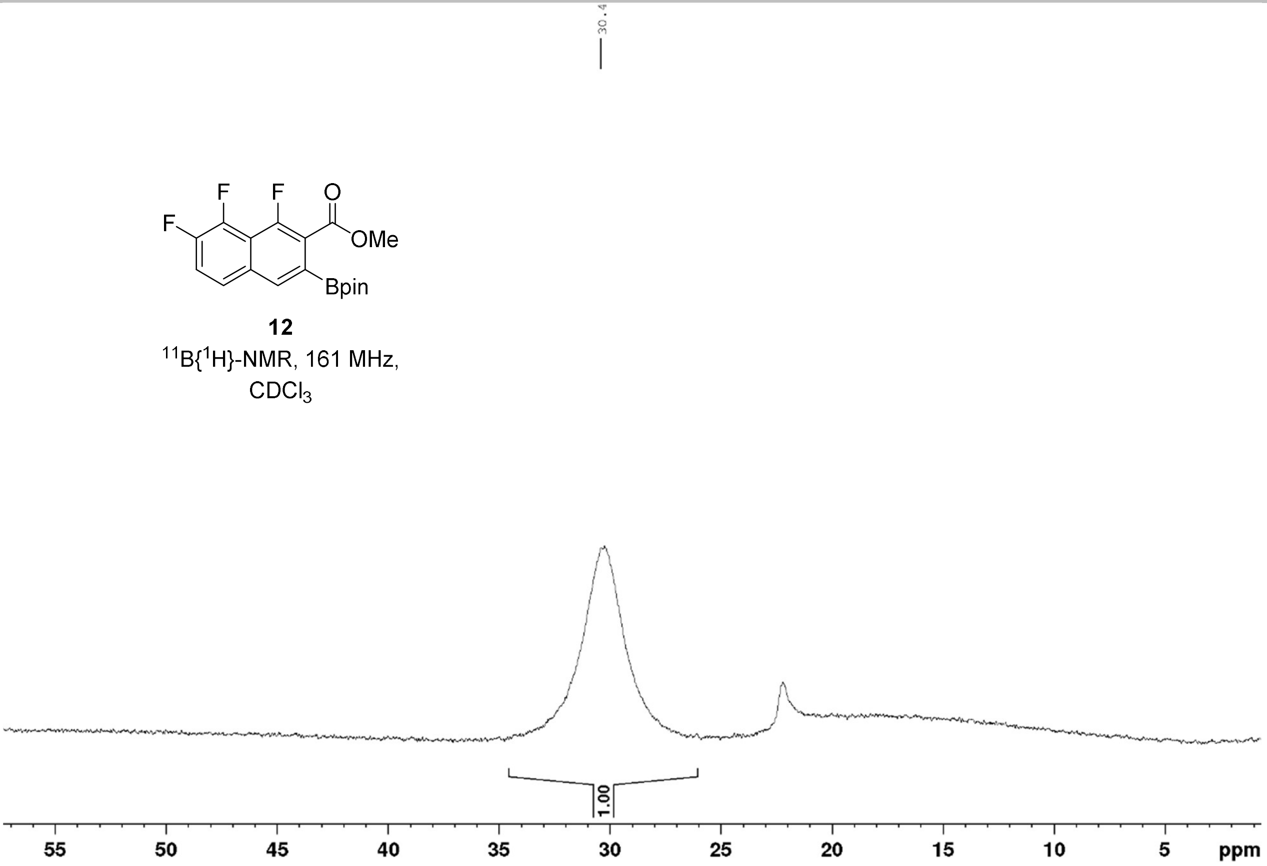
30.4
F
F
O
F
OMe
Bpin
12
$^{11}B\{^{1}H\}$-NMR, 161 MHz, $CDCl_3$
1.00
55
50
45
40
35
30
25
20
15
10
5
ppm

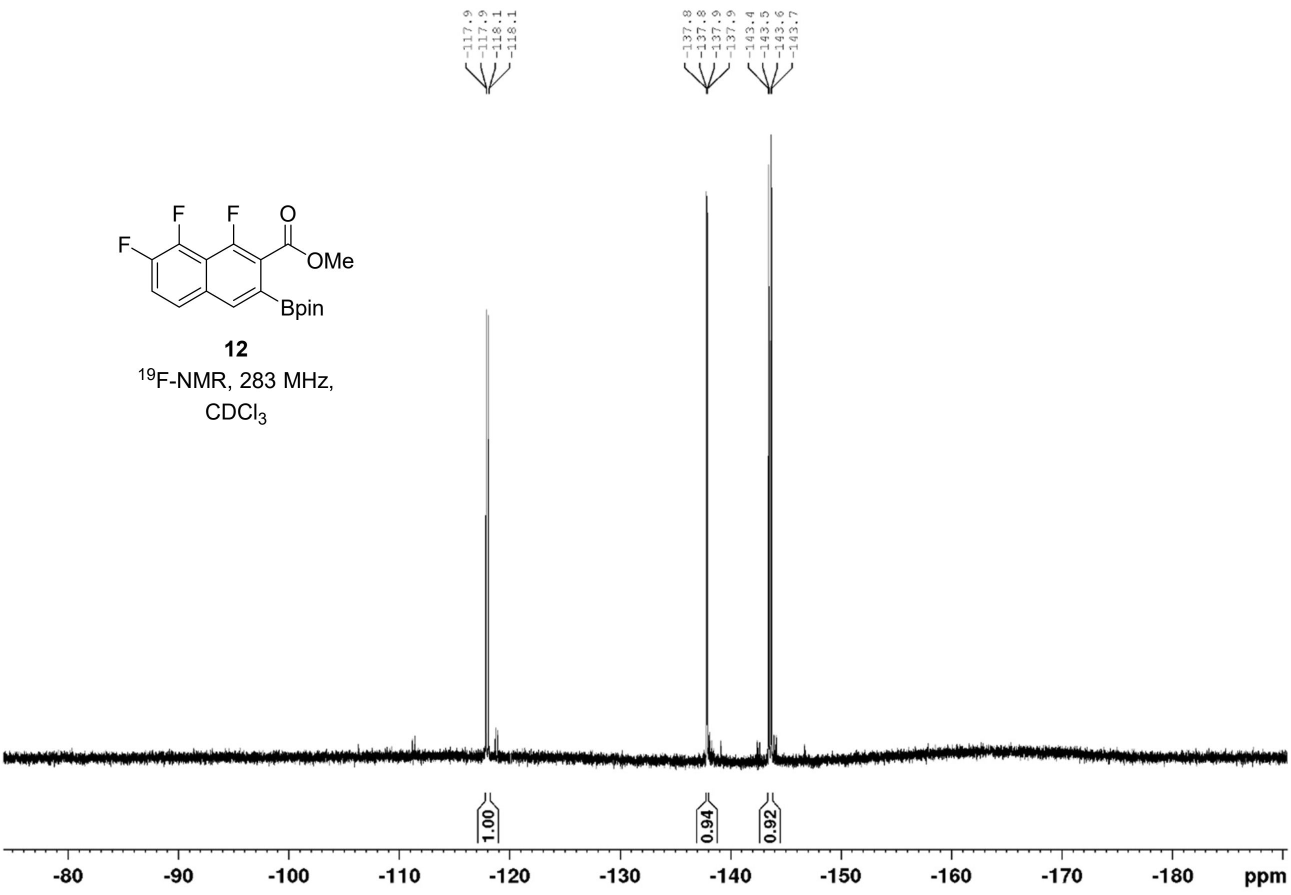
-117.9
-117.9
-118.1
-118.1
-137.8
-137.8
-137.9
-137.9
-143.4
-143.5
-143.6
-143.7
F
F
O
F
OMe
Bpin
12
^{19}F-NMR, 283 MHz, $CDCl_3$
1.00
0.94
0.92
-80
-90
-100
-110
-120
-130
-140
-150
-160
-170
-180
ppm

4. **(2-(diisopropylsilyl)phenyl)diphenylphosphane**

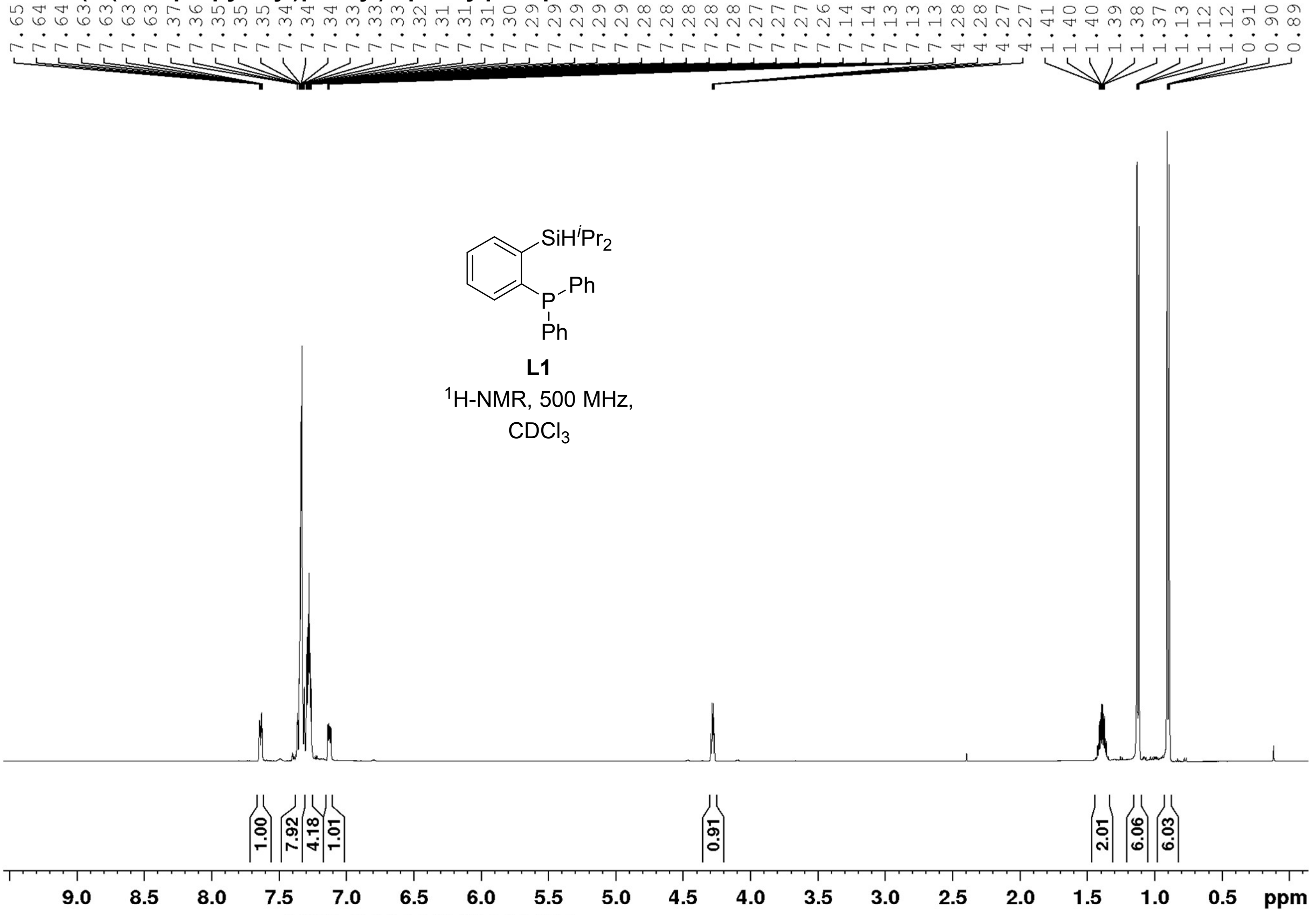

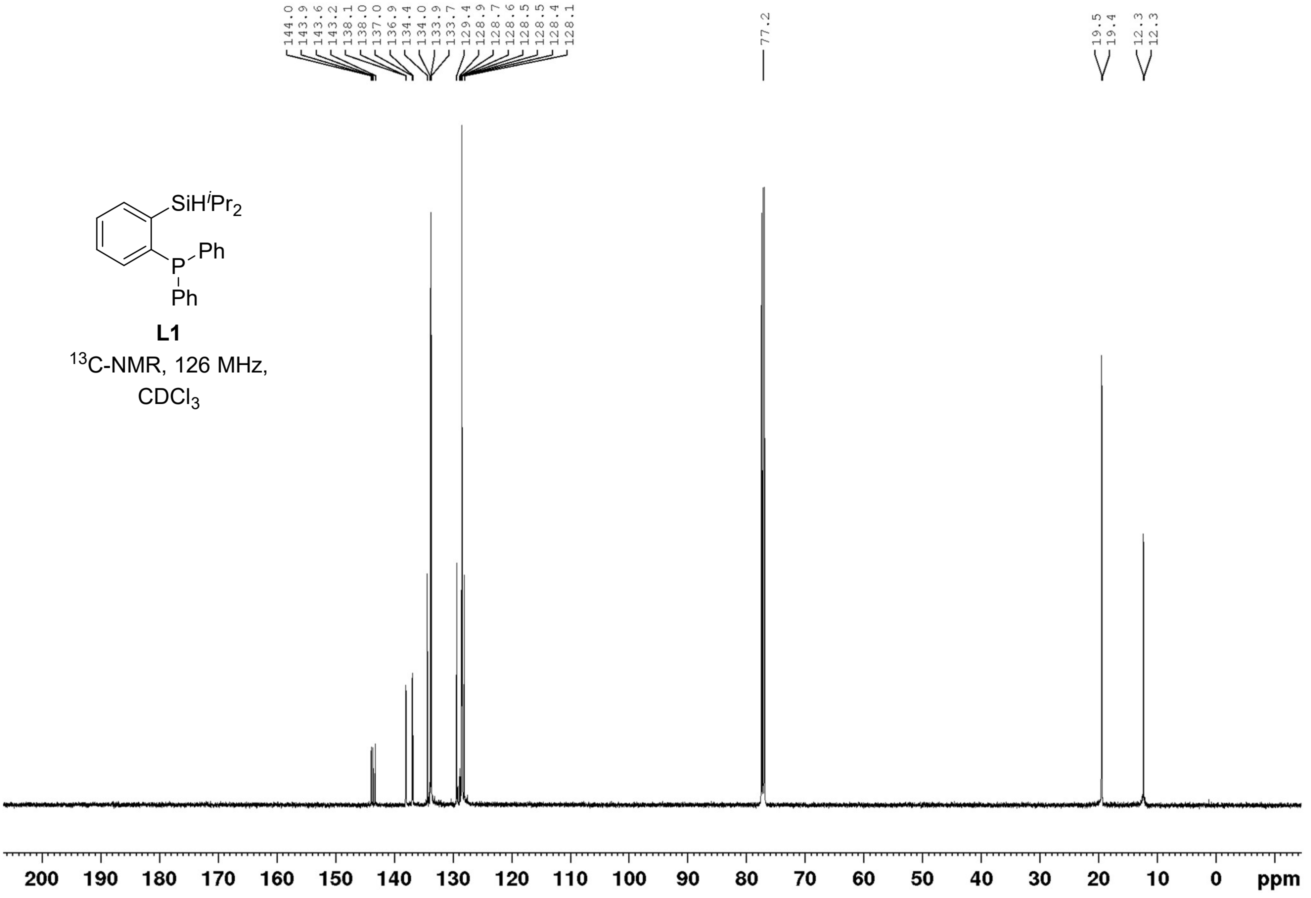

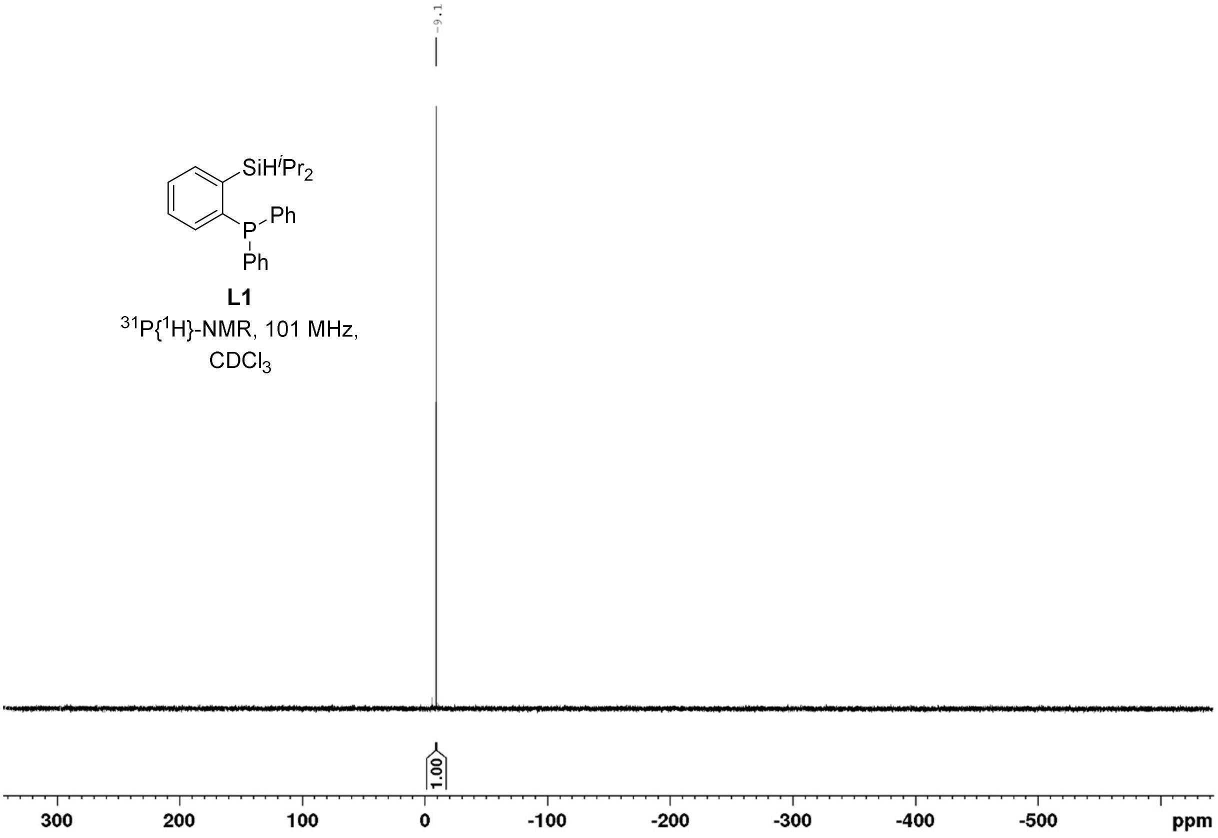

L1
$^{31}P\{^1H\}$-NMR, 101 MHz, $CDCl_3$

5. 1,2,8-trifluoro-7-(methoxymethoxy)naphthalene

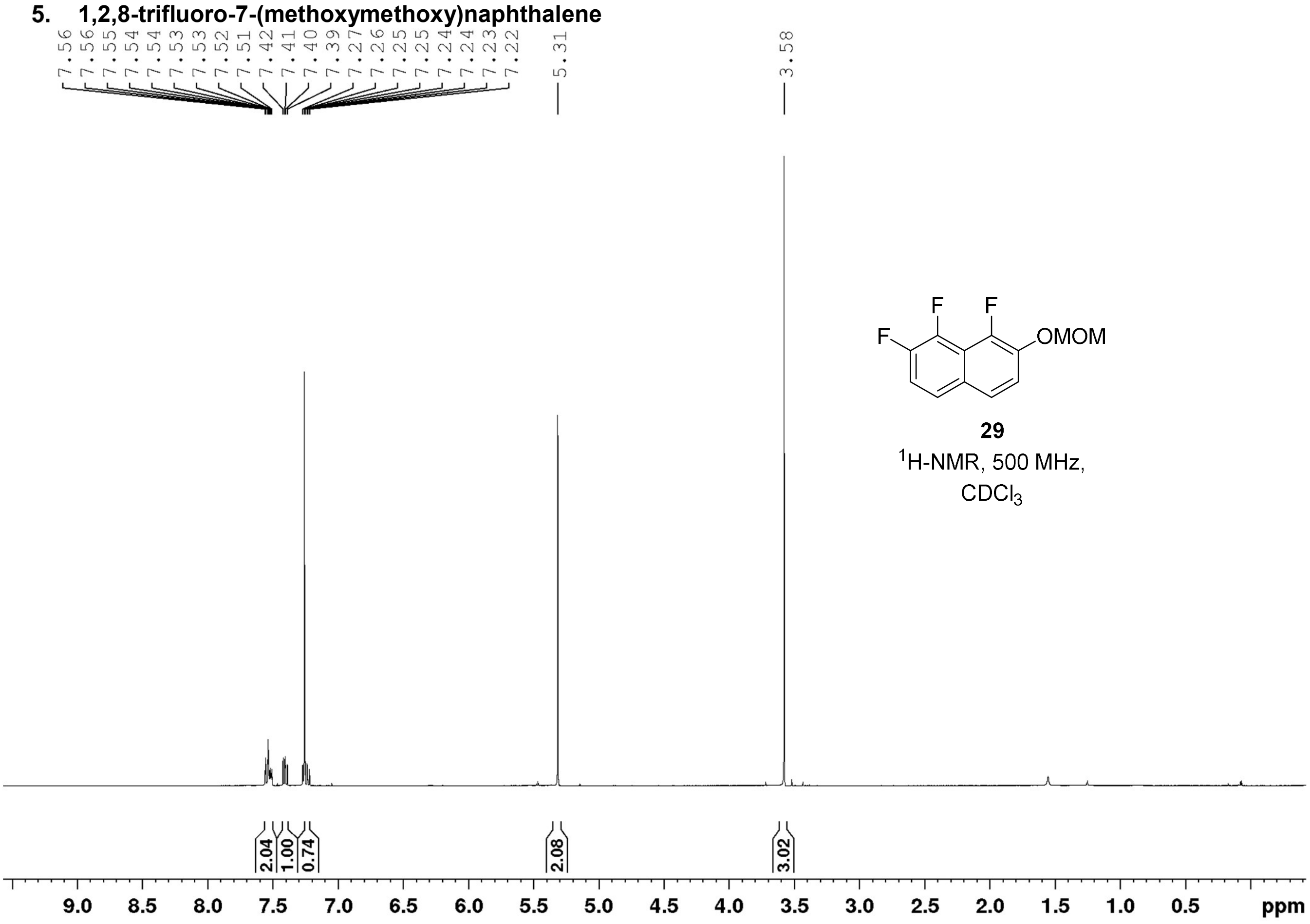

29
^1H-NMR, 500 MHz, $CDCl_3$

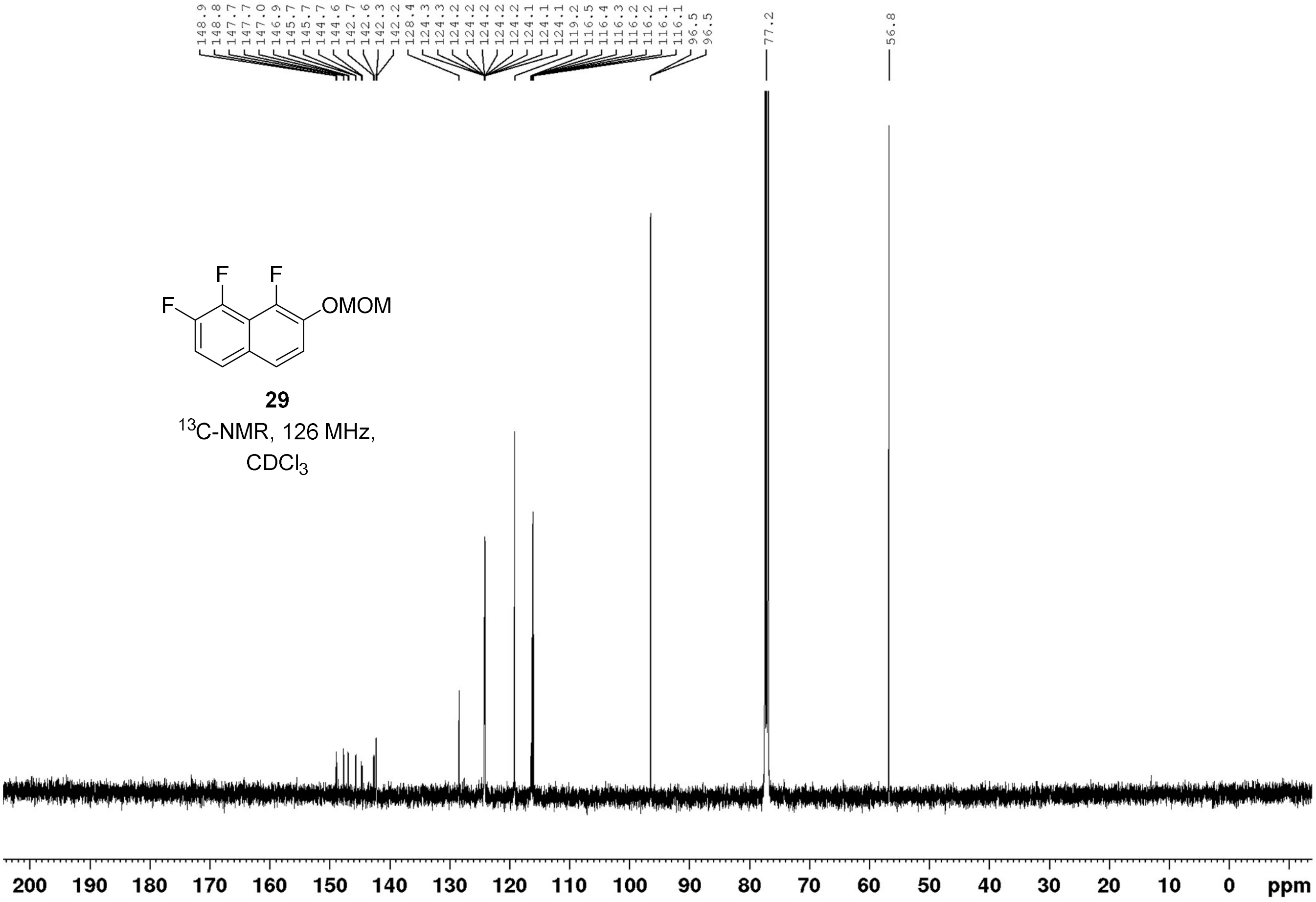
F
F
F
OMOM
29
13C-NMR, 126 MHz,
CDCl3
200
190
180
170
160
150
140
130
120
110
100
90
80
70
60
50
40
30
20
10
0
ppm

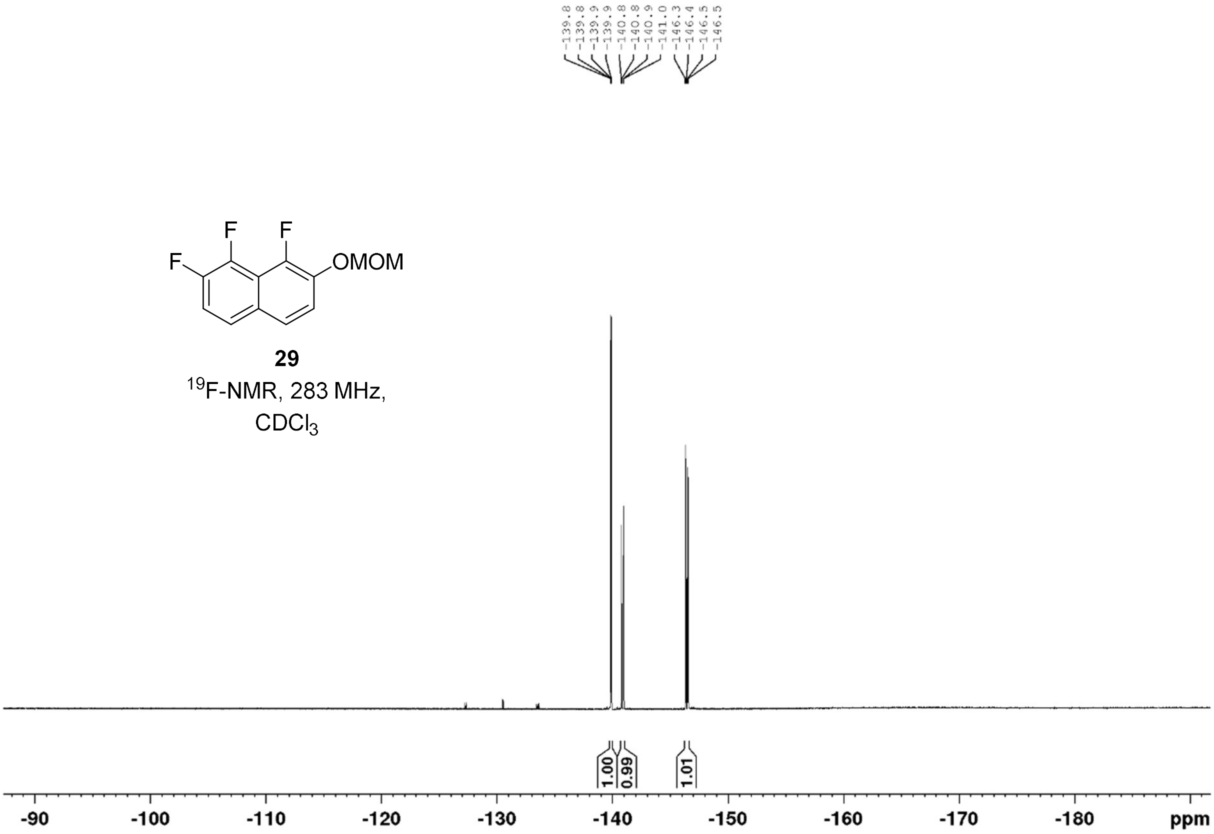
F
F
F
OMOM
29
19F-NMR, 283 MHz,
CDCl3
1.00
0.99
1.01
-90
-100
-110
-120
-130
-140
-150
-160
-170
-180
ppm

6. 4,5,6-trifluoro-3-(methoxymethoxy)-2-naphthaldehyde

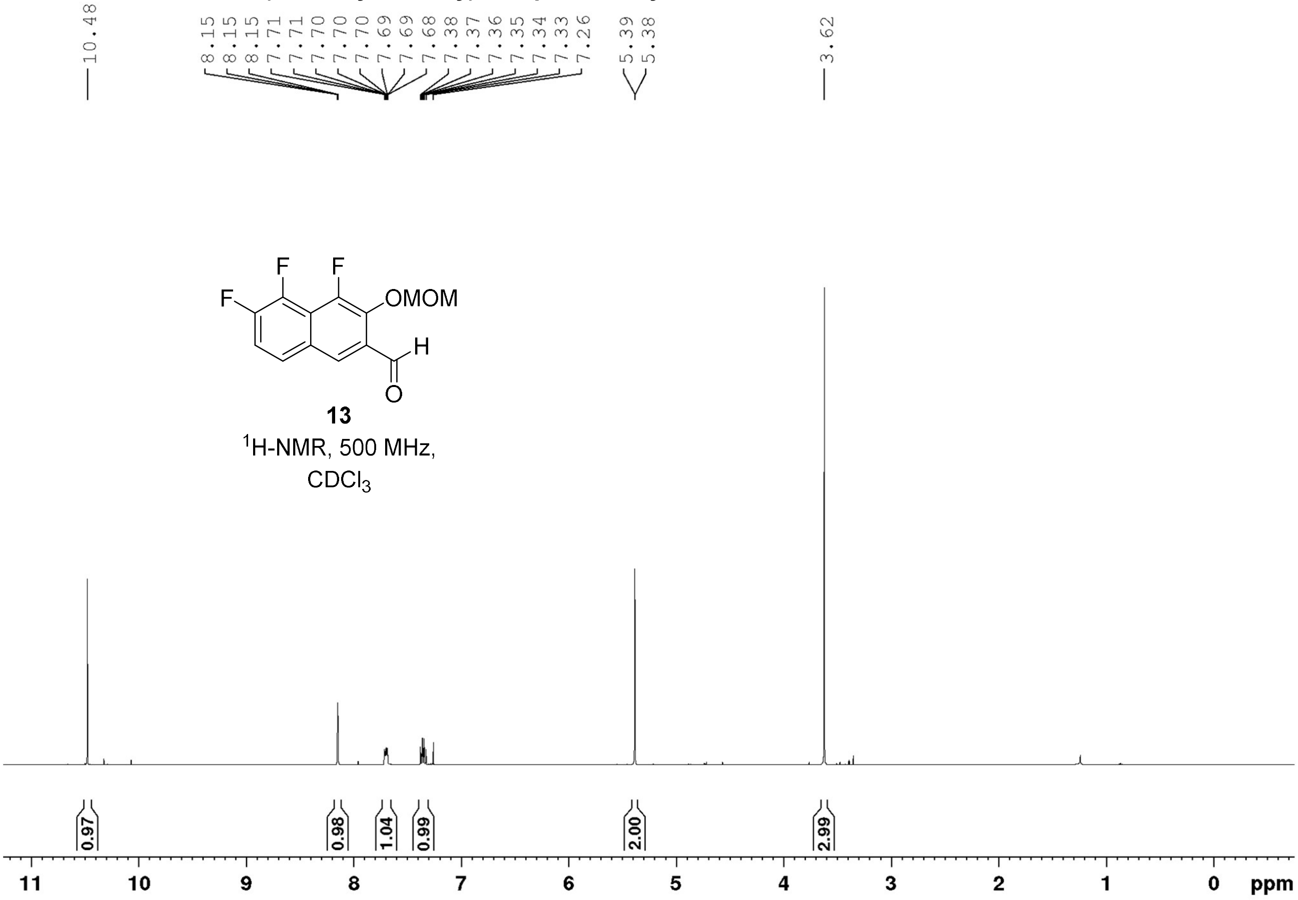

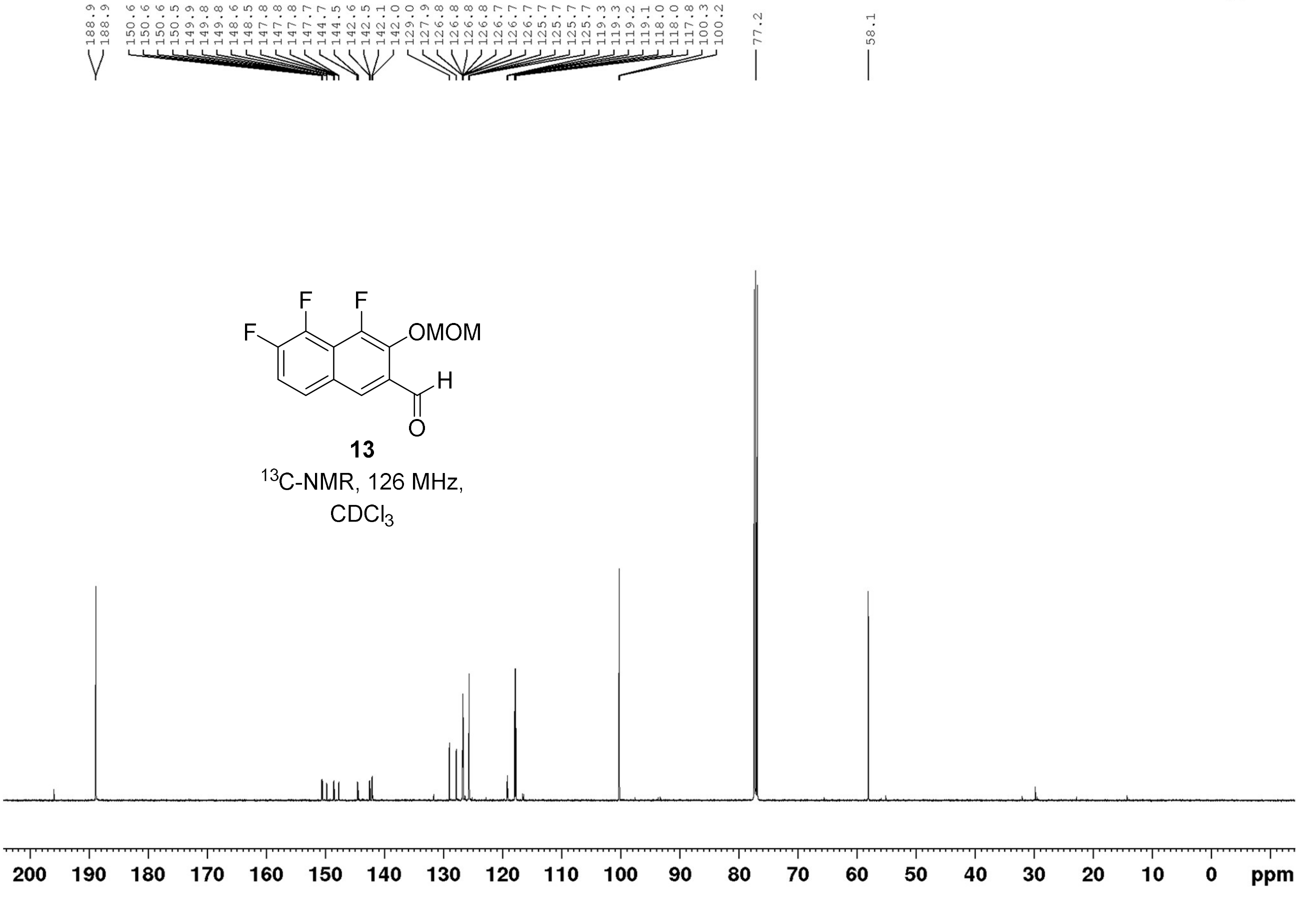

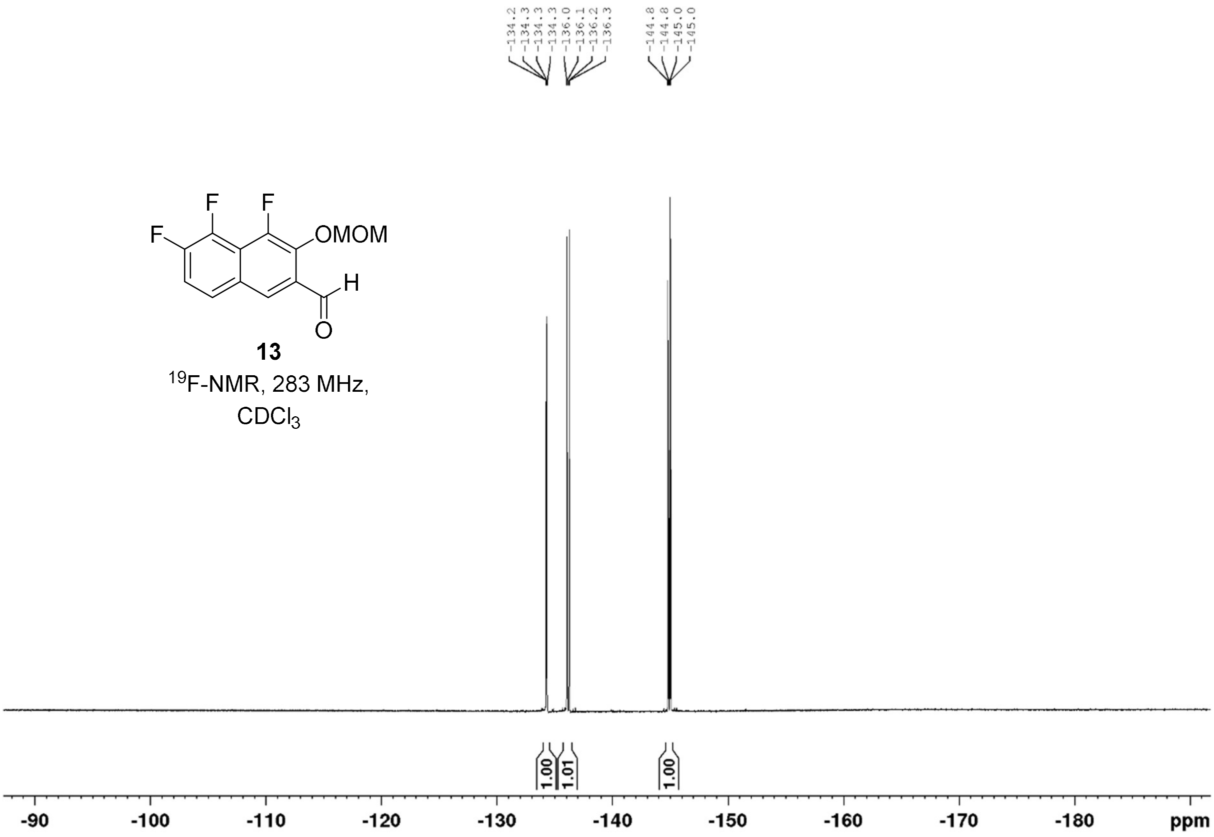

7. (4,5,6-trifluoro-3-(methoxymethoxy)naphthalen-2-yl)methanol

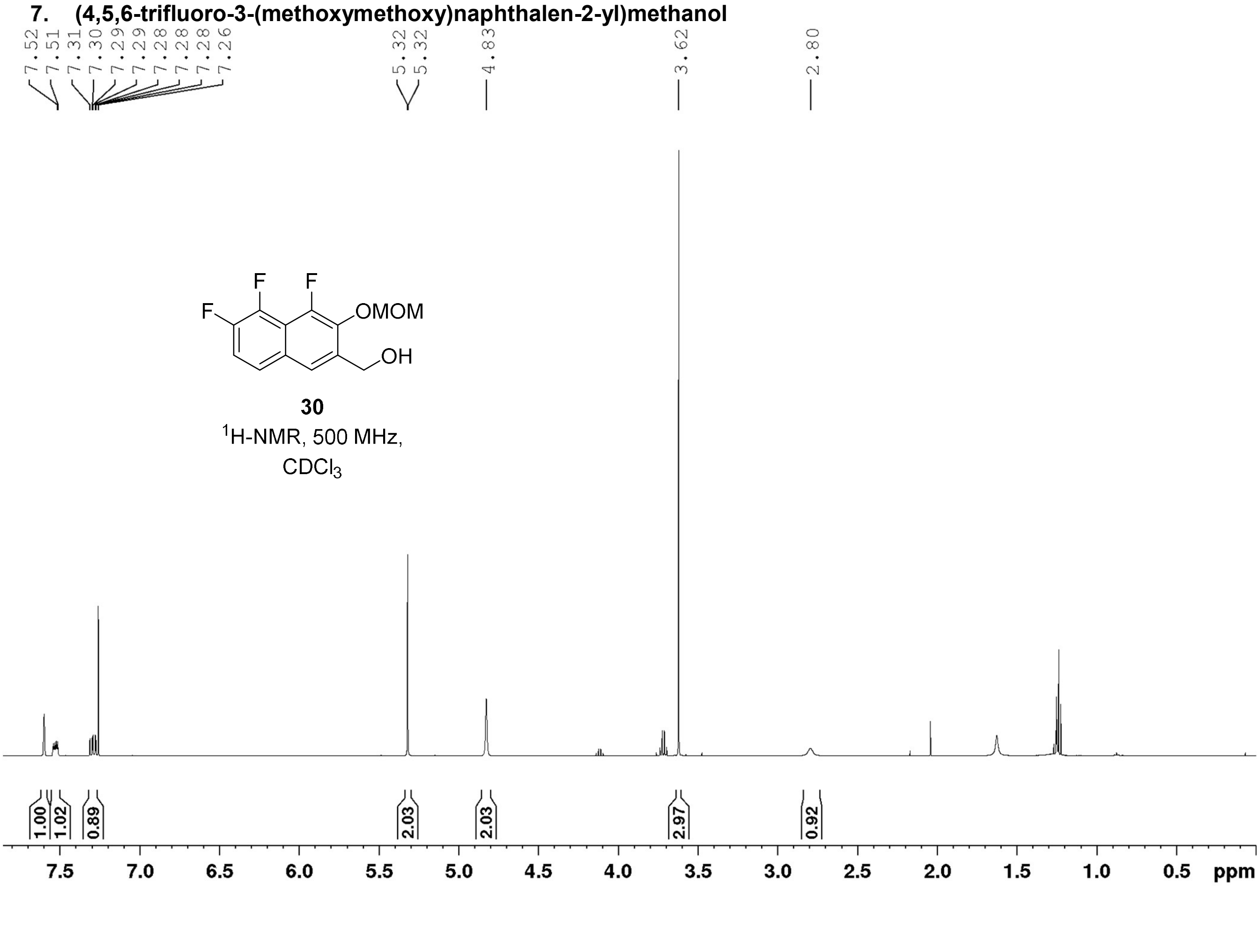

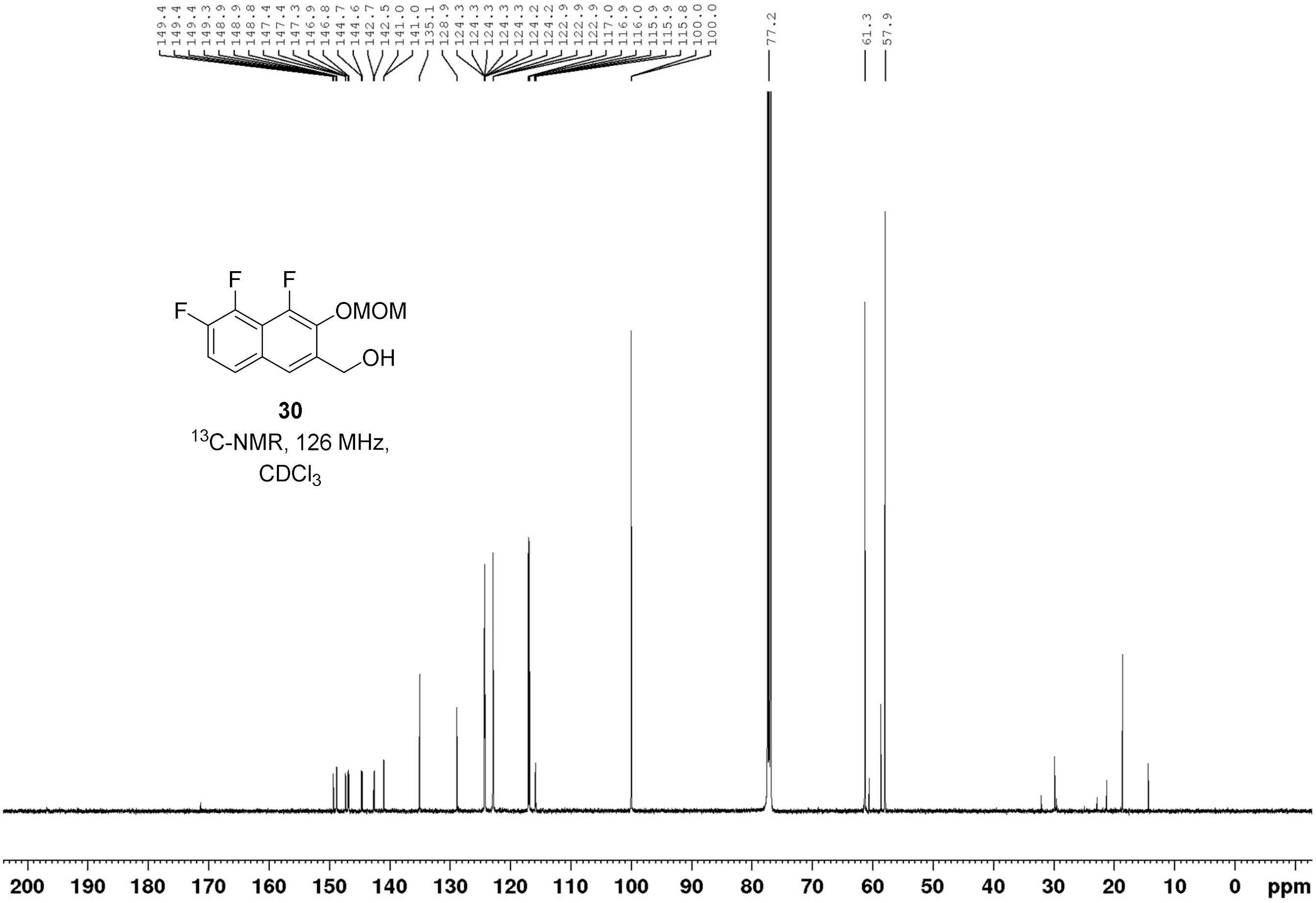
149.4
149.4
149.4
149.3
148.9
148.9
148.8
147.4
147.4
147.3
146.9
146.8
144.7
144.6
142.7
142.5
141.0
141.0
135.1
128.9
124.3
124.3
124.3
124.3
124.3
124.2
124.2
122.9
122.9
122.9
117.0
116.9
116.0
115.9
115.9
115.8
100.0
100.0
77.2
61.3
57.9
F
F
F
OMOM
OH
30
^{13}C-NMR, 126 MHz,
$CDCl_3$
200 190 180 170 160 150 140 130 120 110 100 90 80 70 60 50 40 30 20 10 0 ppm

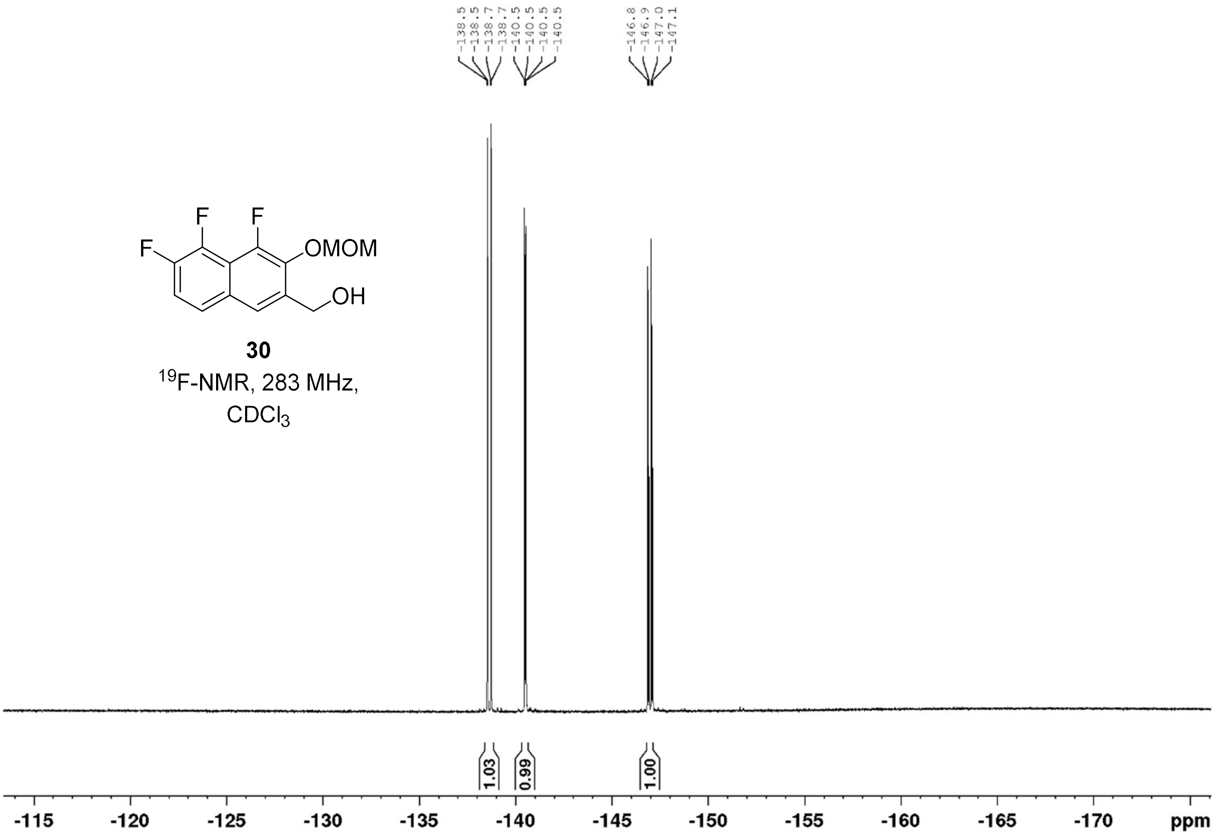
-138.5
-138.5
-138.7
-138.7
-140.5
-140.5
-140.5
-140.5
-146.8
-146.9
-147.0
-147.1
F
F
F
OMOM
OH
30
^{19}F-NMR, 283 MHz,
$CDCl_3$
1.03
0.99
1.00
-115 -120 -125 -130 -135 -140 -145 -150 -155 -160 -165 -170 ppm

8. 3-(bromomethyl)-1,7,8-trifluoro-2-(methoxymethoxy)naphthalene

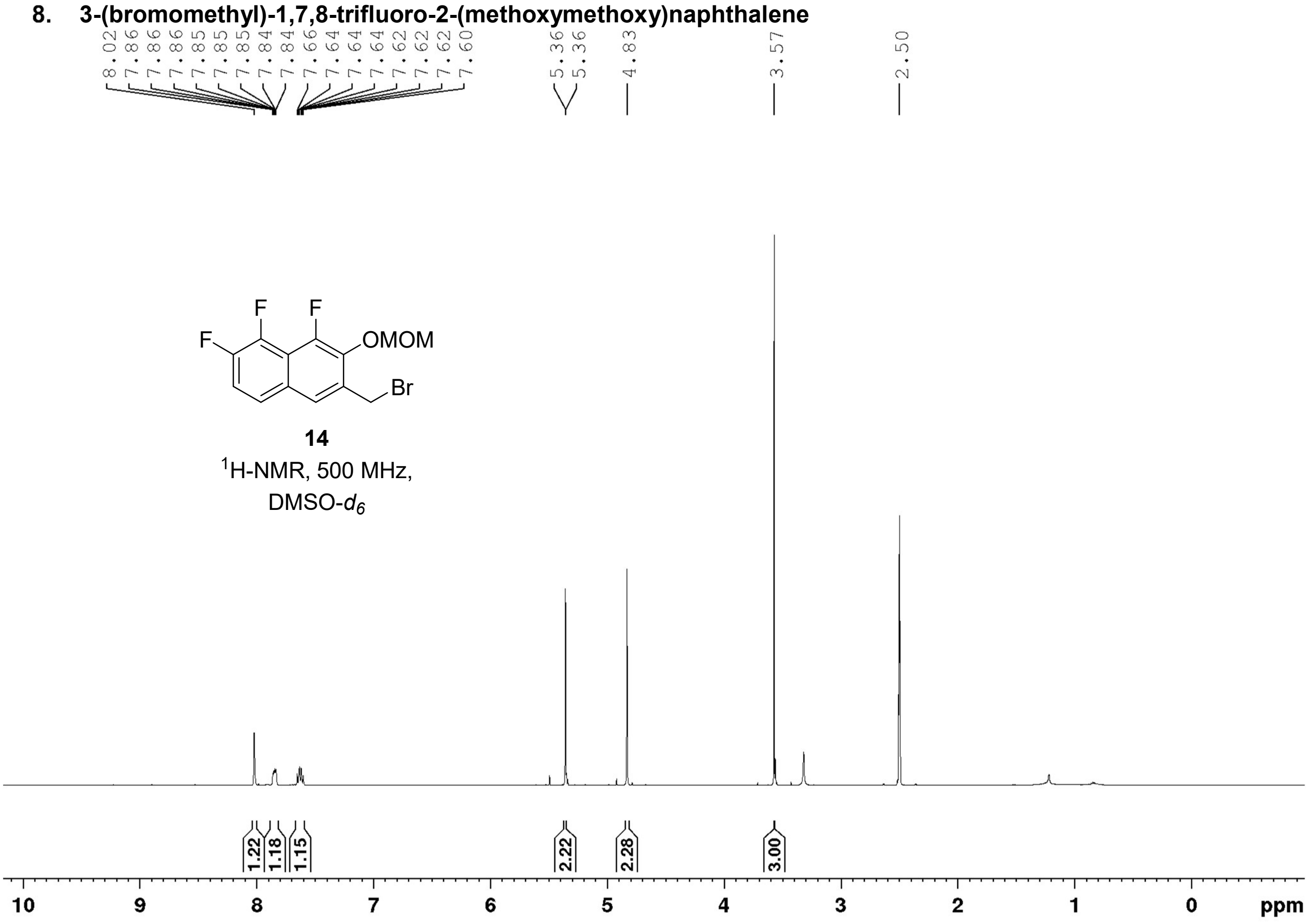

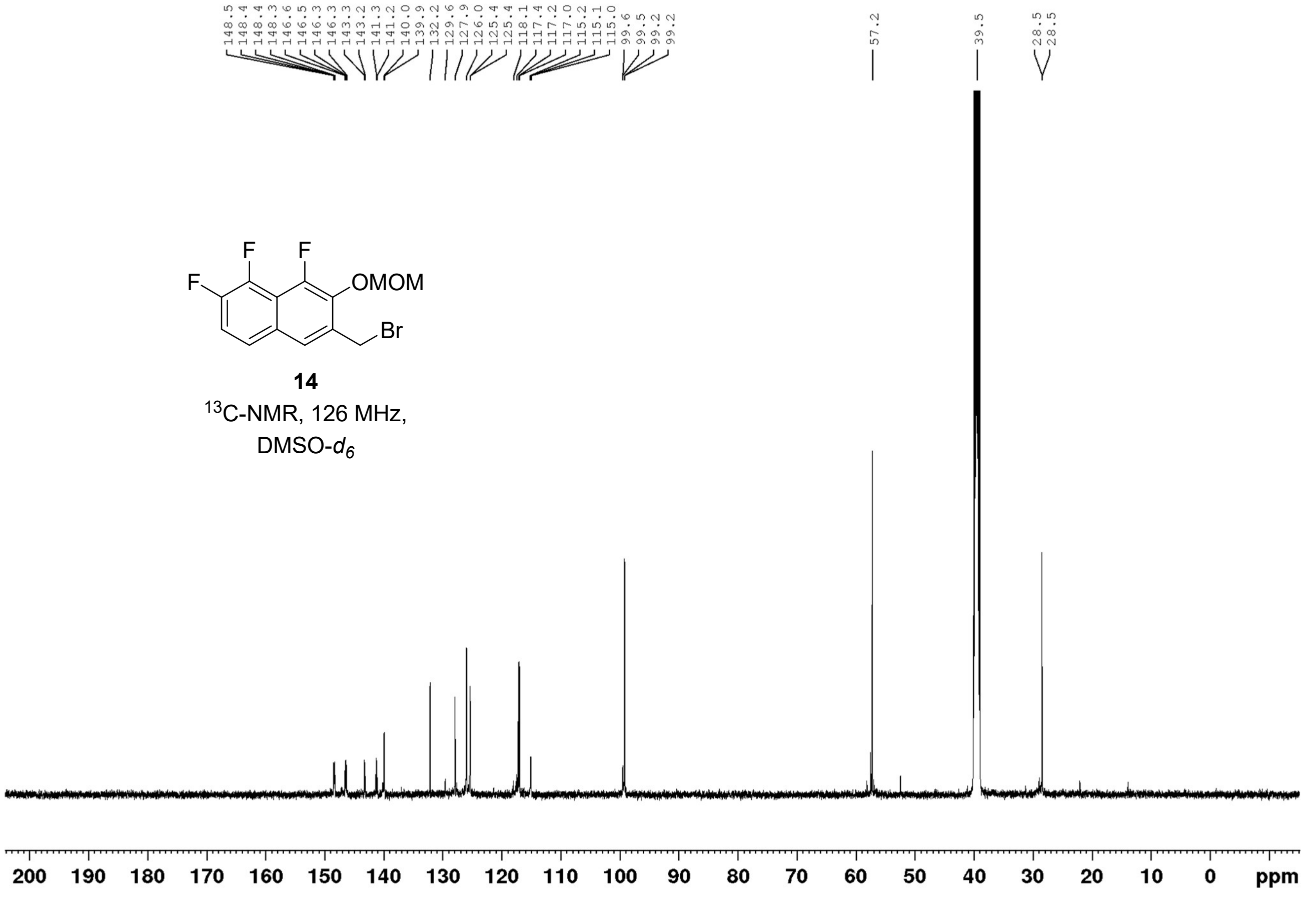

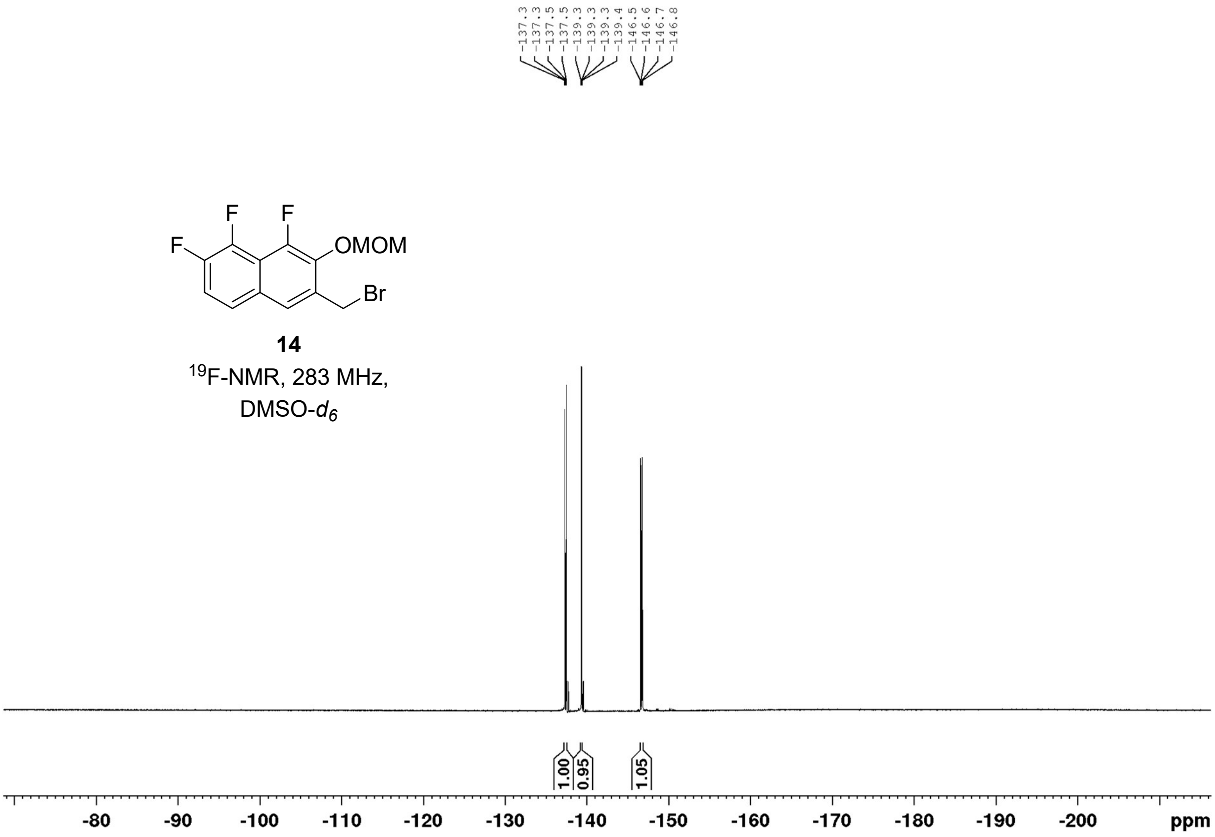

9. methyl 1,7,8-trifluoro-3-((4,5,6-trifluoro-3-(methoxymethoxy)naphthalen-2-yl)methyl)-2-naphthoate

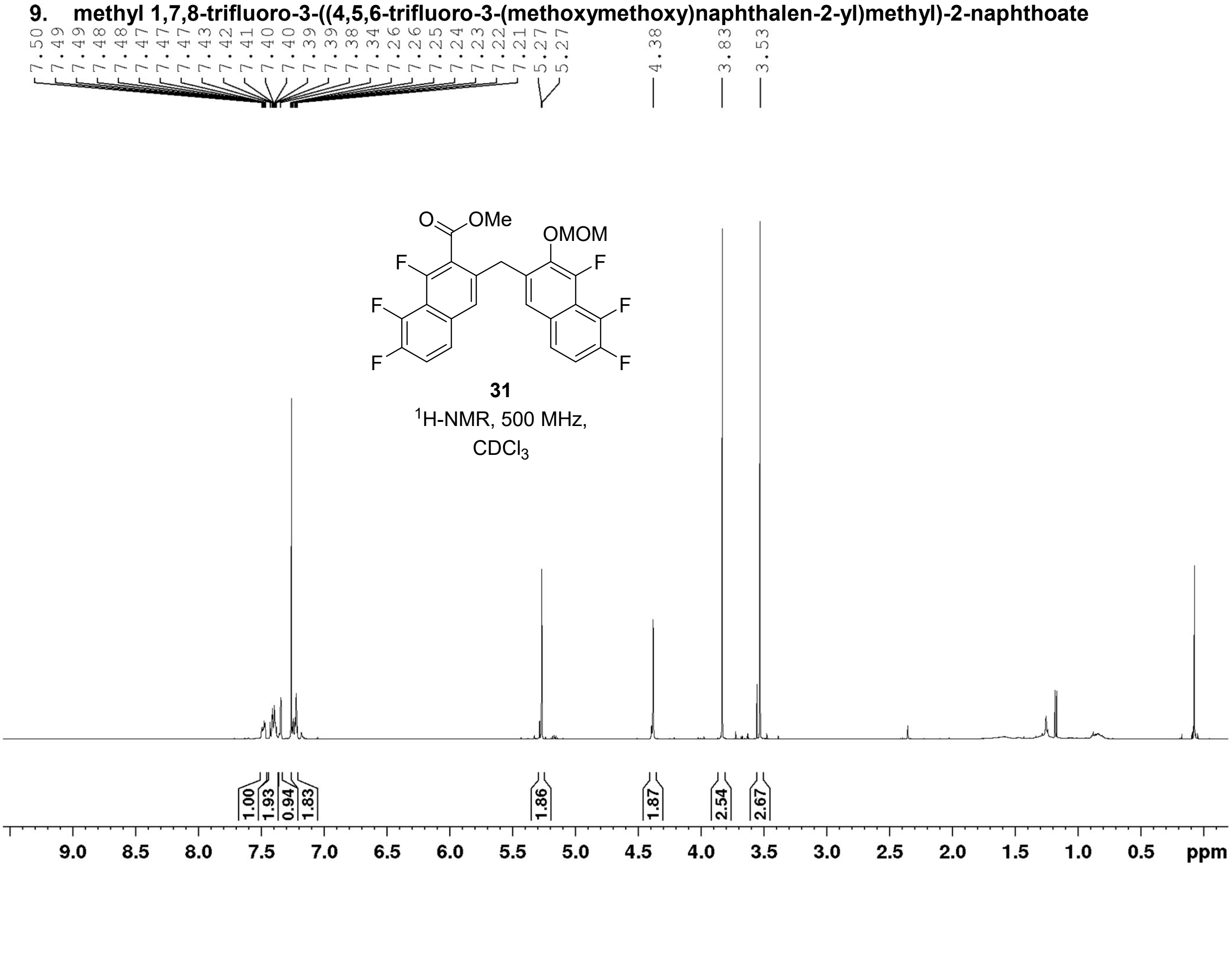

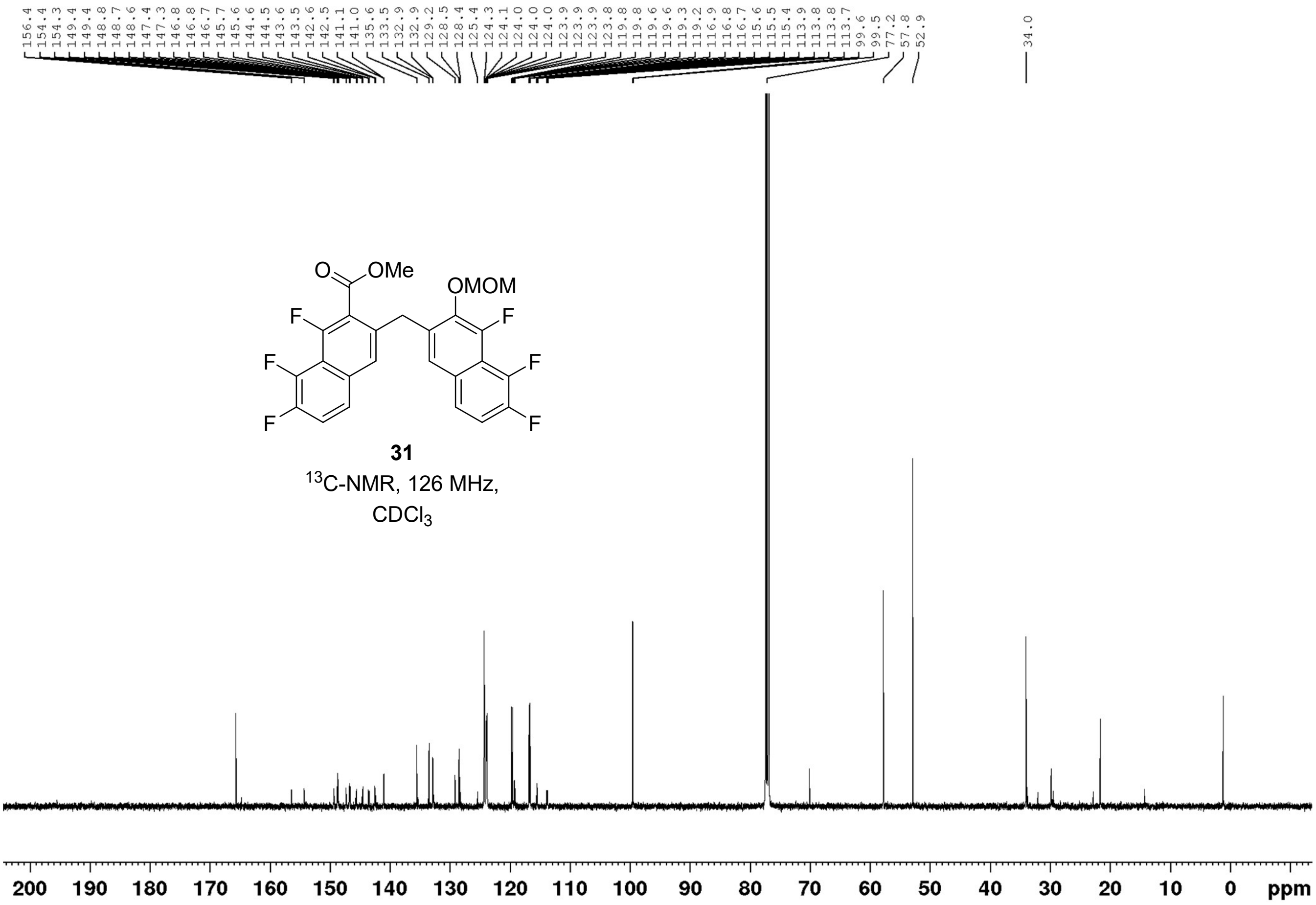

31

^{13}C-NMR, 126 MHz, $CDCl_3$

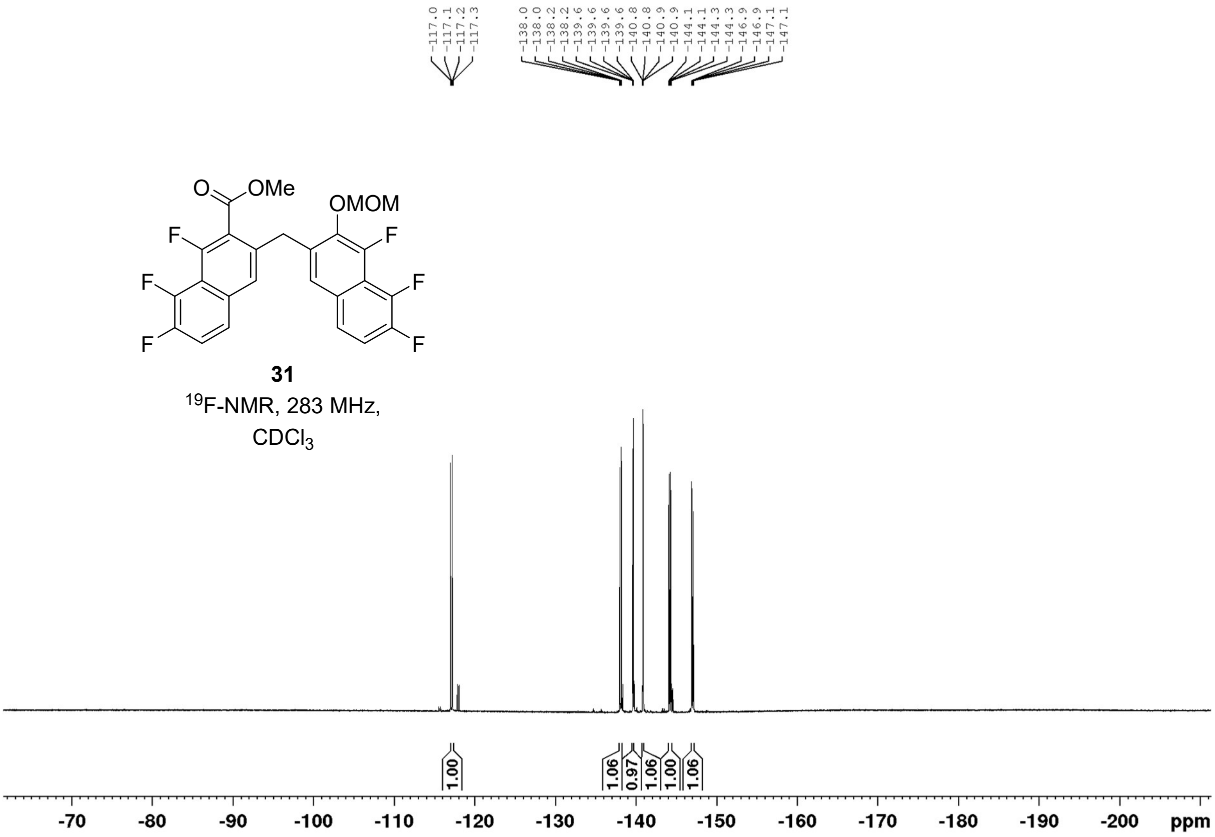

31

^{19}F-NMR, 283 MHz, $CDCl_3$

10. methyl 1,7,8-trifluoro-3-((4,5,6-trifluoro-3-hydroxynaphthalen-2-yl)methyl)-2-naphthoate

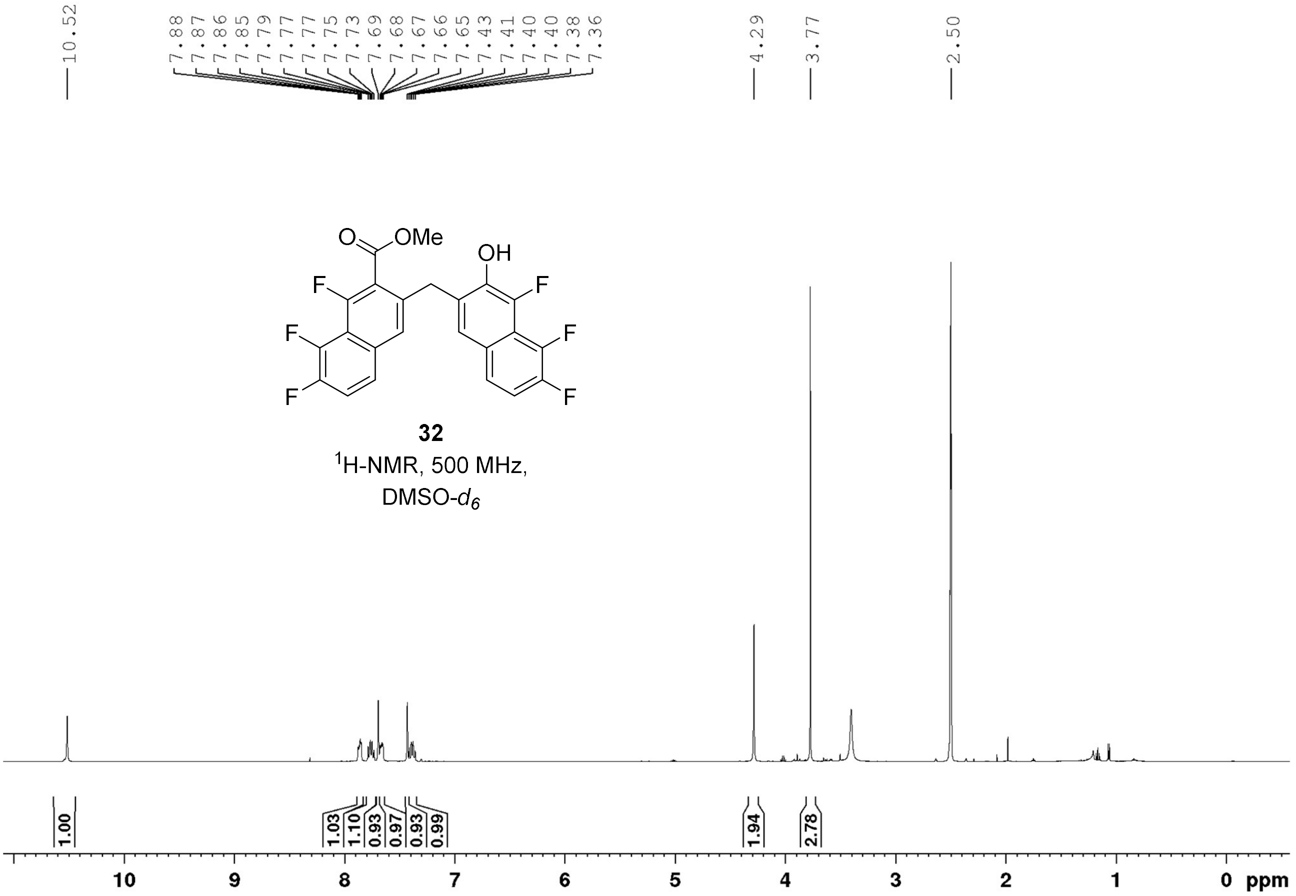

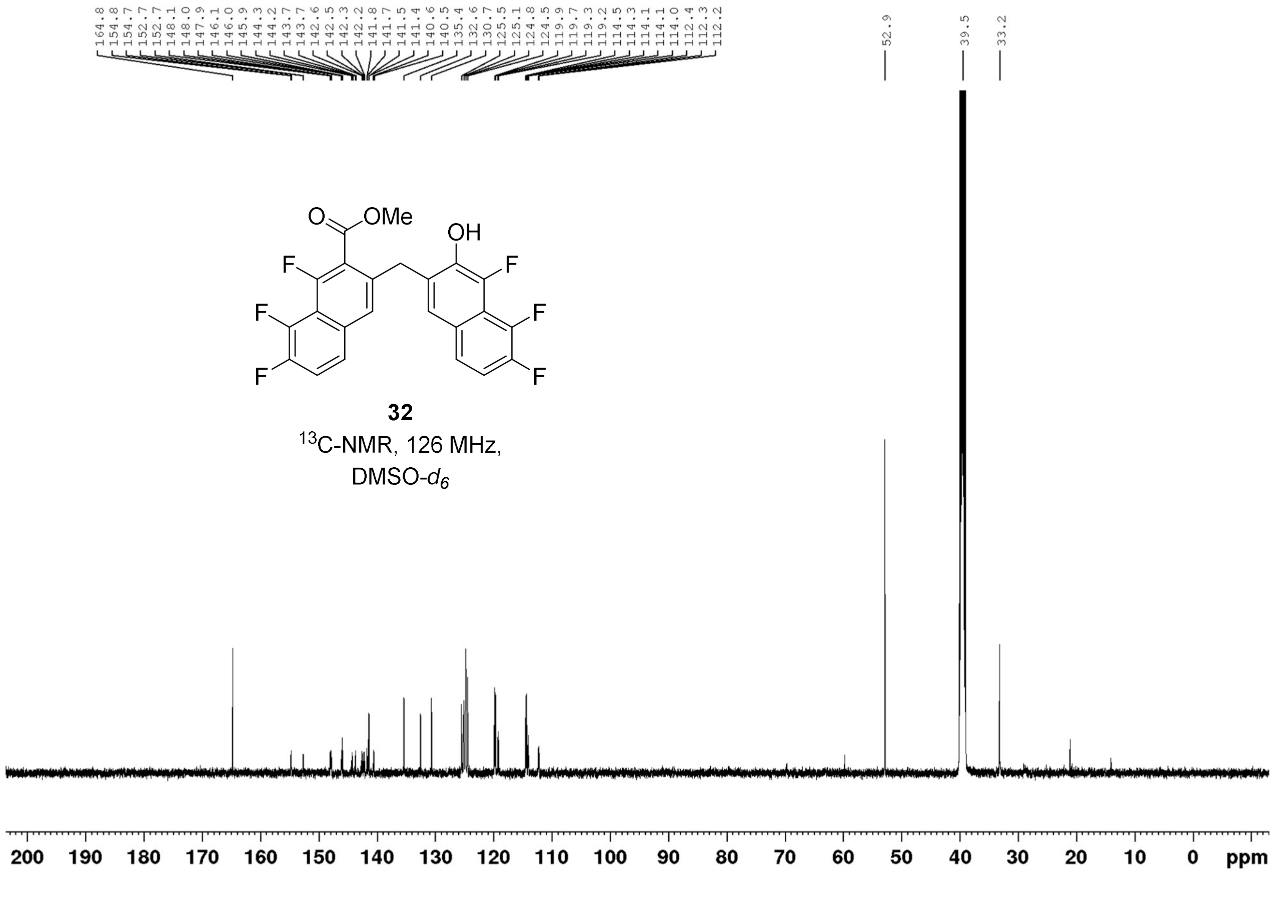

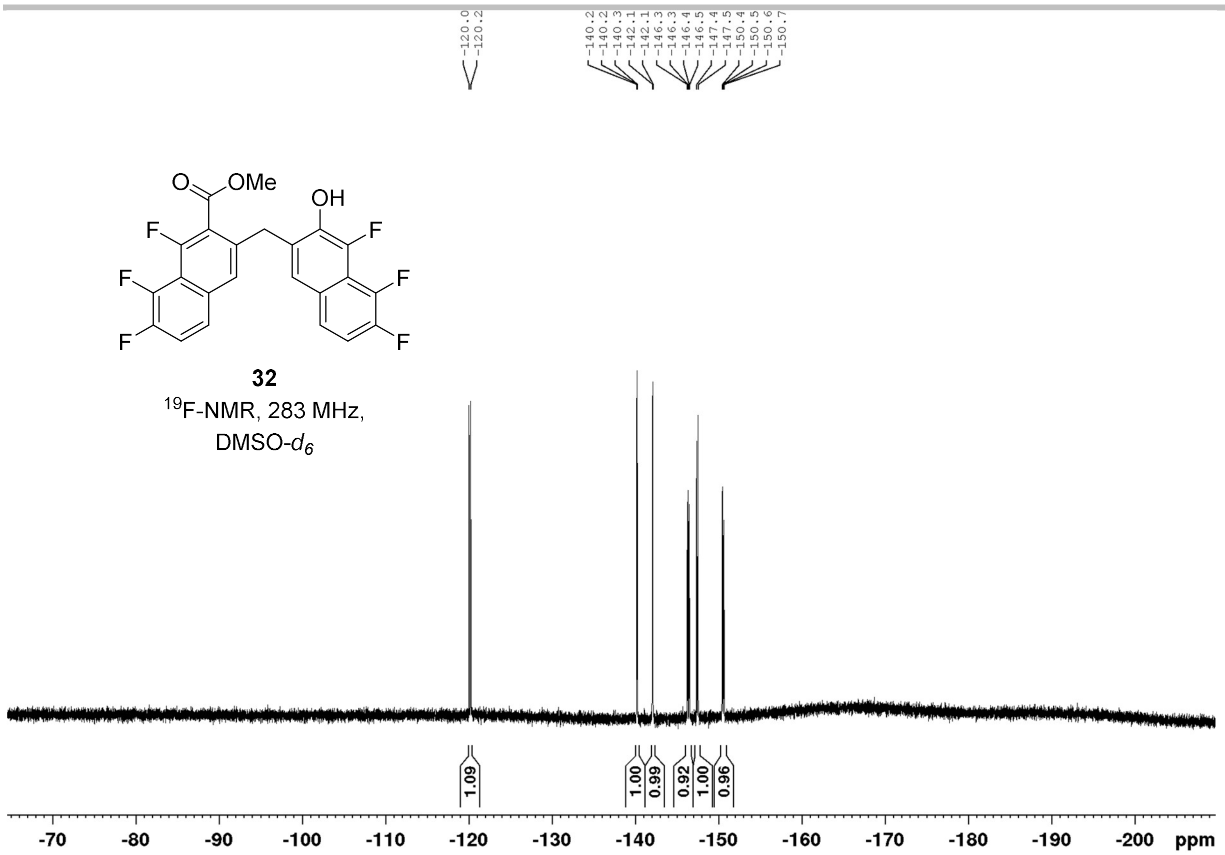

11. methyl 1,7,8-trifluoro-3-((4,5,6-trifluoro-3-(((trifluoromethyl)sulfonyl)oxy)naphthalen-2-yl)methyl)-2-naphthoate

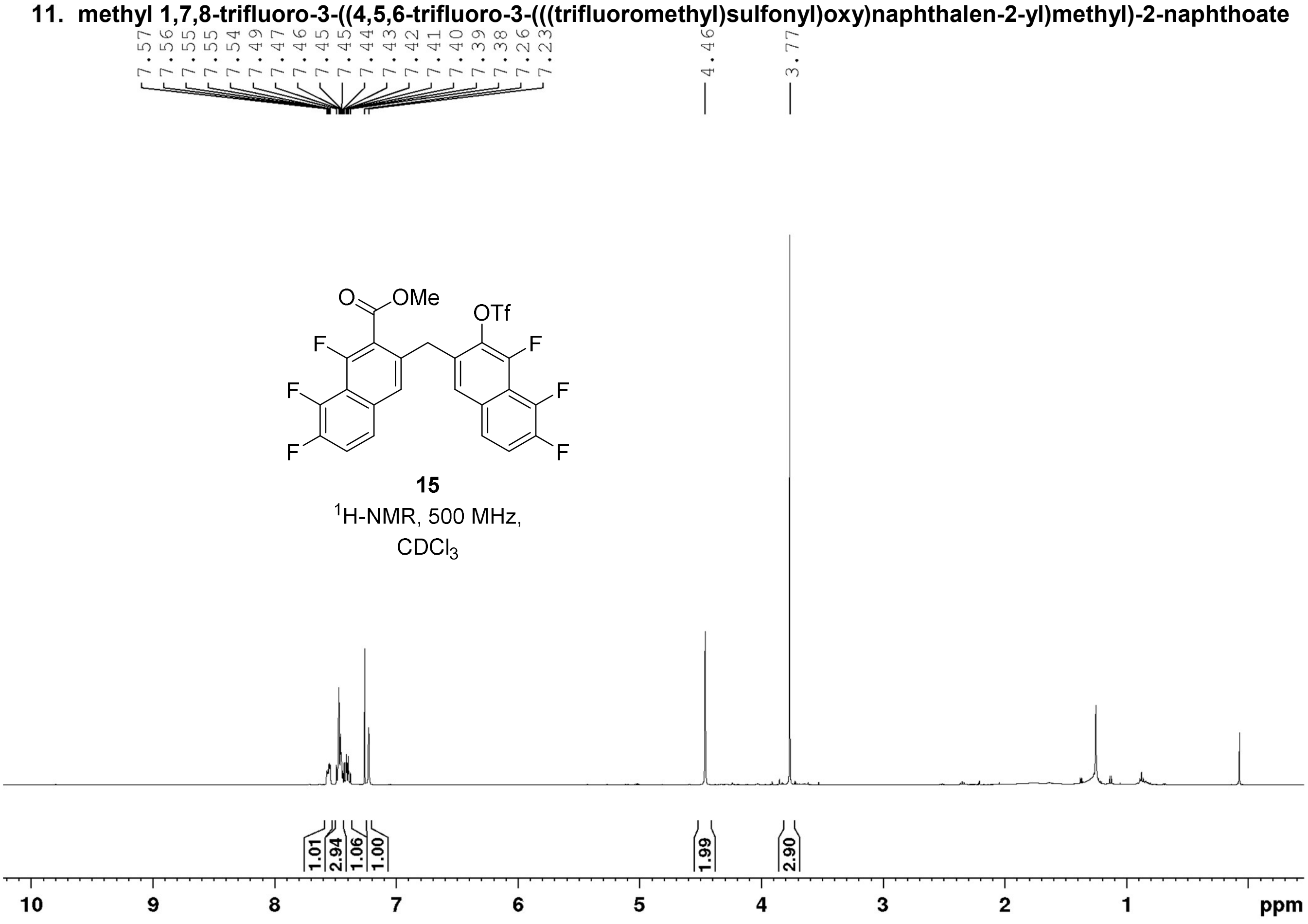

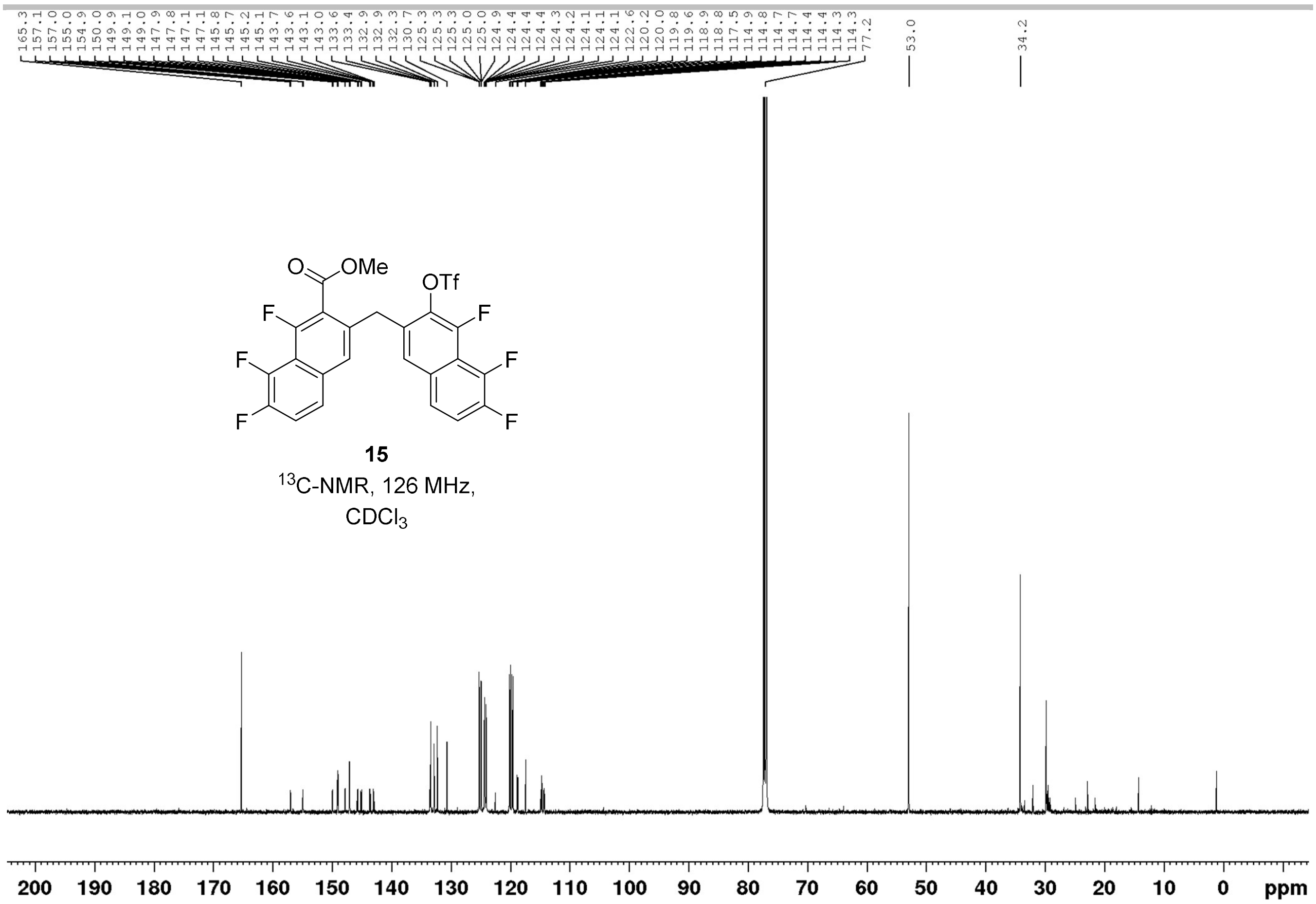
165.3
157.1
157.0
155.0
154.9
150.0
149.9
149.1
149.0
147.9
147.8
147.1
147.1
145.8
145.7
145.2
145.1
143.7
143.6
143.1
143.0
133.6
133.4
132.9
132.9
132.3
130.7
125.3
125.3
125.3
125.0
125.0
124.9
124.4
124.4
124.4
124.3
124.2
124.1
124.1
124.1
122.6
120.2
120.0
119.8
119.6
118.9
118.8
117.5
114.9
114.8
114.7
114.7
114.4
114.4
114.3
114.3
77.2
53.0
34.2
O
OMe
OTf
F
F
F
F
F
F
15
13C-NMR, 126 MHz,
CDCl3
200 190 180 170 160 150 140 130 120 110 100 90 80 70 60 50 40 30 20 10 0 ppm

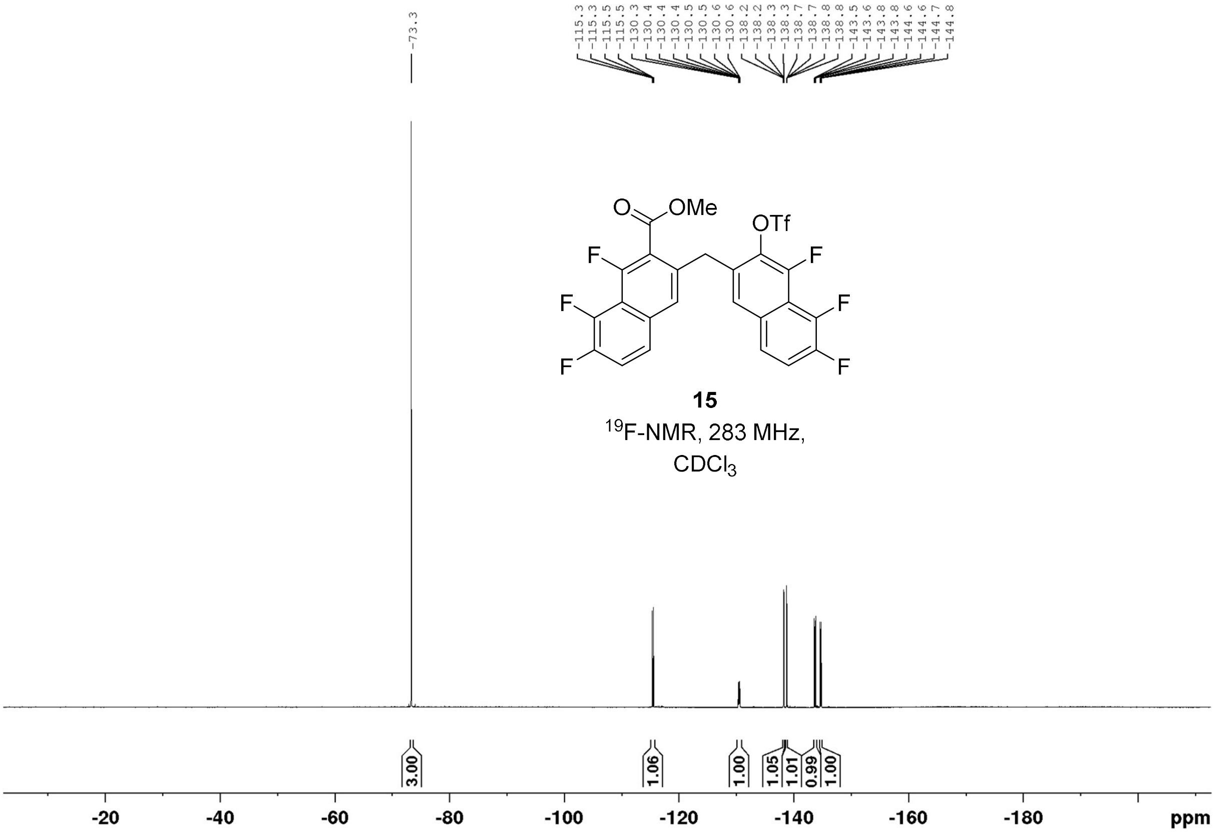
-73.3
-115.3
-115.3
-115.5
-115.5
-130.3
-130.4
-130.4
-130.4
-130.5
-130.5
-130.6
-130.6
-138.2
-138.2
-138.3
-138.3
-138.7
-138.7
-138.8
-138.8
-143.5
-143.6
-143.8
-143.8
-144.6
-144.6
-144.7
-144.8
O
OMe
OTf
F
F
F
F
F
F
15
19F-NMR, 283 MHz,
CDCl3
3.00
1.06
1.00
1.05
1.01
0.99
1.00
-20 -40 -60 -80 -100 -120 -140 -160 -180 ppm

12. 1,7,8-trifluoro-3-((4,5,6-trifluoro-3-(hydroxymethyl)naphthalen-2-yl)methyl)naphthalen-2-yl trifluoromethanesulfonate

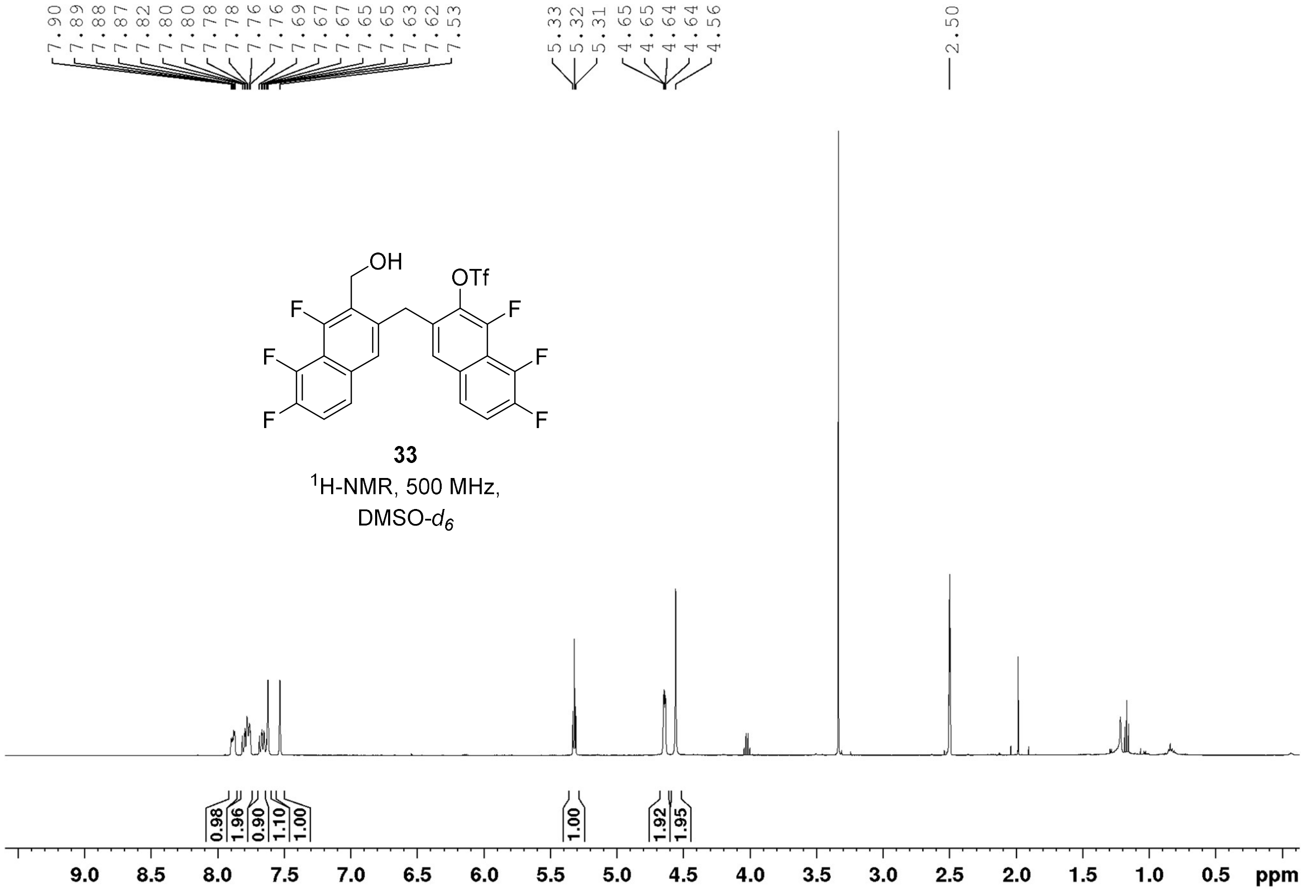

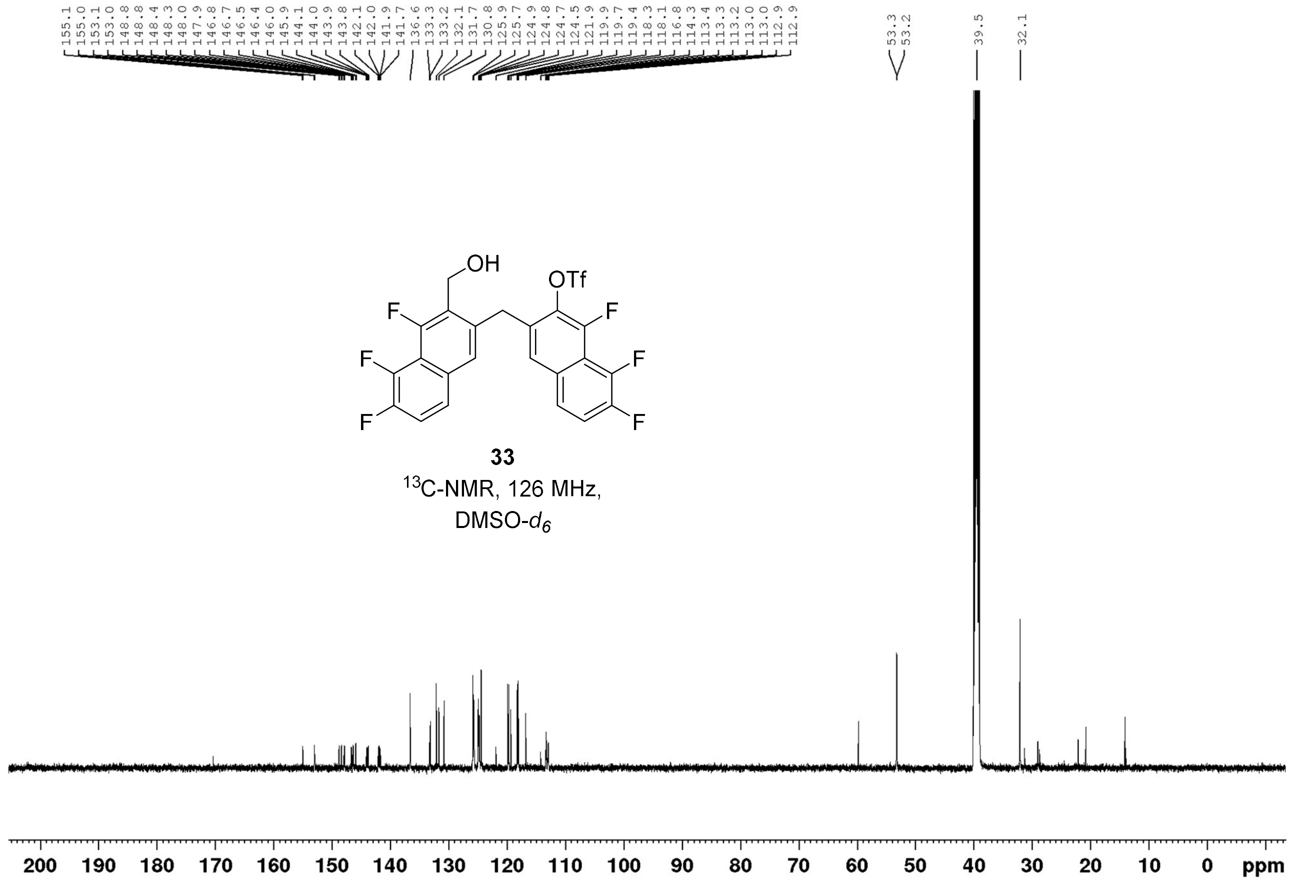

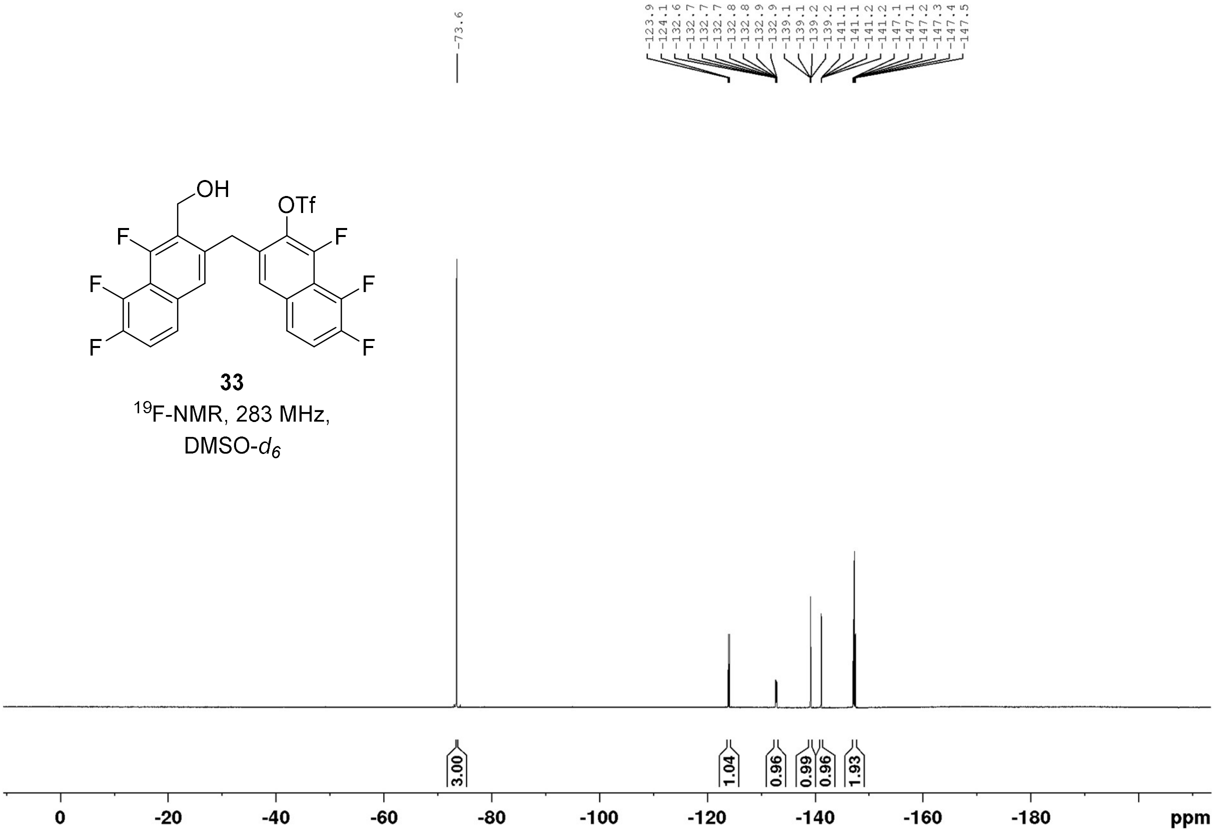

13. 1,7,8-trifluoro-3-((4,5,6-trifluoro-3-formylnaphthalen-2-yl)methyl)naphthalen-2-yl trifluoromethanesulfonate

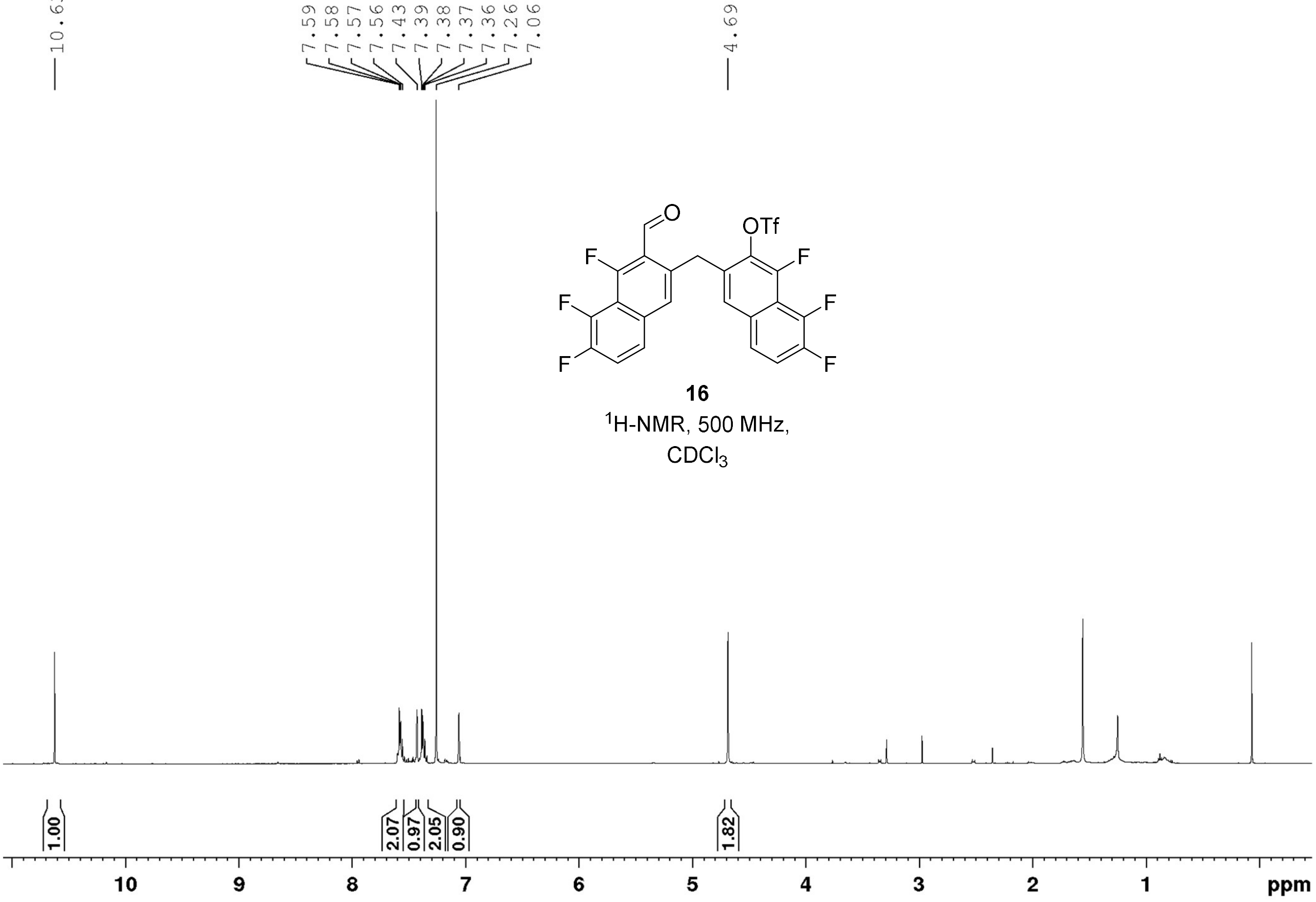

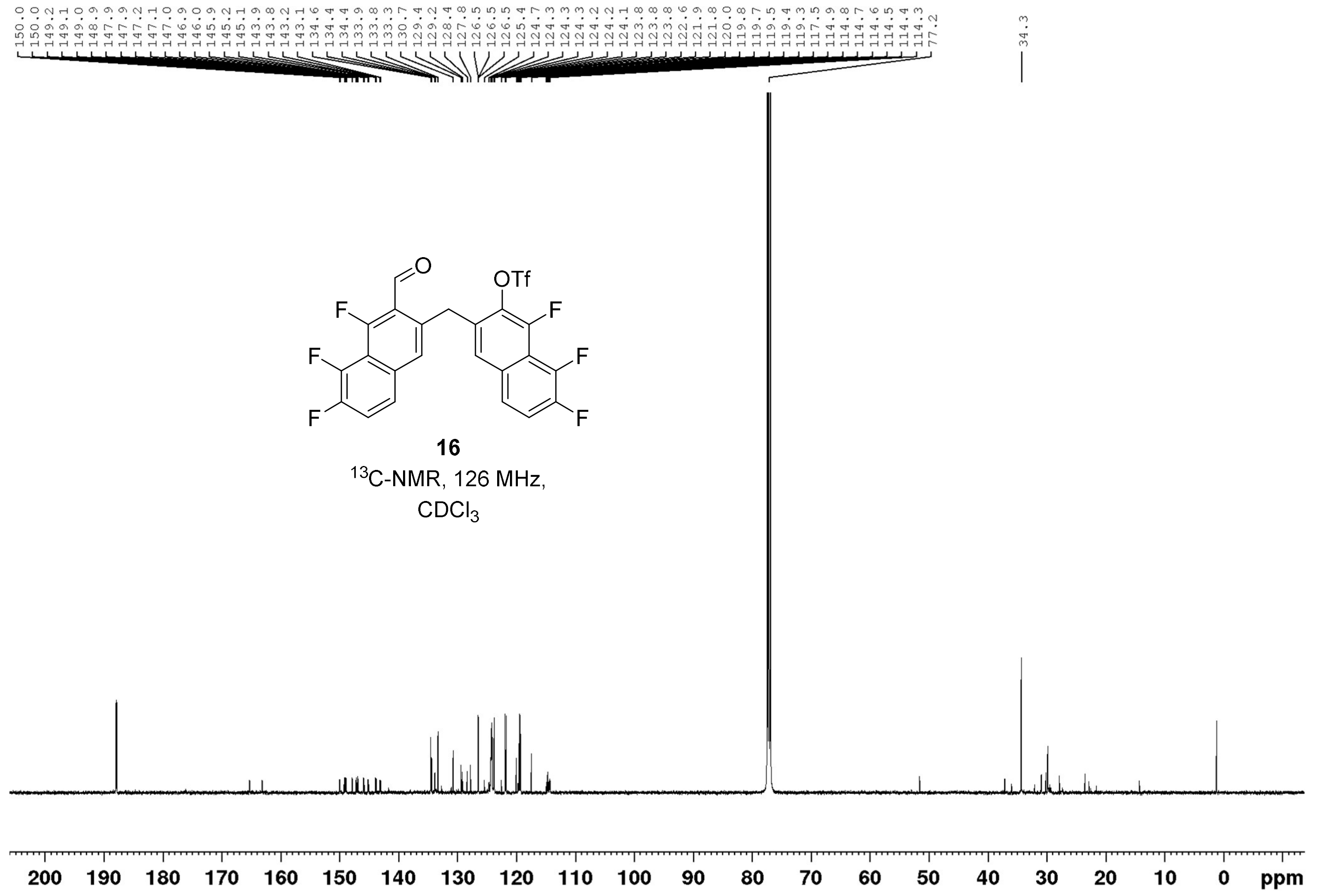
150.0
150.0
149.2
149.1
149.0
148.9
147.9
147.9
147.2
147.1
147.0
146.9
146.0
145.9
145.2
145.1
143.9
143.8
143.2
143.1
134.6
134.4
134.4
133.9
133.8
133.3
130.7
129.4
129.2
128.4
127.8
126.5
126.5
126.5
125.4
124.7
124.3
124.3
124.3
124.2
124.2
124.1
123.8
123.8
123.8
122.6
121.9
121.8
120.0
119.8
119.7
119.5
119.4
119.3
117.5
114.9
114.8
114.7
114.6
114.5
114.4
114.3
77.2
34.3
O
OTf
F
F
F
F
F
F
16
13C-NMR, 126 MHz,
CDCl3
200 190 180 170 160 150 140 130 120 110 100 90 80 70 60 50 40 30 20 10 0 ppm

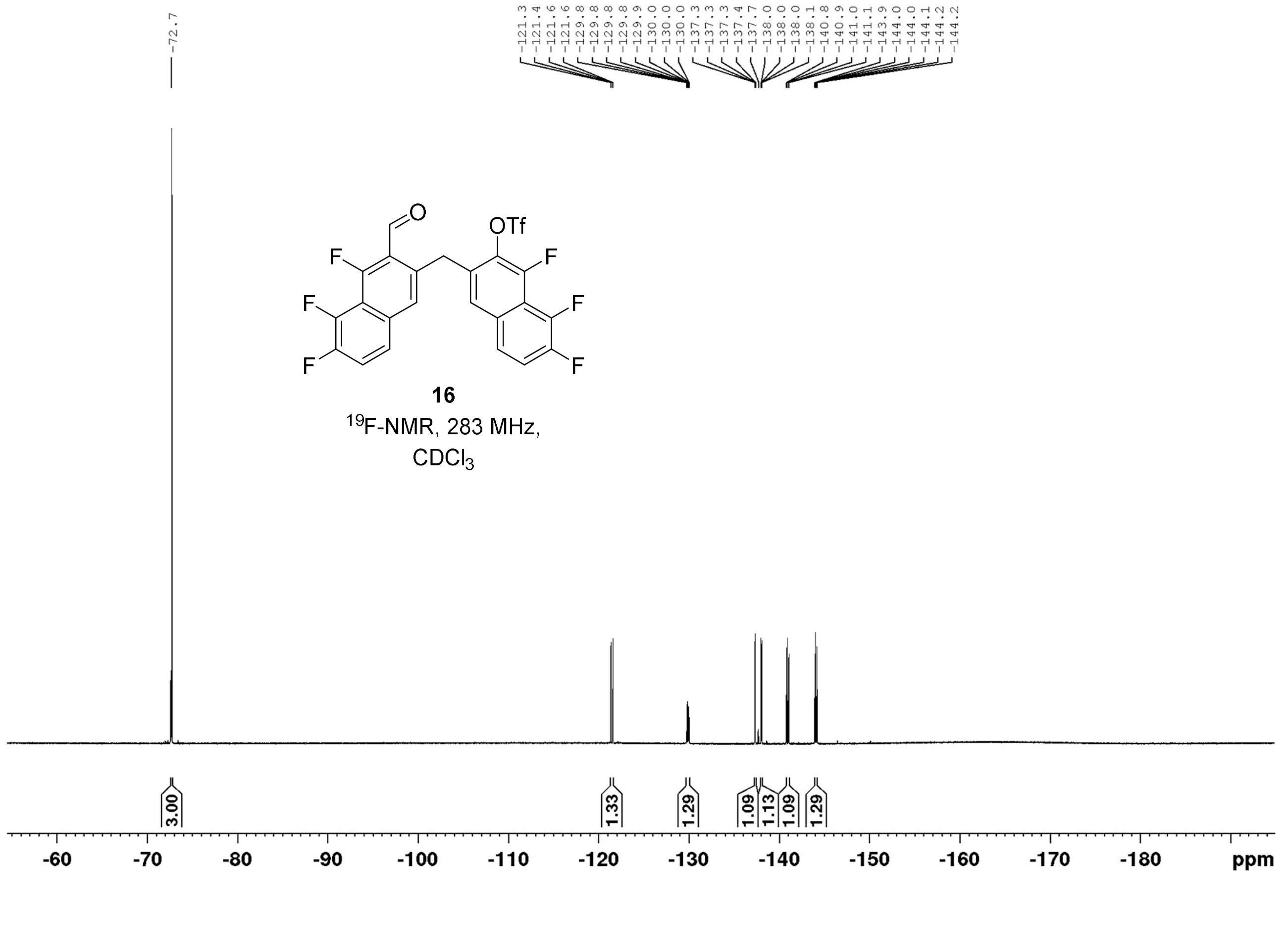
-72.7
-121.3
-121.4
-121.6
-121.6
-129.8
-129.8
-129.8
-129.9
-130.0
-130.0
-130.0
-137.3
-137.3
-137.3
-137.4
-137.7
-138.0
-138.0
-138.0
-138.1
-140.8
-140.9
-141.0
-141.1
-143.9
-144.0
-144.1
-144.2
-144.2
O
OTf
F
F
F
F
F
F
16
19F-NMR, 283 MHz,
CDCl3
3.00
1.33
1.29
1.09
1.13
1.09
1.29
-60 -70 -80 -90 -100 -110 -120 -130 -140 -150 -160 -170 -180 ppm

14. 3,4,5,7,8,9-hexafluoro-6,13-dihydropentacen-6-ol

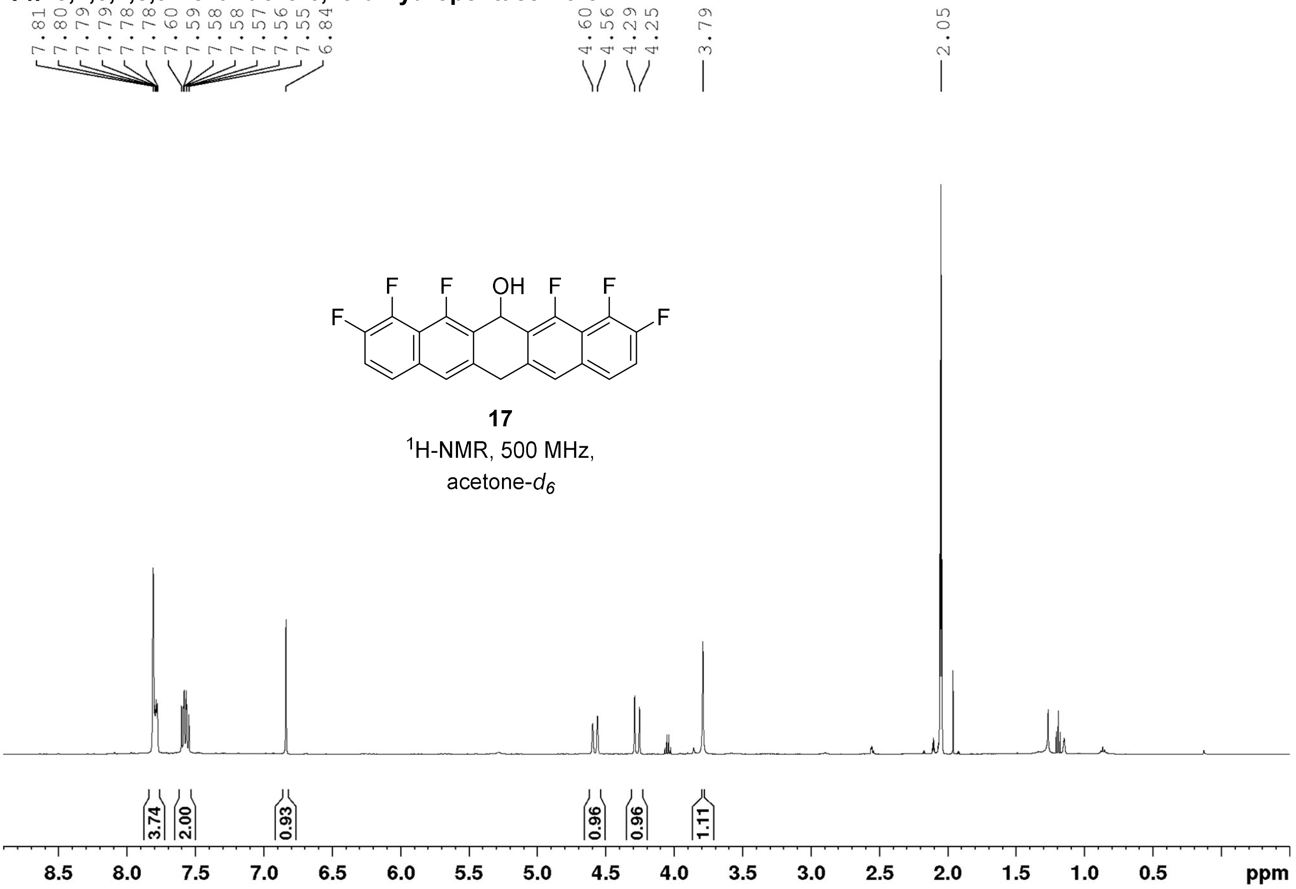

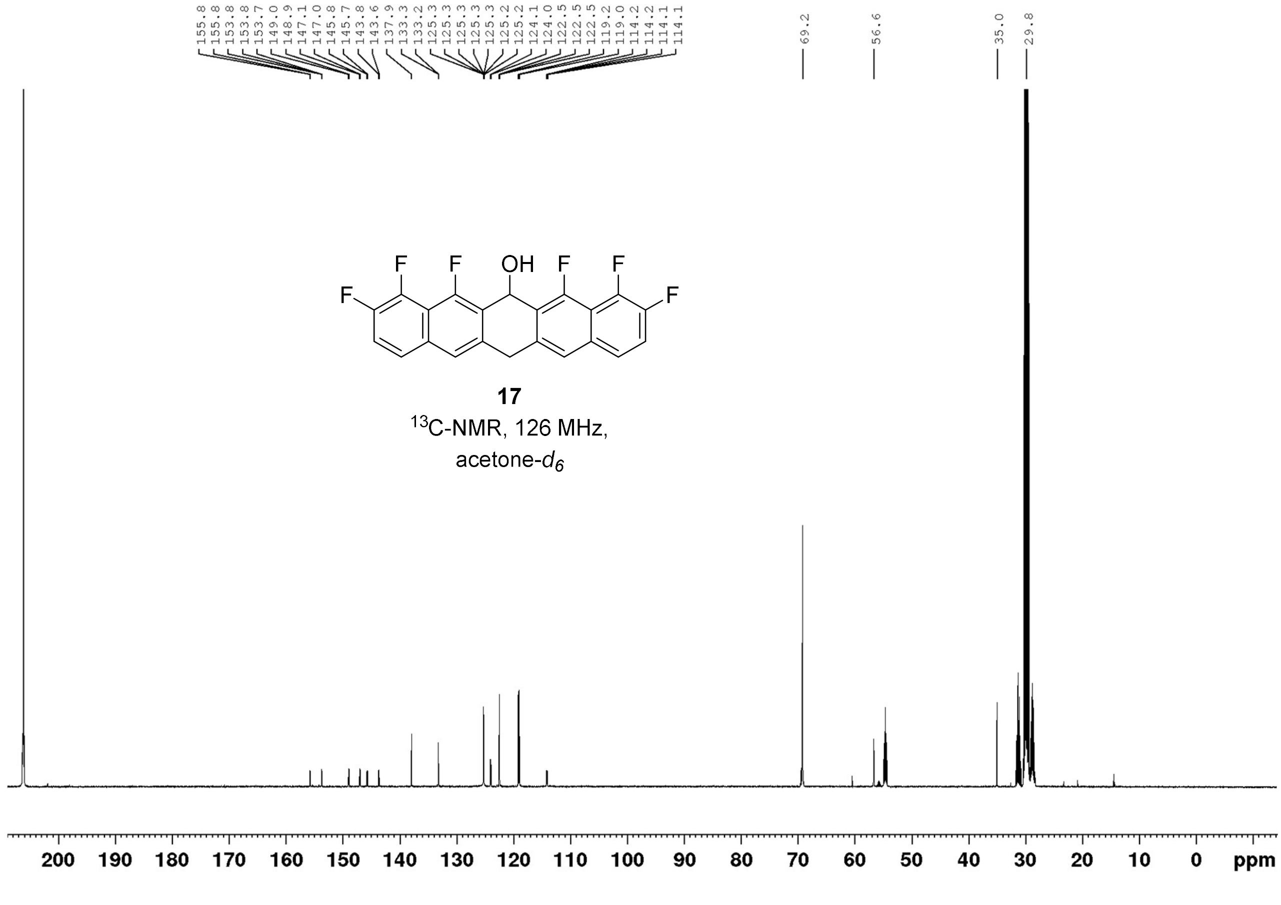

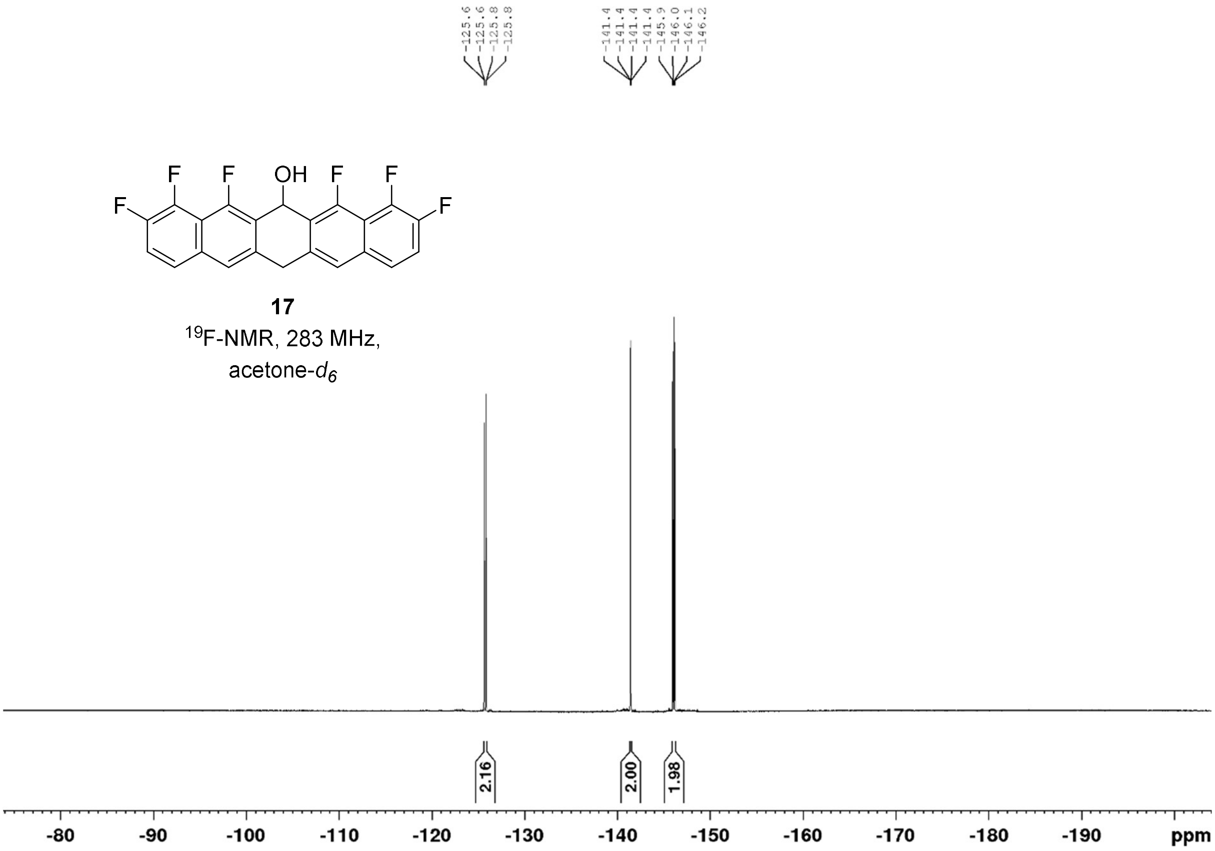

15. 1,2,10,11,12,14-hexafluoropentacene

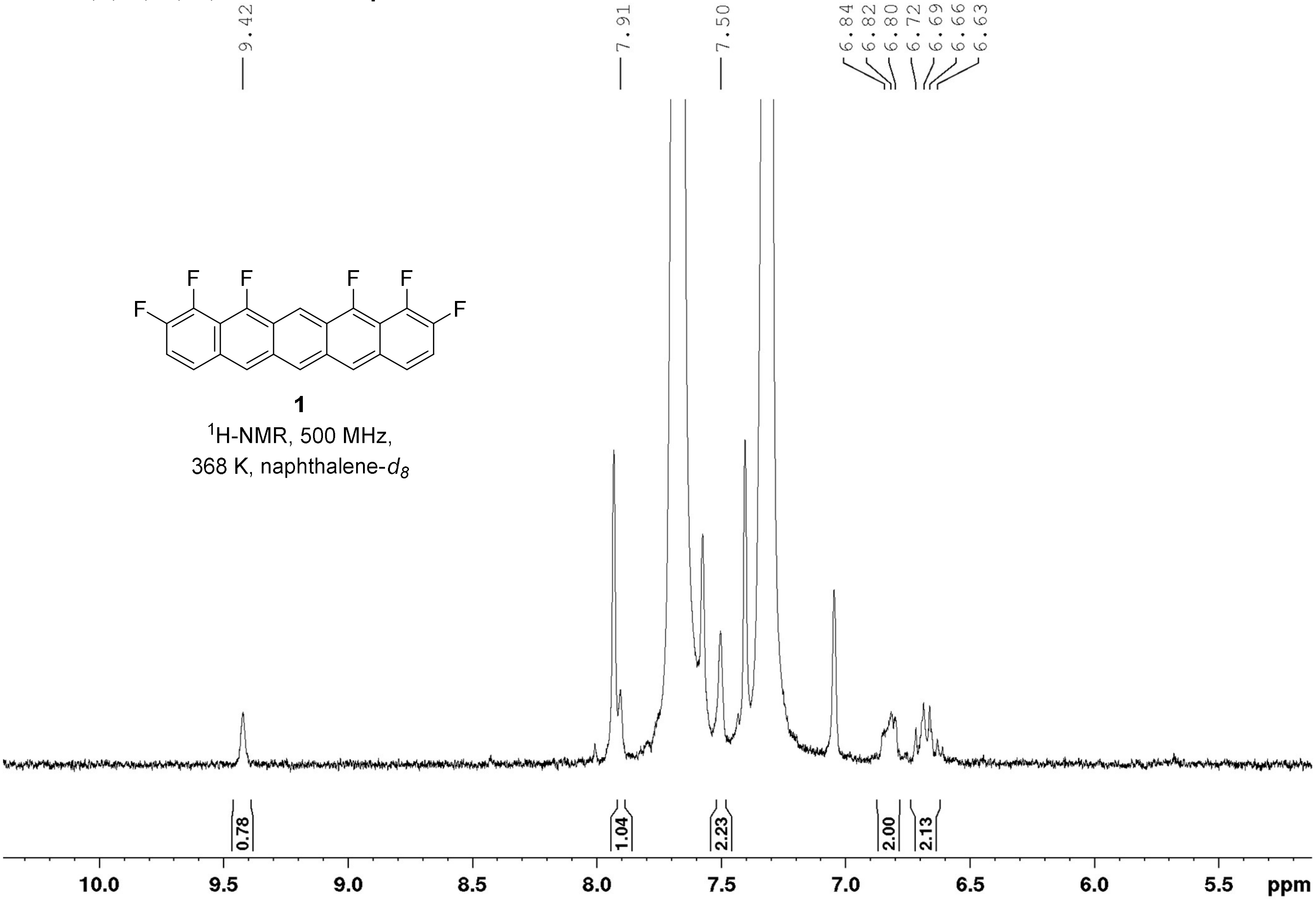

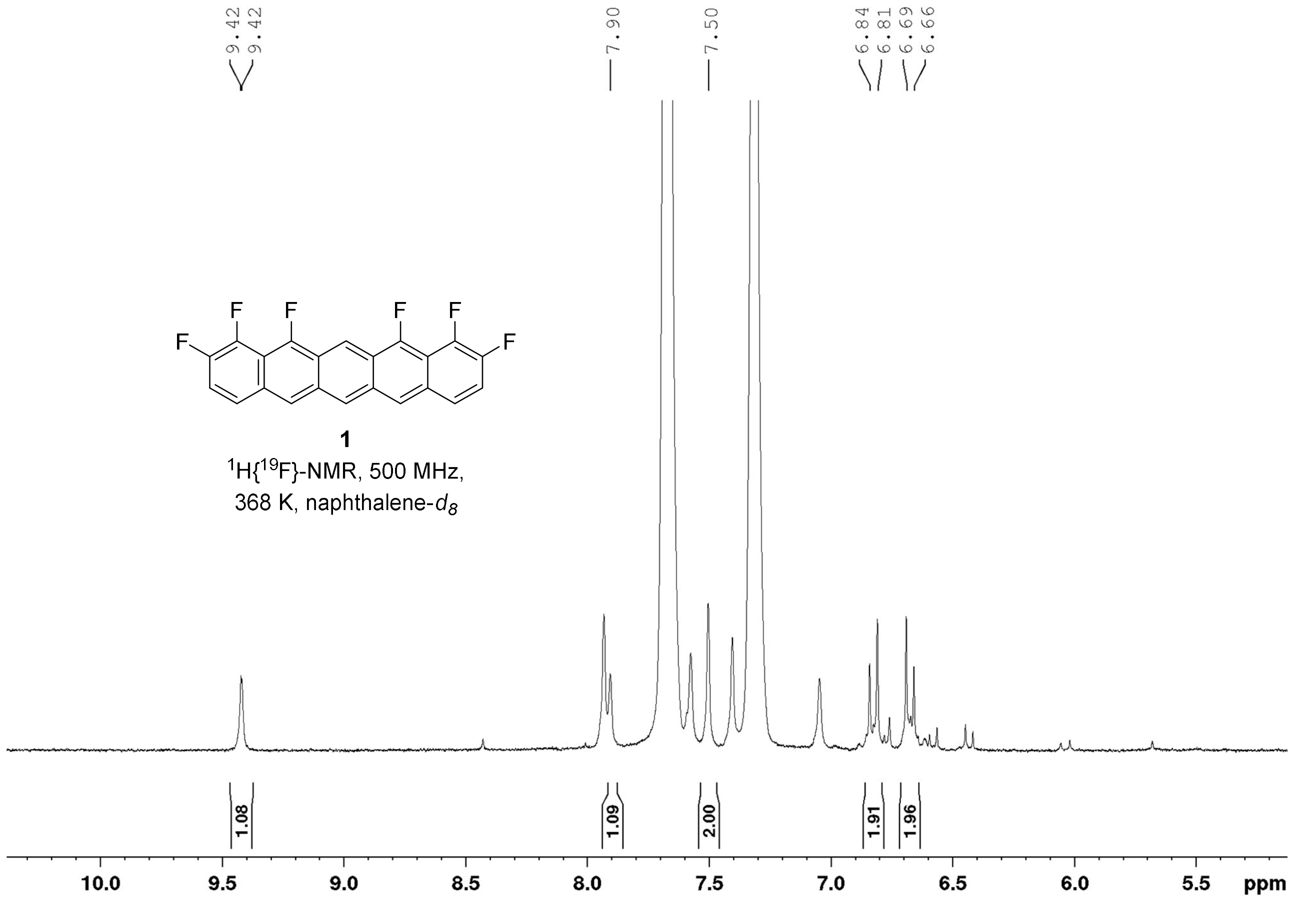
9.42
9.42
7.90
7.50
6.84
6.81
6.69
6.66
F
1
$^1H\{^{19}F\}$-NMR, 500 MHz,
368 K, naphthalene-d_8
1.08
1.09
2.00
1.91
1.96
10.0
9.5
9.0
8.5
8.0
7.5
7.0
6.5
6.0
5.5
ppm

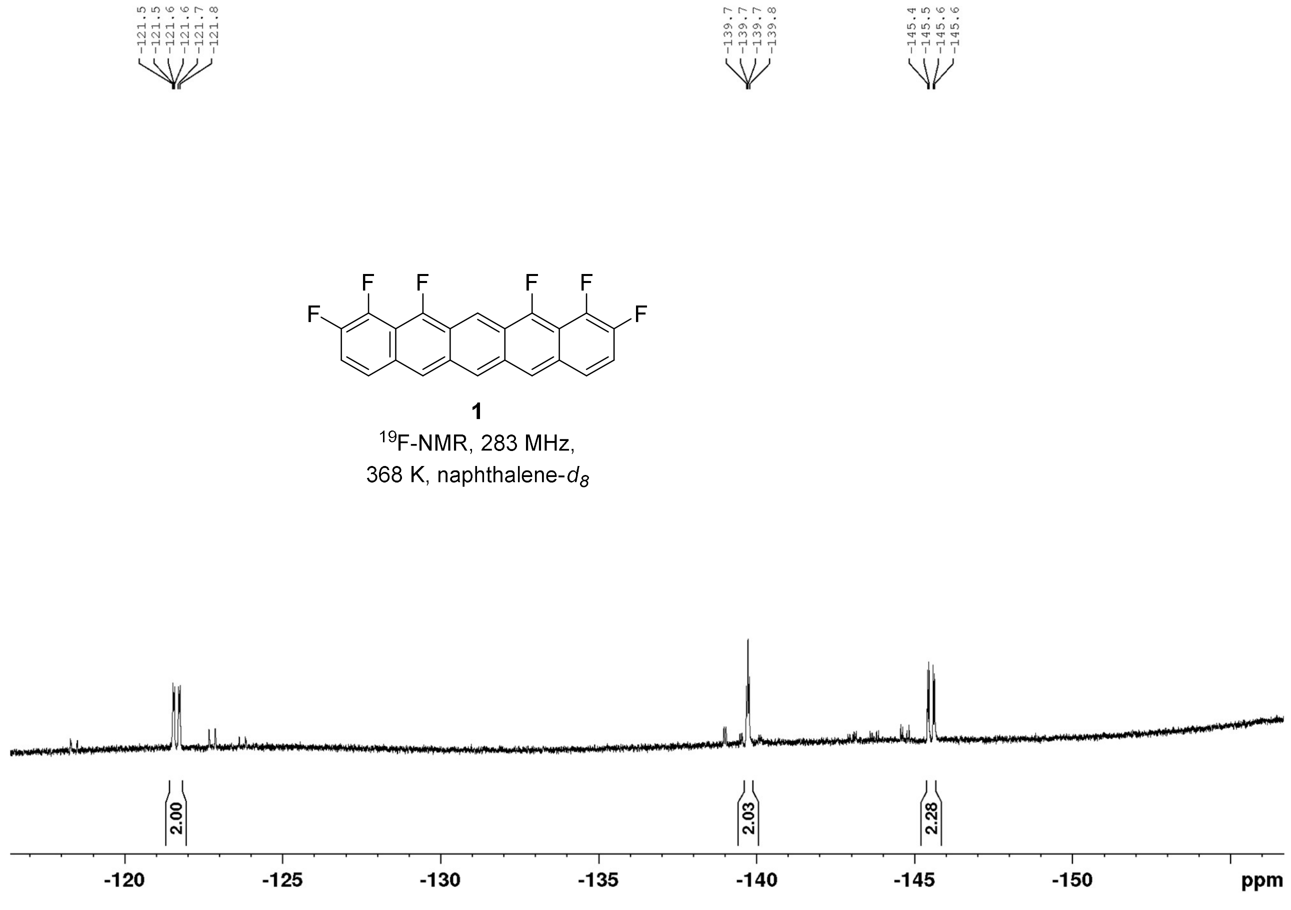
-121.5
-121.5
-121.6
-121.6
-121.7
-121.8
-139.7
-139.7
-139.7
-139.8
-145.4
-145.5
-145.6
-145.6
F
1
^{19}F-NMR, 283 MHz,
368 K, naphthalene-d_8
2.00
2.03
2.28
-120
-125
-130
-135
-140
-145
-150
ppm

16. methyl 3,4-difluoro-2-methoxybenzoate

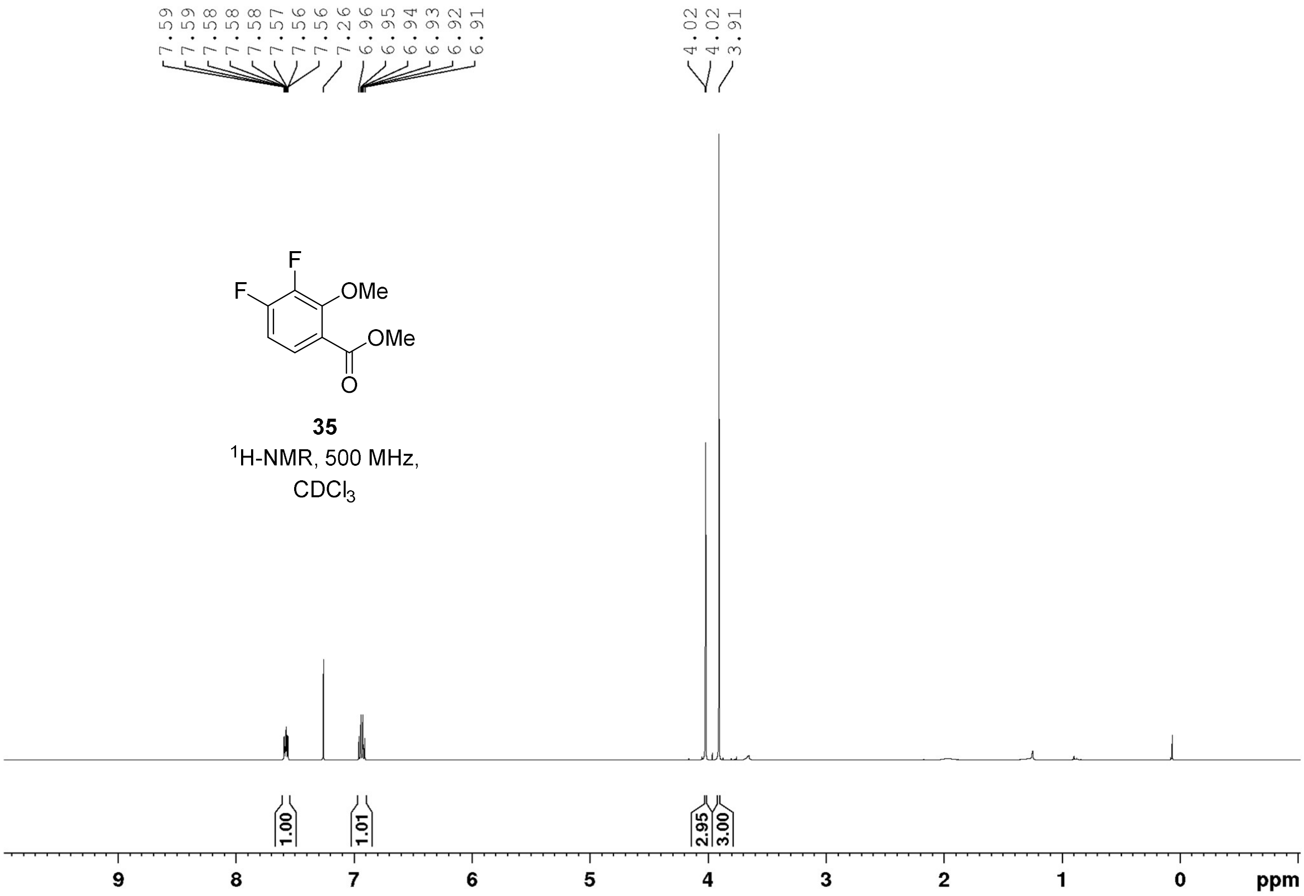

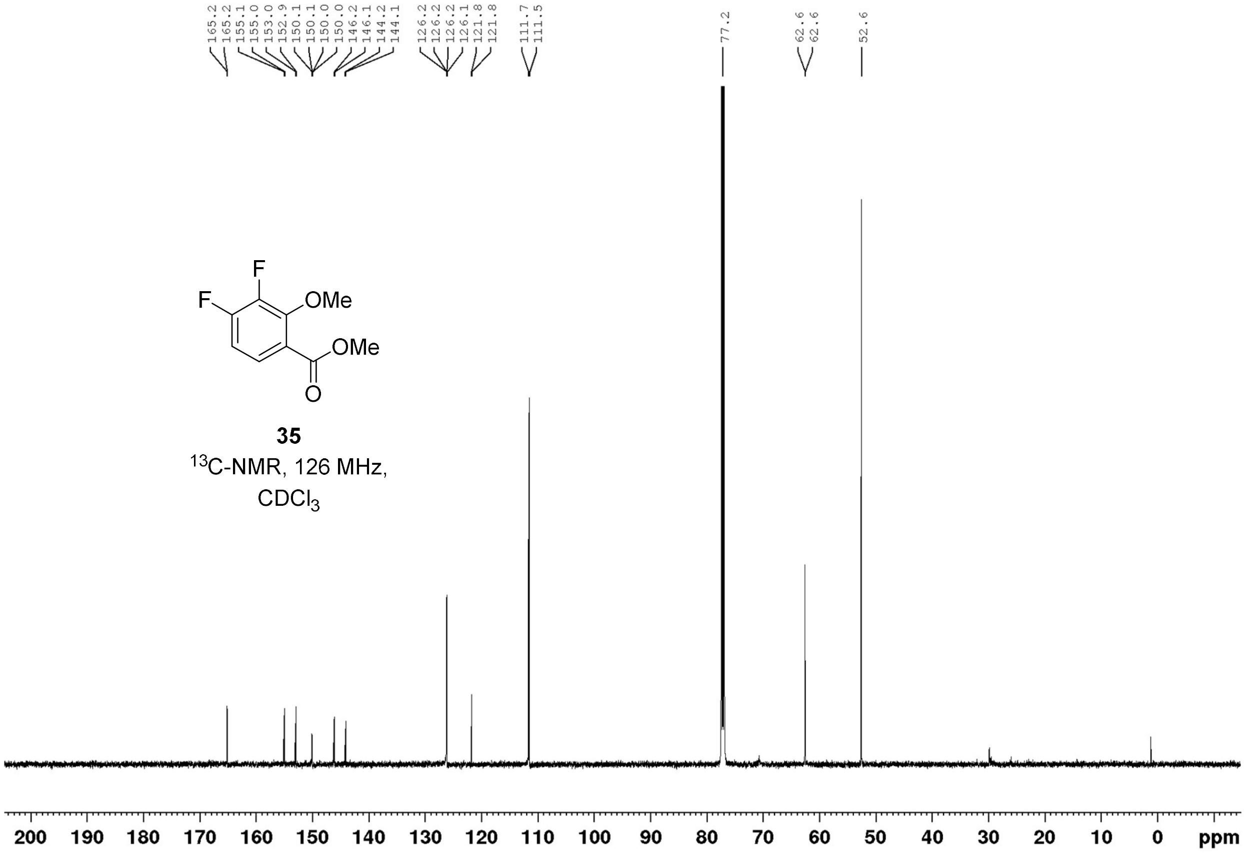

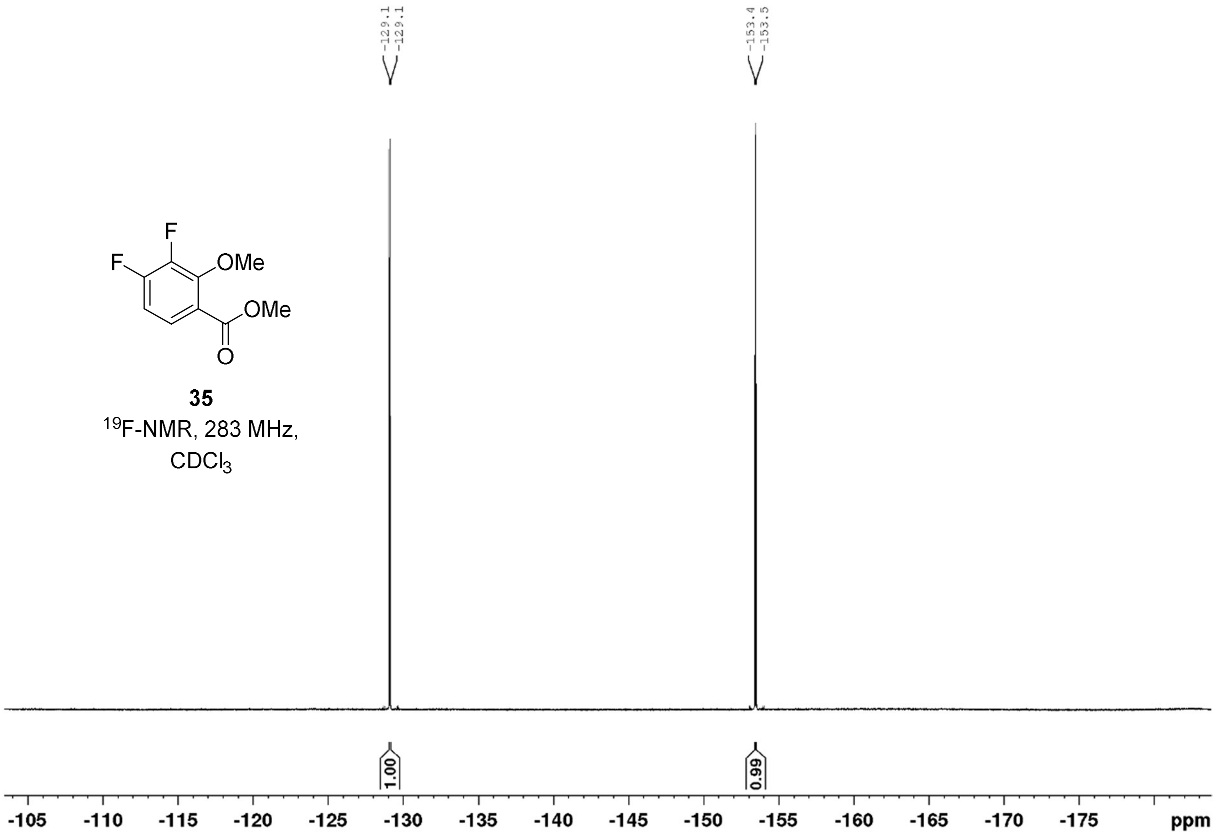

17. (3,4-difluoro-2-methoxyphenyl)methanol

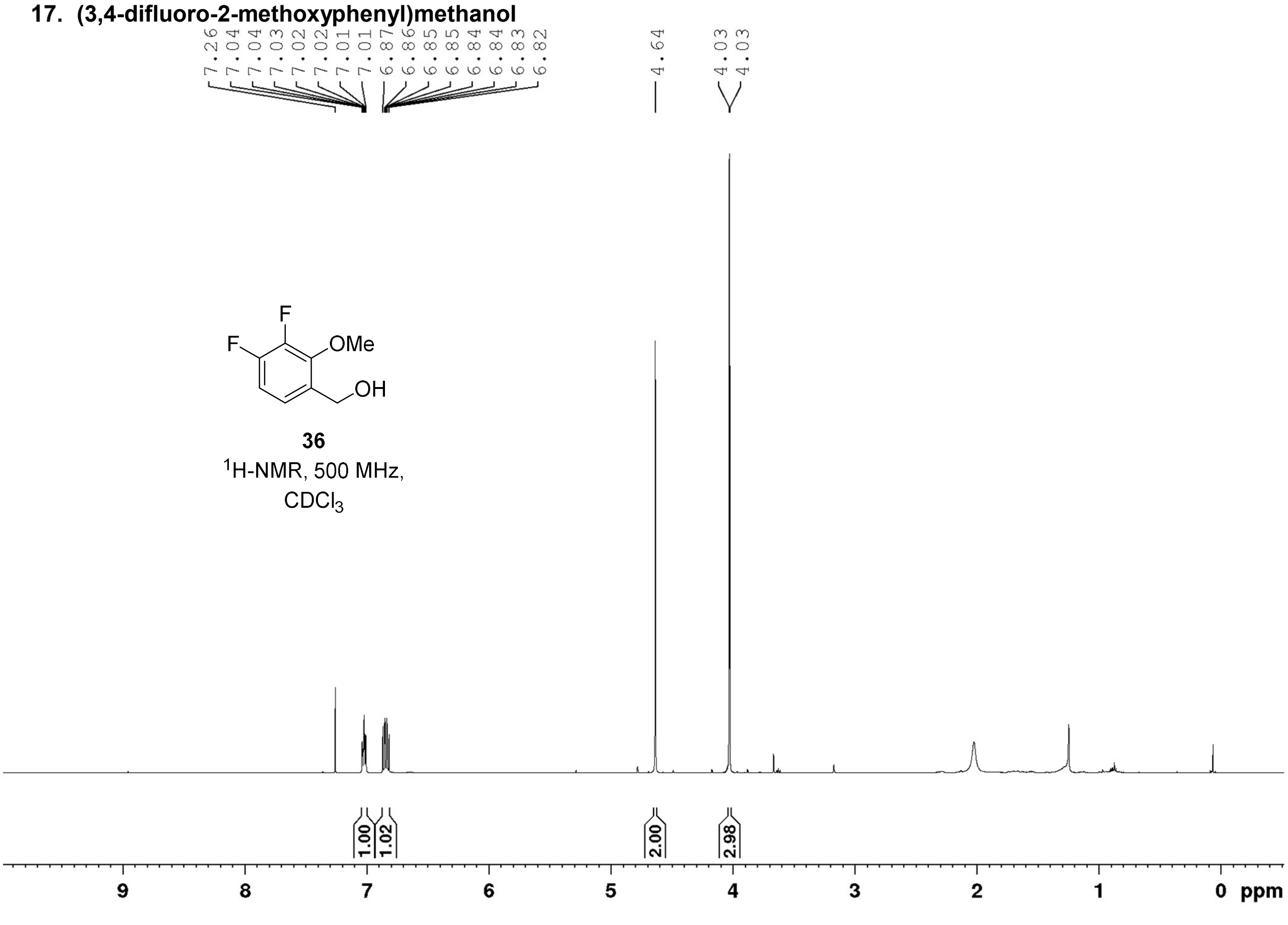

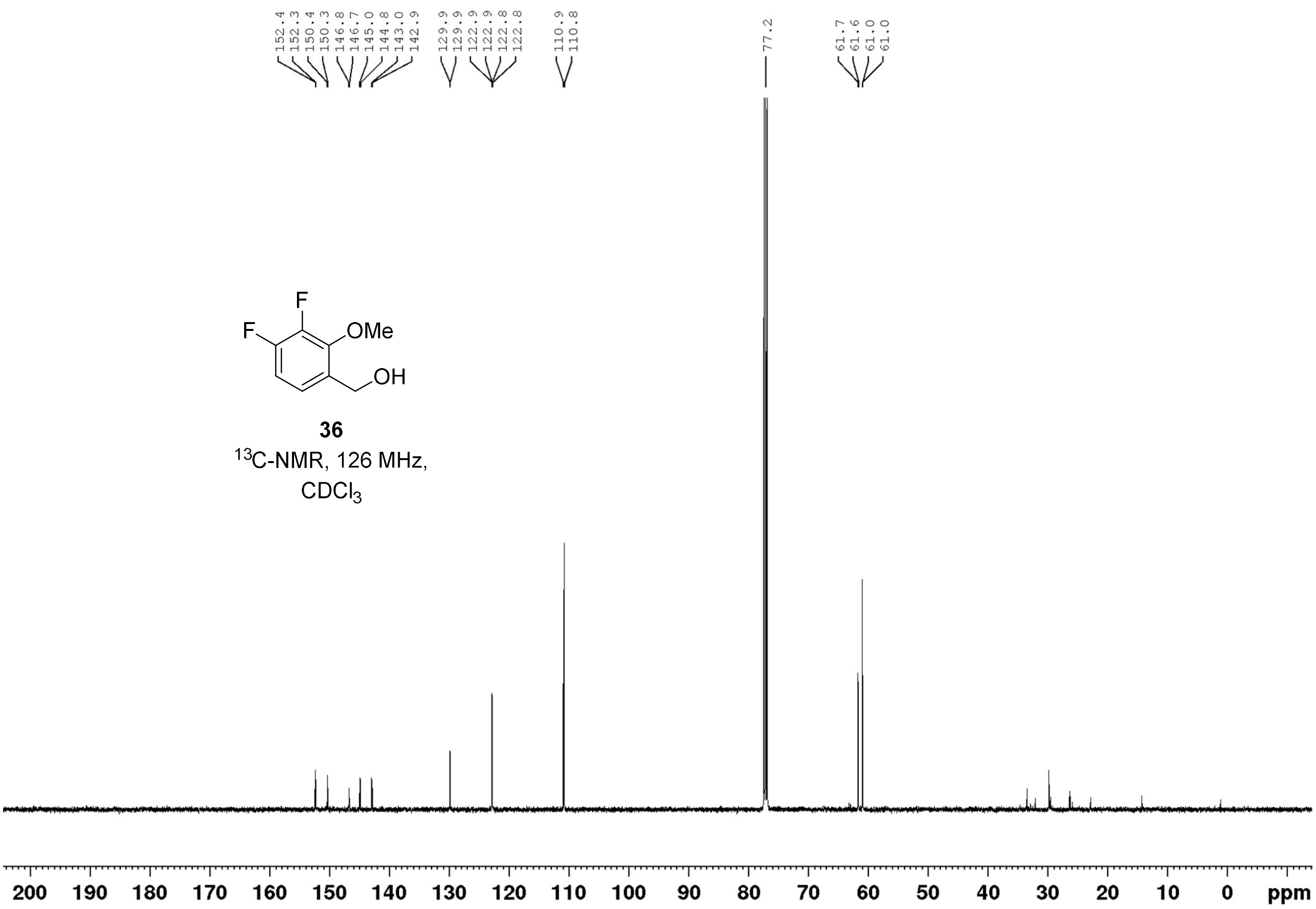
152.4
152.3
150.4
150.3
146.8
146.7
145.0
144.8
143.0
142.9
129.9
129.9
122.9
122.9
122.8
122.8
110.9
110.8
77.2
61.7
61.6
61.0
61.0
F
F
OMe
OH
36
^{13}C-NMR, 126 MHz,
$CDCl_3$
200 190 180 170 160 150 140 130 120 110 100 90 80 70 60 50 40 30 20 10 0 ppm

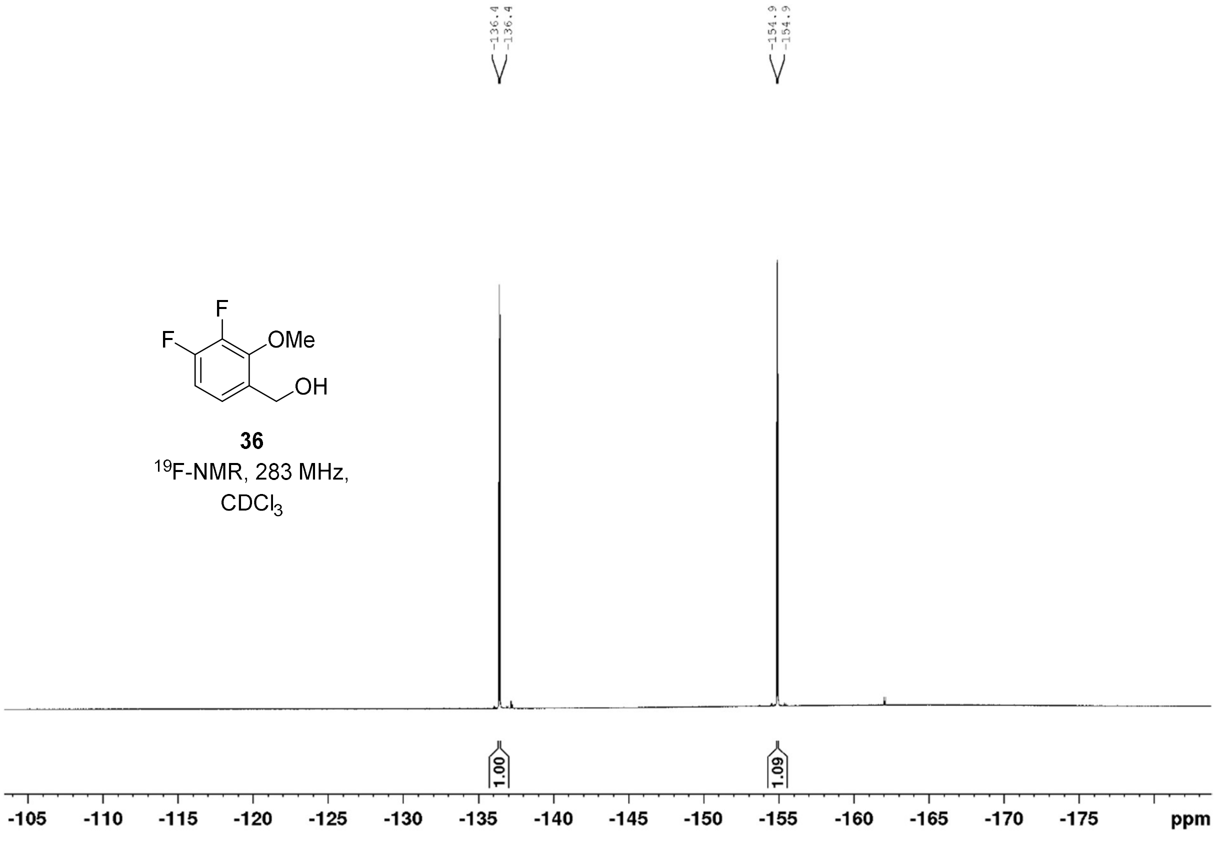
-136.4
-136.4
-154.9
-154.9
F
F
OMe
OH
36
^{19}F-NMR, 283 MHz,
$CDCl_3$
1.00
1.09
-105 -110 -115 -120 -125 -130 -135 -140 -145 -150 -155 -160 -165 -170 -175 ppm

18. 1-(bromomethyl)-3,4-difluoro-2-methoxybenzene

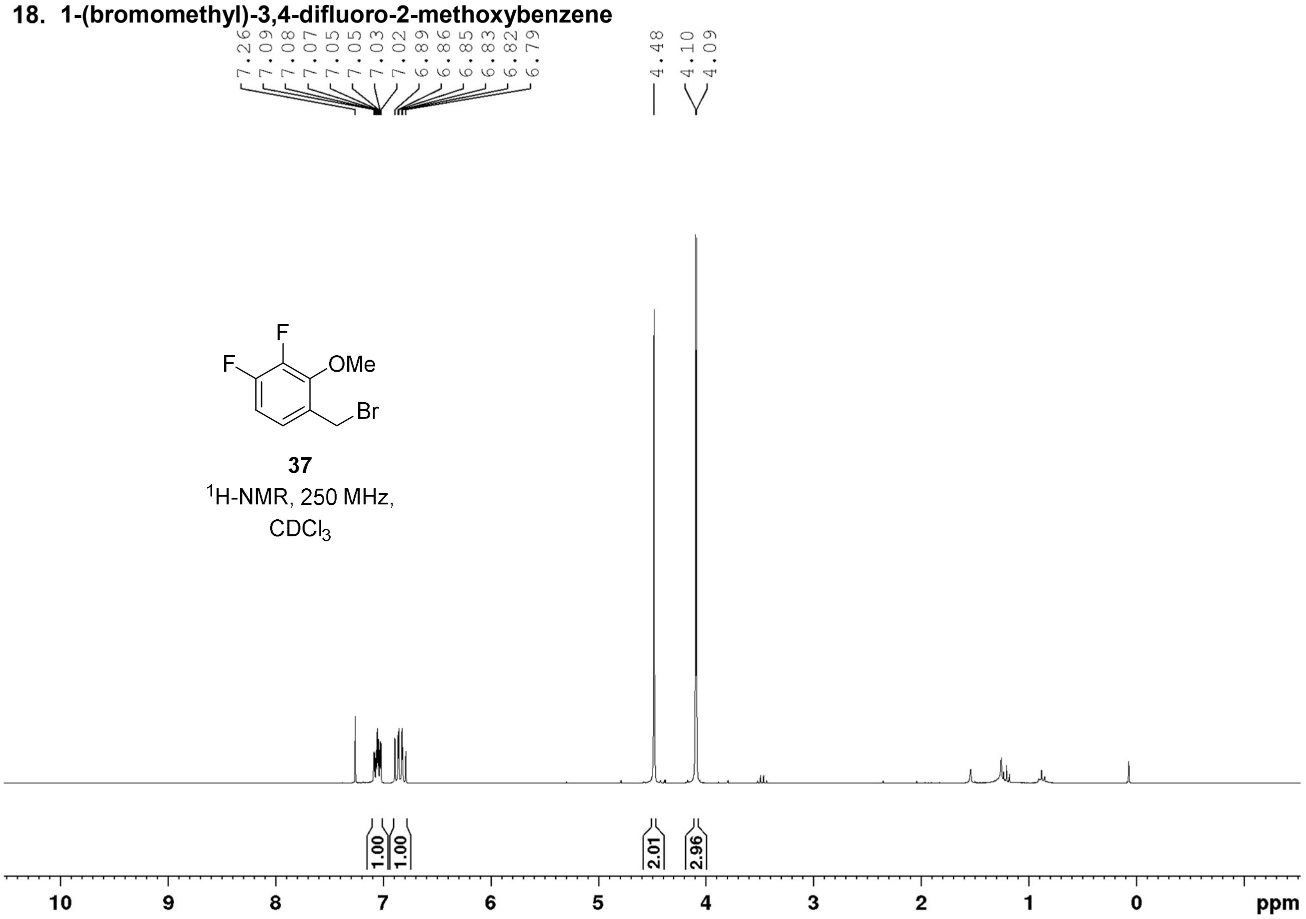

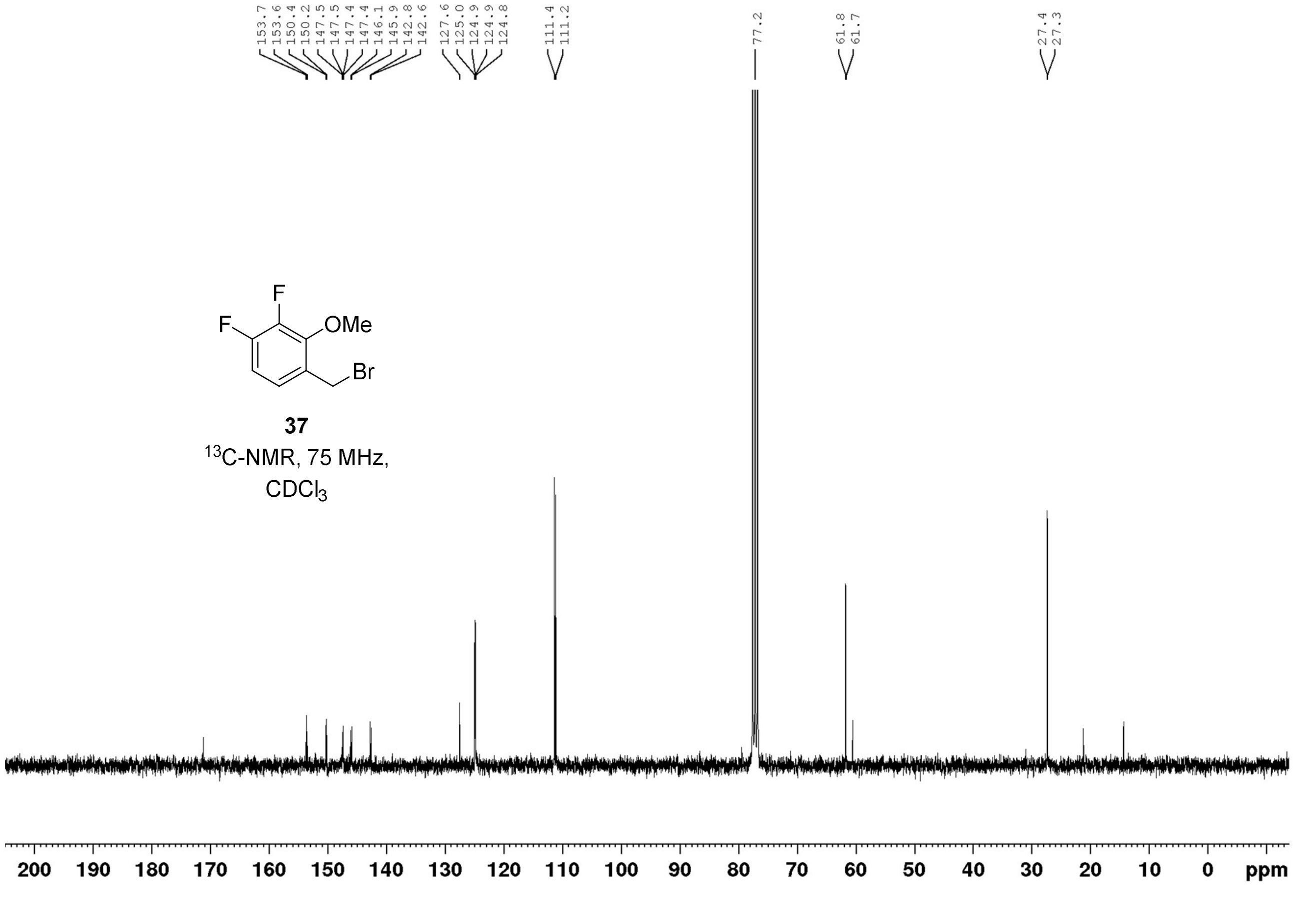

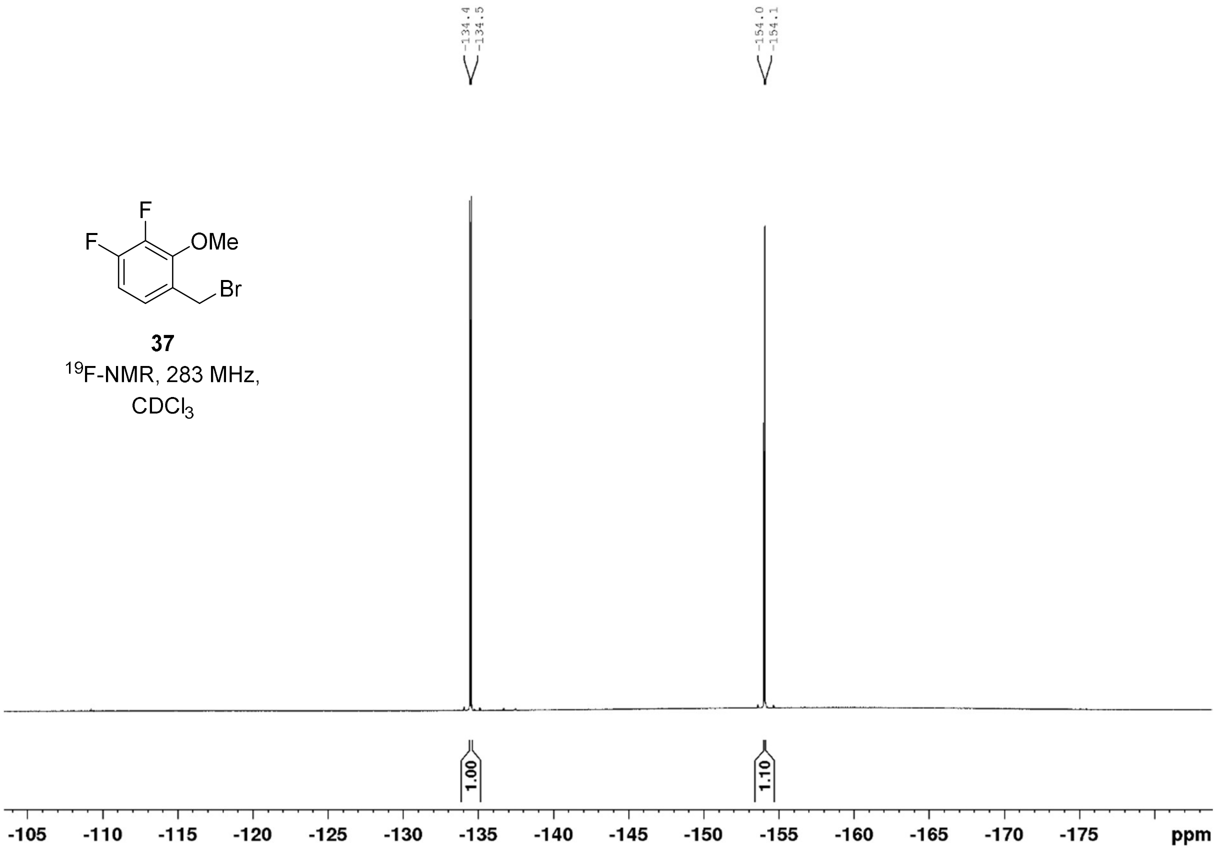

19. methyl 3-(3,4-difluoro-2-methoxybenzyl)-1,7,8-trifluoro-2-naphthoate

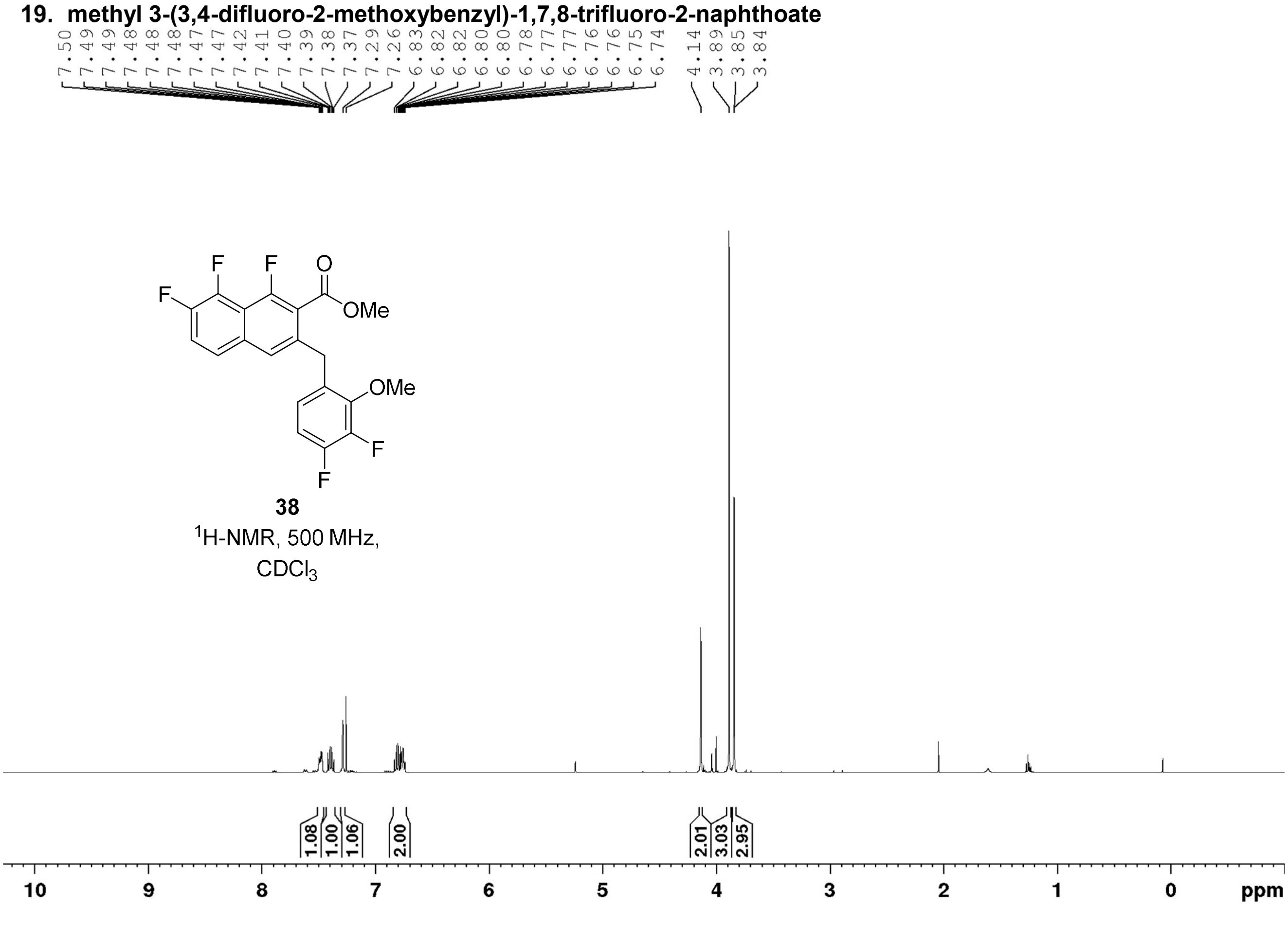

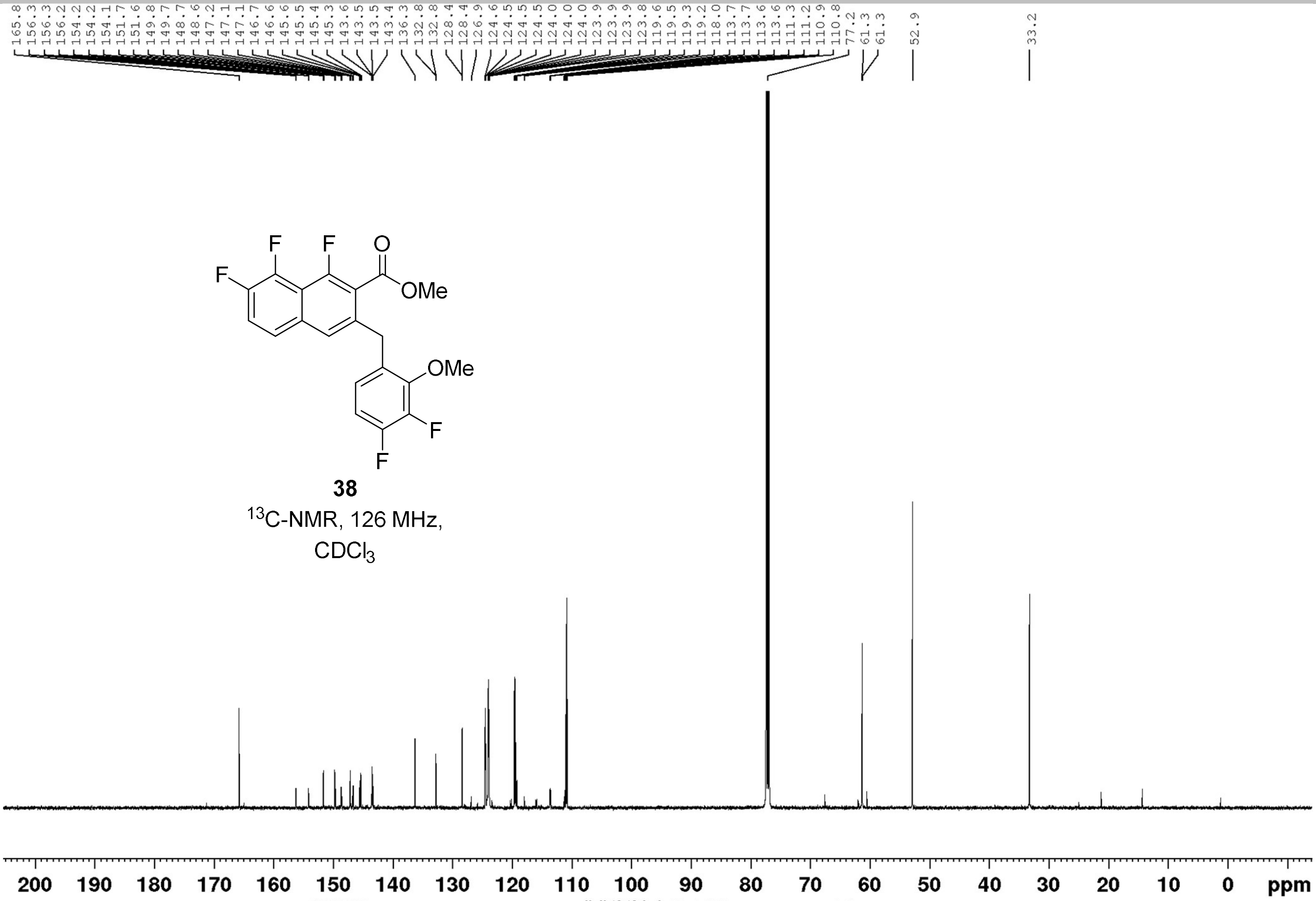
38
^{13}C-NMR, 126 MHz, $CDCl_3$
ppm

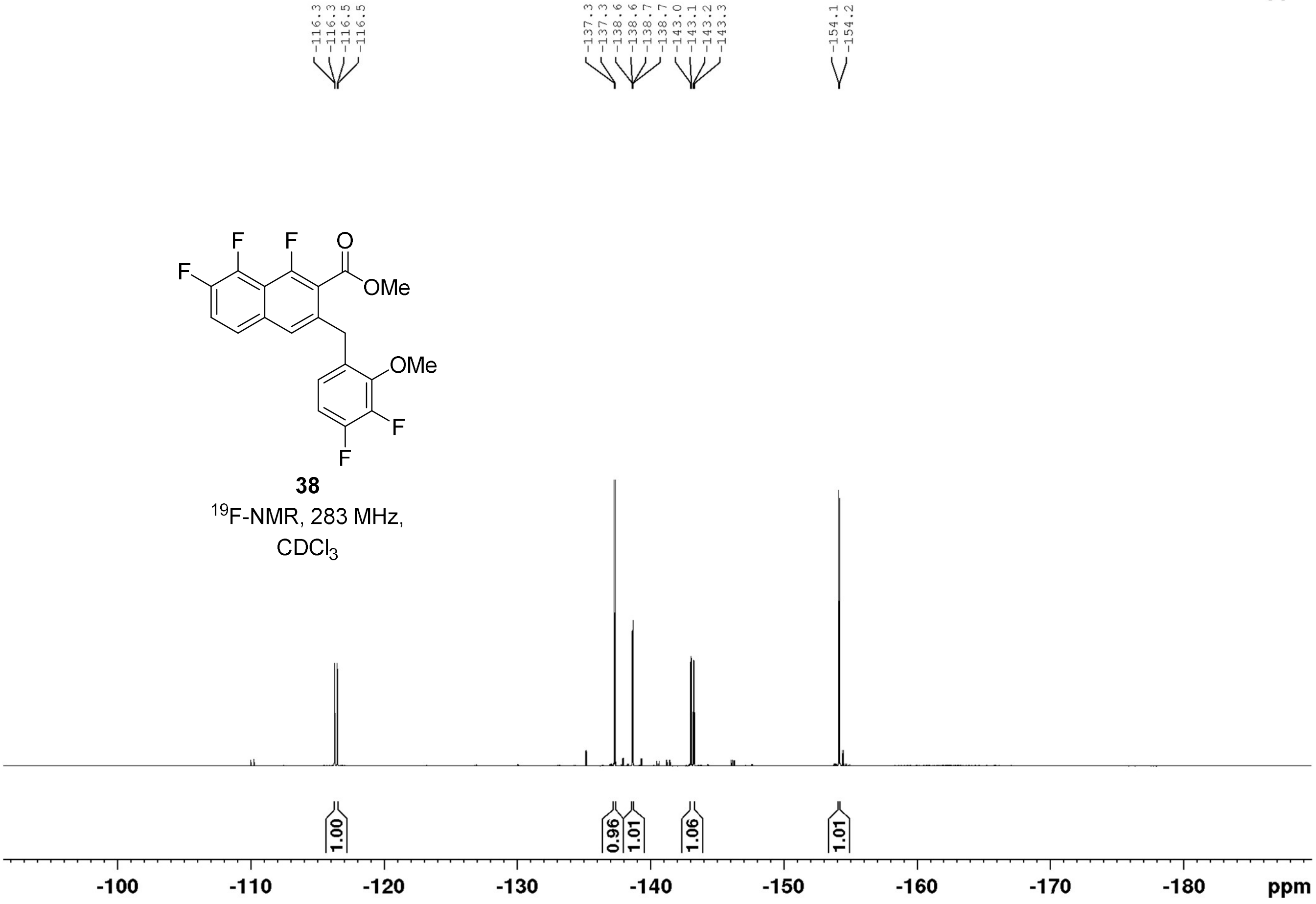
38
^{19}F-NMR, 283 MHz, $CDCl_3$
ppm

20. methyl 3-(3,4-difluoro-2-hydroxybenzyl)-1,7,8-trifluoro-2-naphthoate

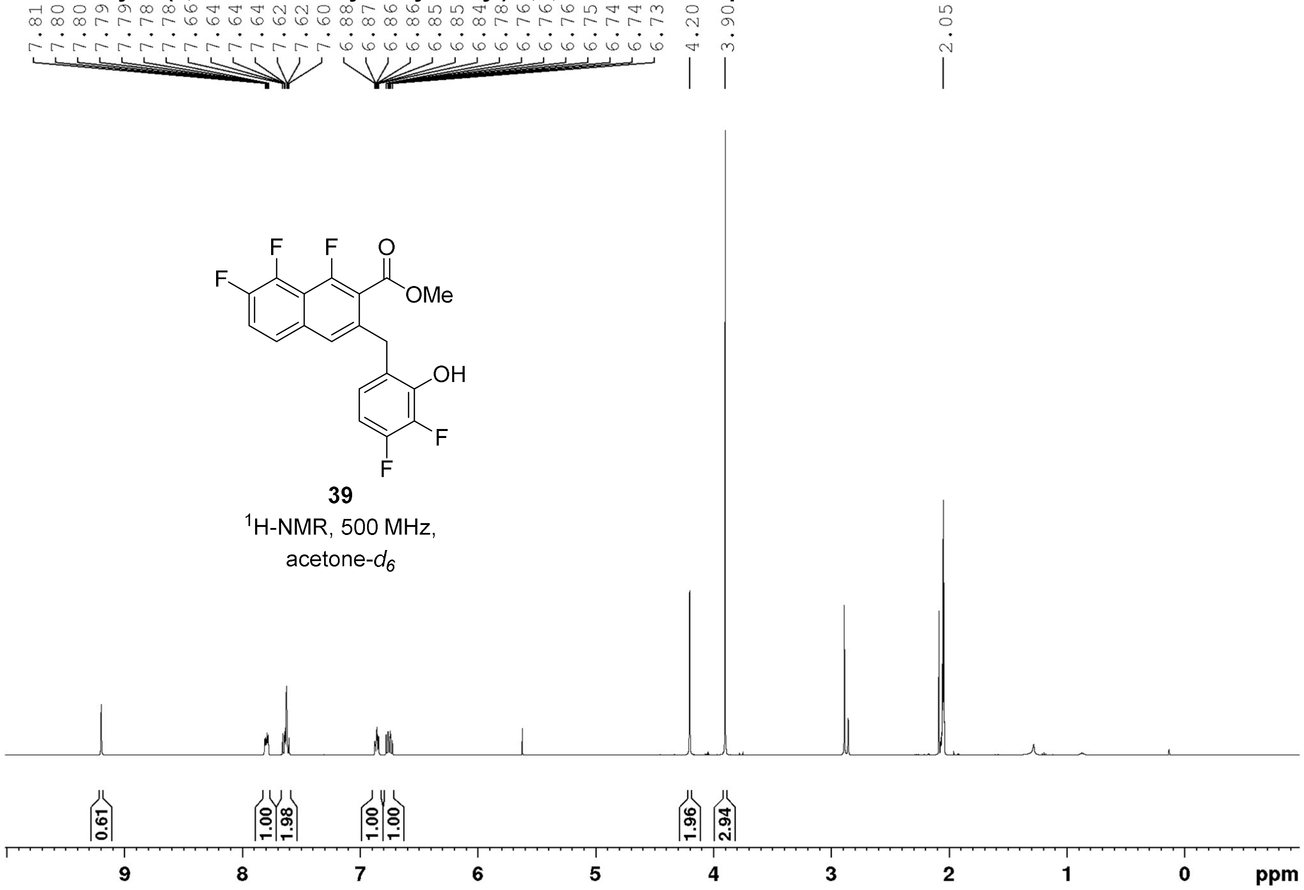

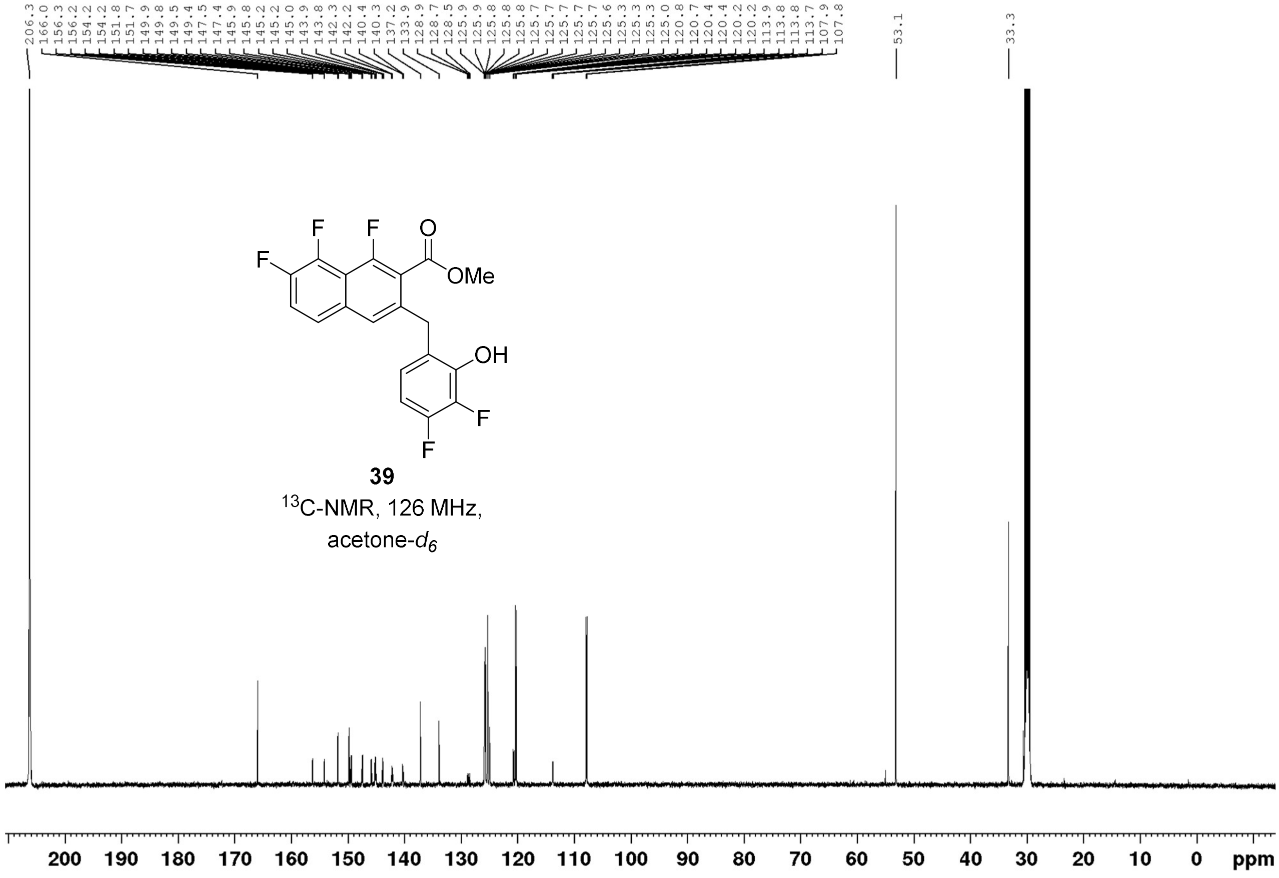

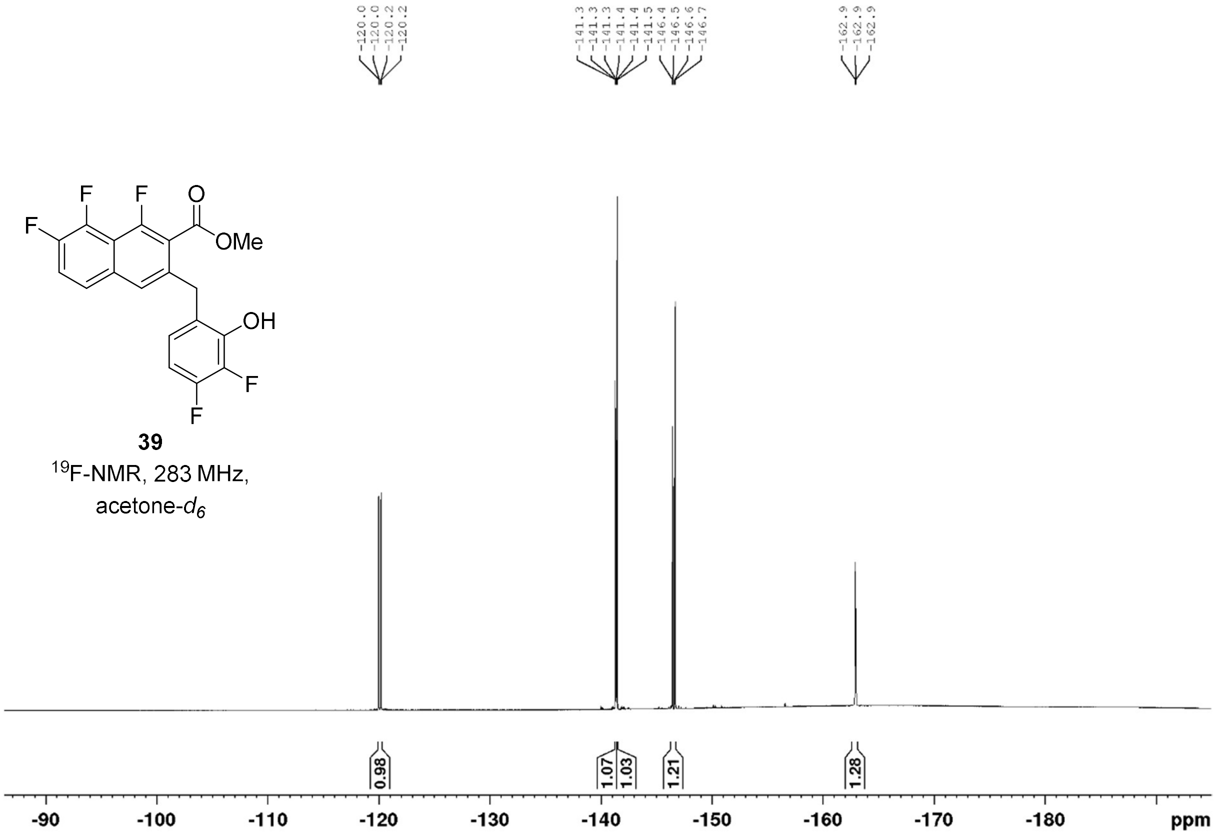

21. methyl 3-(3,4-difluoro-2-(((trifluoromethyl)sulfonyl)oxy)benzyl)-1,7,8-trifluoro-2-naphthoate

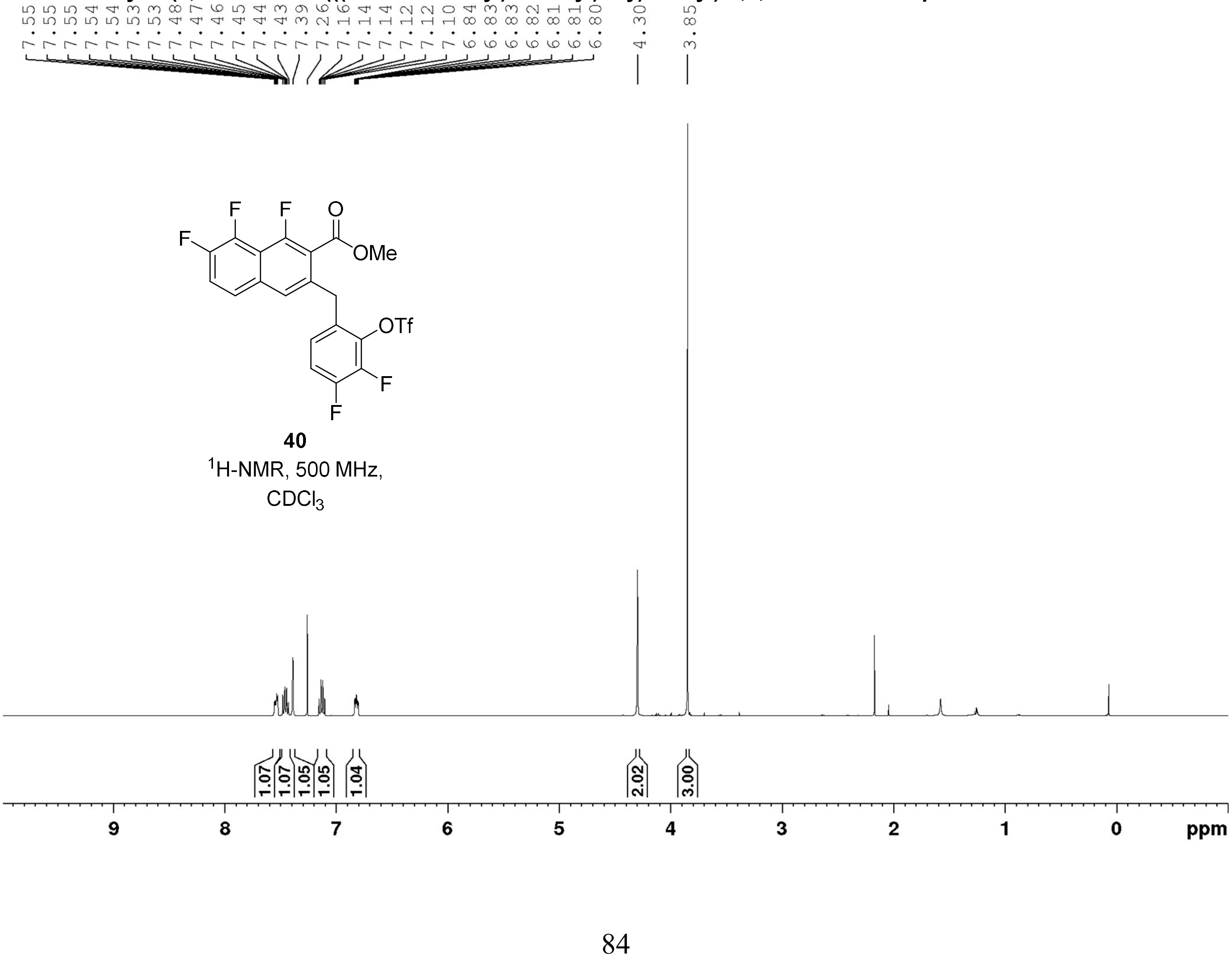

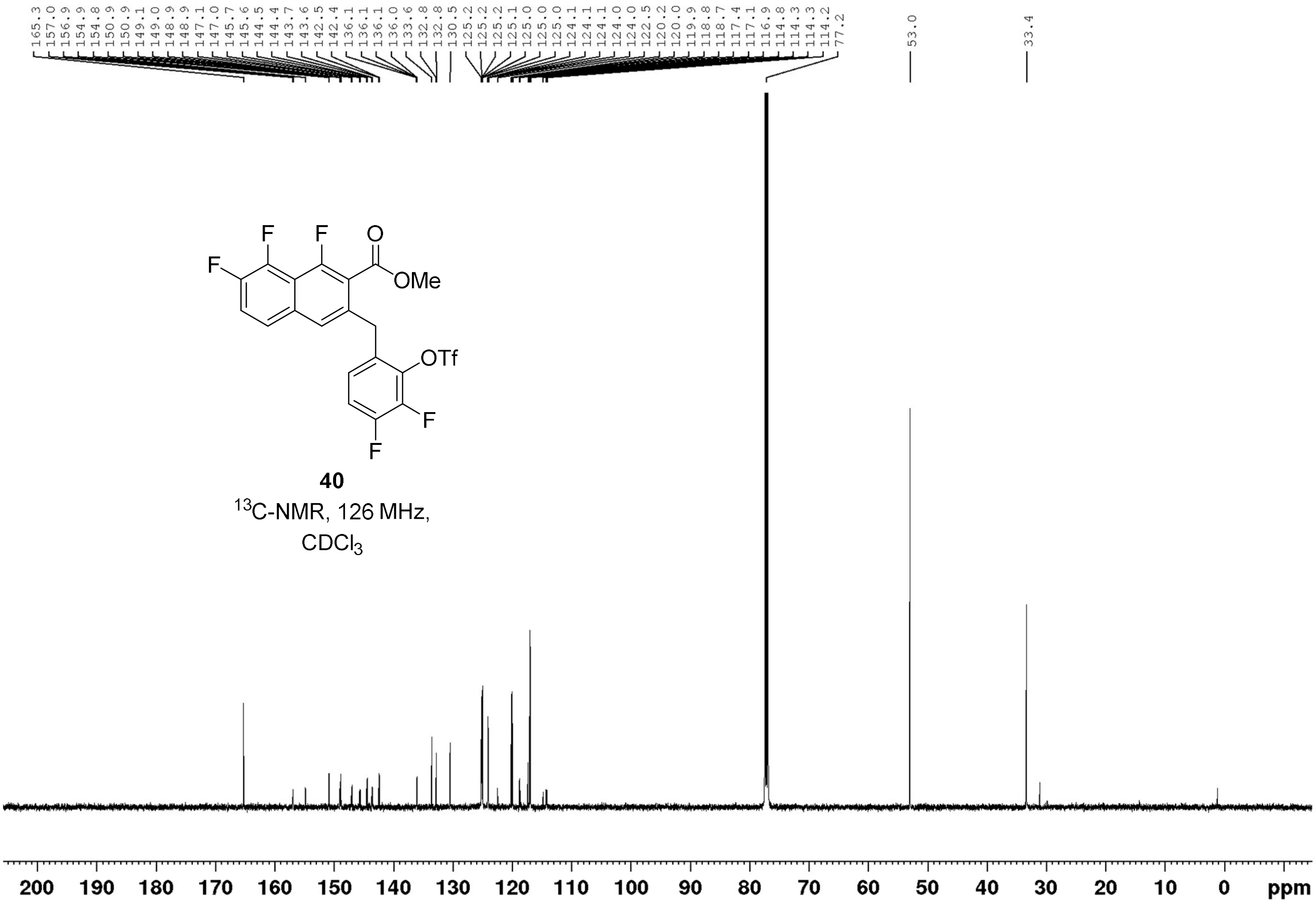
165.3
157.0
156.9
154.9
154.8
150.9
150.9
149.1
149.0
148.9
148.9
147.1
147.0
145.7
145.6
144.5
144.4
143.7
143.6
142.5
142.4
136.1
136.1
136.1
136.0
133.6
132.8
132.8
130.5
125.2
125.2
125.2
125.1
125.0
125.0
125.0
124.1
124.1
124.1
124.0
122.5
120.2
120.0
119.9
118.8
118.7
117.4
117.1
116.9
114.8
114.3
114.2
77.2
53.0
33.4
F
F
F
O
OMe
OTf
F
F
40
13C-NMR, 126 MHz,
CDCl3
200 190 180 170 160 150 140 130 120 110 100 90 80 70 60 50 40 30 20 10 0 ppm

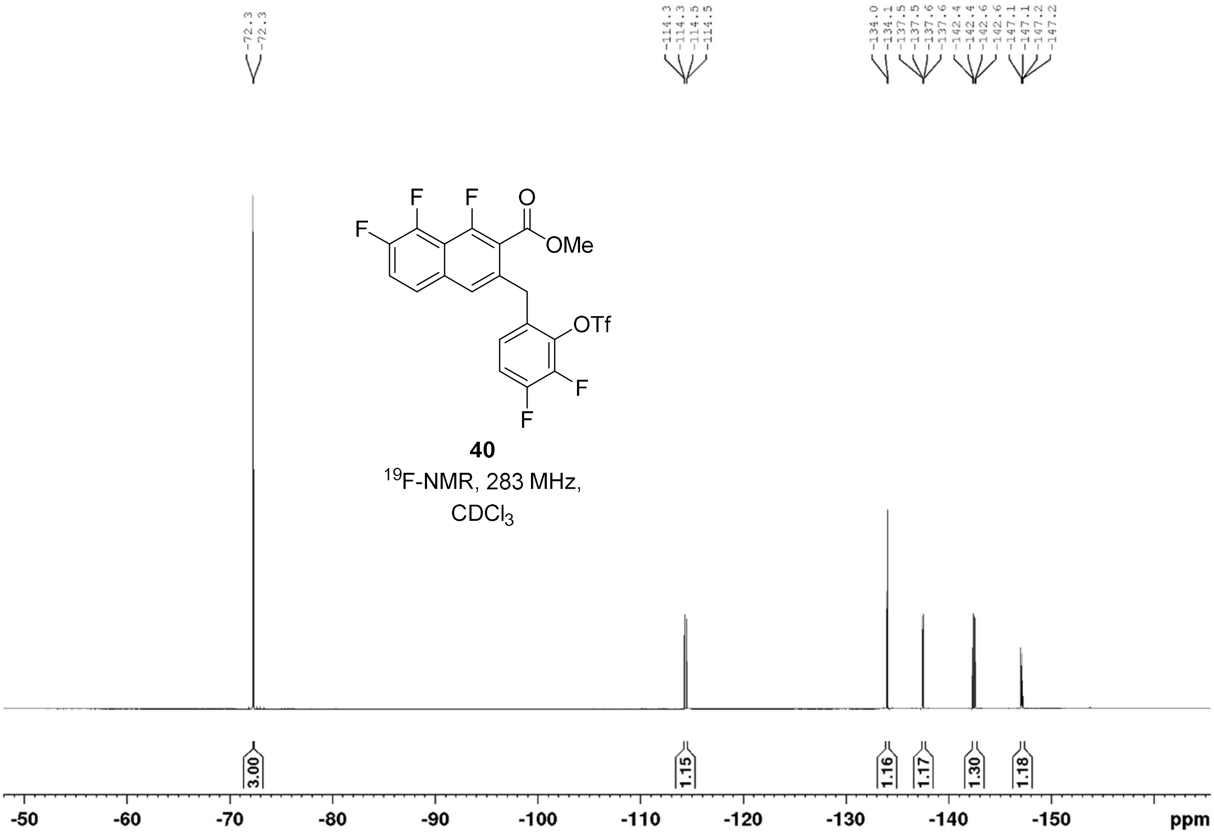
-72.3
-72.3
-114.3
-114.3
-114.5
-114.5
-134.0
-134.1
-137.5
-137.6
-142.4
-142.6
-147.1
-147.2
-147.2
F
F
F
O
OMe
OTf
F
F
40
19F-NMR, 283 MHz,
CDCl3
3.00
1.15
1.16
1.17
1.30
1.18
-50 -60 -70 -80 -90 -100 -110 -120 -130 -140 -150 ppm

22. 2,3-difluoro-6-((4,5,6-trifluoro-3-(hydroxymethyl)naphthalen-2-yl)methyl)phenyl trifluoromethanesulfonate

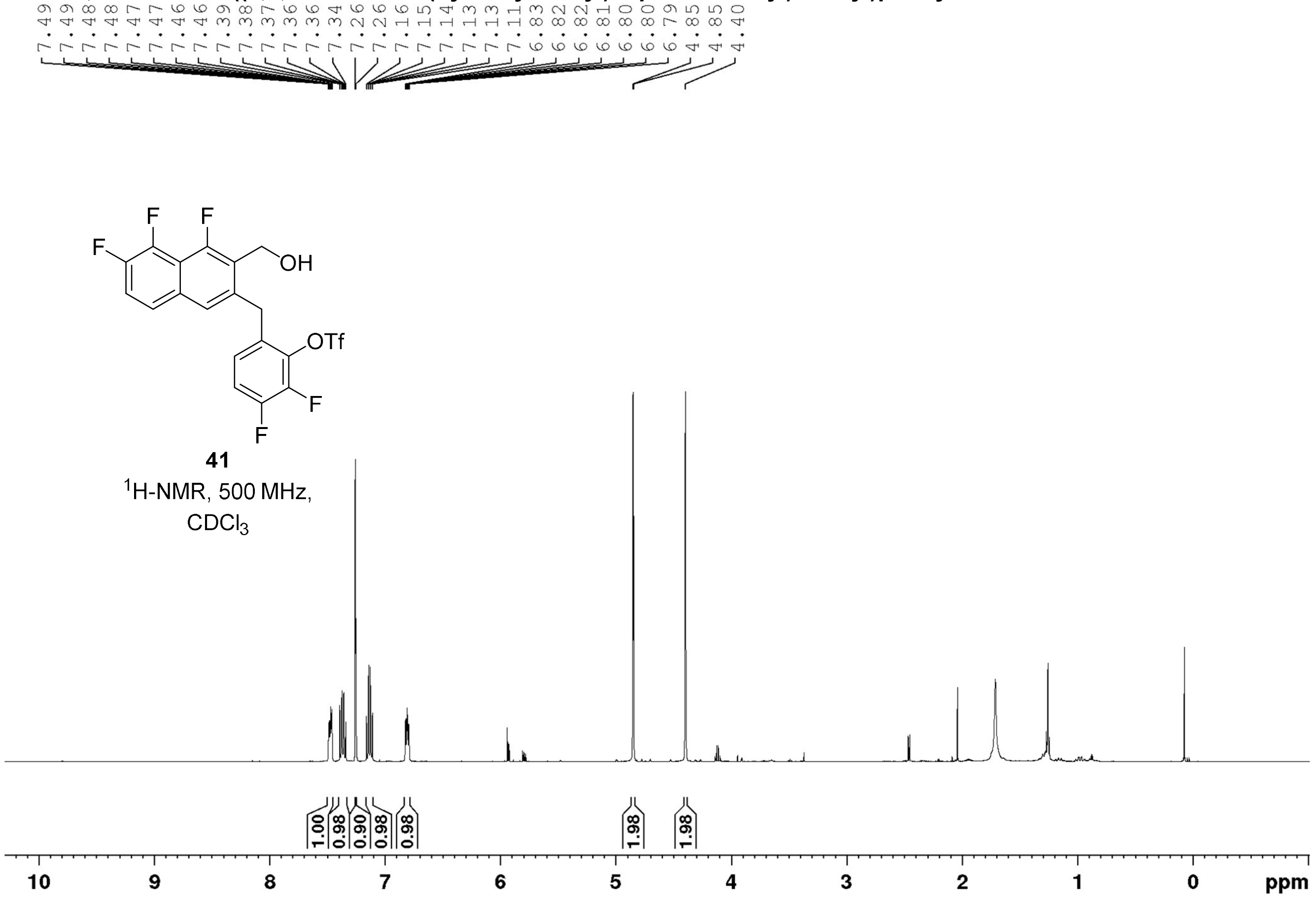

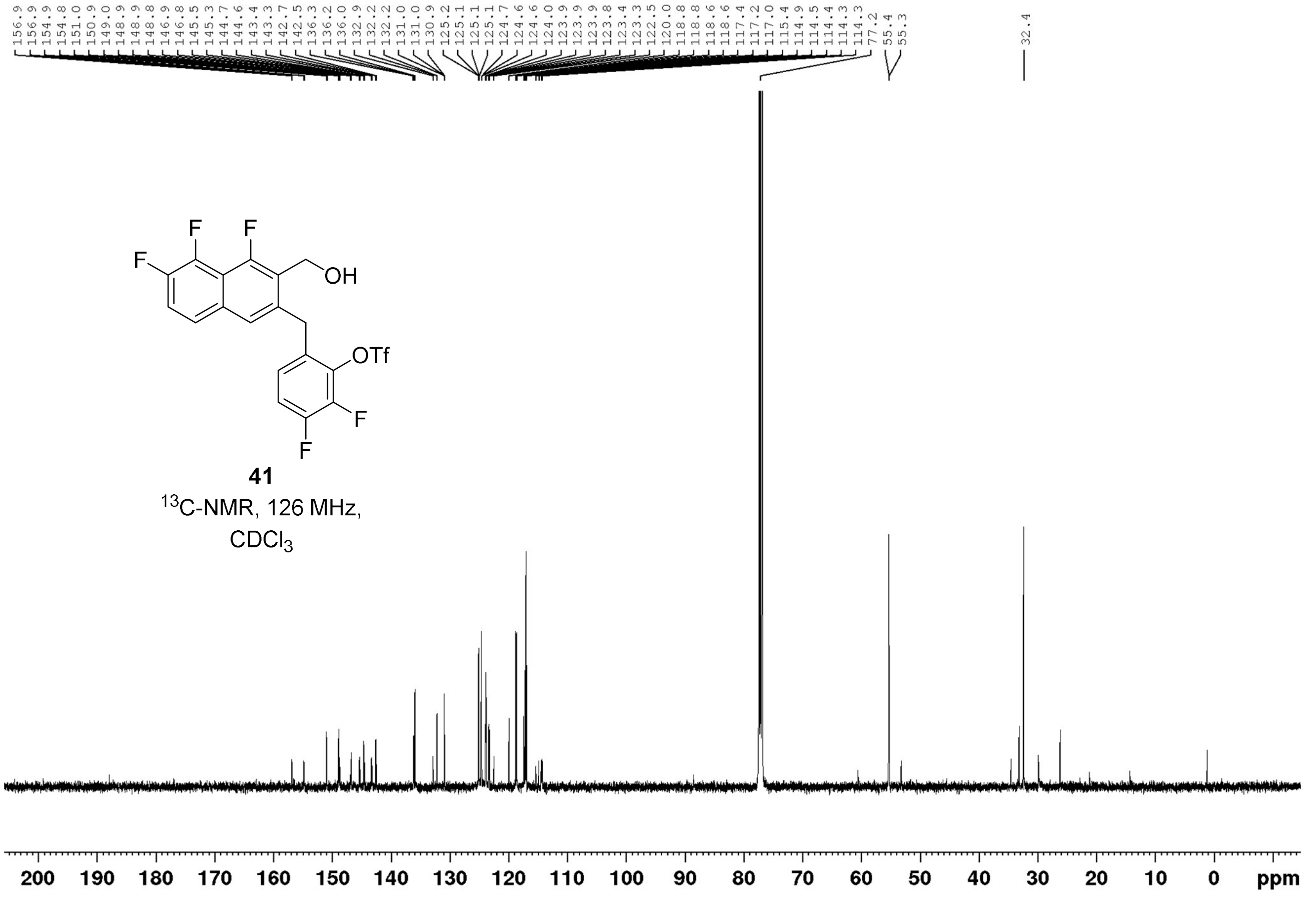

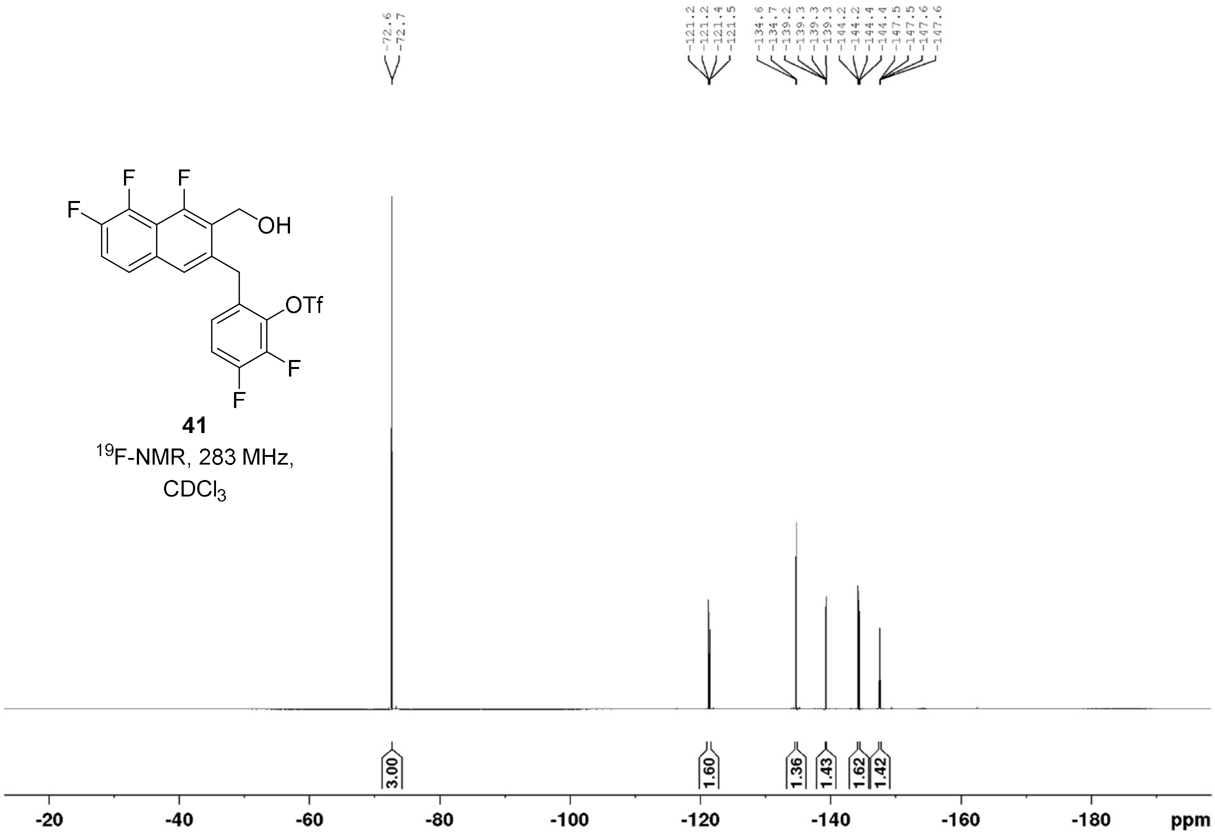

23. 2,3-difluoro-6-((4,5,6-trifluoro-3-formylnaphthalen-2-yl)methyl)phenyl trifluoromethanesulfonate

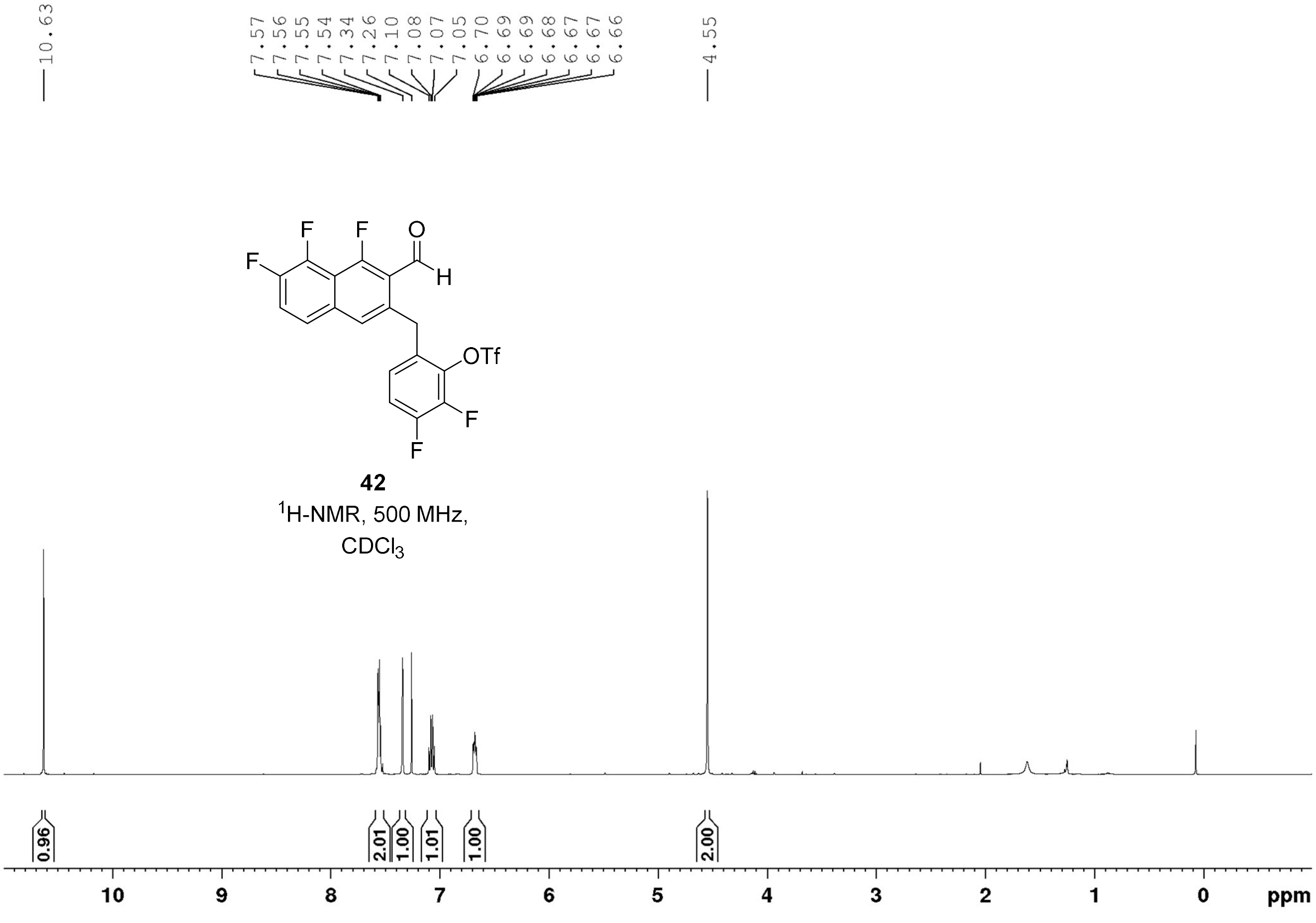

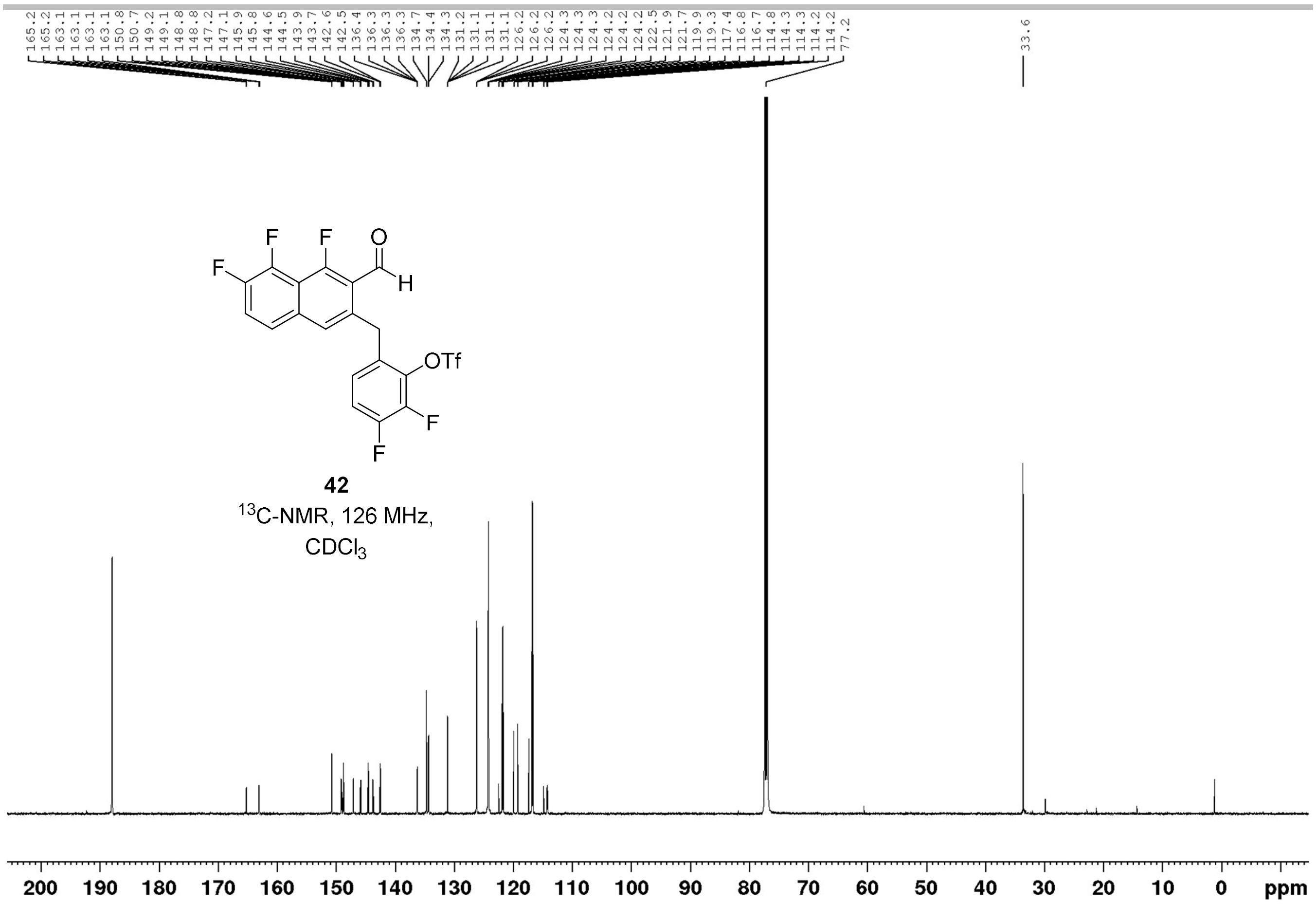

42
^{13}C-NMR, 126 MHz, $CDCl_3$

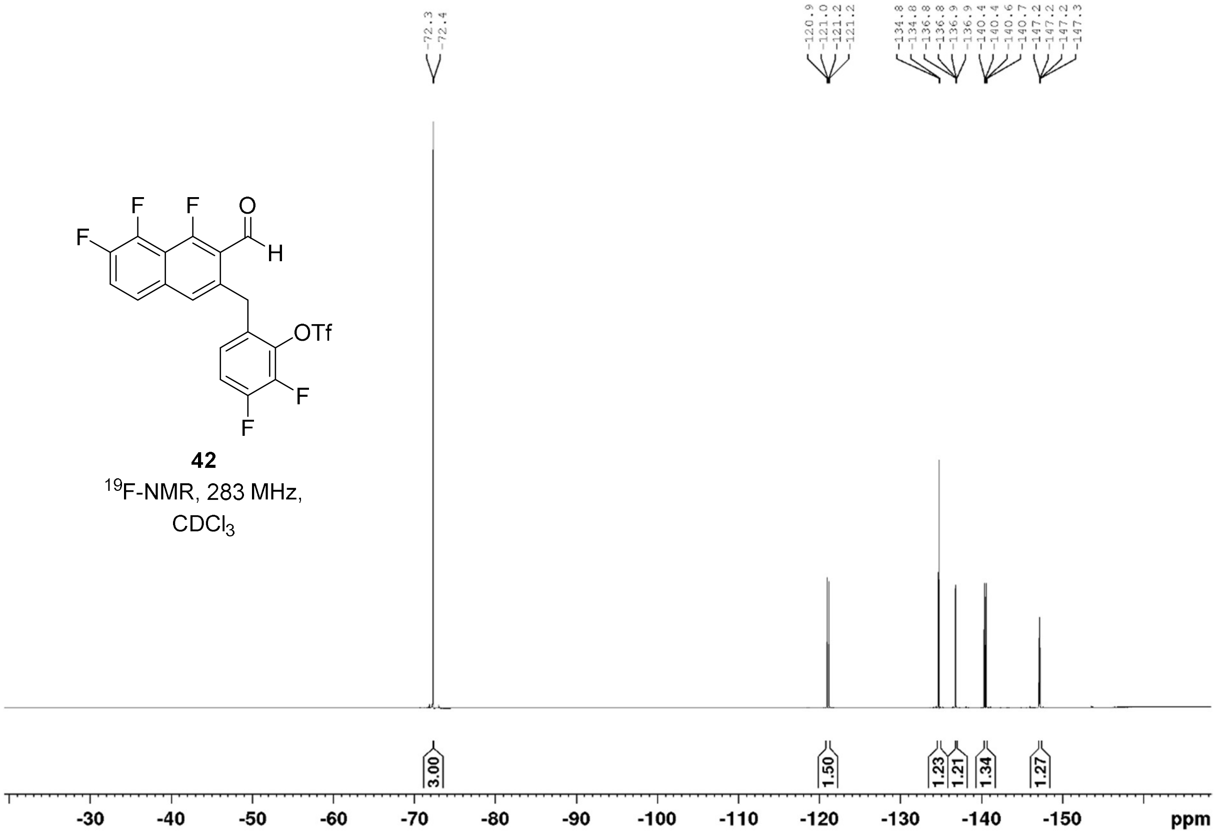

42
^{19}F-NMR, 283 MHz, $CDCl_3$

24. 3,4,6,7,8-pentafluoro-5,12-dihydrotetracen-5-ol

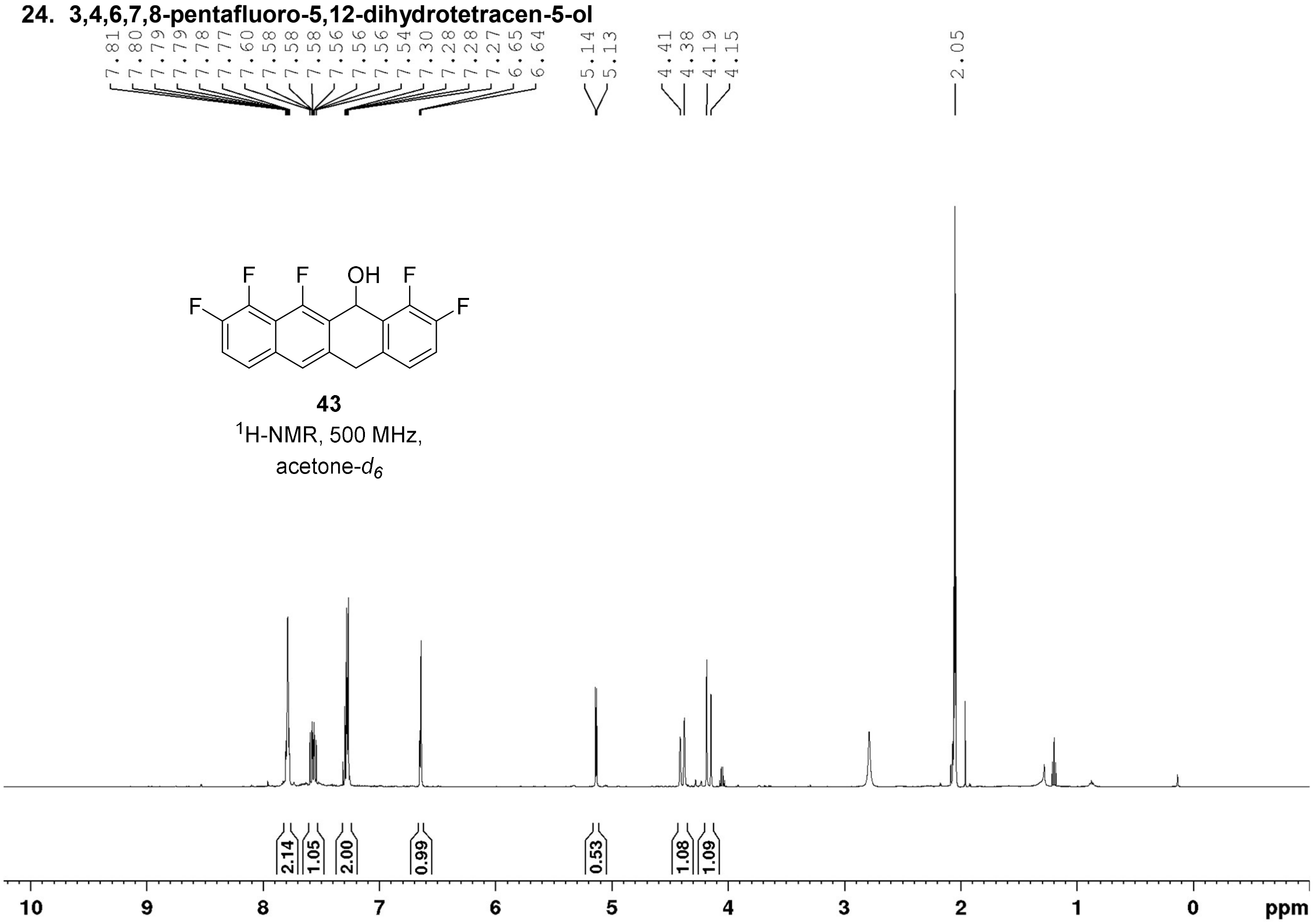

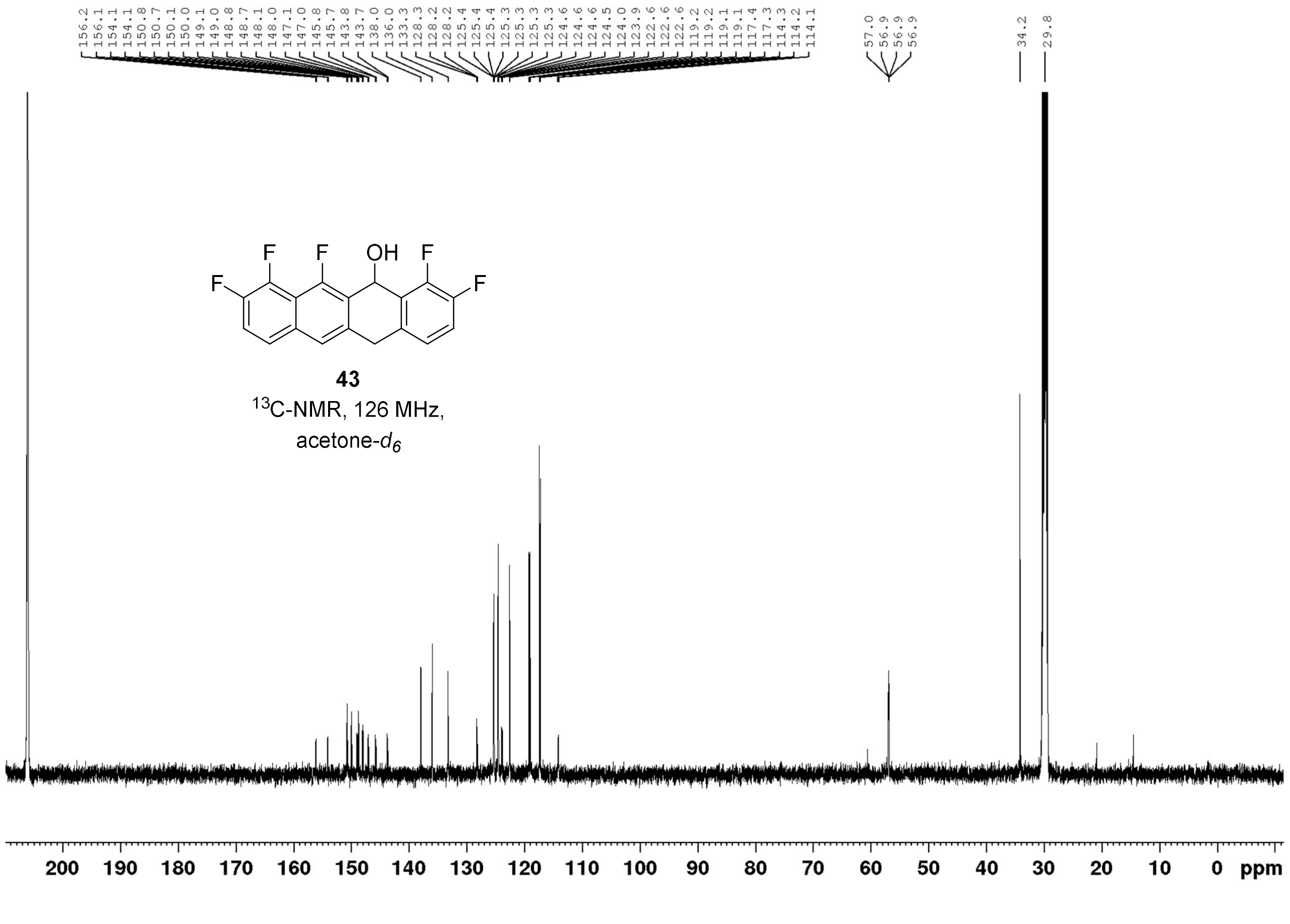

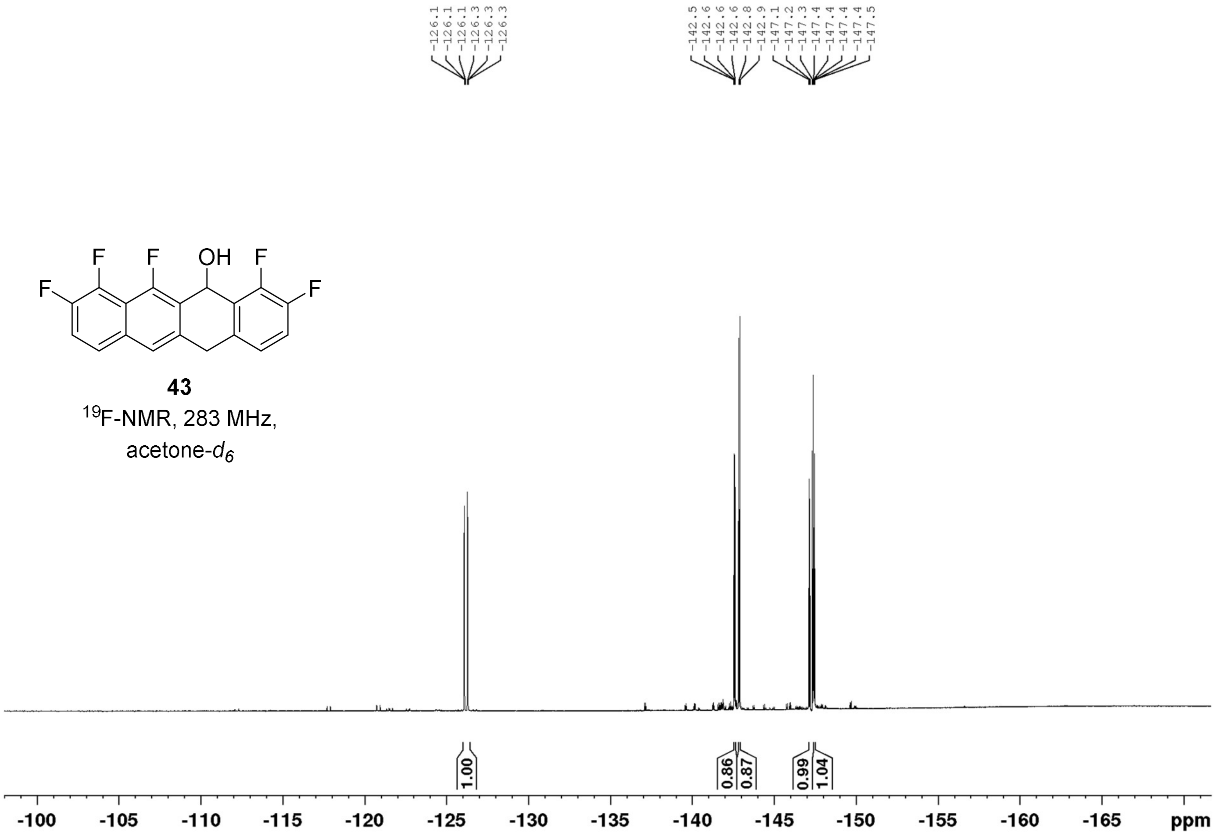

25. 1,2,9,10,11-pentafluorotetracene

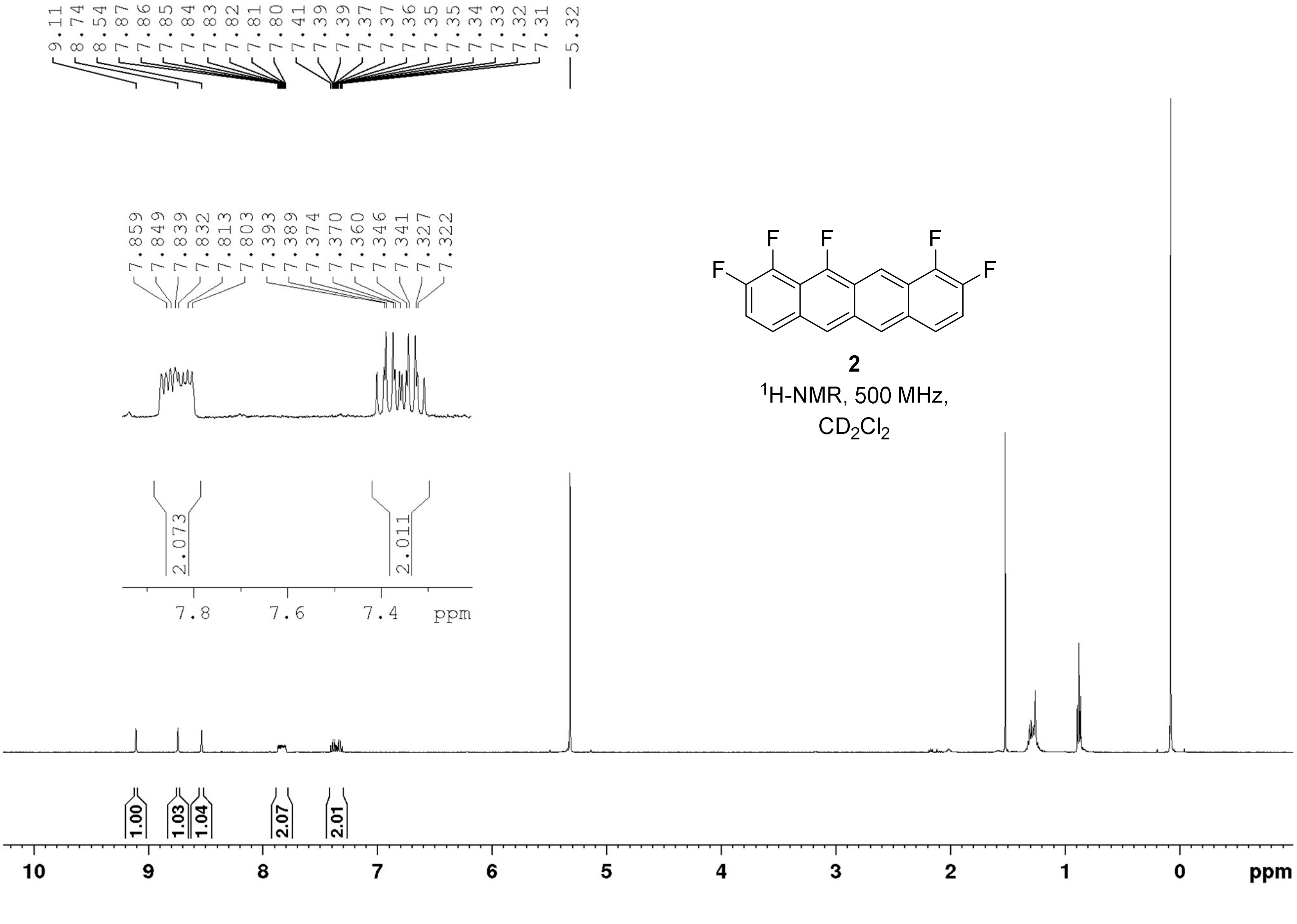

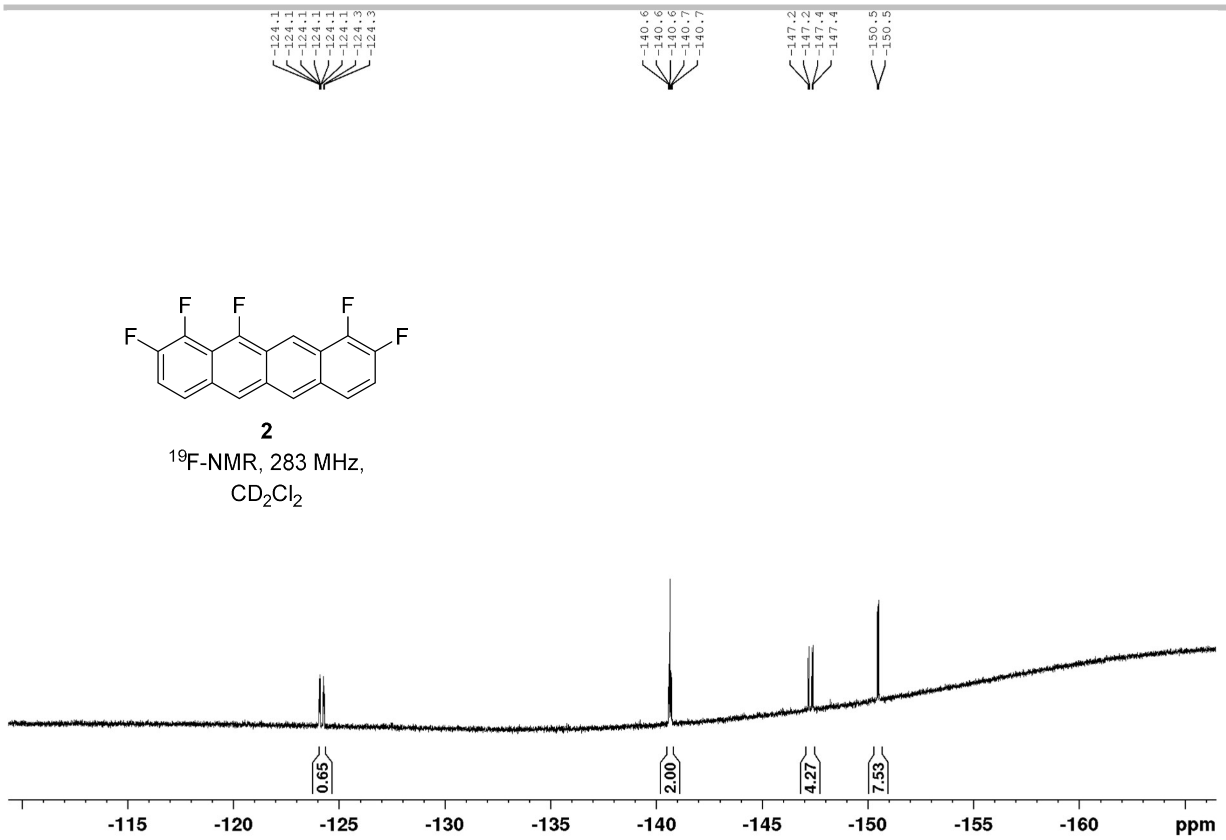
-124.1
-124.1
-124.1
-124.1
-124.1
-124.1
-124.3
-124.3
-140.6
-140.6
-140.6
-140.7
-140.7
-147.2
-147.2
-147.4
-147.4
-150.5
-150.5
F
F
F
F
F
F
2
^{19}F-NMR, 283 MHz,
CD_2Cl_2
0.65
2.00
4.27
7.53
-115
-120
-125
-130
-135
-140
-145
-150
-155
-160
ppm

12. References

[1] A. Pick, M. Klues, A. Rinn, K. Harms, S. Chatterjee, G. Witte, *Cryst. Growth Des.* **2015**, *15*, 5495-5504.
[2] T. Takeyama, S. Maruyama, Y. Matsumoto, *Cryst. Growth Des.* **2011**, *11*, 2273-2278.
[3] I. Meyenburg, T. Breuer, A. Karthäuser, S. Chatterjee, G. Witte, W. Heimbrodt, *Phys. Chem. Chem. Phys.* **2016**, *18*, 3825-3831.
[4] T. Breuer, M. Klues, G. Witte, *J. Electron. Spectrosc.* **2015**, *204*, 102-115.
[5] X-Area, STOE & Cie GmbH, Darmstadt, Germany, **2018**.
[6] G. M. Sheldrick, *Acta Crystallogr., Sect. A: Found. Adv.* **2015**, *71*, 3–8.
[7] G. M. Sheldrick, *Acta Crystallogr., Sect. C: Struct. Chem.* **2015**, *71*, 3–8.
[8] WinXPOW, STOE & Cie GmbH, Darmstadt, Germany, **2018**.
[9] A. A. Coelho, *J. Appl. Crystallogr.* **2018**, *51*, 210–218.
[10] D. Quintard, M. Keller, B. Breit, *Synthesis* **2004**, *6*, 905-908.
[11] F. Zhang, L. Wang, S.-H. Chang, K.-L. Huang, Y. Chi, W.-Y. Hung, C.-M. Chen, G.-H. Lee, P. T. Chou, *Dalton Trans.* **2013**, *42*, 7111-7119.
[12] K. Araki, T. Katagiri, M. Inoue, *J. Fluor. Chem.* **2014**, *157*, 41-47.
[13] a) J. K. Vandavasi, X. Hua, H. B. Halima, S. G. Newman, *Angew. Chem. Int. Ed.* **2017**, *56*, 15441-15445; b) T. Verheyen, L. van Turnhout, J. K. Vandavasi, E. S. Isbrandt, W. M. De Borggraeve, S. G. Newman, *J. Am. Chem. Soc.* **2019**, *141*, 6869-6874.
[14] K. J. Garcia, M. M. Gilbert, D. J. Weix, *J. Am. Chem. Soc.* **2019**, *141*, 1823-1827.
[15] T. Moragas, A. Correa, R. Martin, *Chem. Eur. J.* **2014**, *20*, 8242-8258.
[16] M. Presset, J. Paul, G. N. Cherif, N. Ratnam, N. Laloi, E. Léonel, C. Gosmini, E. Le Gall, *Chem. Eur. J.* **2019**, *25*, 4491-4495.
[17] For activated carbonyls see T. Fujihara, K. Nogi, T. Xu, J. Terao, Y. Tsuji, *J. Am. Chem. Soc.* **2012**, *134*, 9106-9109.
[18] For directed CH-activation and addition to aldehydes see Y. Li, X.-S. Zhang, K. Chen, K.-H. He, F. Pan, B.-J. Li, Z.-J. Shi, *Org. Lett.* **2012**, *14*, 636-639.
[19] K. Harms, P. E. Hofmann, U. Koert CCDC 2001008: Experimental Crystal Structure Determination, **2020**, DOI: 10.5517/ccdc.csd.cc2556n7
[20] V. Oja, E. M. Suuberg, *J. Chem. Eng. Data* **1998**, *43*, 486-492.
[21] M. W. Schmidt, K. K. Baldridge, J. A. Boatz, S. T. Elbert, M. S. Gordon, J. H. Jensen, S. Koseki, N. Matsunaga, K. A. Nguyen, S. Su, T. L. Windus, M. Dupuis, J. A. Montgomery, *J. Comput. Chem.* **1993**, *14*, 1347-1363.
[22] M. S. Gordon, M. W. Schmidt, Advances in Electronic Structure Theory: GAMESS a Decade Later. In Theory and Applications of Computational Chemistry: The Firs*t Forty Years*; C. E. Dykstra, G. Frenking, K. S. Kim, G. E. Scuseria, Eds.; Elsevier: Amsterdam, The Netherlands, **2005**; Chapter *41*, pp 1167-1189.
[23] B. M. Bode, M. S. Gordon, *J. Mol. Graphics Mod.,* **1998**, *16*, 133-138.
[24] U. Varetto, Molekel 5.4, Swiss National Supercomputing Centre: Lugano Switzerland, 2009.
[25] M. Klues, G. Witte, *CrystEngComm* **2018**, *20*, 63-74.
[26] R. B. Campbell, J. M. Robertson, *Acta Cryst.* **1962**, *15*, 289-290.
[27] Y. Sakamoto, T. Suzuki, Y. G. Kobayashi, Y. Fukai, Y. Inoue, F. Sato, S. Tokito, *J. Am. Chem. Soc.* **2004**, *126*, 8138-8140.
[28] M. A. Spackman, D. Jayatilaka, *CrystEngComm*, **2009**, *11*, 19-32
[29] M. J. Turner, J. J. McKinnon, S. K. Wolff, D. J. Grimwood, P. R. Spackman, D. Jayatilaka, M. A. Spackman, *CrystalExplorer17*; University of Western Australia, **2017**.
[30] I. Salzmann, A. Moser, M. Oehzelt, T. Breuer, X. Feng, Z.-Y. Juang, D. Nabok, R. G. Della Valla, S. Duhm, G. Heimel, A. Brillante, E. Venuti, I. Bilotti, C. Christodoulou, J. Frisch, P. Puschnig, C. Draxl, G. Witte, K. Müllen, N. Koch, *ACS Nano* **2012**, *6*, 10874-10883.

Author Contributions

G.W. and U.K. planned the study and supervised the experiments. P.E.H., M.W.T., Y.G. and A.L.C.S. synthesized the hexafluoropentacene and M.W.T. synthesized the pentafluorotetracene based on the developed synthetic method. T.B. performed the NEXAFS measurements. D.B. grew the single crystals and performed the UV/Vis and sublimation measurements as well as all DFT calculations and the Hirshfeld surface analysis. S.I.I. conducted the crystal structure analysis. U.K. and G.W. wrote the manuscript and all authors gave their approval to the final manuscript.
